# Supplementary figures and images for: Genome-wide screen reveals Rab12 GTPase as a critical activator of Parkinson’s disease-linked LRRK2 kinase (part 1 of 2)
Source: eLife. 2023 Oct 24;12:e87098. doi: 10.7554/eLife.87098 (PMC10708890; doi:10.7554/eLife.87098)

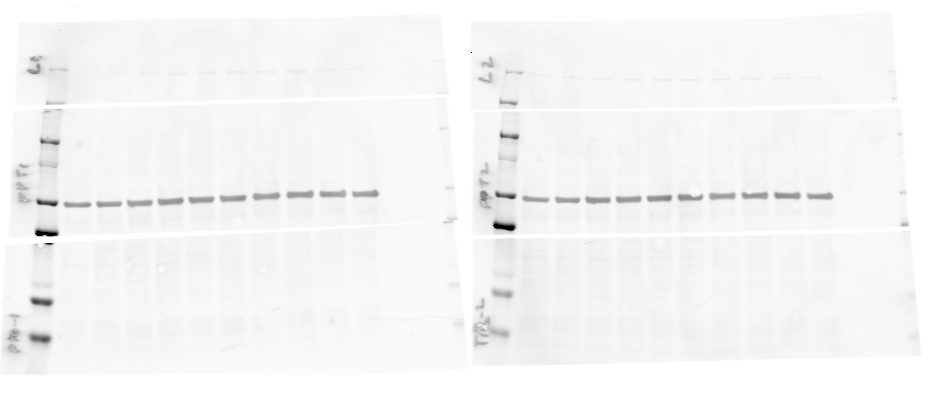

Supplement: Figure 2—source data 1. [file elife-87098-fig2-data1.zip › Figure 2A-source data 1/700.tif]

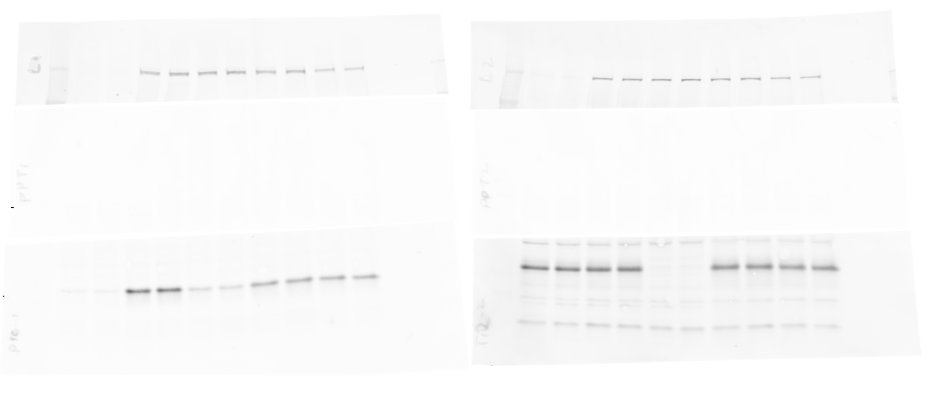

Supplement: Figure 2—source data 1. [file elife-87098-fig2-data1.zip › Figure 2A-source data 1/800_1.tif]

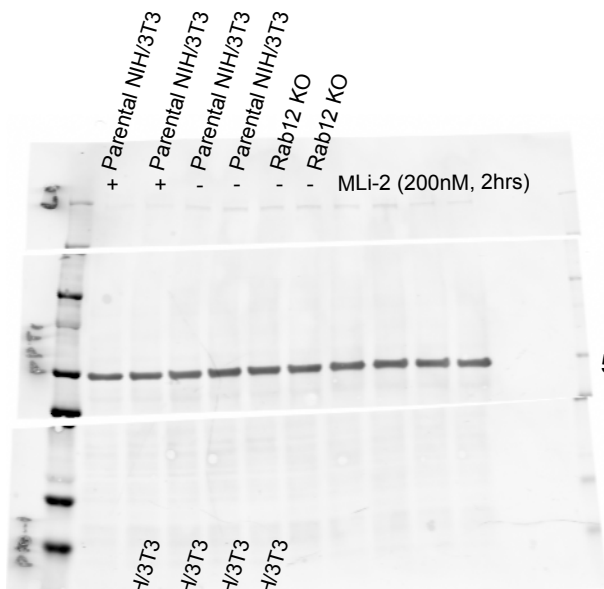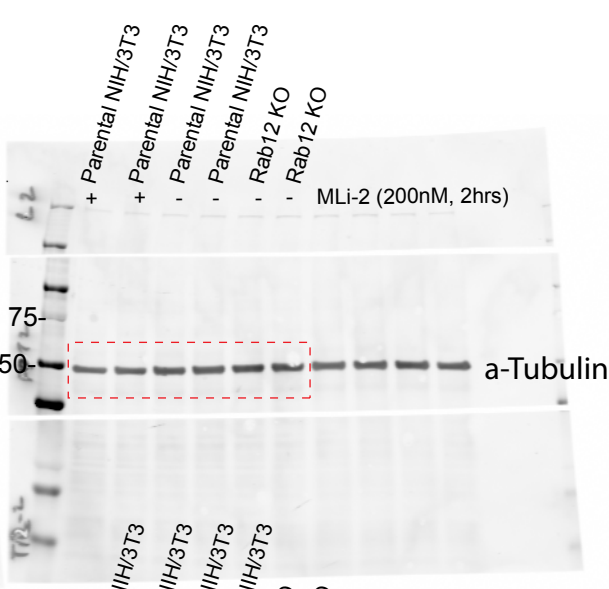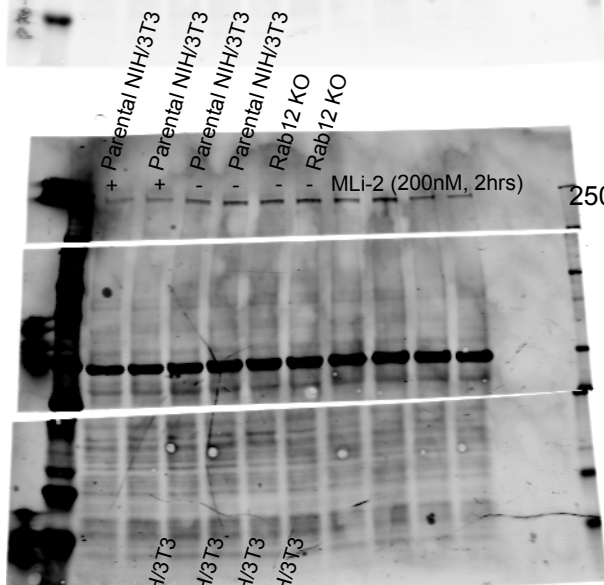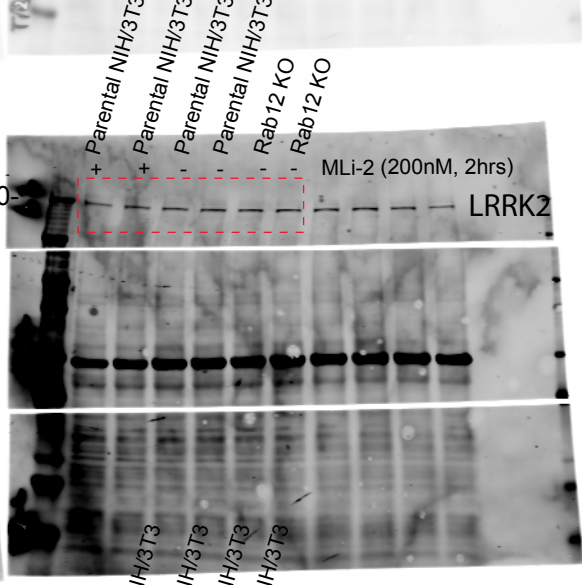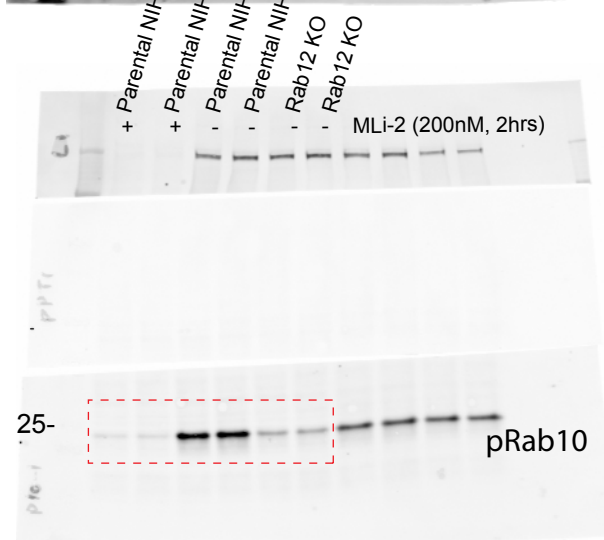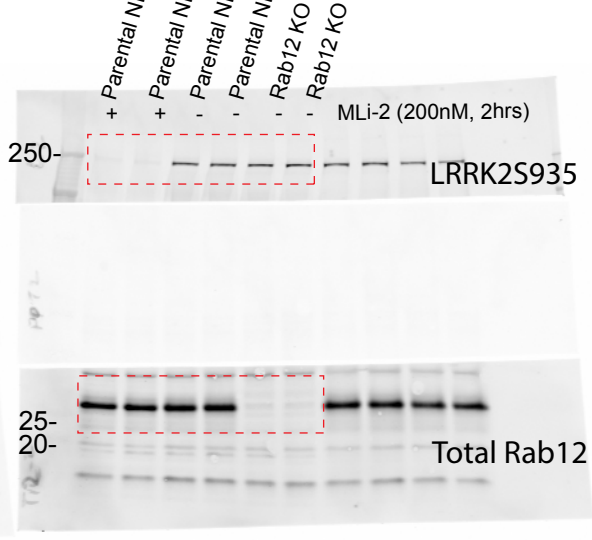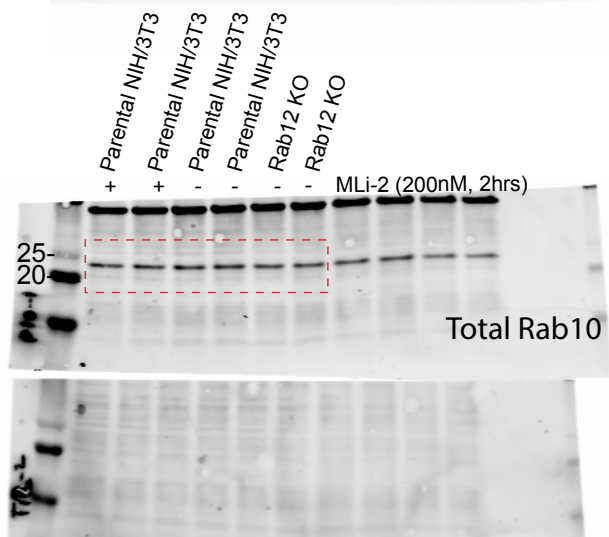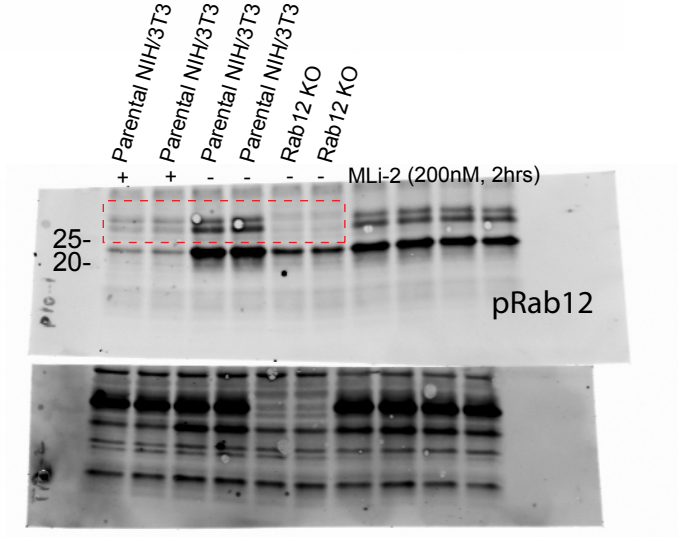

Supplement: Figure 2—source data 1. [file elife-87098-fig2-data1.zip › Figure 2A-source data 1/Supporting material for figure 2A_B_annotated blots.pdf]

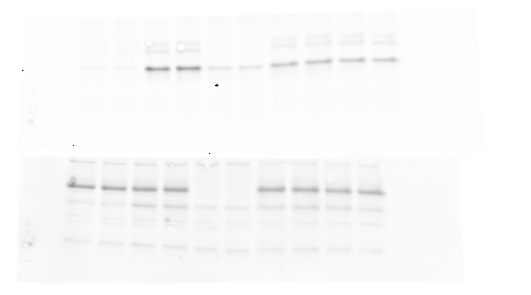

Supplement: Figure 2—source data 1. [file elife-87098-fig2-data1.zip › Figure 2A-source data 1/800_3.tif]

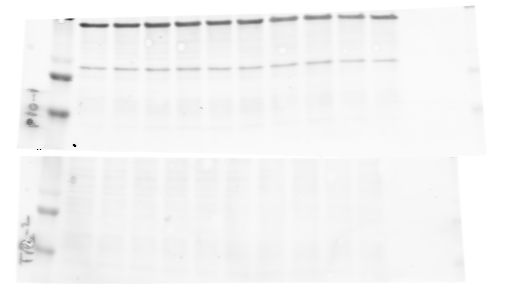

Supplement: Figure 2—source data 1. [file elife-87098-fig2-data1.zip › Figure 2A-source data 1/700_3.tif]

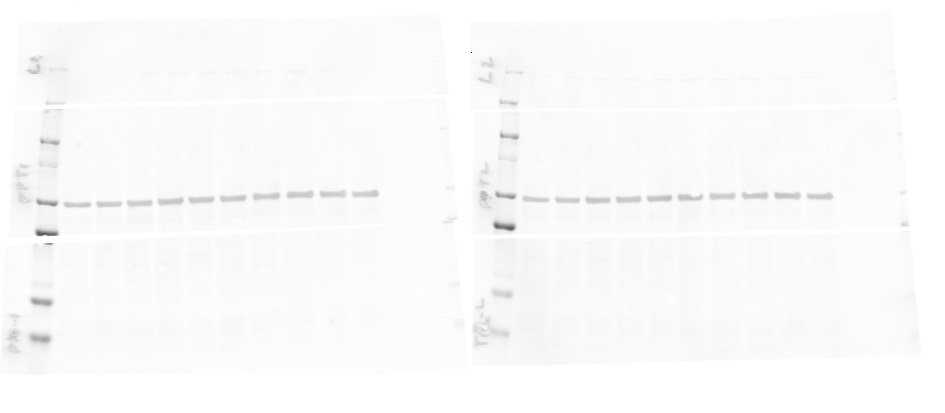

Supplement: Figure 2—source data 1. [file elife-87098-fig2-data1.zip › Figure 2A-source data 1/700_1.tif]

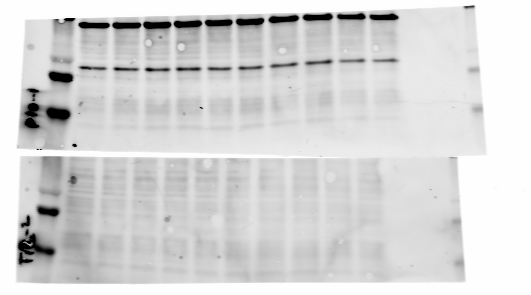

Supplement: Figure 2—source data 1. [file elife-87098-fig2-data1.zip › Figure 2A-source data 1/700_2.tif.tif]

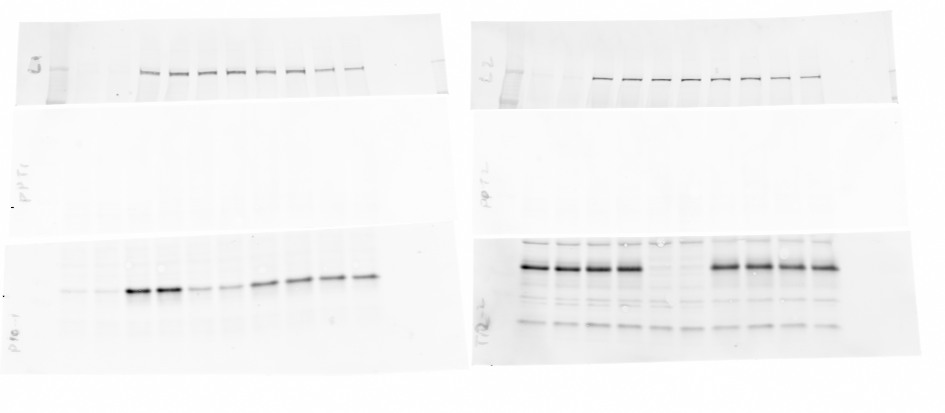

Supplement: Figure 2—source data 1. [file elife-87098-fig2-data1.zip › Figure 2A-source data 1/800.tif]

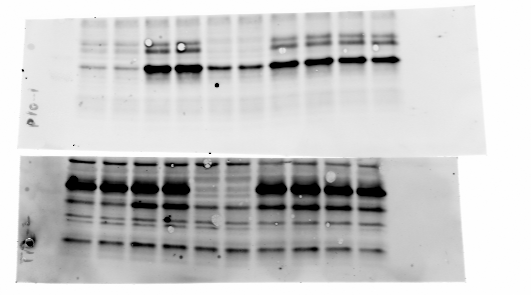

Supplement: Figure 2—source data 1. [file elife-87098-fig2-data1.zip › Figure 2A-source data 1/800_2.tif.tif]

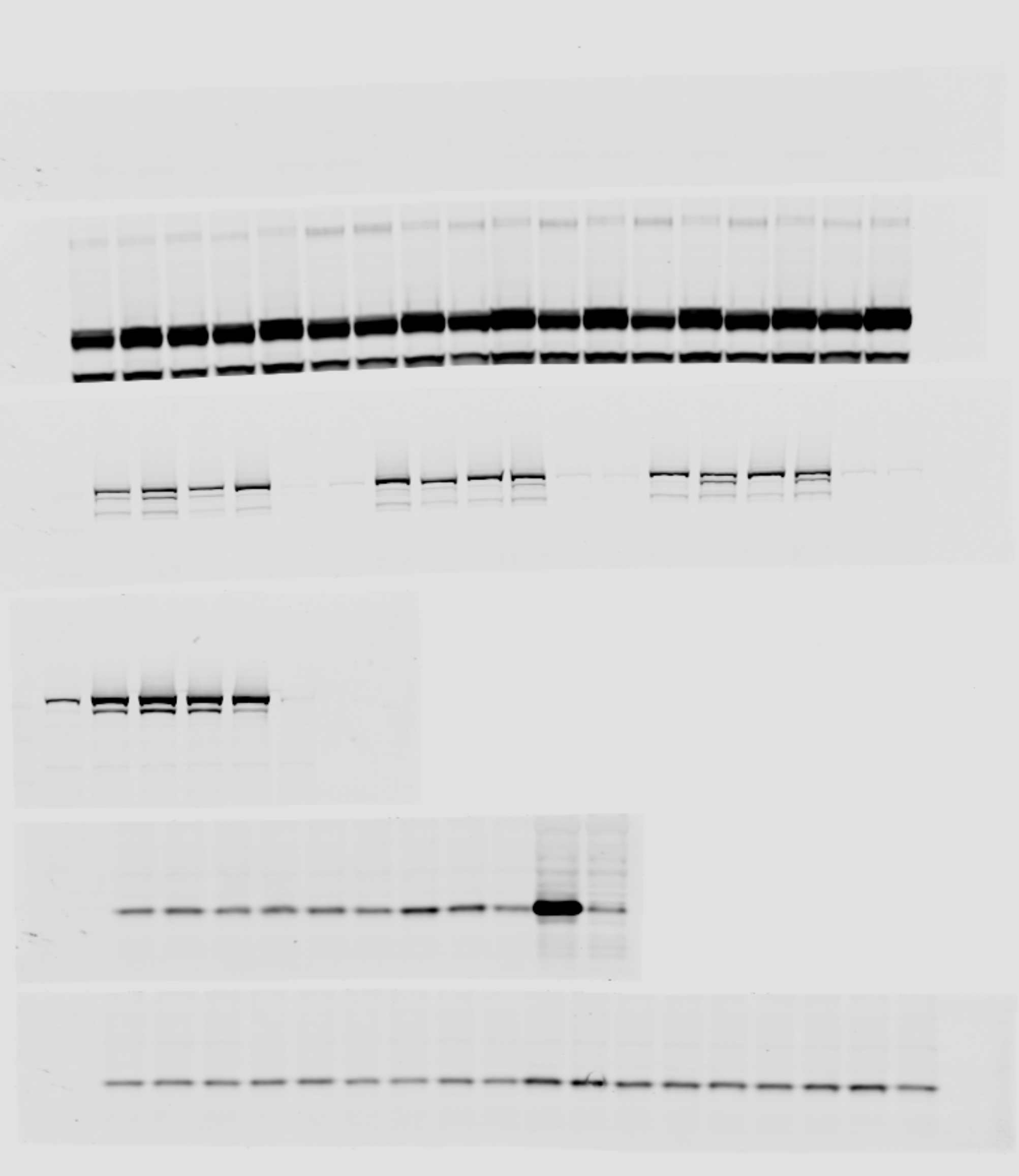

Supplement: Figure 2—figure supplement 1—source data 1. [file elife-87098-fig2-figsupp1-data1.zip › Figure 2-figure supplement 1-source data 1/raw images/Fig2_Suppl1_31-08-22_800.tif]

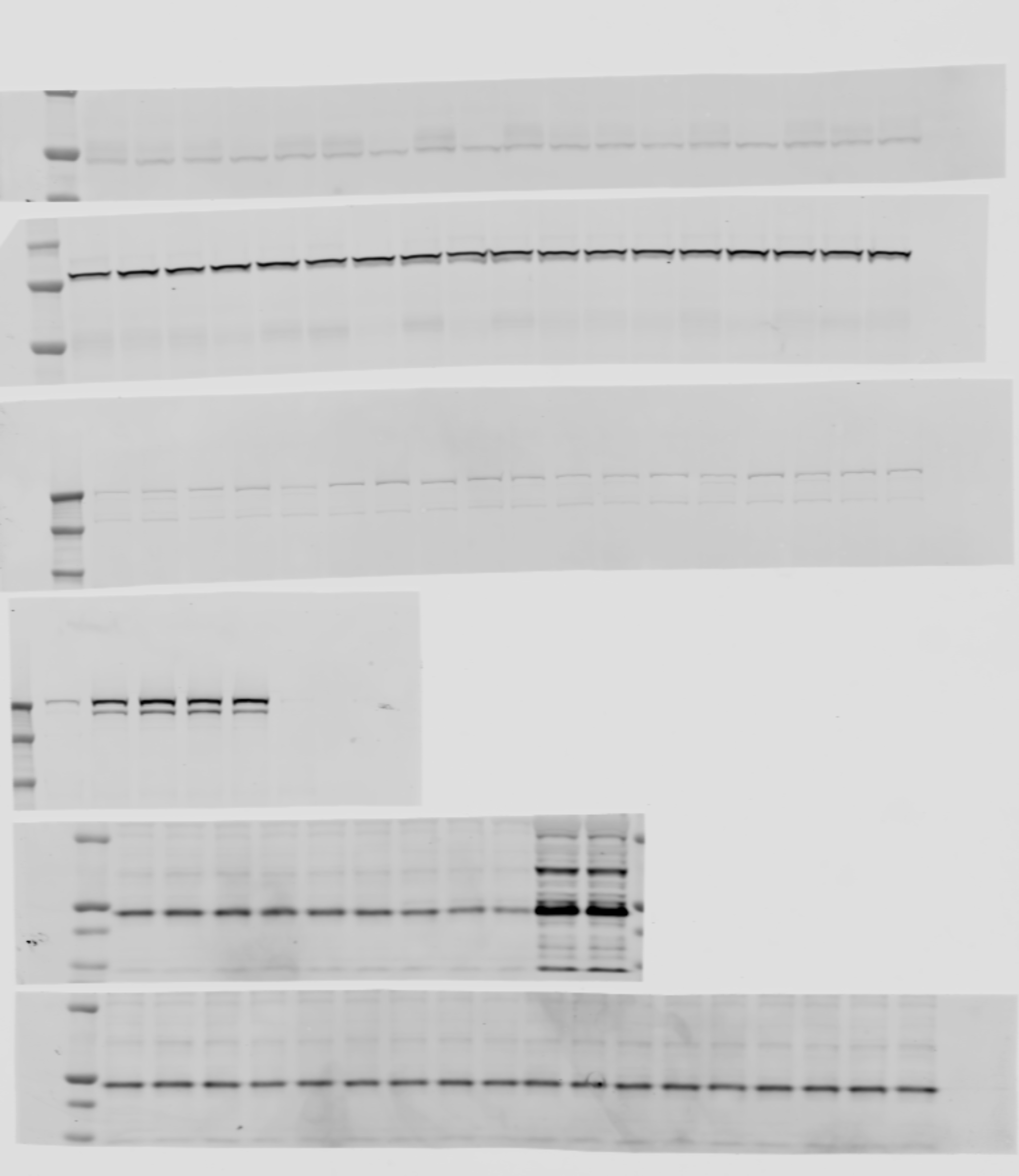

Supplement: Figure 2—figure supplement 1—source data 1. [file elife-87098-fig2-figsupp1-data1.zip › Figure 2-figure supplement 1-source data 1/raw images/Fig2_Suppl1_31-08-22_700.tif]

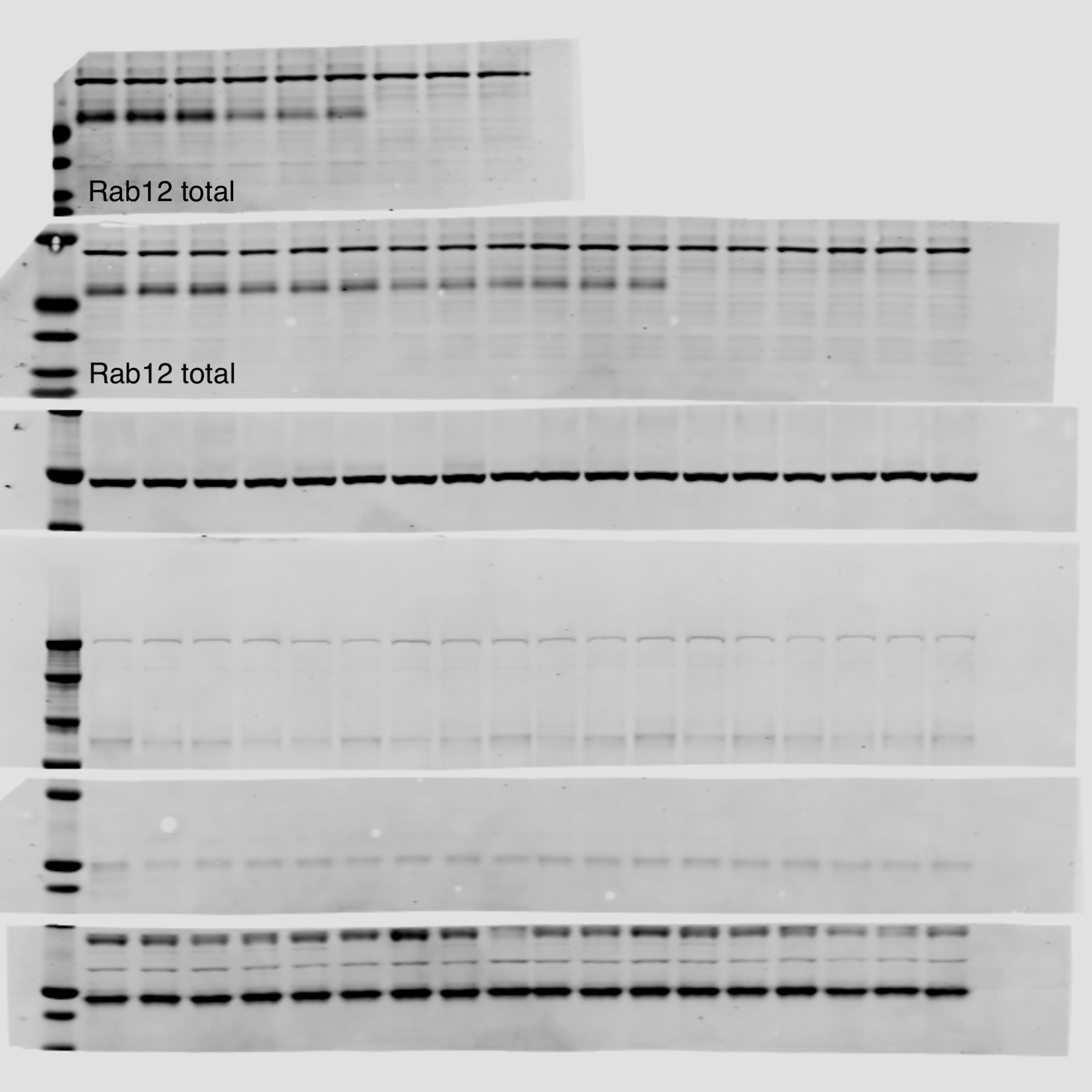

Supplement: Figure 2—figure supplement 1—source data 1. [file elife-87098-fig2-figsupp1-data1.zip › Figure 2-figure supplement 1-source data 1/annotated/Figure 2 Figure Suppl 1 MEF_700_re-scanned.tif]

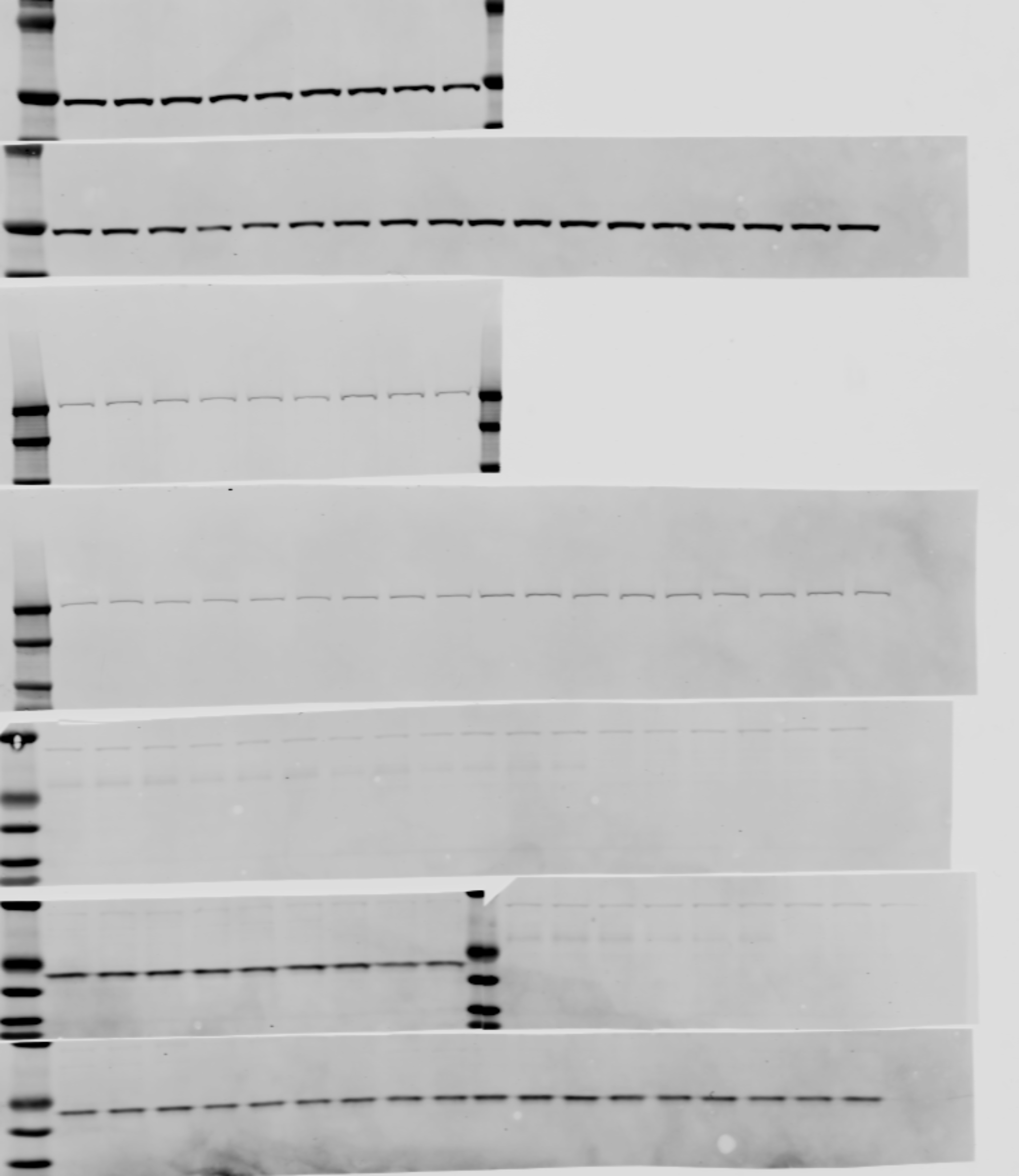

Supplement: Figure 2—figure supplement 1—source data 1. [file elife-87098-fig2-figsupp1-data1.zip › Figure 2-figure supplement 1-source data 1/raw images/Fig2_Suppl1_10-08-22_700.tif]

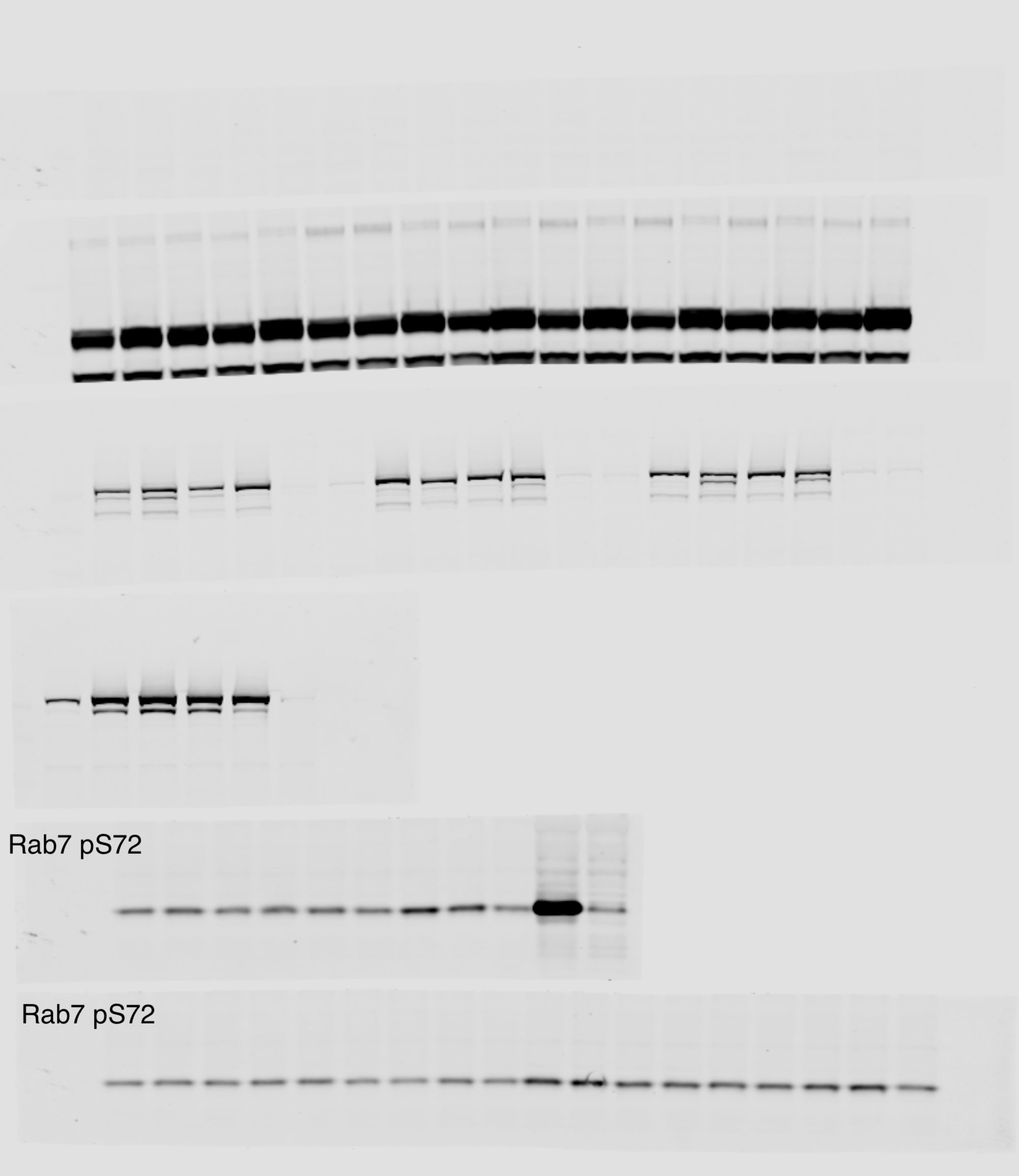

Supplement: Figure 2—figure supplement 1—source data 1. [file elife-87098-fig2-figsupp1-data1.zip › Figure 2-figure supplement 1-source data 1/annotated/Figure 2 Figure Suppl 1 MEF_800-2.tif]

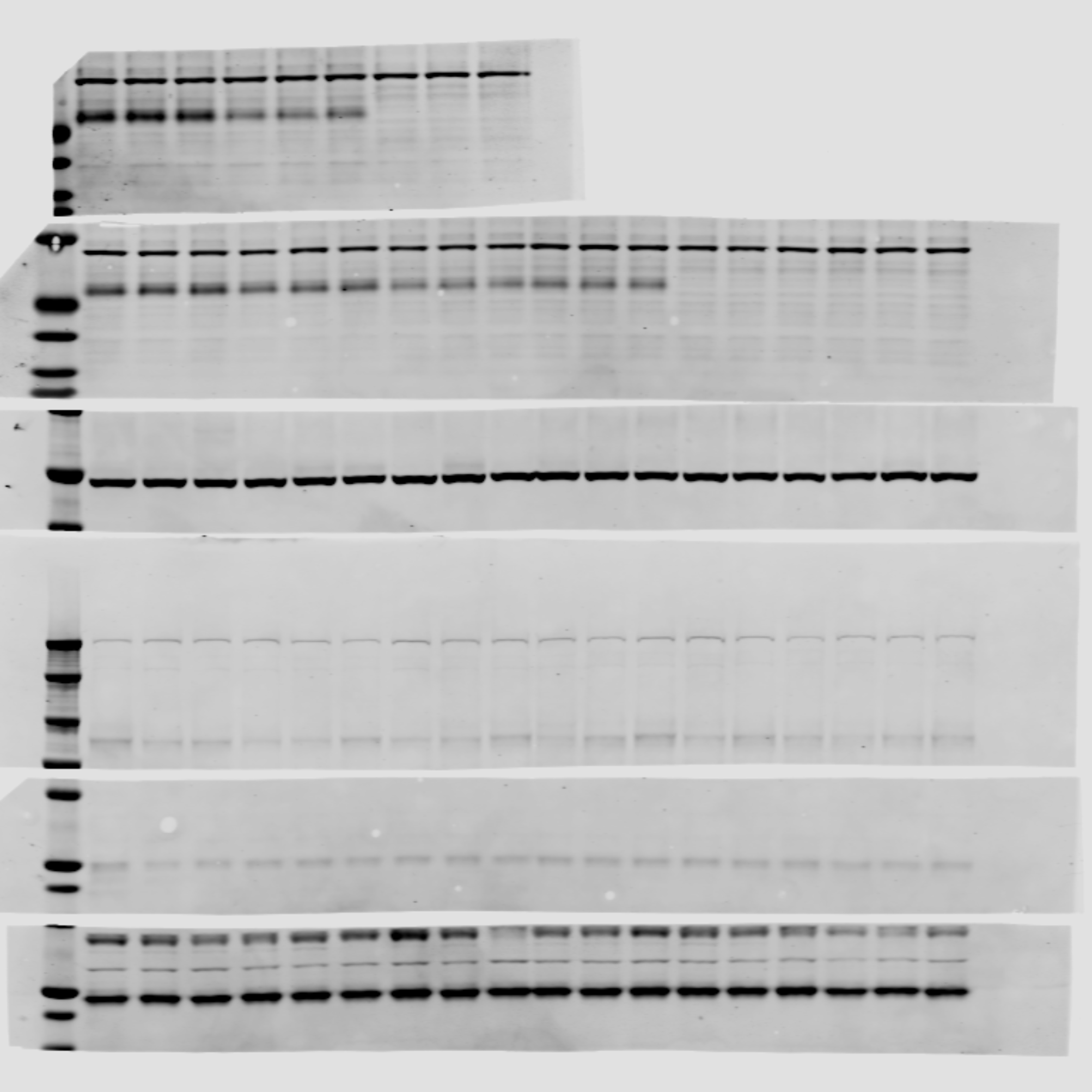

Supplement: Figure 2—figure supplement 1—source data 1. [file elife-87098-fig2-figsupp1-data1.zip › Figure 2-figure supplement 1-source data 1/raw images/Fig2_Suppl1_10-08-22_700_re-scanned.tif]

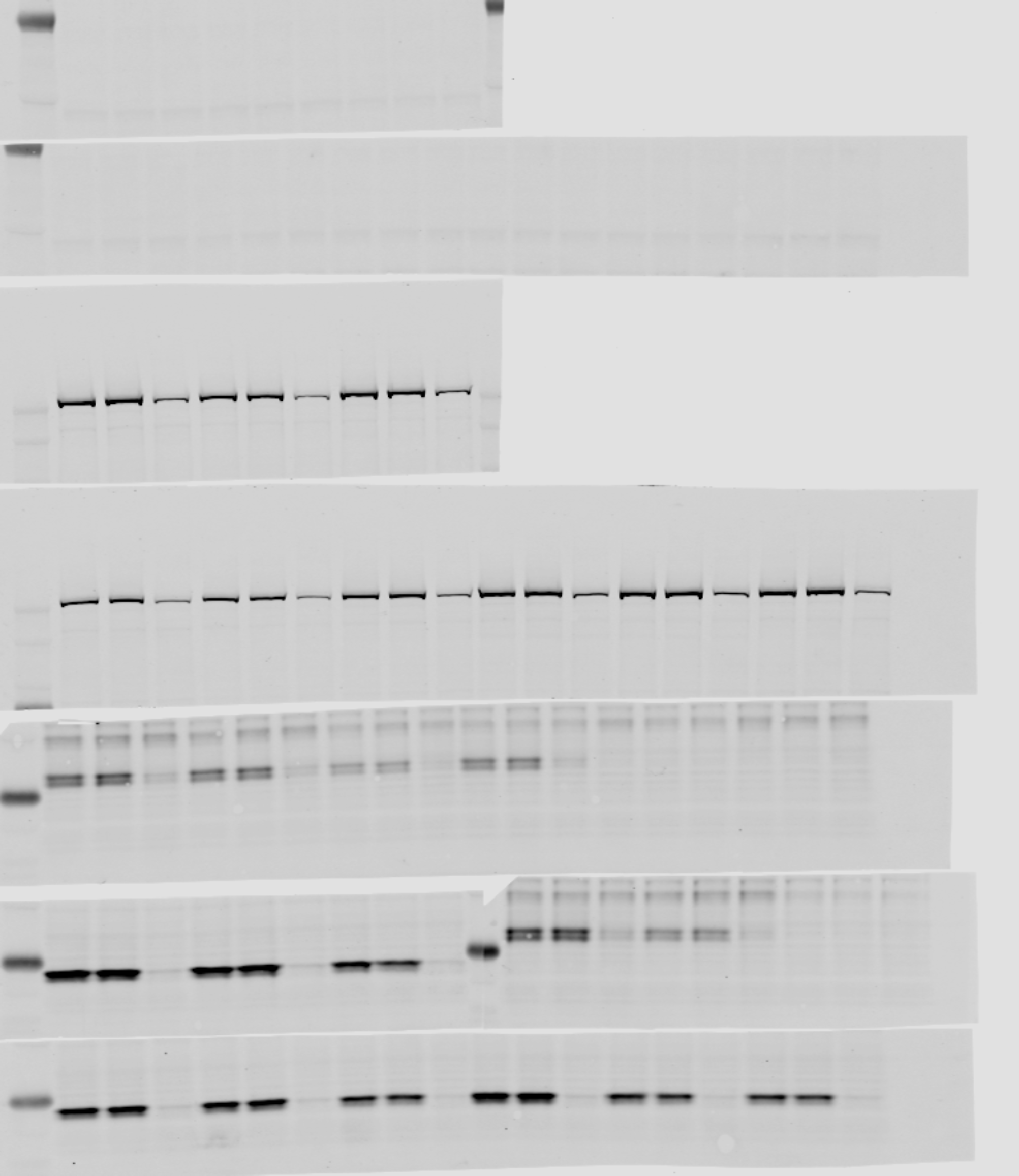

Supplement: Figure 2—figure supplement 1—source data 1. [file elife-87098-fig2-figsupp1-data1.zip › Figure 2-figure supplement 1-source data 1/raw images/Fig2_Suppl1_10-08-22_800-high.tif]

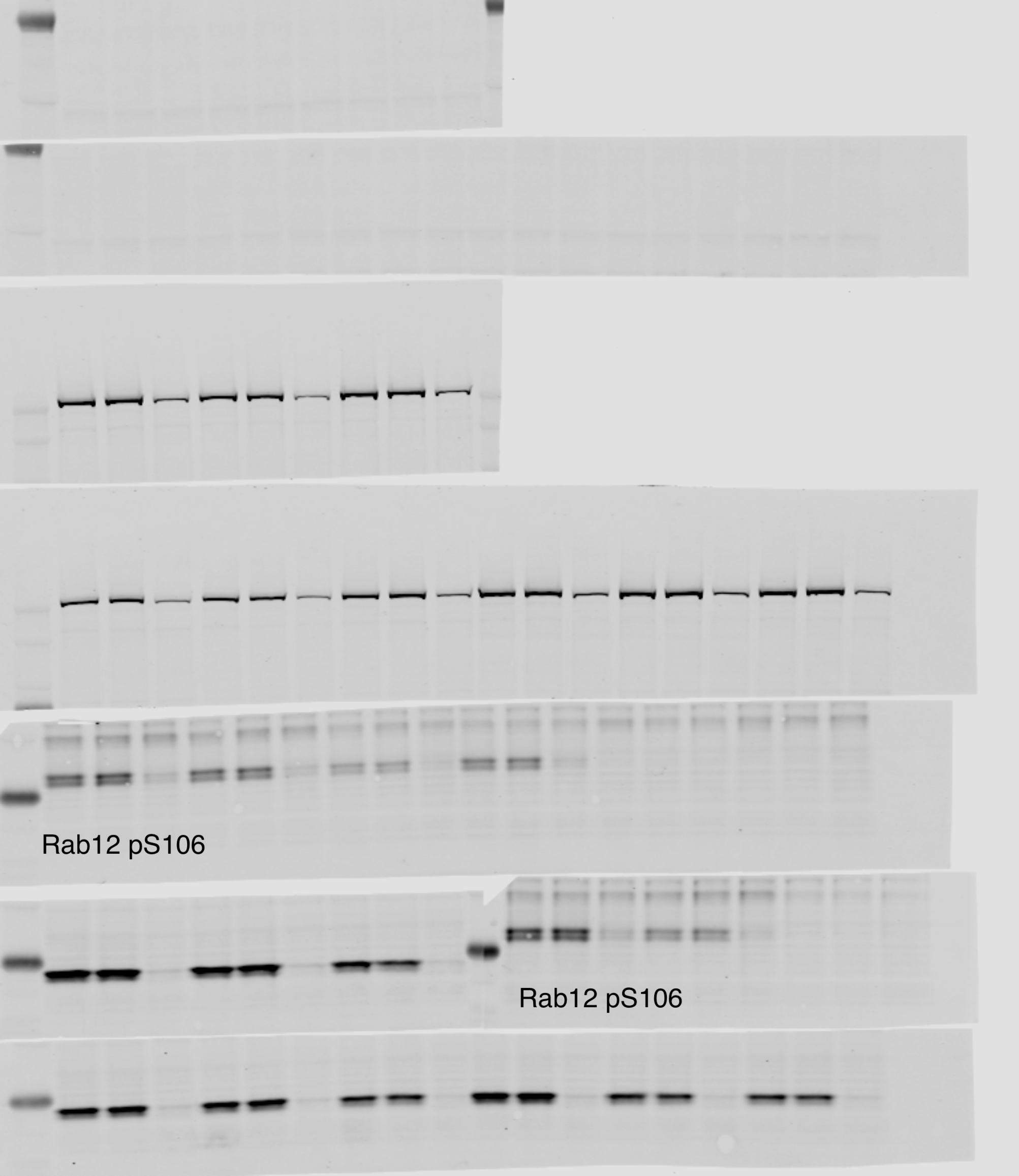

Supplement: Figure 2—figure supplement 1—source data 1. [file elife-87098-fig2-figsupp1-data1.zip › Figure 2-figure supplement 1-source data 1/annotated/Figure 2 Figure Suppl 1 MEF_800-high.tif]

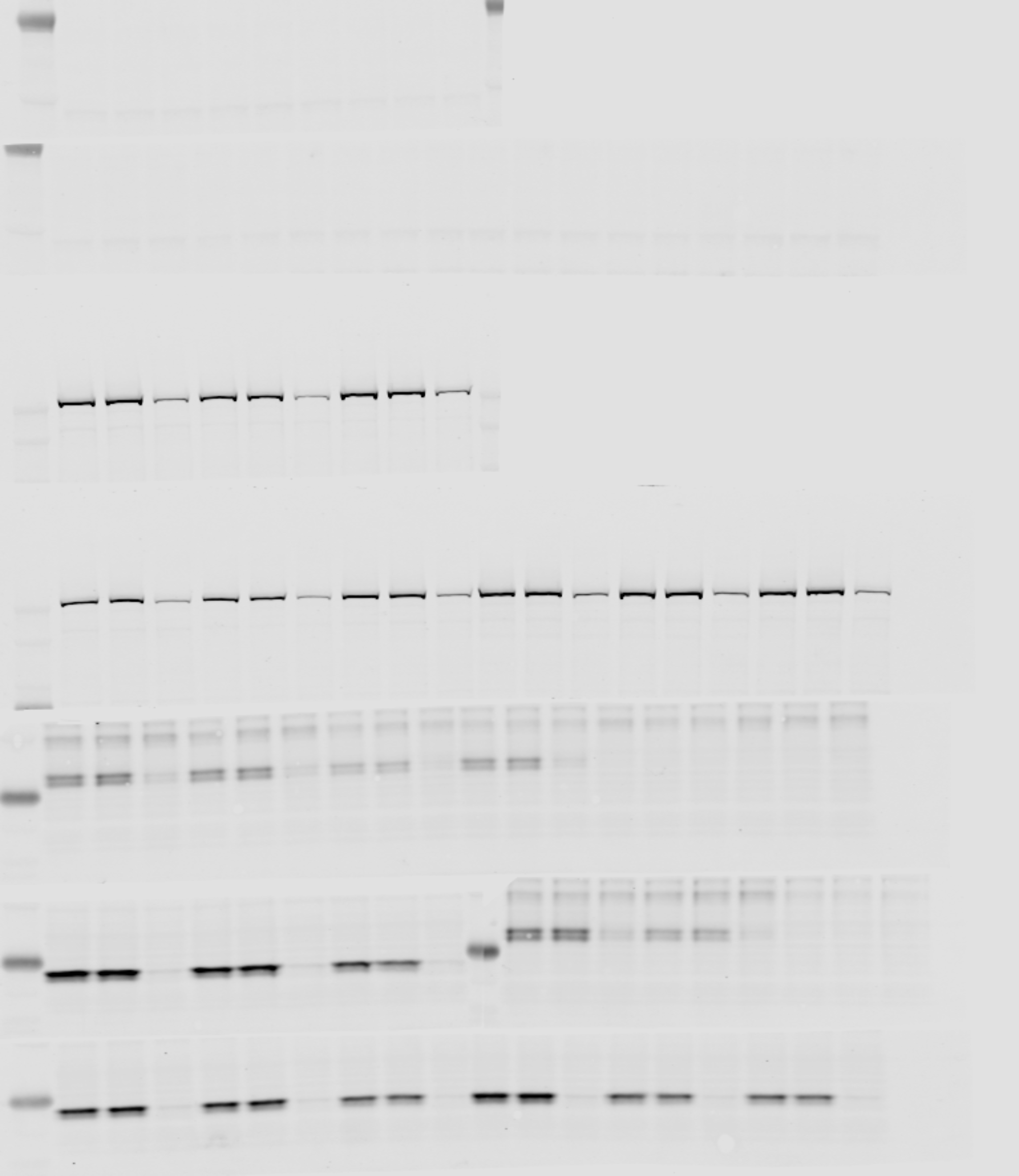

Supplement: Figure 2—figure supplement 1—source data 1. [file elife-87098-fig2-figsupp1-data1.zip › Figure 2-figure supplement 1-source data 1/raw images/Fig2_Suppl1_10-08-22_800.tif]

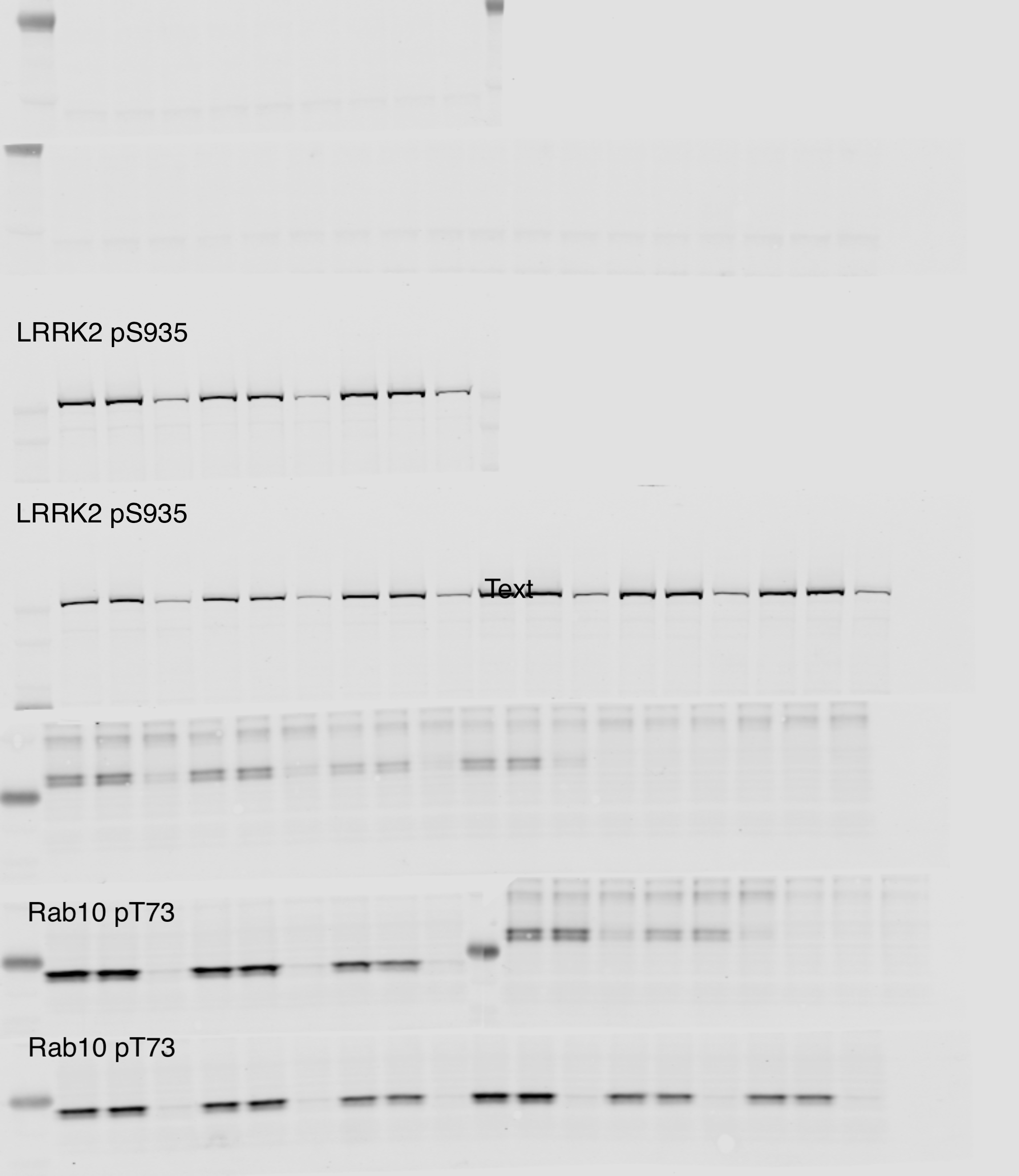

Supplement: Figure 2—figure supplement 1—source data 1. [file elife-87098-fig2-figsupp1-data1.zip › Figure 2-figure supplement 1-source data 1/annotated/Figure 2 Figure Suppl 1 MEF_800.tif]

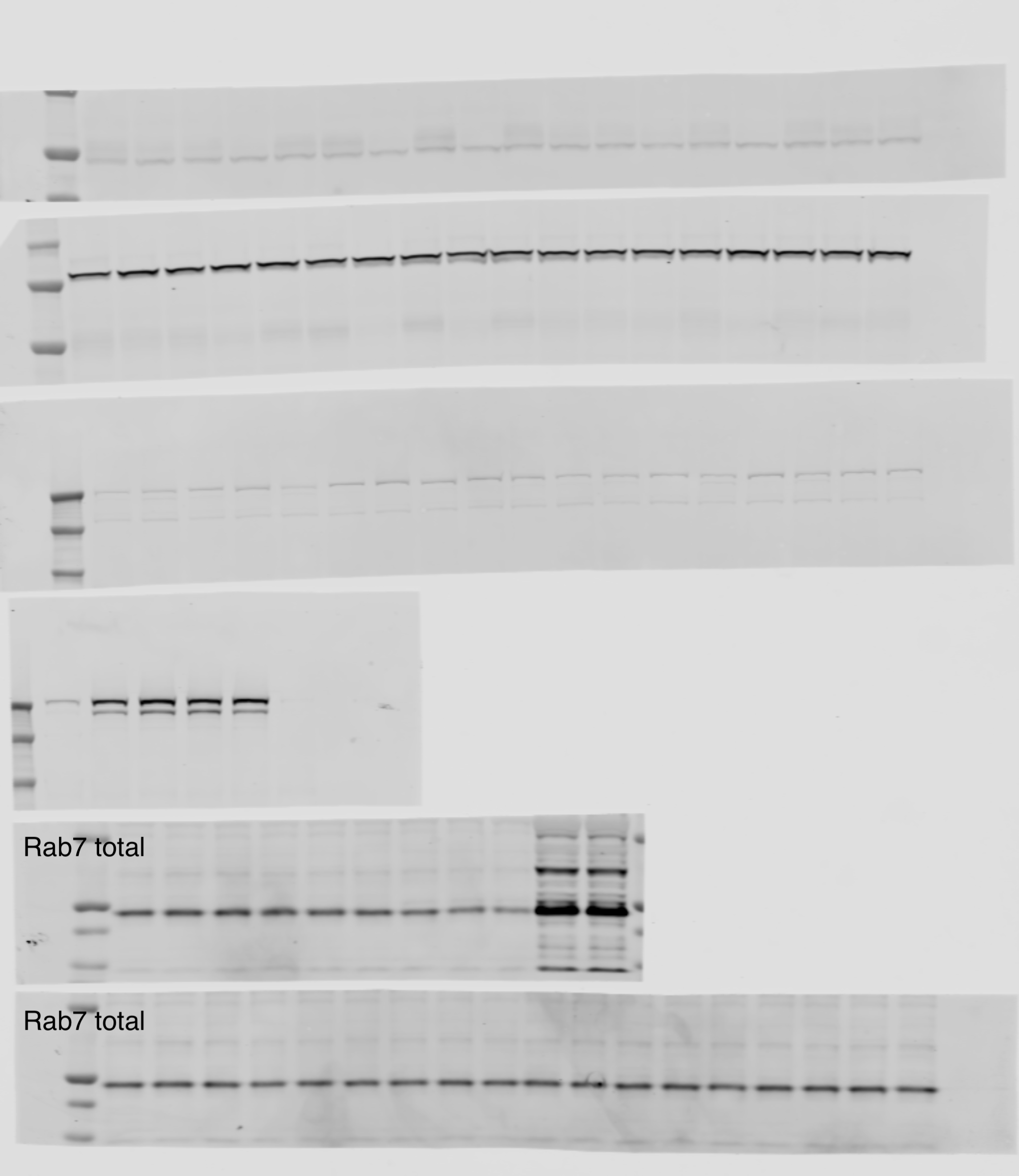

Supplement: Figure 2—figure supplement 1—source data 1. [file elife-87098-fig2-figsupp1-data1.zip › Figure 2-figure supplement 1-source data 1/annotated/Figure 2 Figure Suppl 1 MEF_700-2.tif]

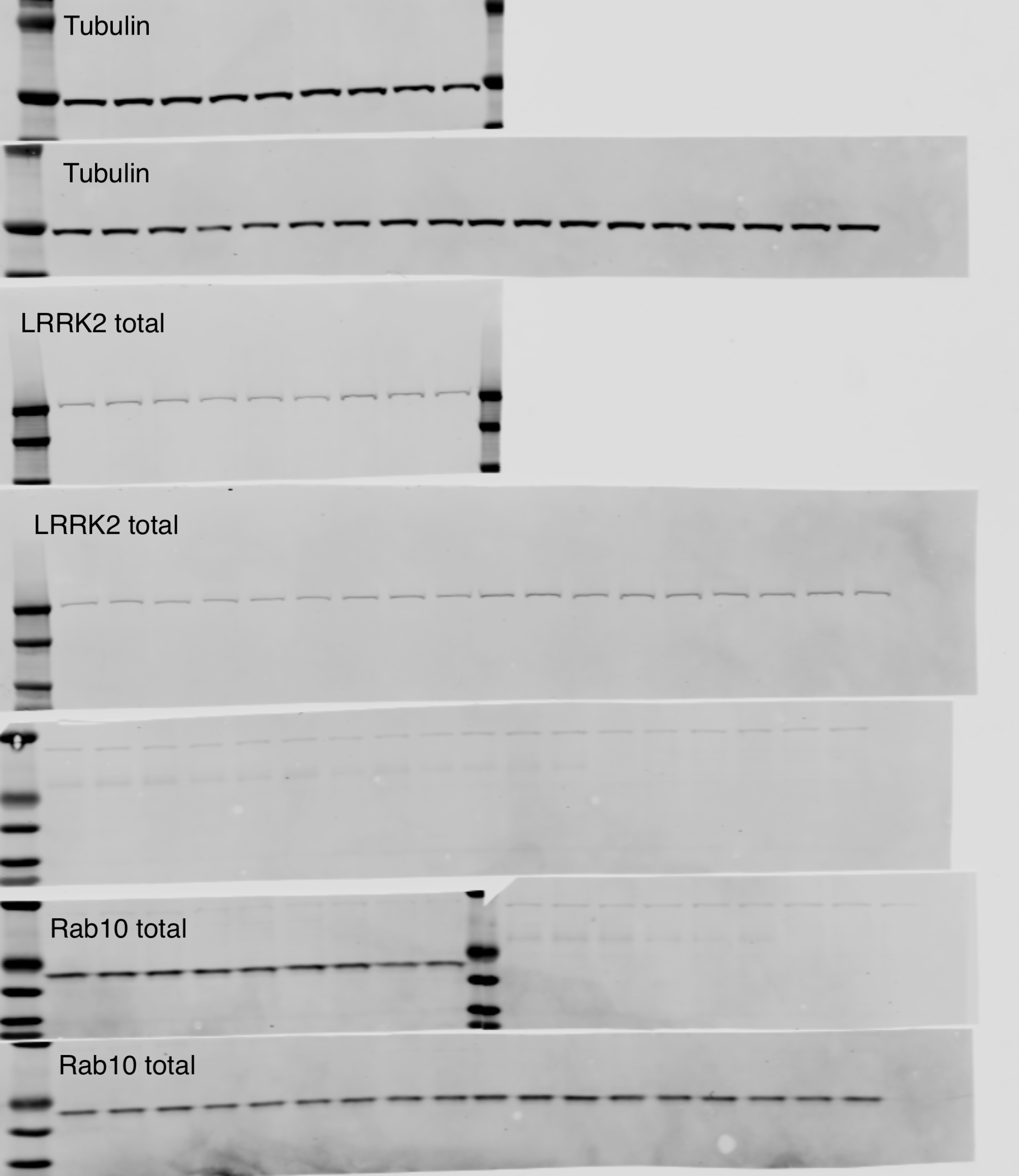

Supplement: Figure 2—figure supplement 1—source data 1. [file elife-87098-fig2-figsupp1-data1.zip › Figure 2-figure supplement 1-source data 1/annotated/Figure 2 Figure Suppl 1 MEF_700.tif]

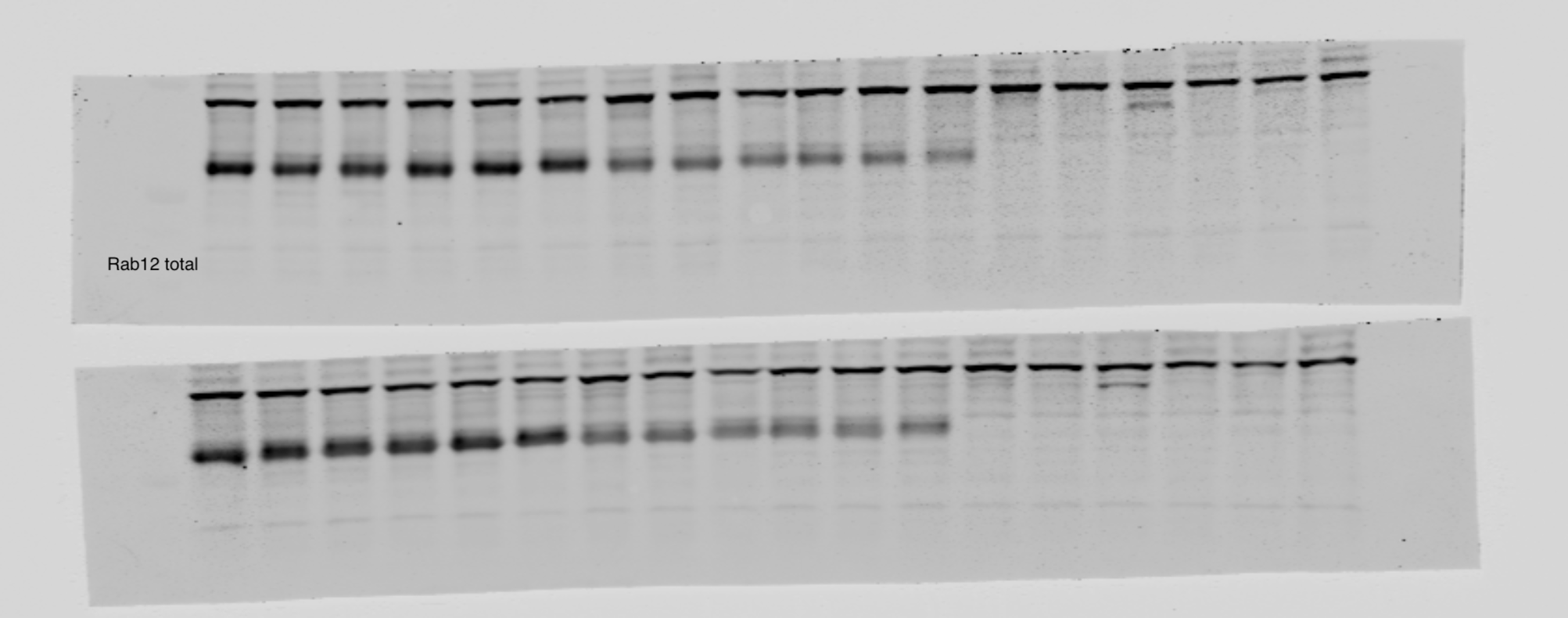

Supplement: Figure 2—figure supplement 2—source data 1. [file elife-87098-fig2-figsupp2-data1.zip › Figure 2-figure supplement 2-source data 1/annotated/Figure 2 Figure Suppl 2 Large intestine_800-2.tif]

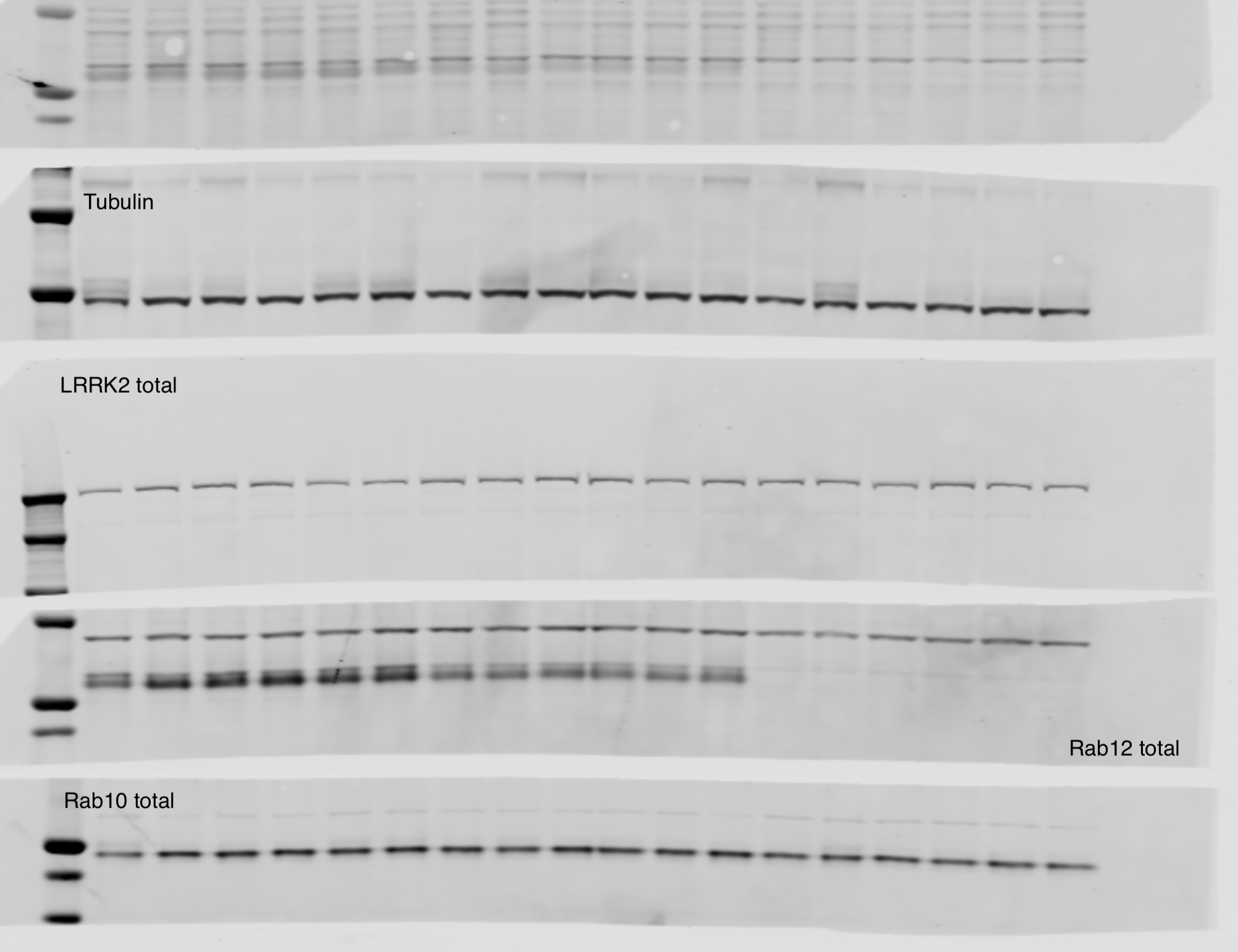

Supplement: Figure 2—figure supplement 2—source data 1. [file elife-87098-fig2-figsupp2-data1.zip › Figure 2-figure supplement 2-source data 1/annotated/Figure 2 Figure Suppl 2 Lung_700.tif]

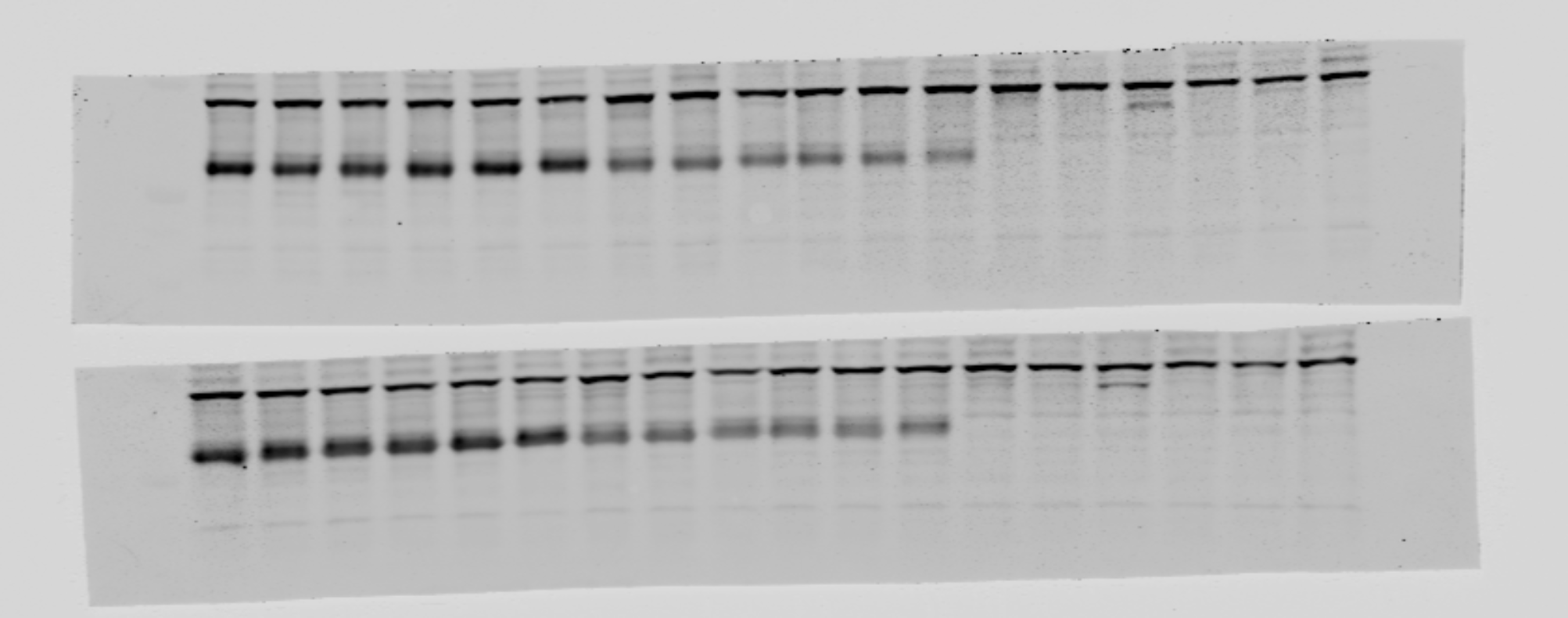

Supplement: Figure 2—figure supplement 2—source data 1. [file elife-87098-fig2-figsupp2-data1.zip › Figure 2-figure supplement 2-source data 1/raw images/Fig2_Suppl2_LargeInt_10-02-23_800.tif]

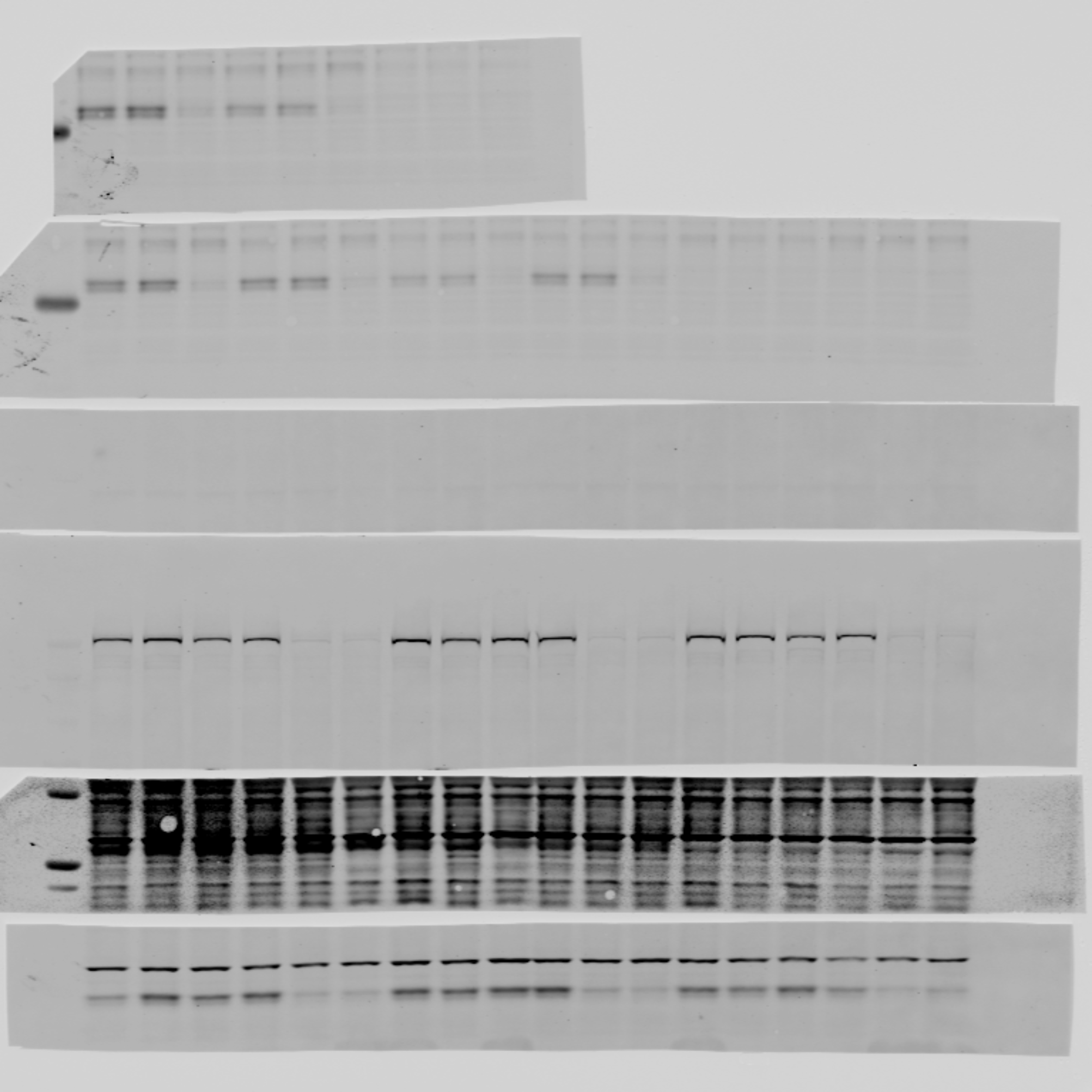

Supplement: Figure 2—figure supplement 2—source data 1. [file elife-87098-fig2-figsupp2-data1.zip › Figure 2-figure supplement 2-source data 1/raw images/Fig2_Suppl2_LargeInt_11-08-22_800.tif]

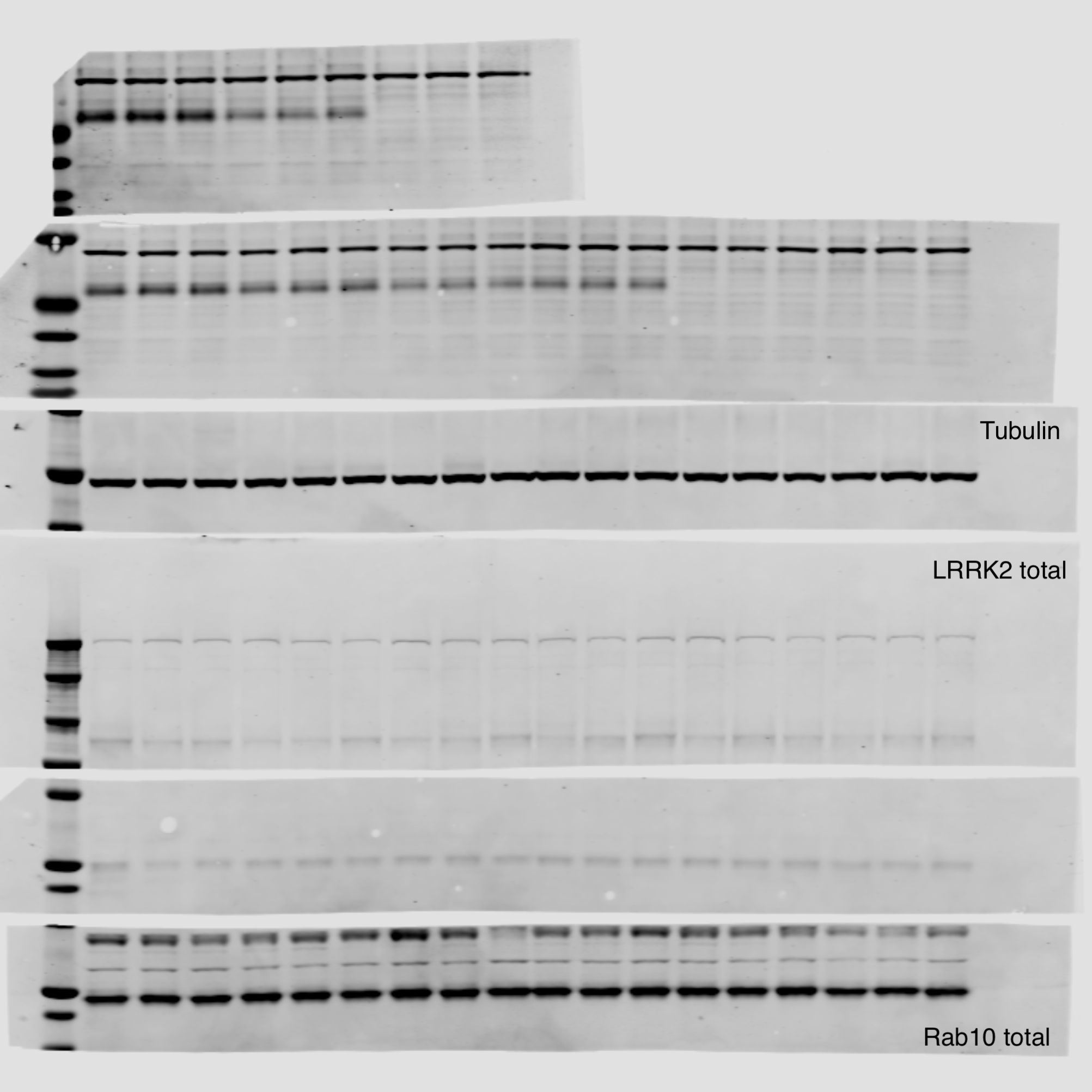

Supplement: Figure 2—figure supplement 2—source data 1. [file elife-87098-fig2-figsupp2-data1.zip › Figure 2-figure supplement 2-source data 1/annotated/Figure 2 Figure Suppl 2 Large intestine_700.tif]

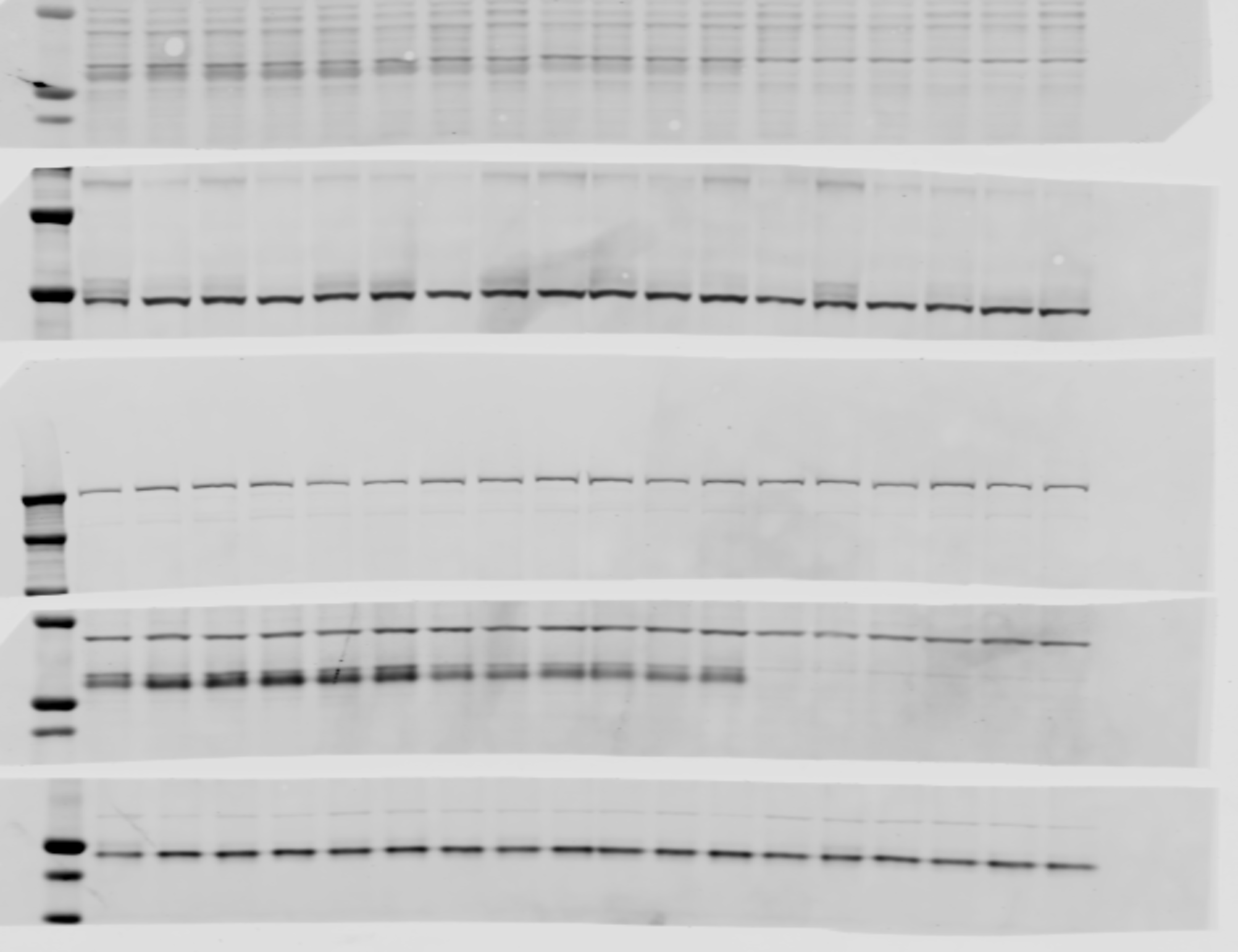

Supplement: Figure 2—figure supplement 2—source data 1. [file elife-87098-fig2-figsupp2-data1.zip › Figure 2-figure supplement 2-source data 1/raw images/Fig2_Suppl2_Lung_18-08-22_700.tif]

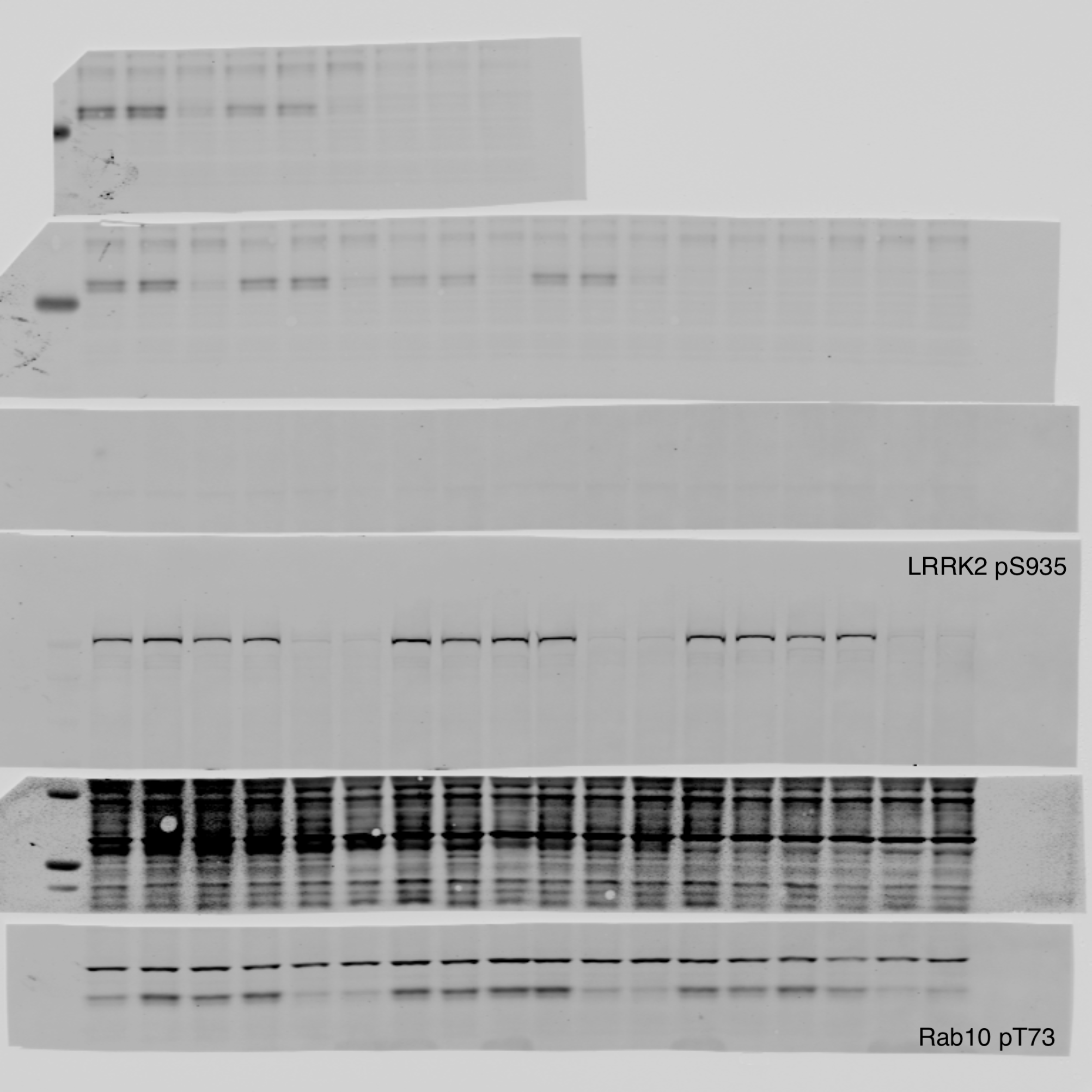

Supplement: Figure 2—figure supplement 2—source data 1. [file elife-87098-fig2-figsupp2-data1.zip › Figure 2-figure supplement 2-source data 1/annotated/Figure 2 Figure Suppl 2 Large intestine_800.tif]

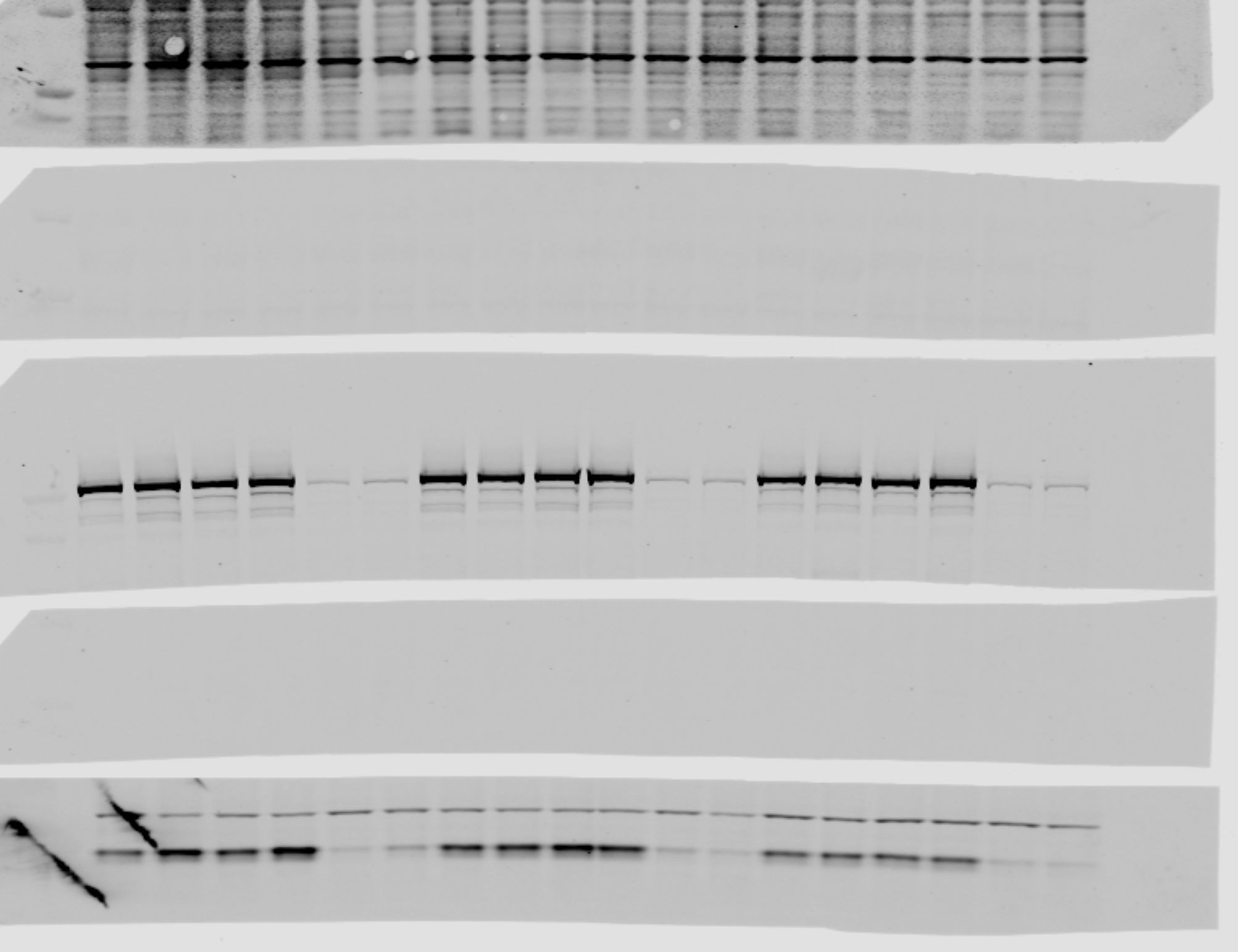

Supplement: Figure 2—figure supplement 2—source data 1. [file elife-87098-fig2-figsupp2-data1.zip › Figure 2-figure supplement 2-source data 1/raw images/Fig2_Suppl2_Lung_18-08-22_800.tif]

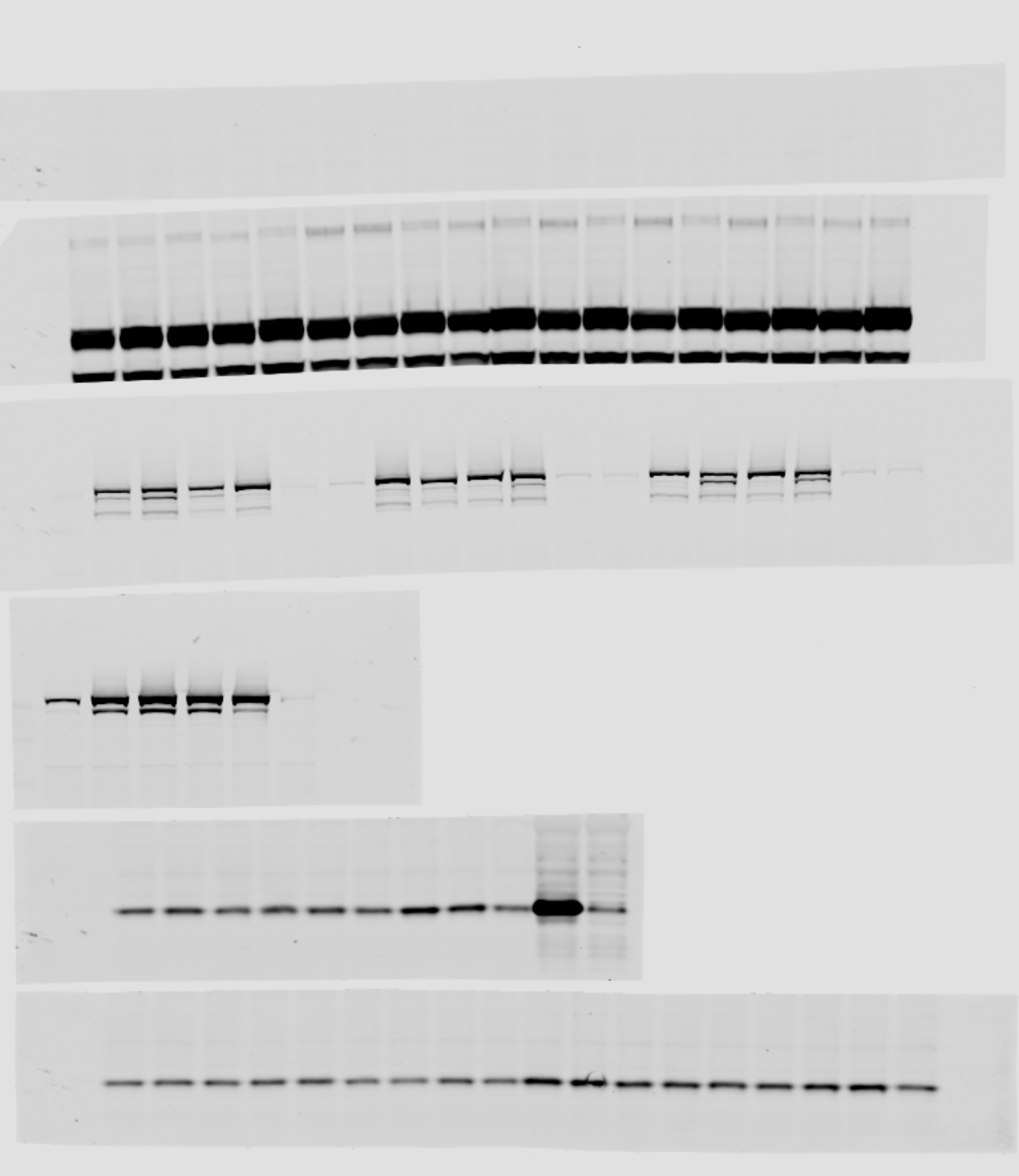

Supplement: Figure 2—figure supplement 2—source data 1. [file elife-87098-fig2-figsupp2-data1.zip › Figure 2-figure supplement 2-source data 1/raw images/Fig2_Suppl2_kidney_31-08-22_re-probed_800.tif]

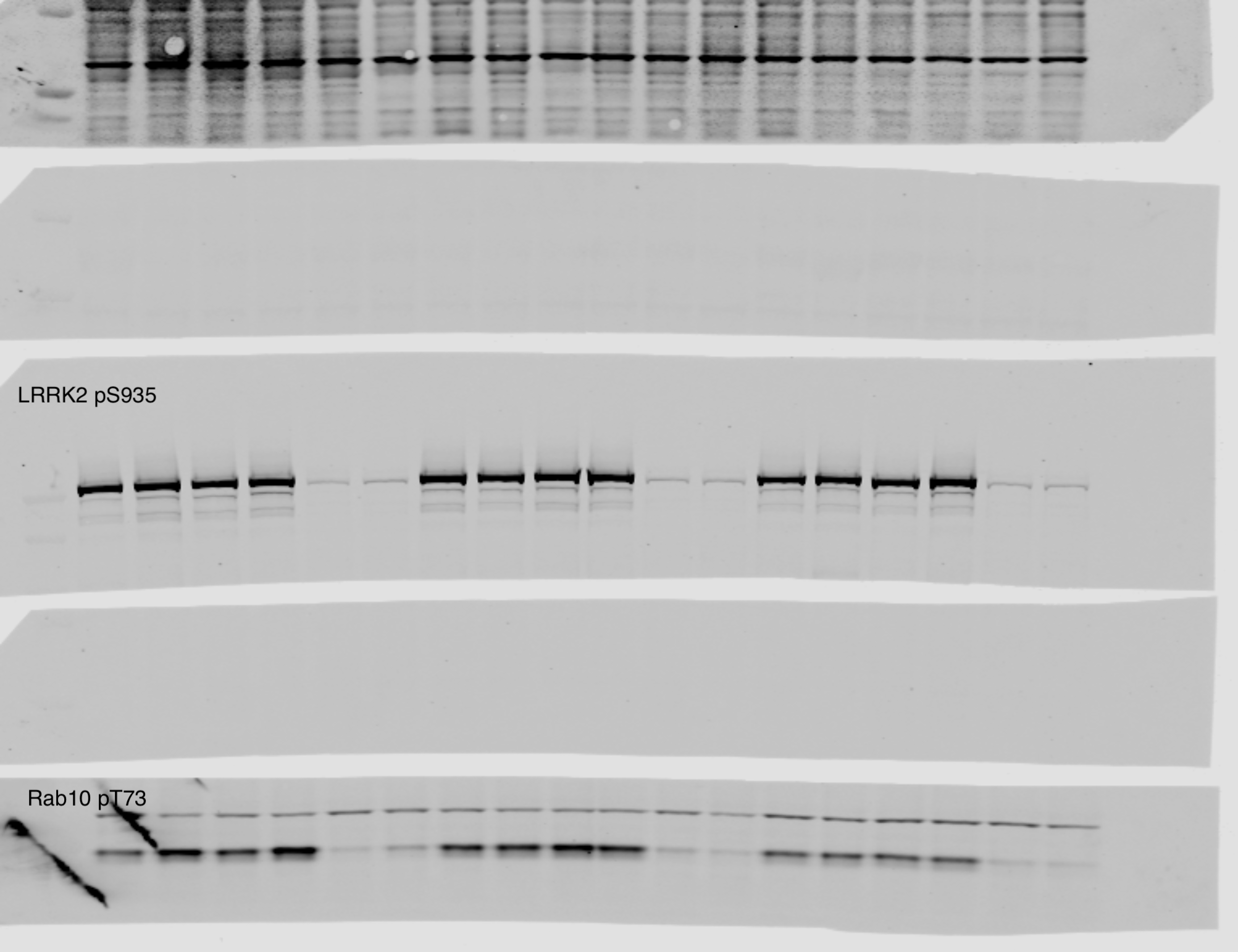

Supplement: Figure 2—figure supplement 2—source data 1. [file elife-87098-fig2-figsupp2-data1.zip › Figure 2-figure supplement 2-source data 1/annotated/Figure 2 Figure Suppl 2 Lung_800.tif]

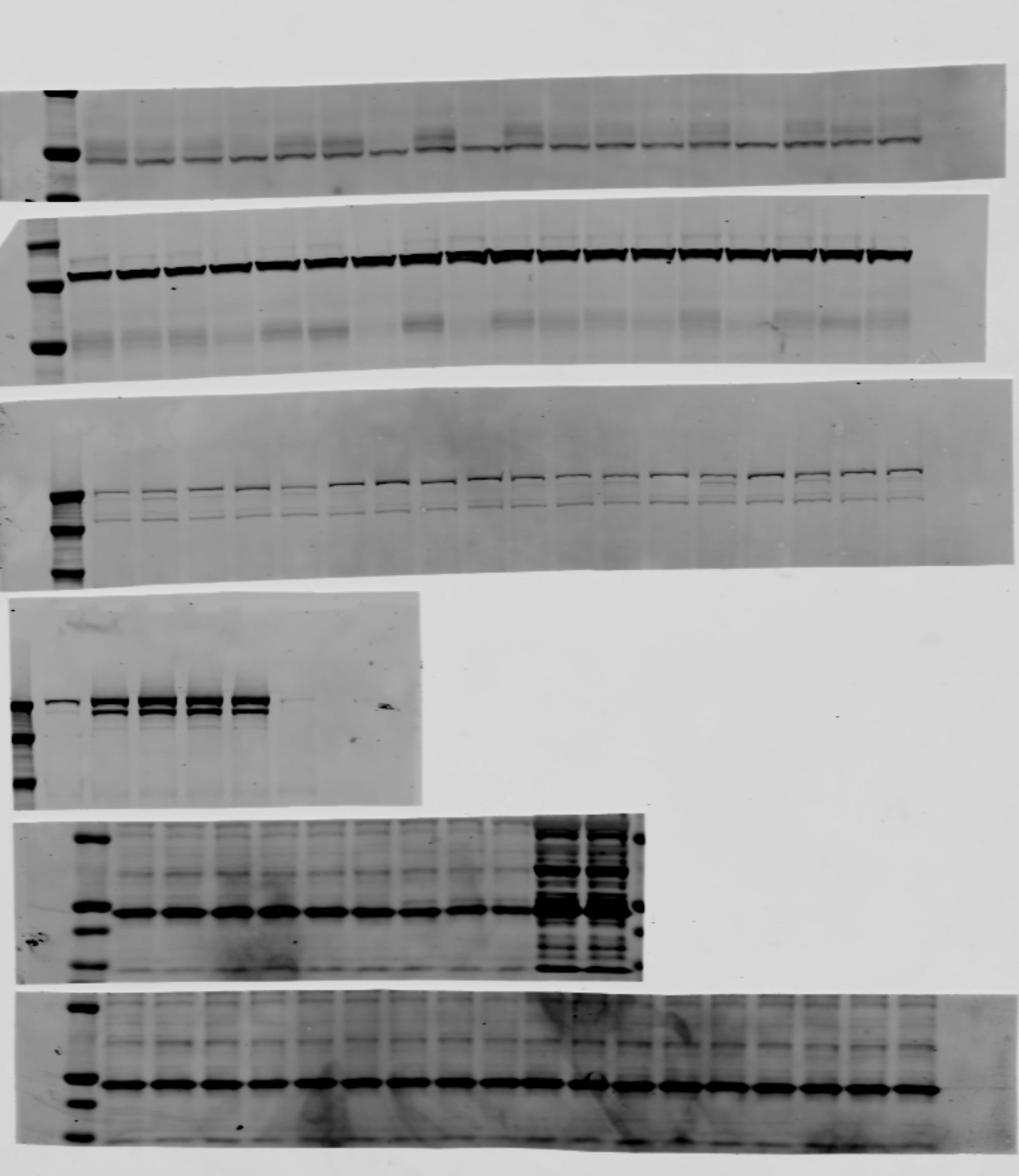

Supplement: Figure 2—figure supplement 2—source data 1. [file elife-87098-fig2-figsupp2-data1.zip › Figure 2-figure supplement 2-source data 1/raw images/Fig2_Suppl2_kidney_31-08-22_re-probed_700.tif]

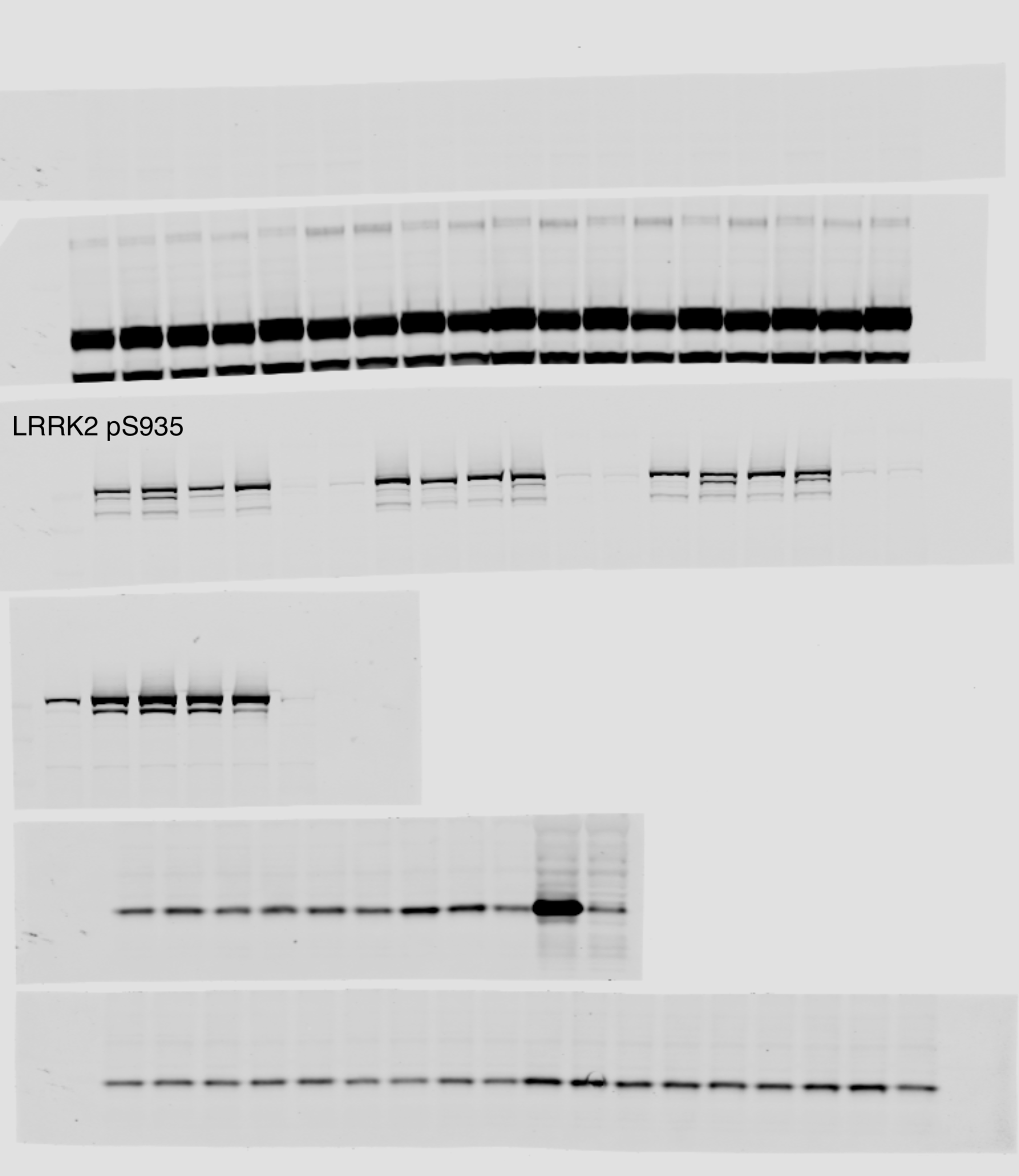

Supplement: Figure 2—figure supplement 2—source data 1. [file elife-87098-fig2-figsupp2-data1.zip › Figure 2-figure supplement 2-source data 1/annotated/Figure 2 Figure Suppl 2 Kidney_re-probed_800.tif]

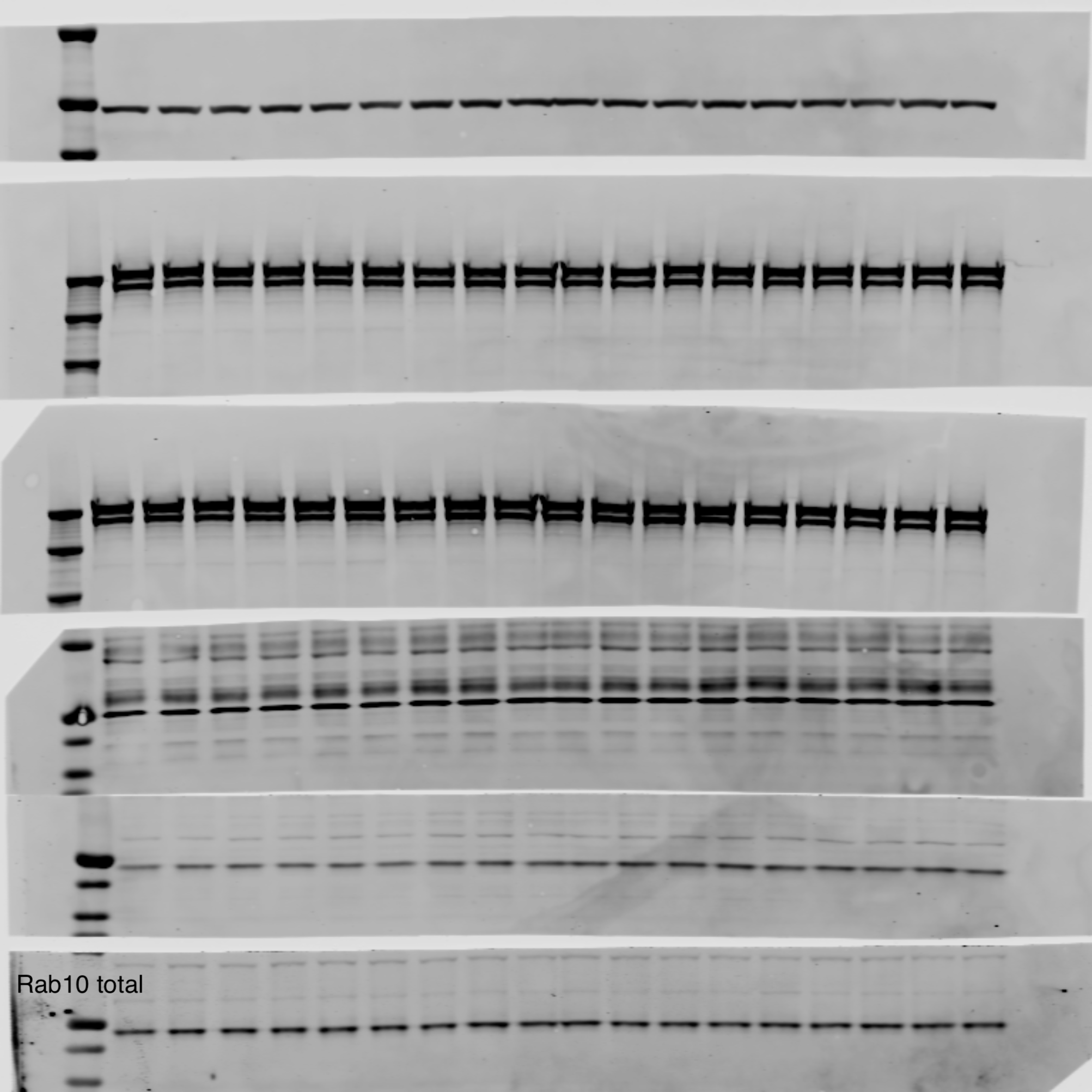

Supplement: Figure 2—figure supplement 2—source data 1. [file elife-87098-fig2-figsupp2-data1.zip › Figure 2-figure supplement 2-source data 1/annotated/Figure 2 Figure Suppl 2 Kidney_700.tif]

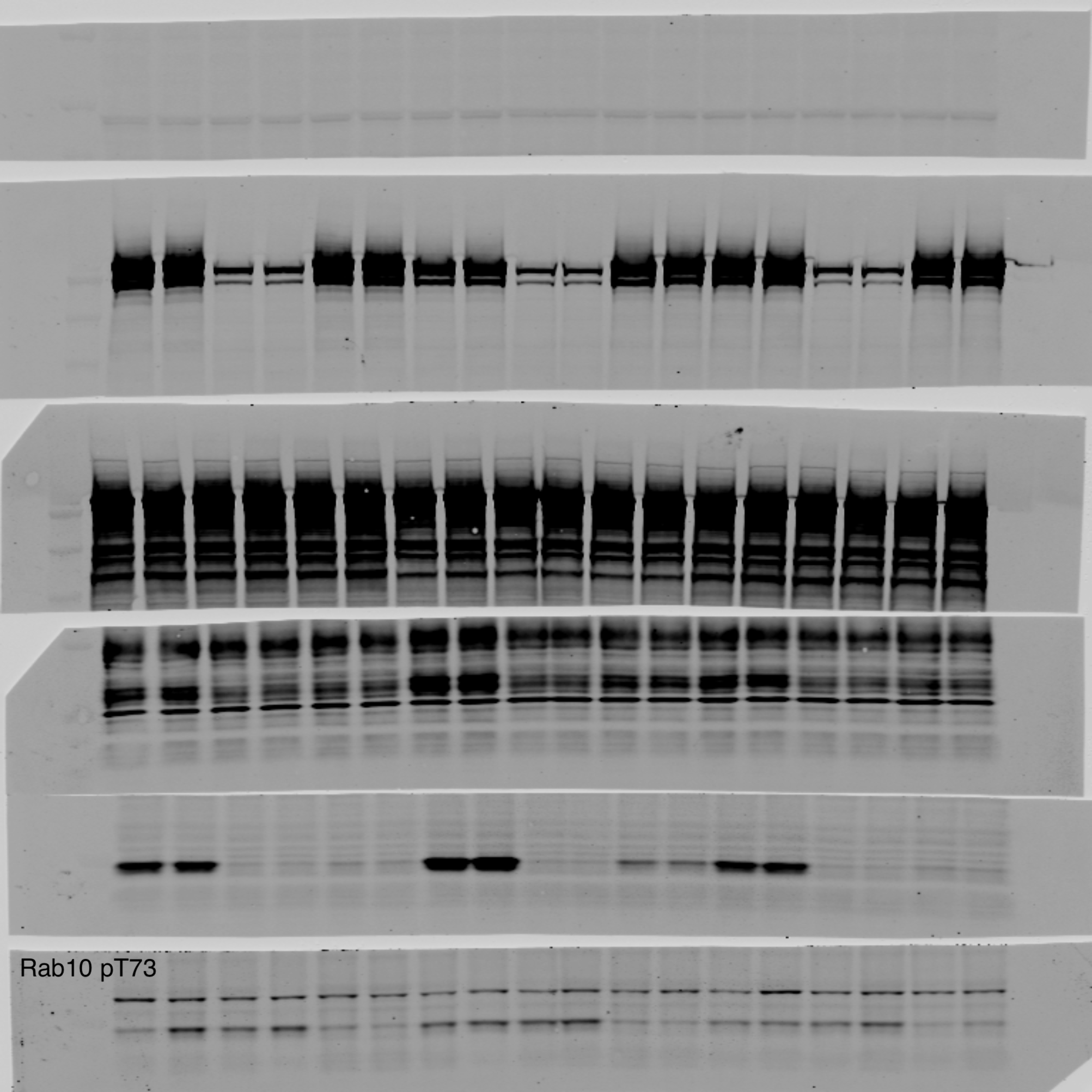

Supplement: Figure 2—figure supplement 2—source data 1. [file elife-87098-fig2-figsupp2-data1.zip › Figure 2-figure supplement 2-source data 1/annotated/Figure 2 Figure Suppl 2 Kidney_800-2.tif]

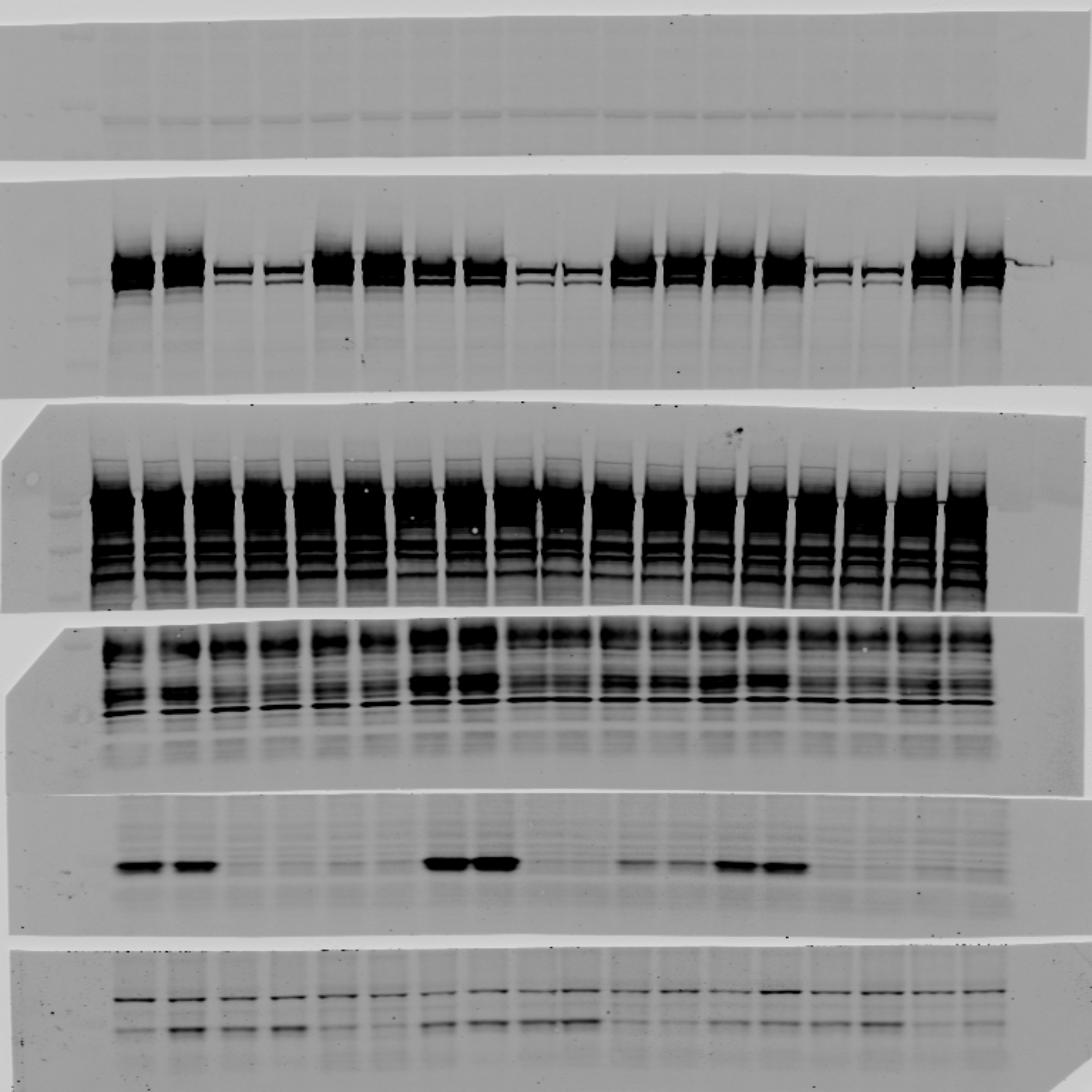

Supplement: Figure 2—figure supplement 2—source data 1. [file elife-87098-fig2-figsupp2-data1.zip › Figure 2-figure supplement 2-source data 1/raw images/Fig2_Suppl2_kidney_01-09-22_800.tif]

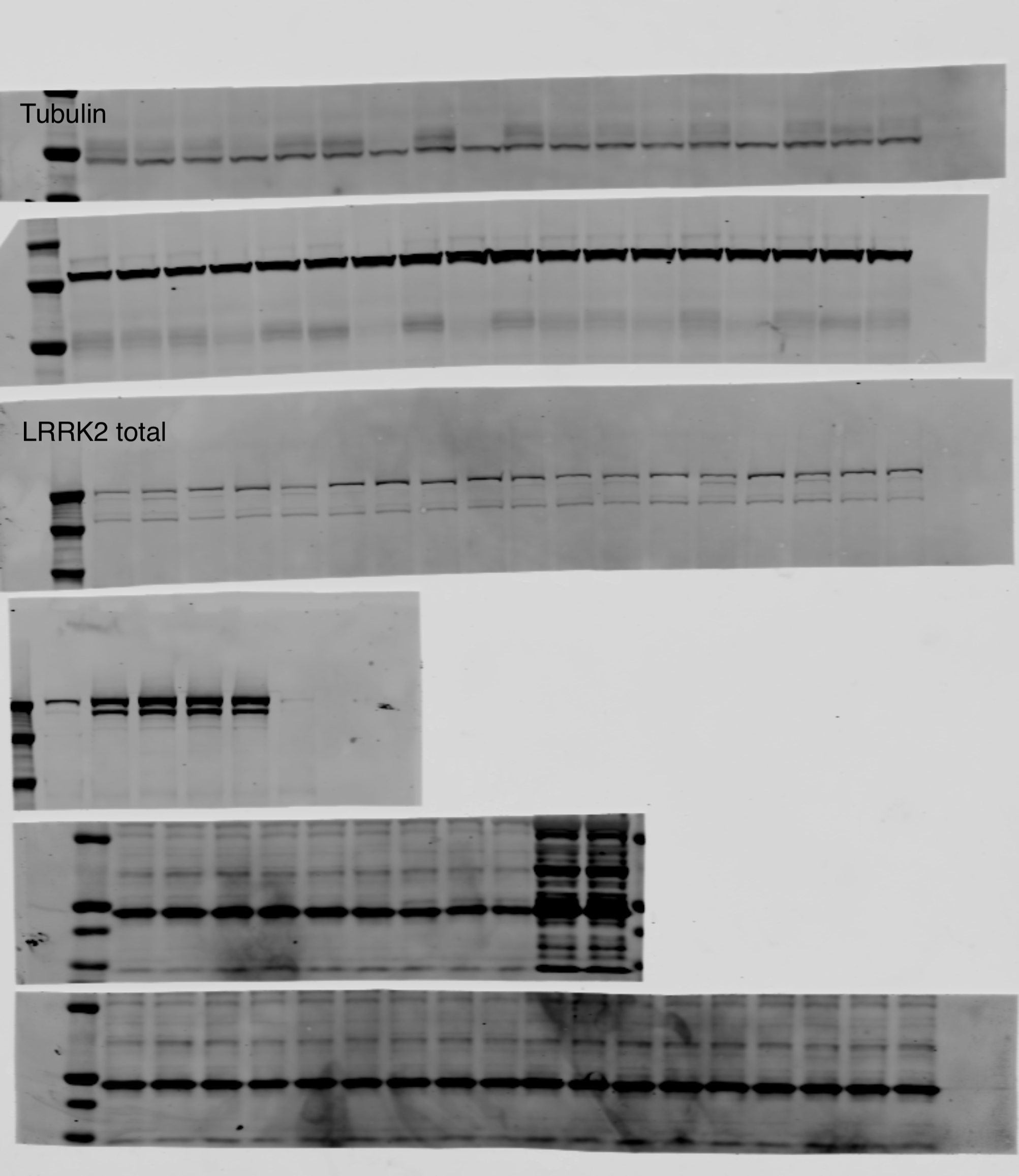

Supplement: Figure 2—figure supplement 2—source data 1. [file elife-87098-fig2-figsupp2-data1.zip › Figure 2-figure supplement 2-source data 1/annotated/Figure 2 Figure Suppl 2 Kidney_re-probed_700.tif]

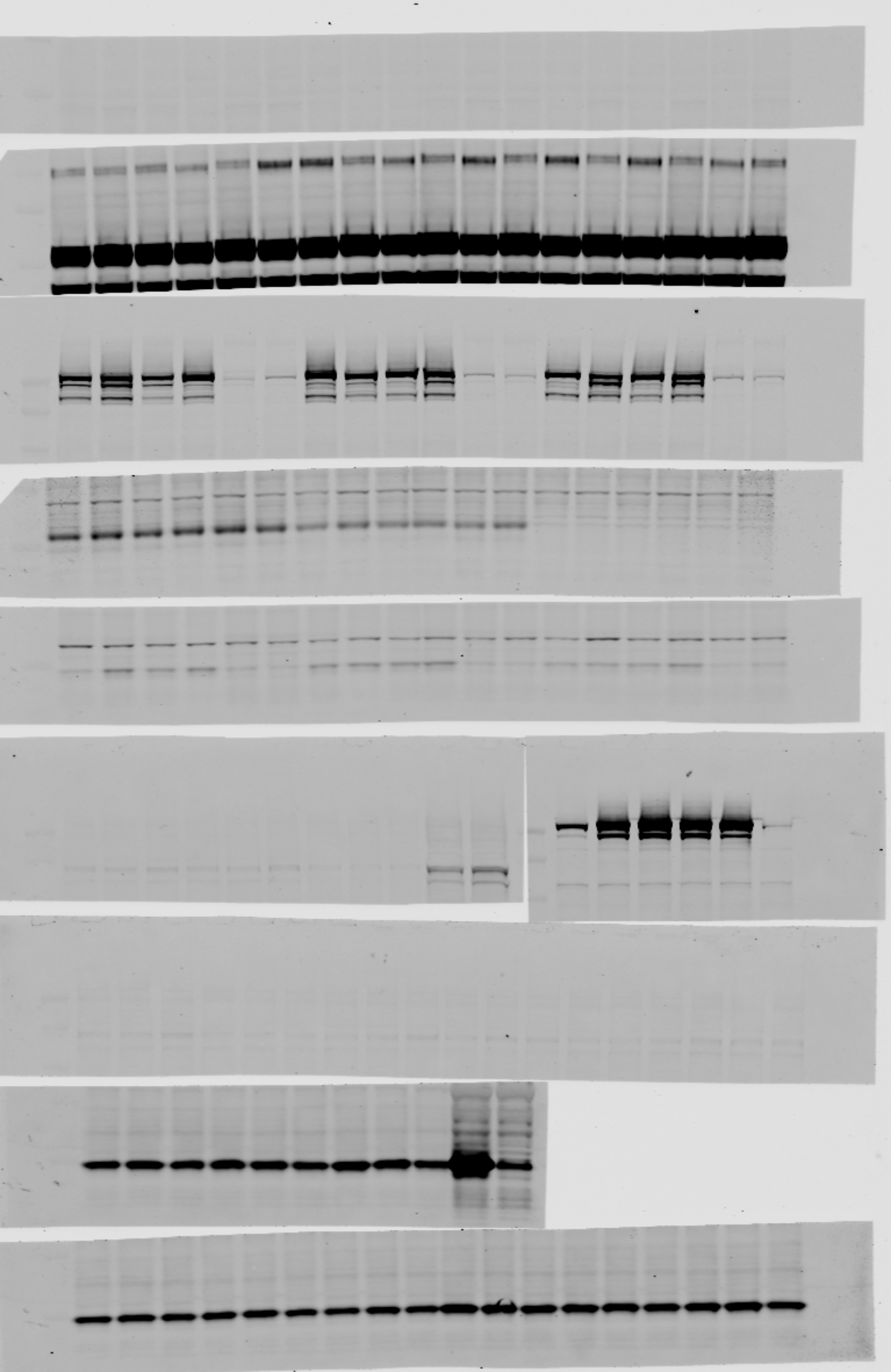

Supplement: Figure 2—figure supplement 2—source data 1. [file elife-87098-fig2-figsupp2-data1.zip › Figure 2-figure supplement 2-source data 1/raw images/Fig2_Suppl2_kidney_31-08-22_800.tif]

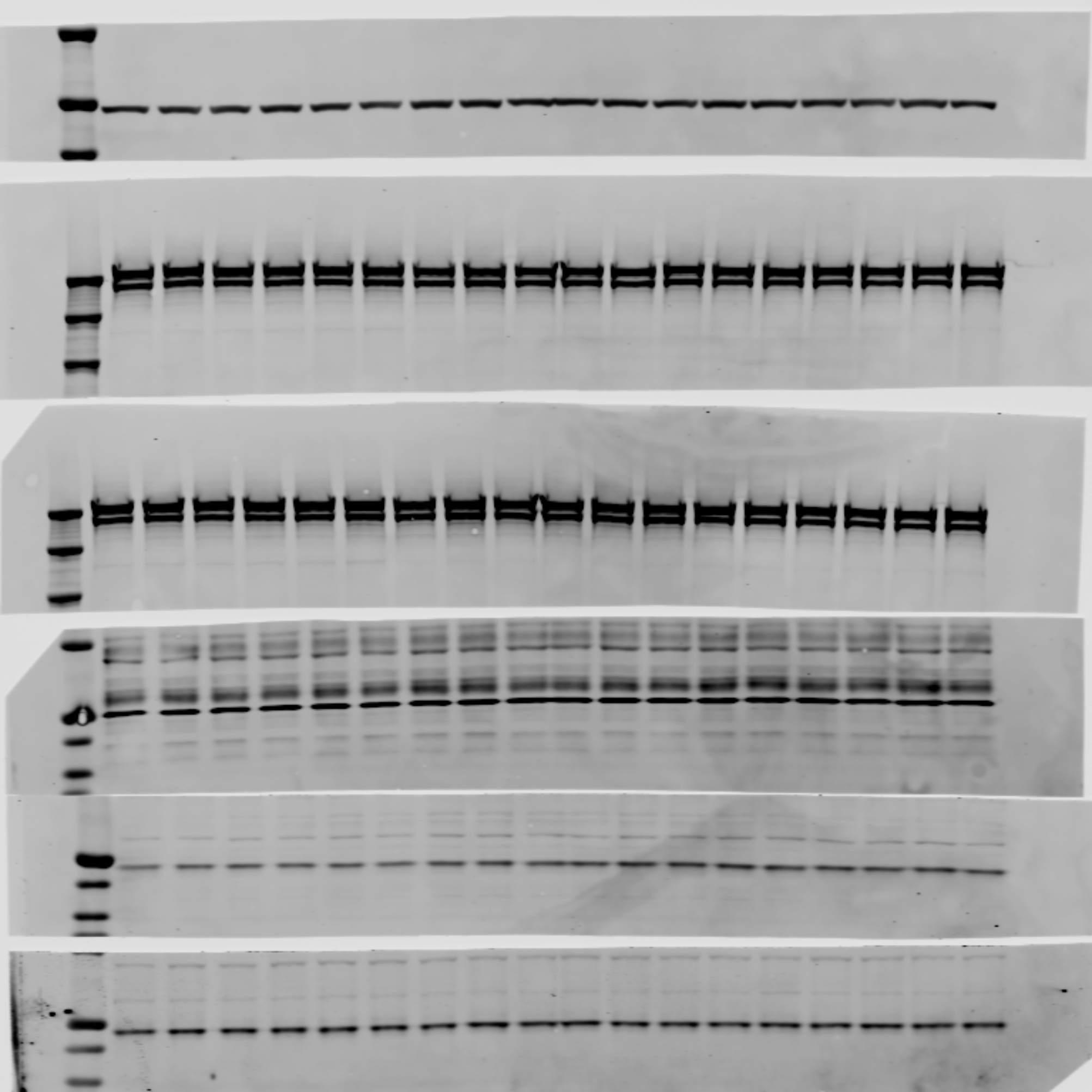

Supplement: Figure 2—figure supplement 2—source data 1. [file elife-87098-fig2-figsupp2-data1.zip › Figure 2-figure supplement 2-source data 1/raw images/Fig2_Suppl2_kidney_01-09-22_700.tif]

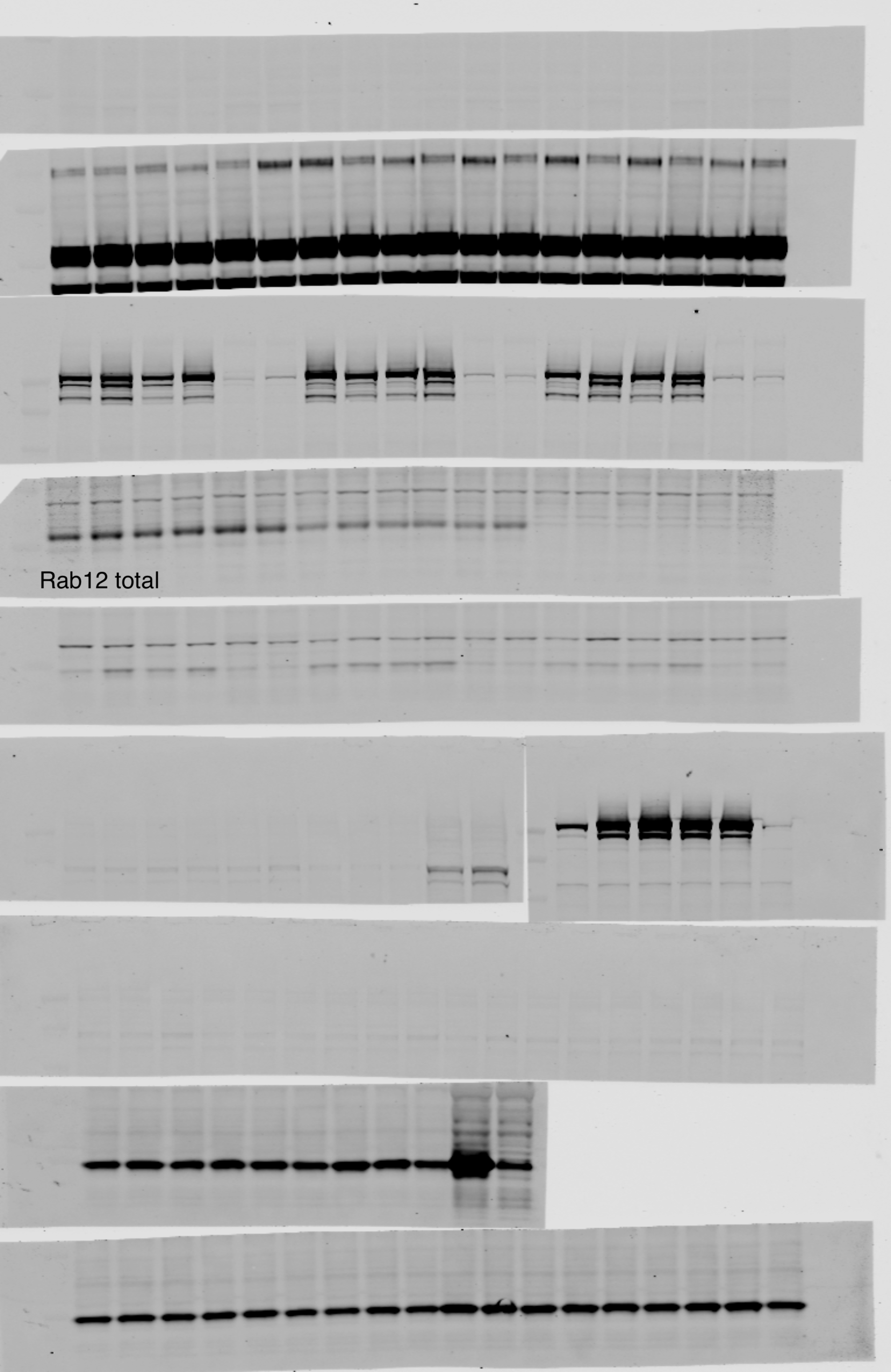

Supplement: Figure 2—figure supplement 2—source data 1. [file elife-87098-fig2-figsupp2-data1.zip › Figure 2-figure supplement 2-source data 1/annotated/Figure 2 Figure Suppl 2 Kidney_800-1.tif]

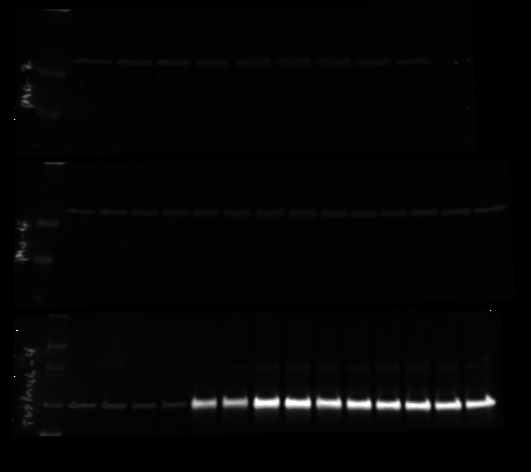

Supplement: Figure 3—source data 1. [file elife-87098-fig3-data1.zip › Figure 3-source data 1/Figure 3A-images/700_5.tif]

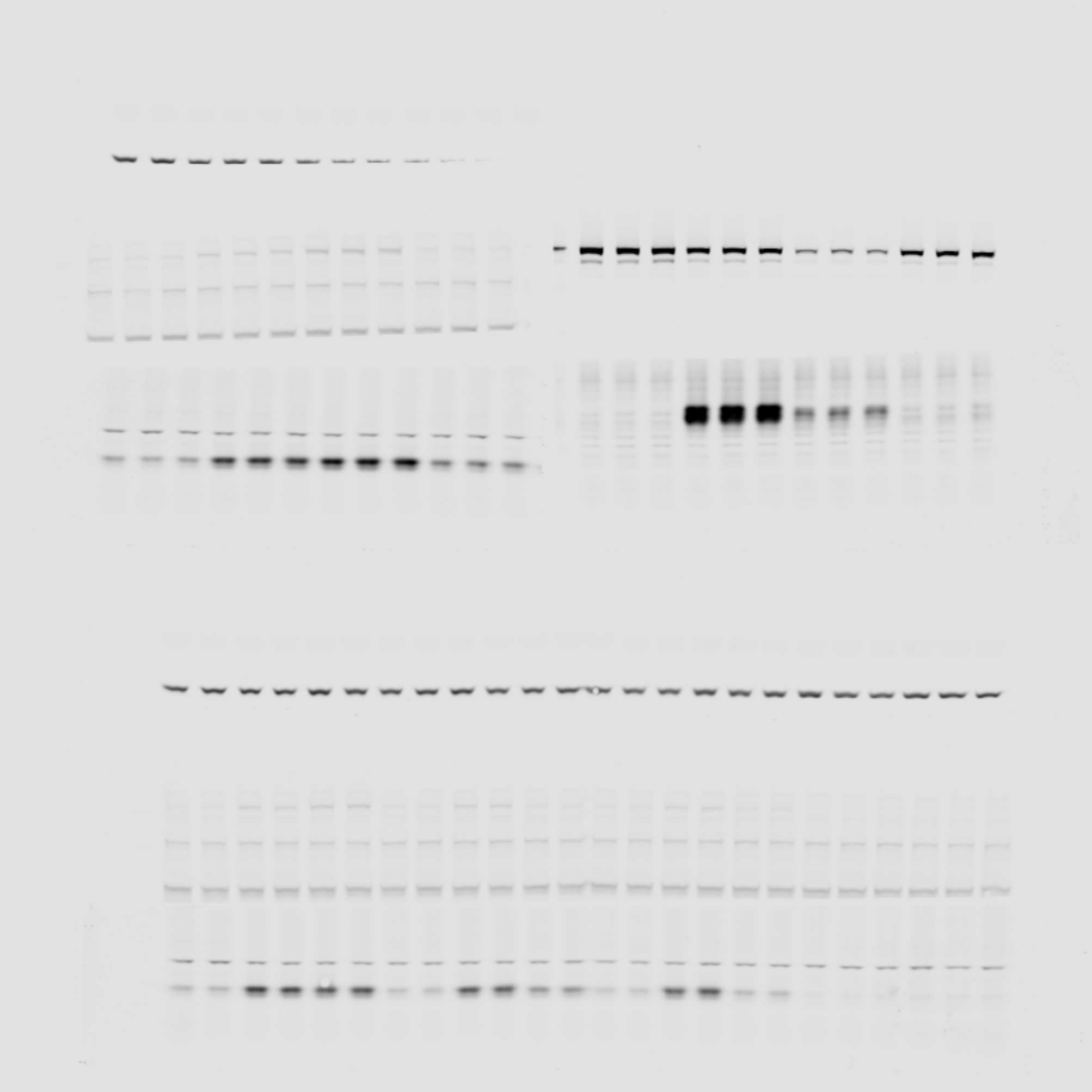

Supplement: Figure 3—source data 1. [file elife-87098-fig3-data1.zip › Figure 3-source data 1/Figure 3G-images/3G_800.tif]

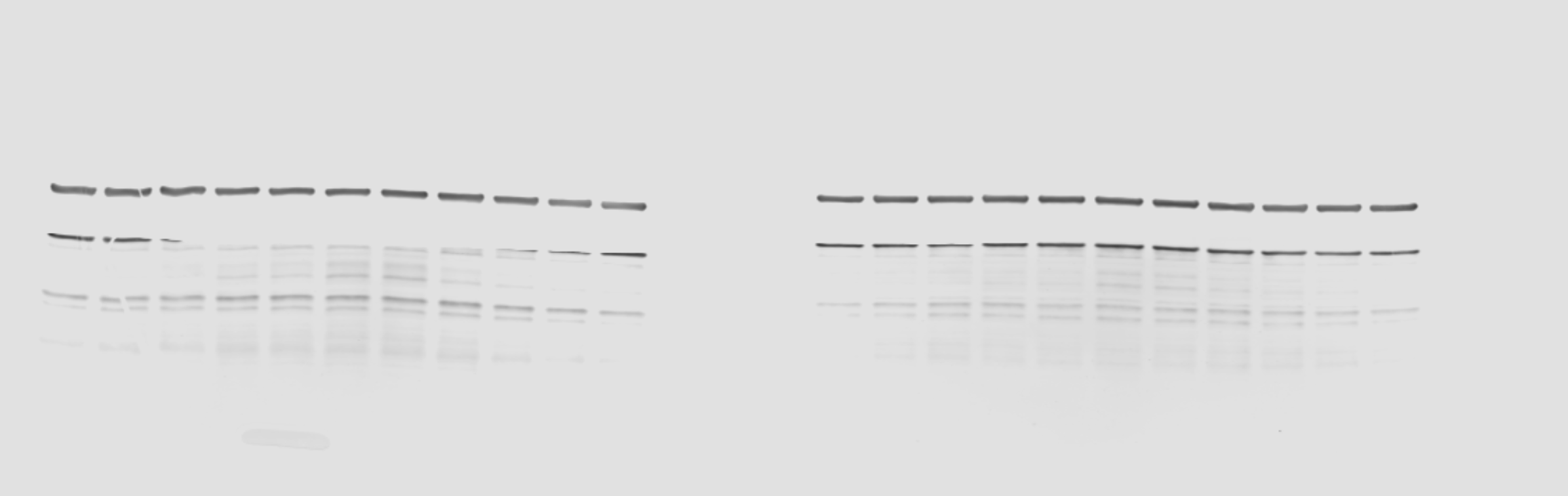

Supplement: Figure 3—source data 1. [file elife-87098-fig3-data1.zip › Figure 3-source data 1/Figure 3C-images/1_tubulin.tif]

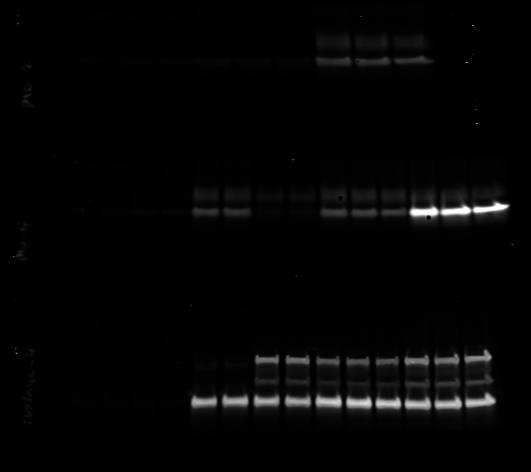

Supplement: Figure 3—source data 1. [file elife-87098-fig3-data1.zip › Figure 3-source data 1/Figure 3A-images/800_5.tif]

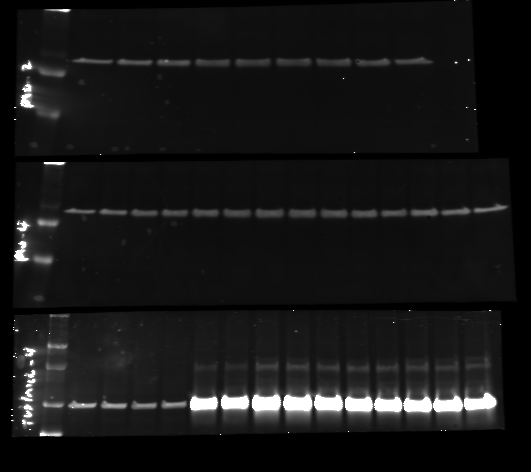

Supplement: Figure 3—source data 1. [file elife-87098-fig3-data1.zip › Figure 3-source data 1/Figure 3A-images/700_6.tif]

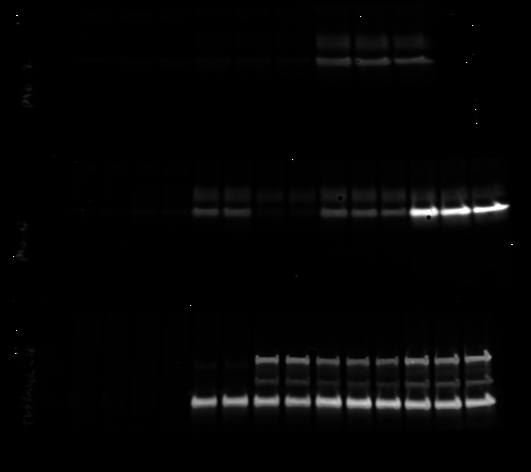

Supplement: Figure 3—source data 1. [file elife-87098-fig3-data1.zip › Figure 3-source data 1/Figure 3A-images/800_4.tif]

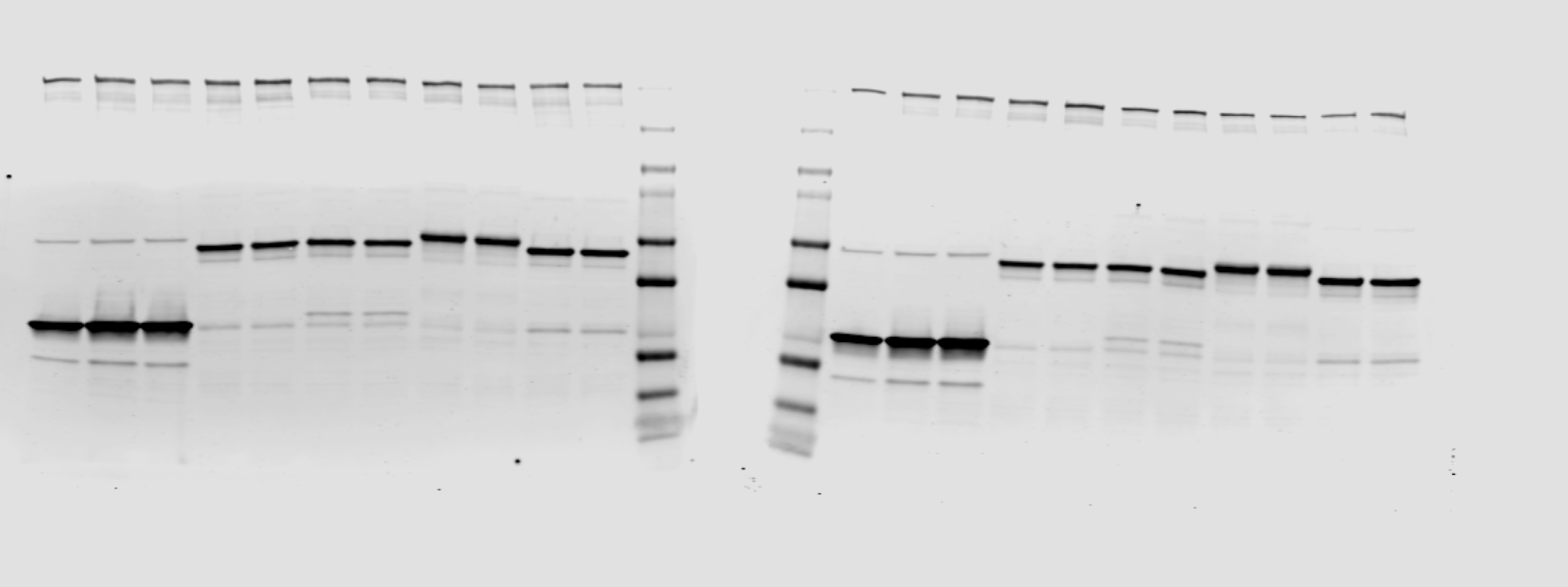

Supplement: Figure 3—source data 1. [file elife-87098-fig3-data1.zip › Figure 3-source data 1/Figure 3C-images/1_first gel_680.tif]

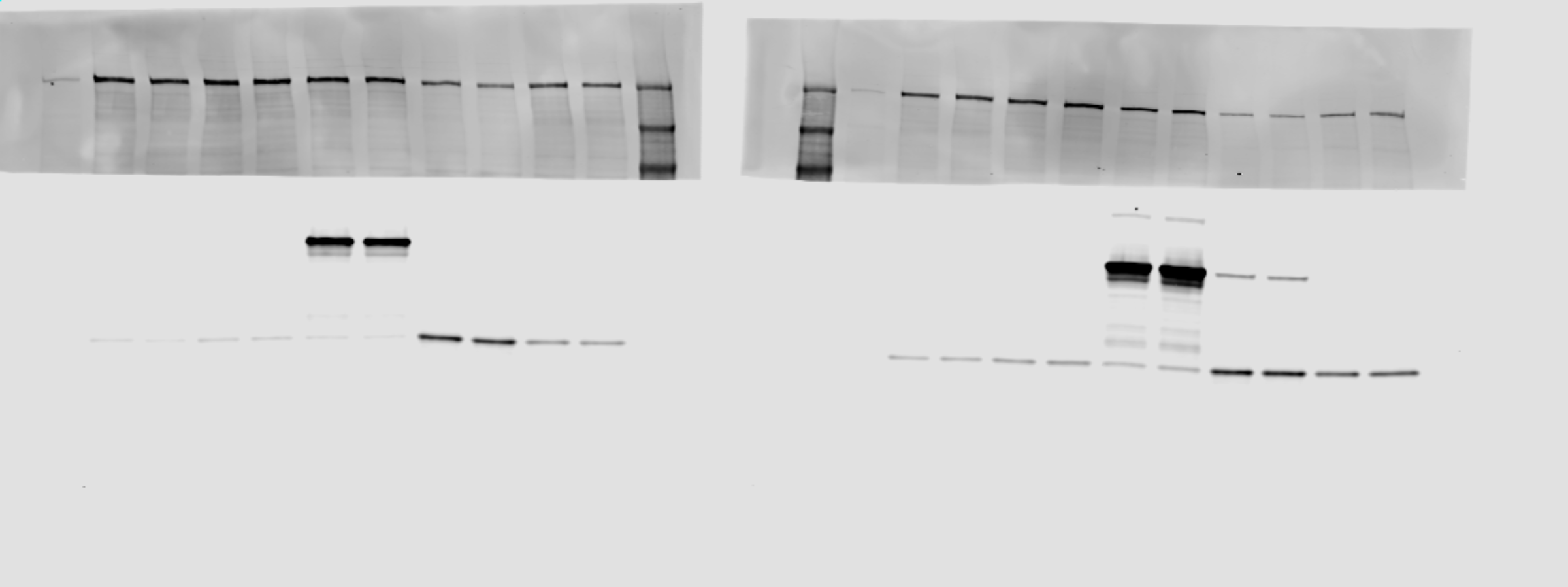

Supplement: Figure 3—source data 1. [file elife-87098-fig3-data1.zip › Figure 3-source data 1/Figure 3C-images/1_first gel_800.tif]

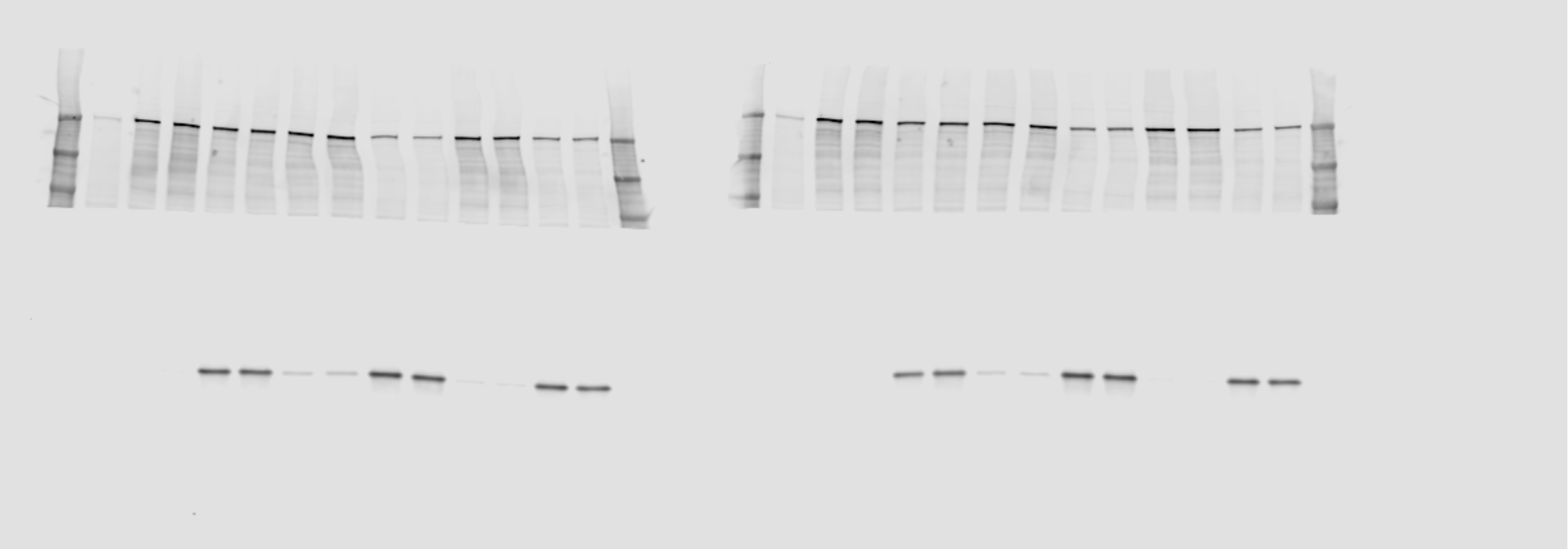

Supplement: Figure 3—source data 1. [file elife-87098-fig3-data1.zip › Figure 3-source data 1/Figure 3E-images/first gel 800.tif]

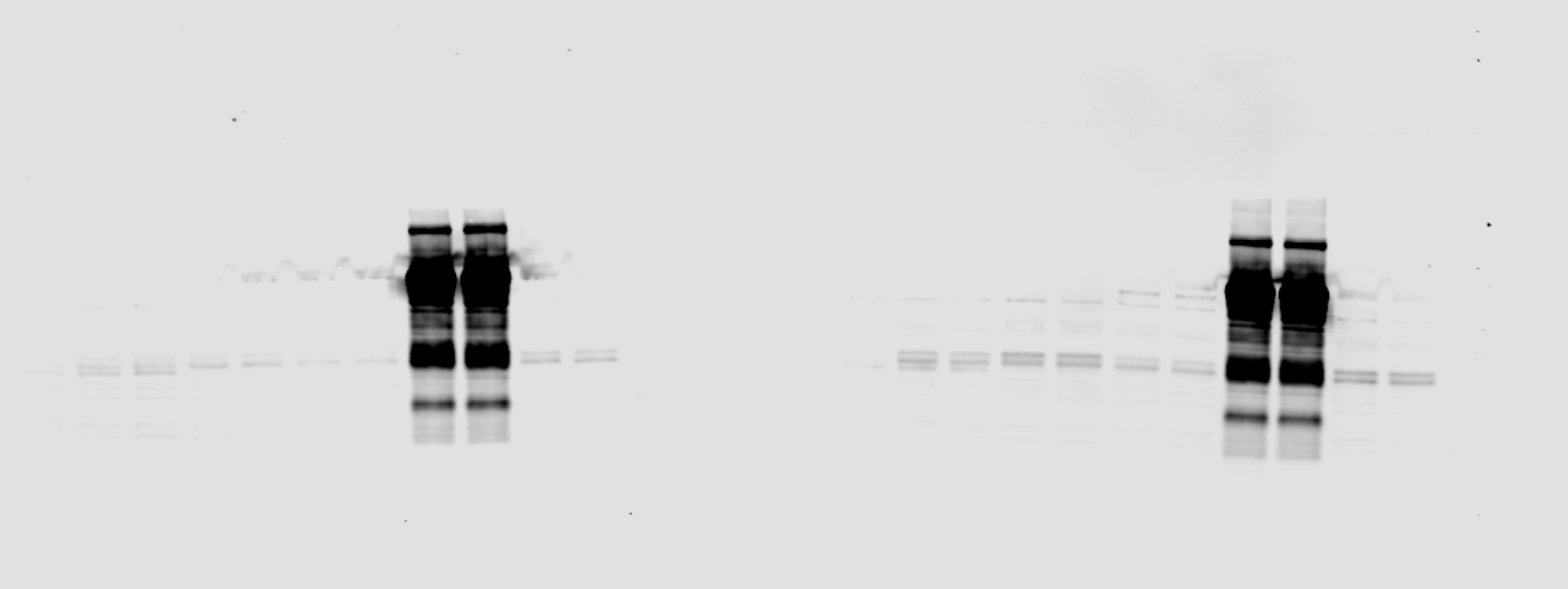

Supplement: Figure 3—source data 1. [file elife-87098-fig3-data1.zip › Figure 3-source data 1/Figure 3C-images/1_second gel_800.tif]

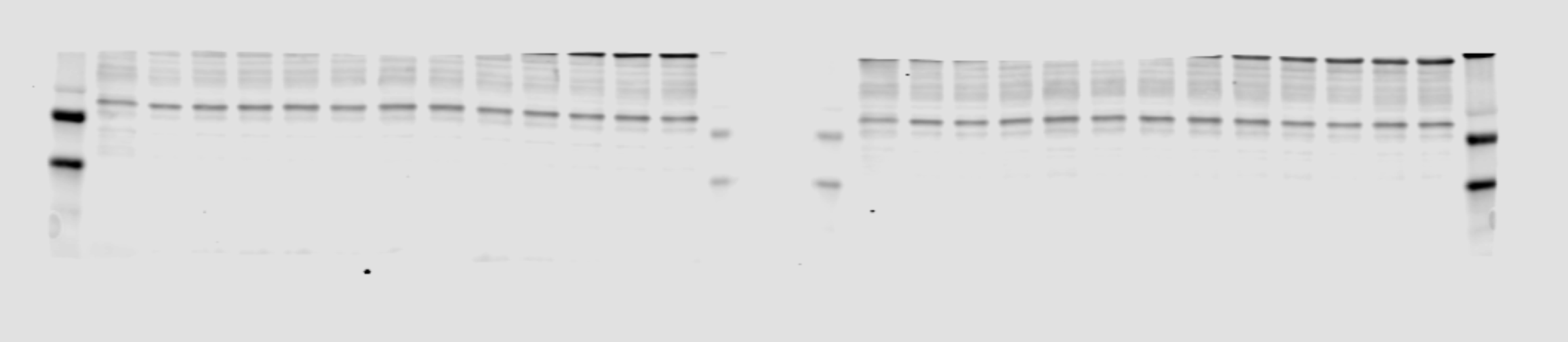

Supplement: Figure 3—source data 1. [file elife-87098-fig3-data1.zip › Figure 3-source data 1/Figure 3E-images/totalRab10 680.tif]

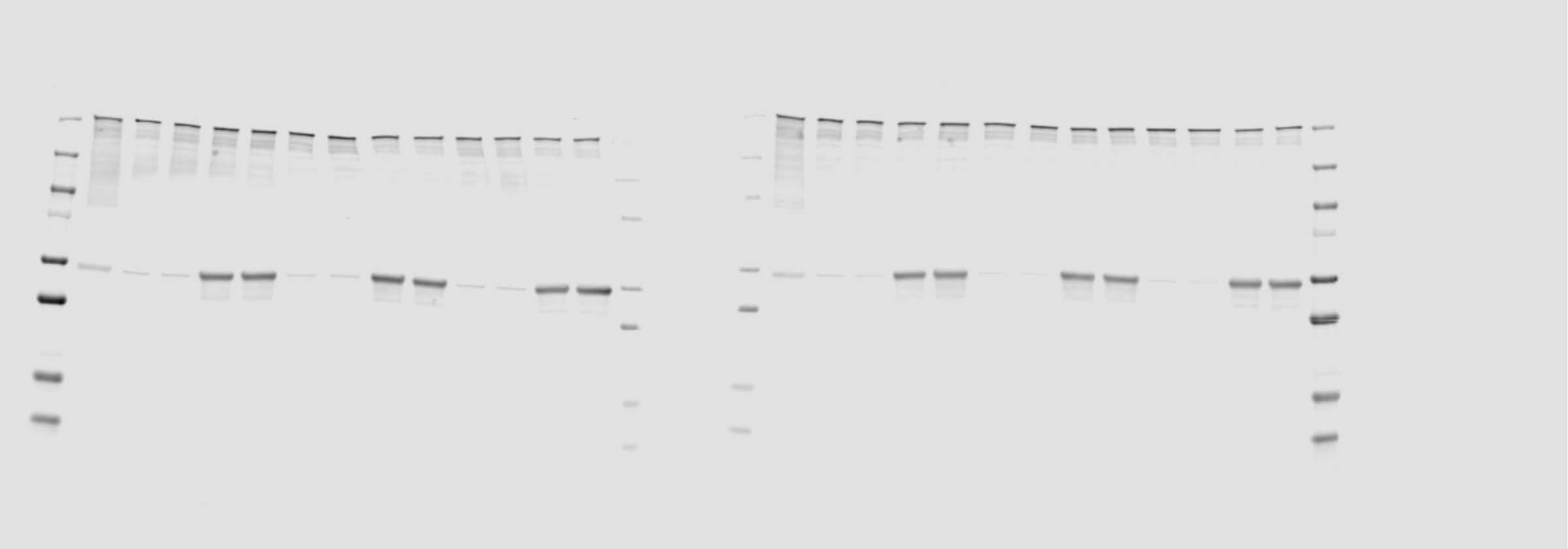

Supplement: Figure 3—source data 1. [file elife-87098-fig3-data1.zip › Figure 3-source data 1/Figure 3E-images/first gel 680.tif]

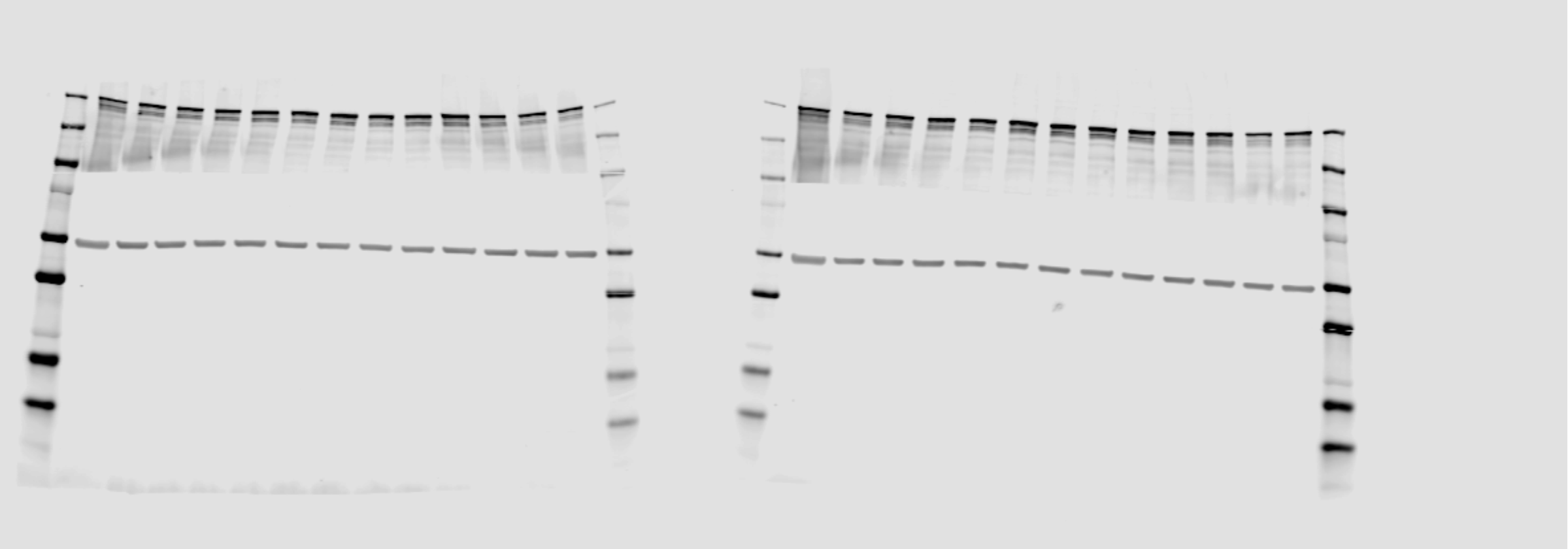

Supplement: Figure 3—source data 1. [file elife-87098-fig3-data1.zip › Figure 3-source data 1/Figure 3E-images/second gel 680.tif]

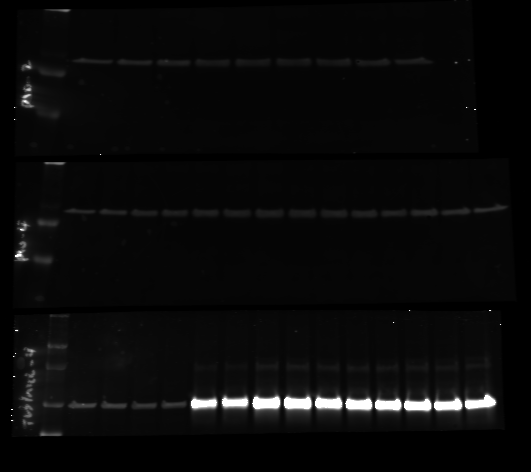

Supplement: Figure 3—source data 1. [file elife-87098-fig3-data1.zip › Figure 3-source data 1/Figure 3A-images/700_4.tif]

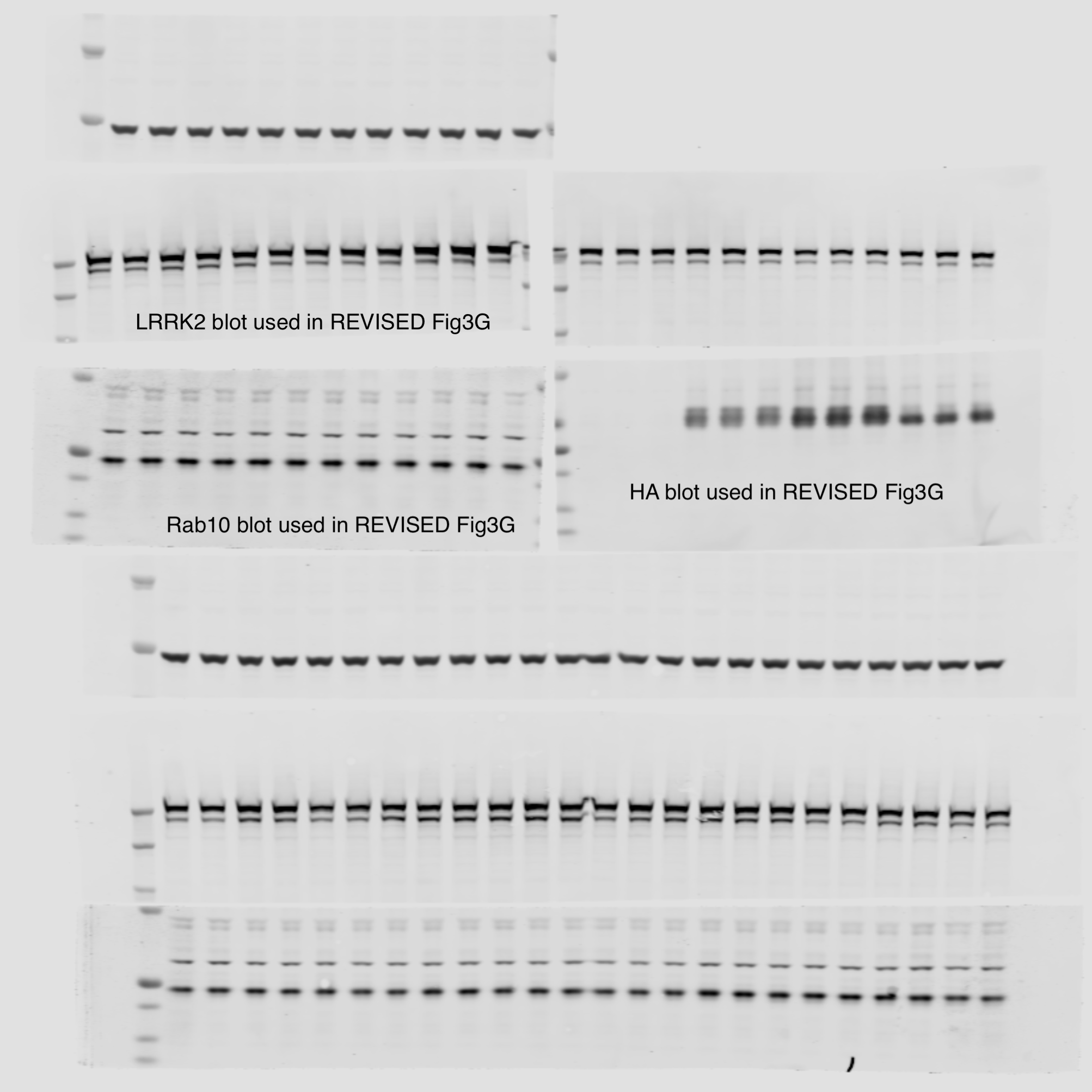

Supplement: Figure 3—source data 1. [file elife-87098-fig3-data1.zip › Figure 3-source data 1/annotated/REVISED-Fig3G_28-04-2023_700.tif]

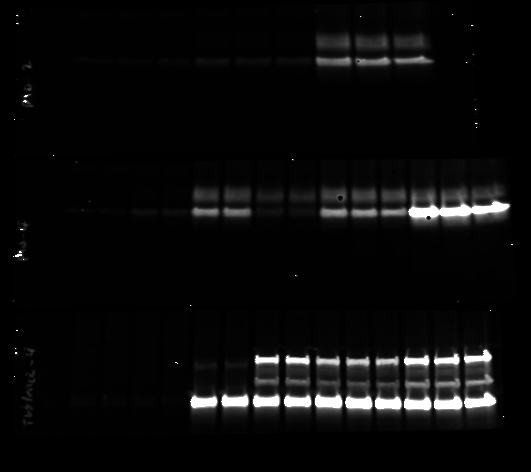

Supplement: Figure 3—source data 1. [file elife-87098-fig3-data1.zip › Figure 3-source data 1/Figure 3A-images/800_6.tif]

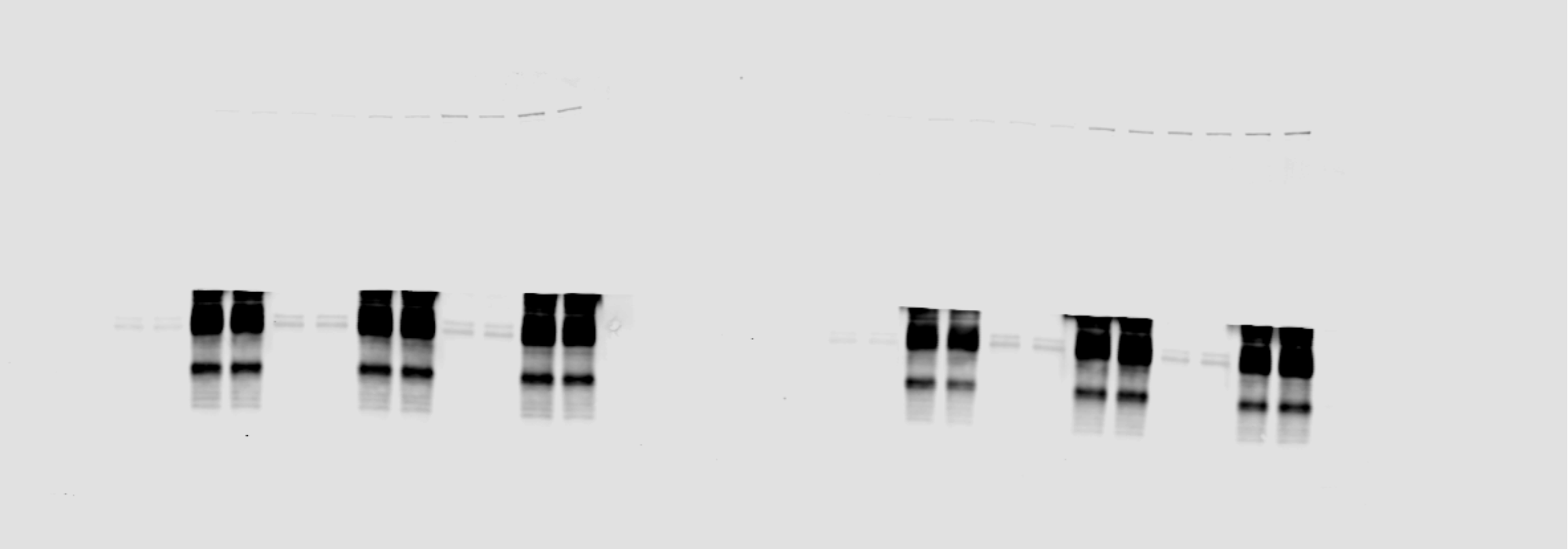

Supplement: Figure 3—source data 1. [file elife-87098-fig3-data1.zip › Figure 3-source data 1/Figure 3E-images/second gel 800 dark.tif]

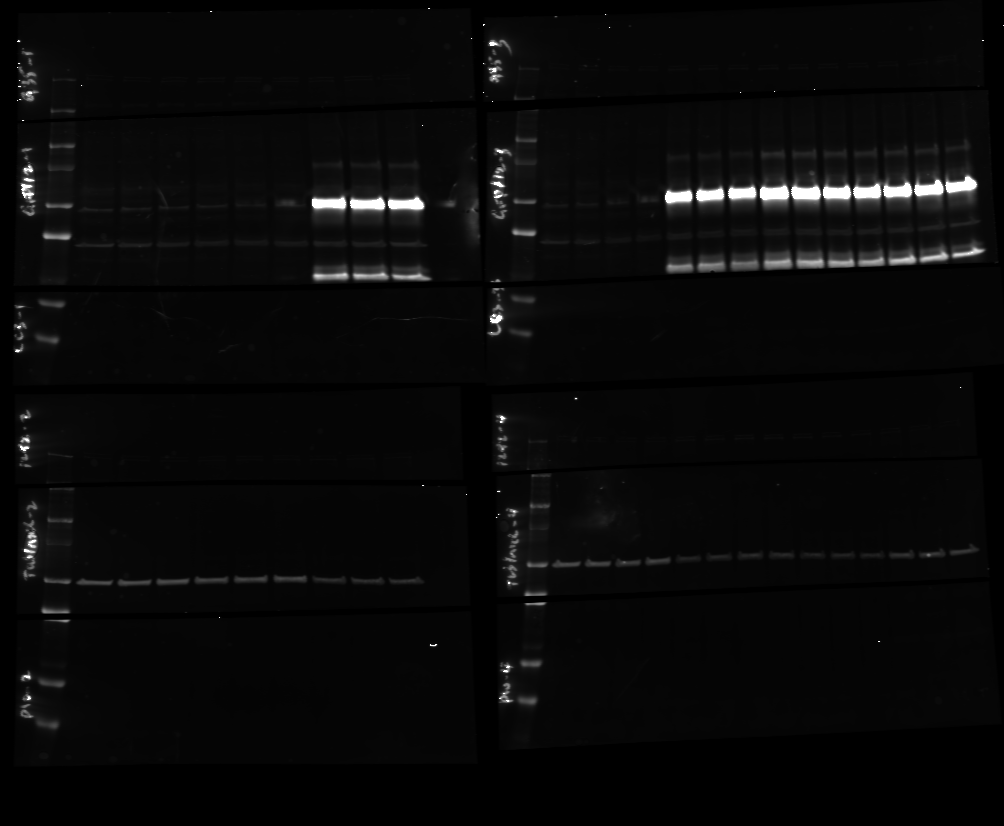

Supplement: Figure 3—source data 1. [file elife-87098-fig3-data1.zip › Figure 3-source data 1/Figure 3A-images/700_3.tif]

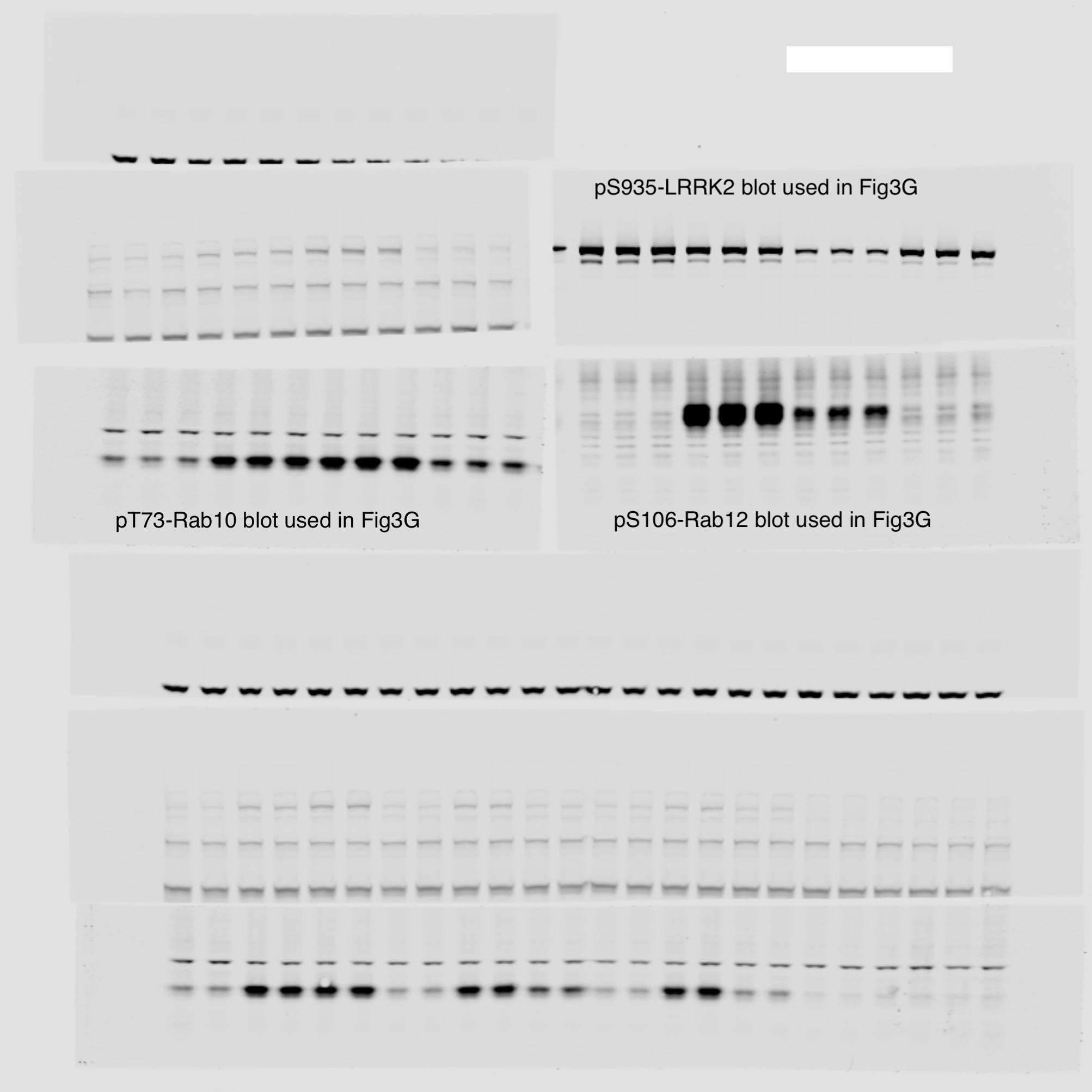

Supplement: Figure 3—source data 1. [file elife-87098-fig3-data1.zip › Figure 3-source data 1/annotated/REVISED-Fig3G_28-04-2023_800.tif]

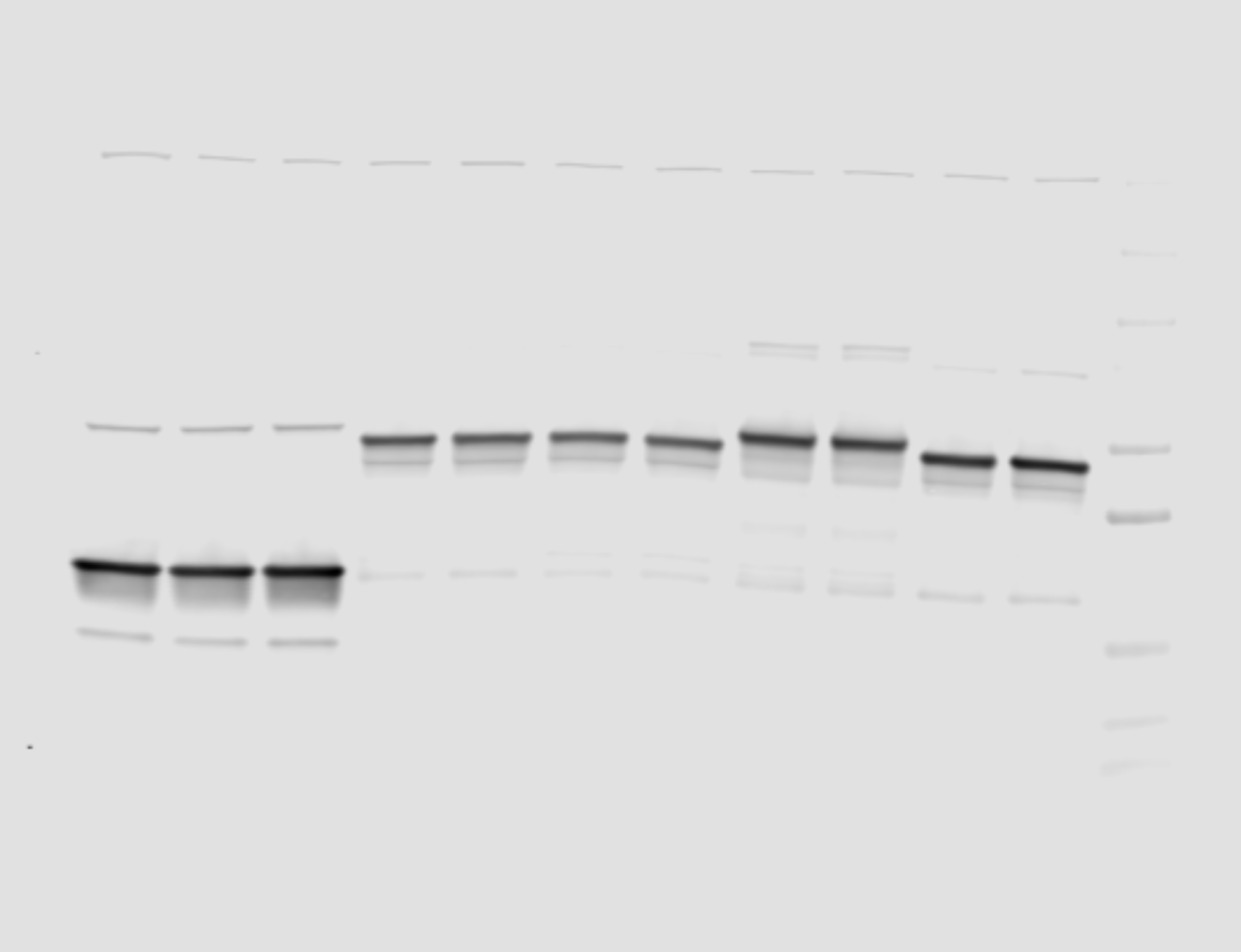

Supplement: Figure 3—source data 1. [file elife-87098-fig3-data1.zip › Figure 3-source data 1/Figure 3C-images/2_first gel_680.tif]

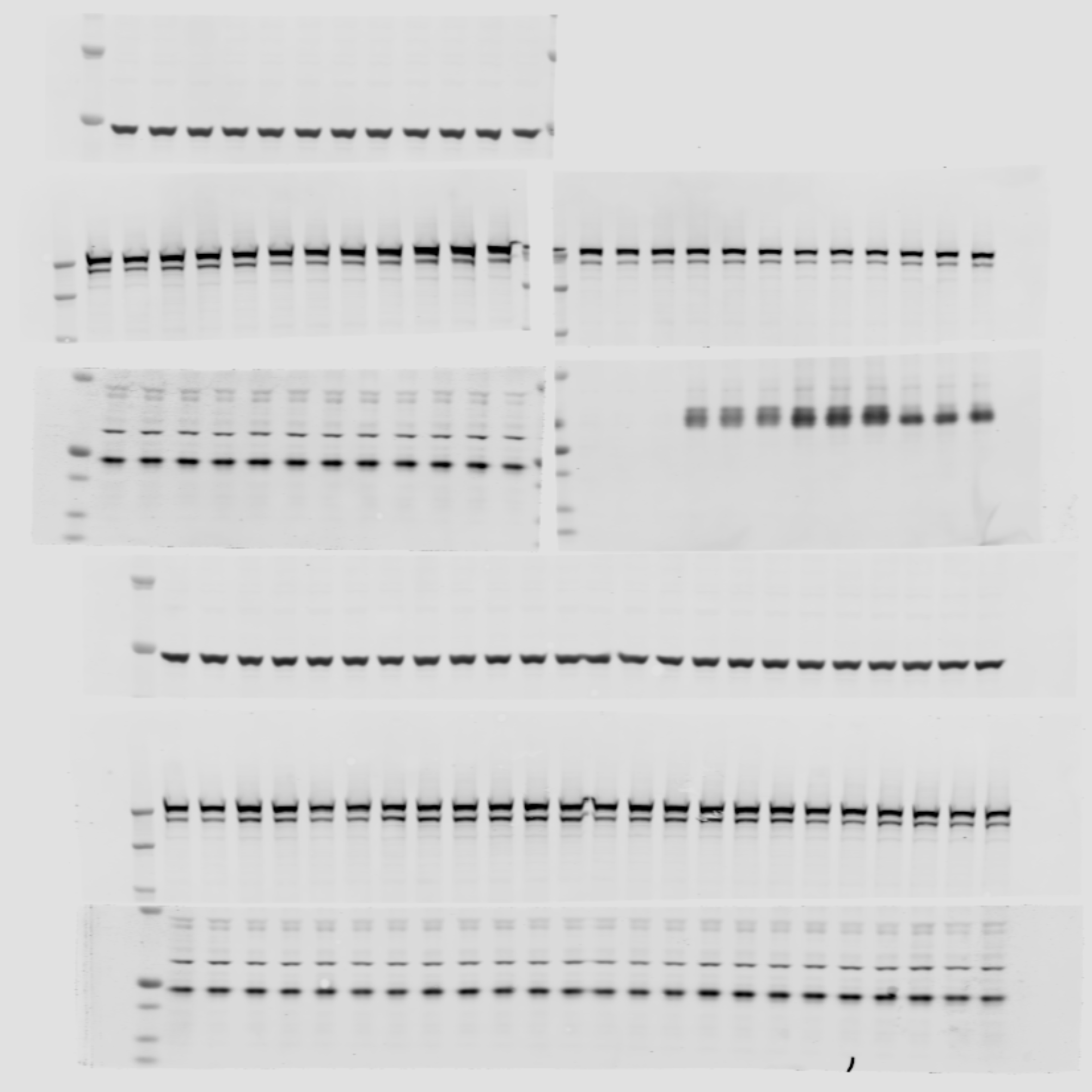

Supplement: Figure 3—source data 1. [file elife-87098-fig3-data1.zip › Figure 3-source data 1/Figure 3G-images/3G_700.tif]

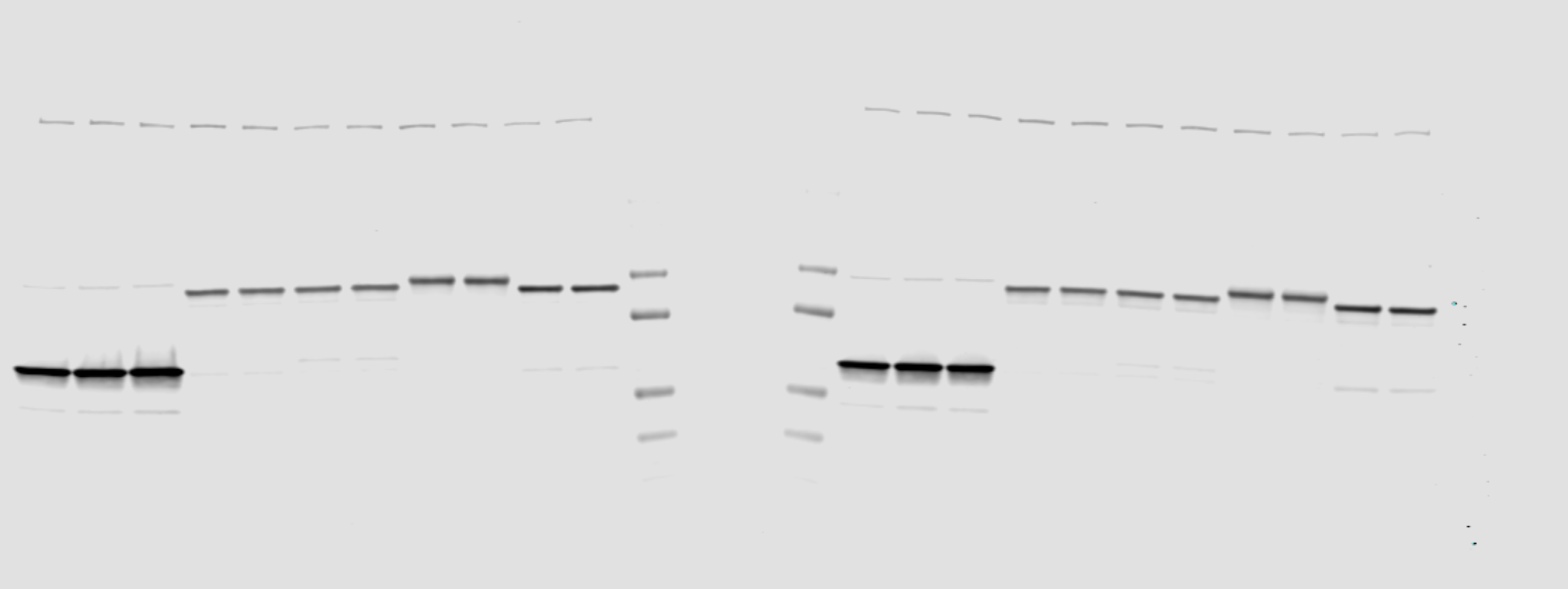

Supplement: Figure 3—source data 1. [file elife-87098-fig3-data1.zip › Figure 3-source data 1/Figure 3C-images/1_second gel_680.tif]

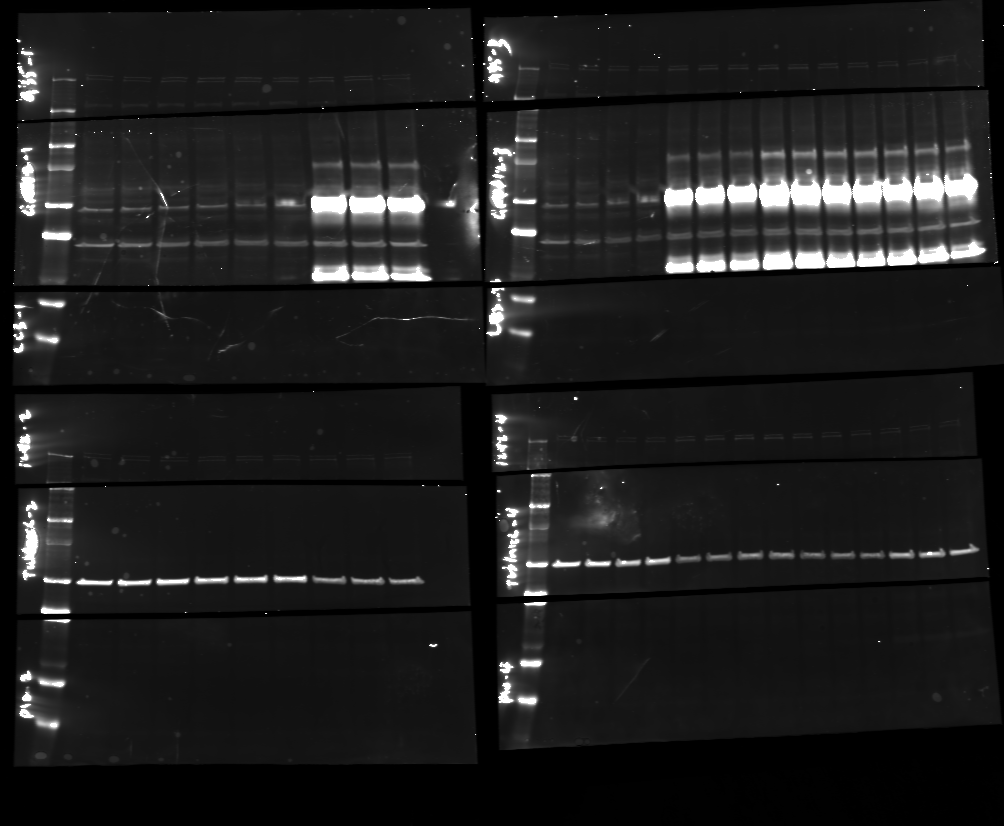

Supplement: Figure 3—source data 1. [file elife-87098-fig3-data1.zip › Figure 3-source data 1/Figure 3A-images/700_2.tif]

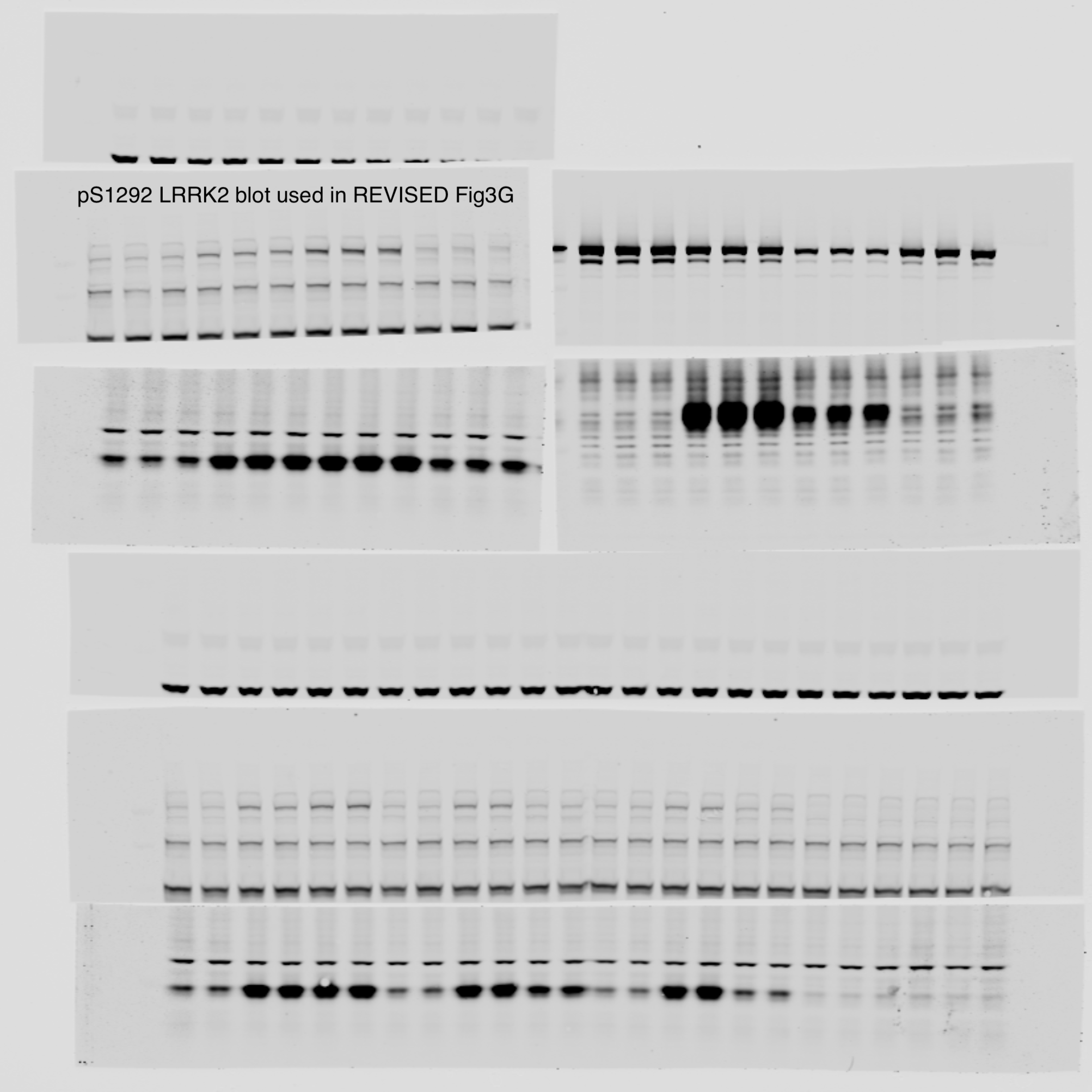

Supplement: Figure 3—source data 1. [file elife-87098-fig3-data1.zip › Figure 3-source data 1/annotated/REVISED-Fig3G_28-04-2023_800-high.tif]

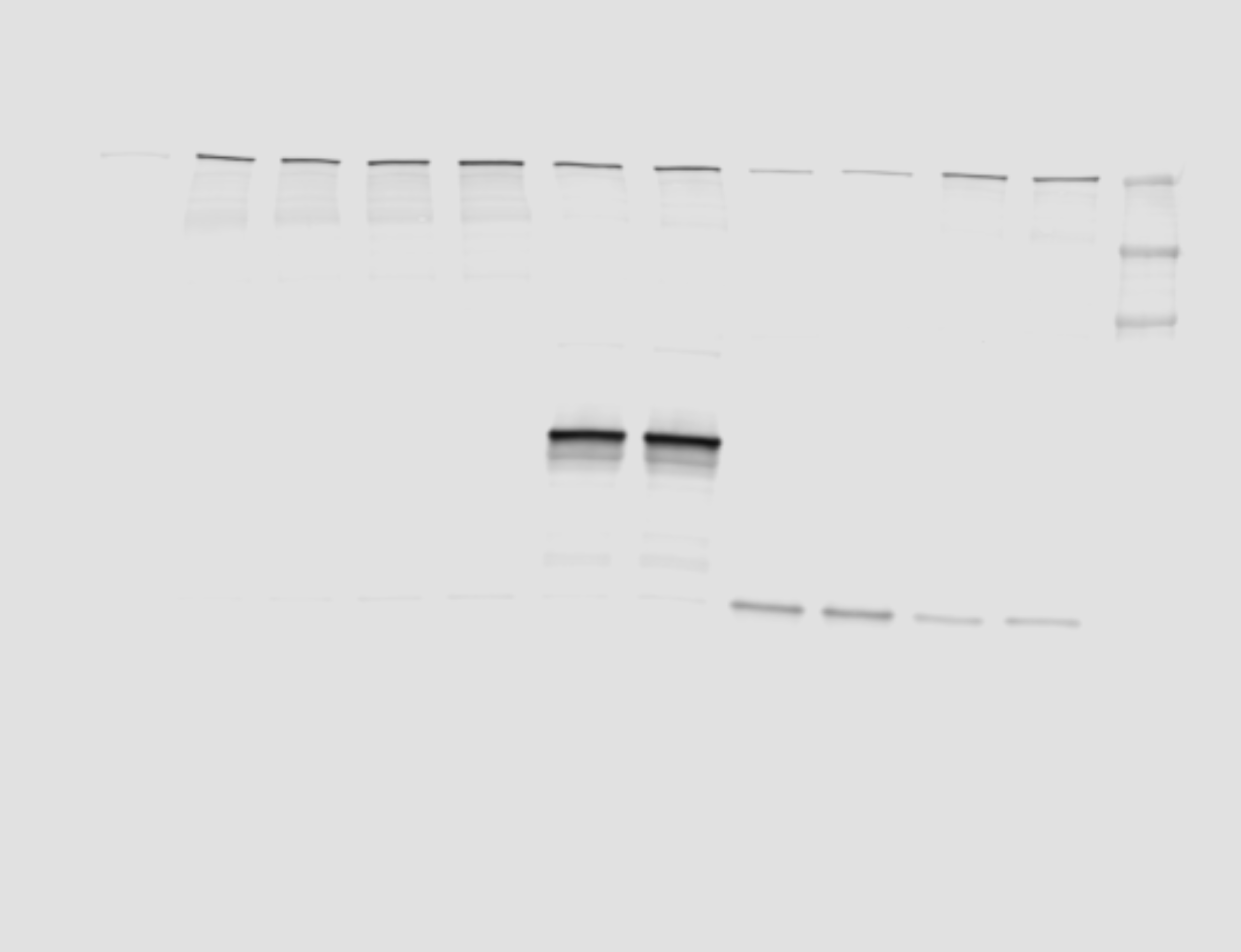

Supplement: Figure 3—source data 1. [file elife-87098-fig3-data1.zip › Figure 3-source data 1/Figure 3C-images/2_first gel_800.tif]

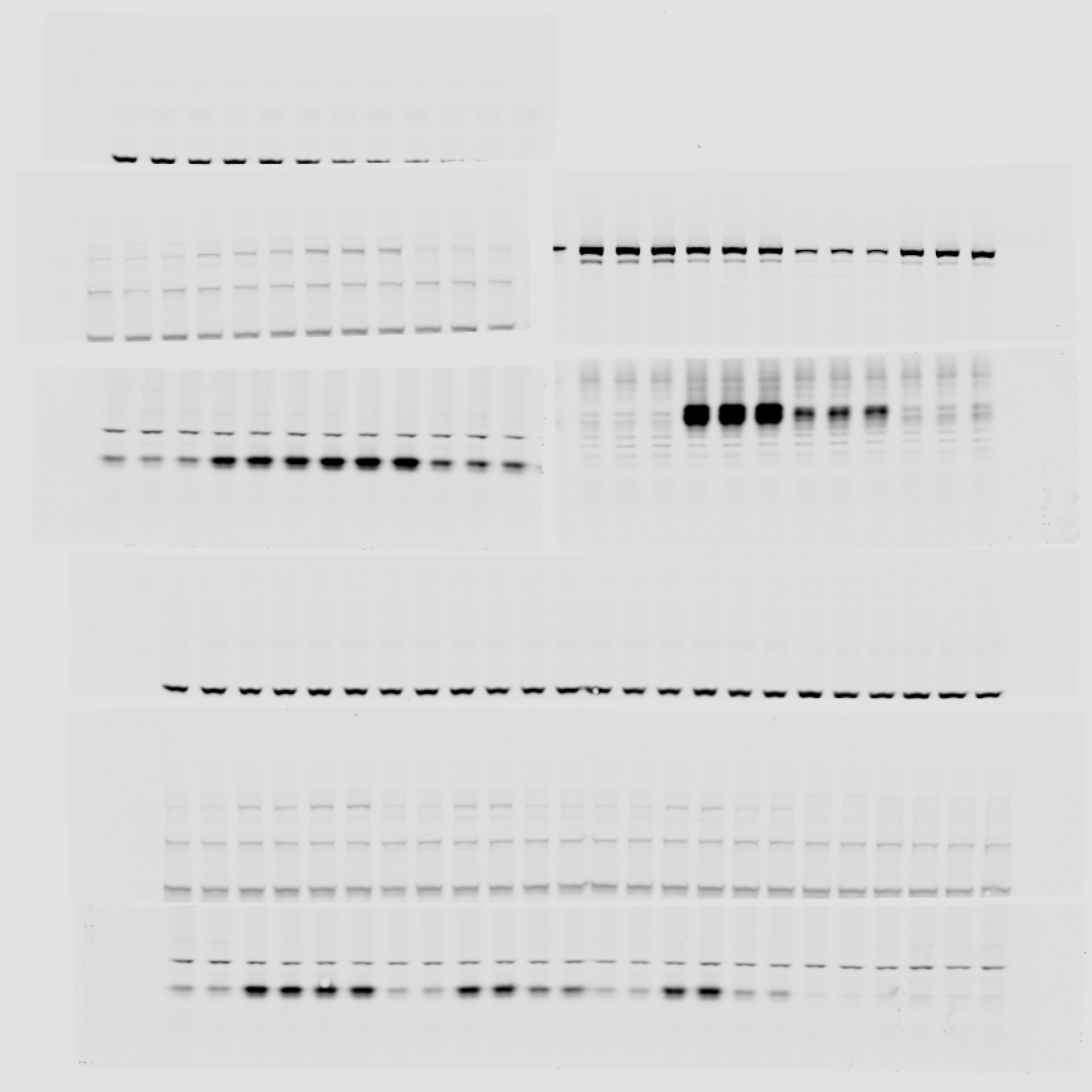

Supplement: Figure 3—source data 1. [file elife-87098-fig3-data1.zip › Figure 3-source data 1/Figure 3G-images/3G_800-high.tif]

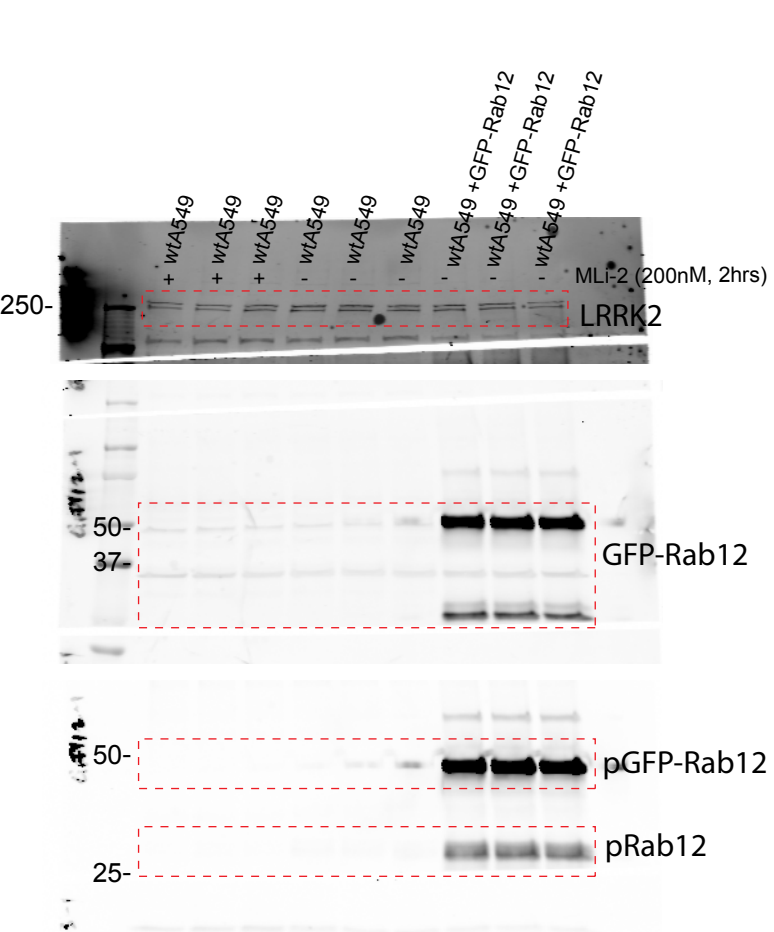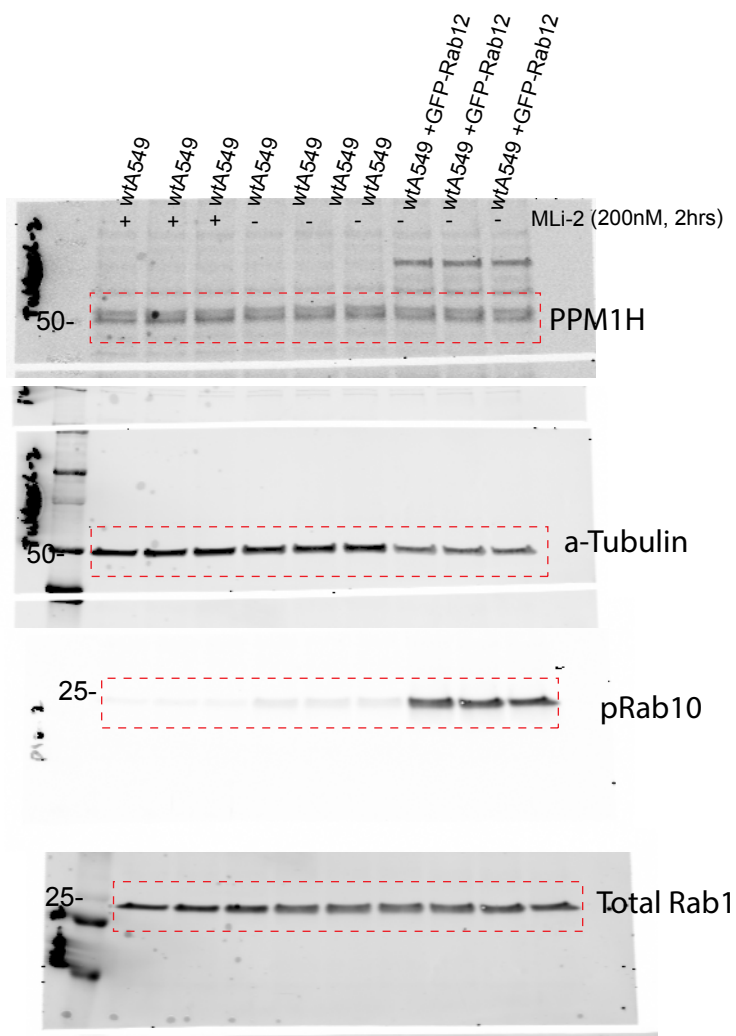

Supplement: Figure 3—source data 1. [file elife-87098-fig3-data1.zip › Figure 3-source data 1/annotated/Supporting material for figure 3A_B_annotated blots.pdf]

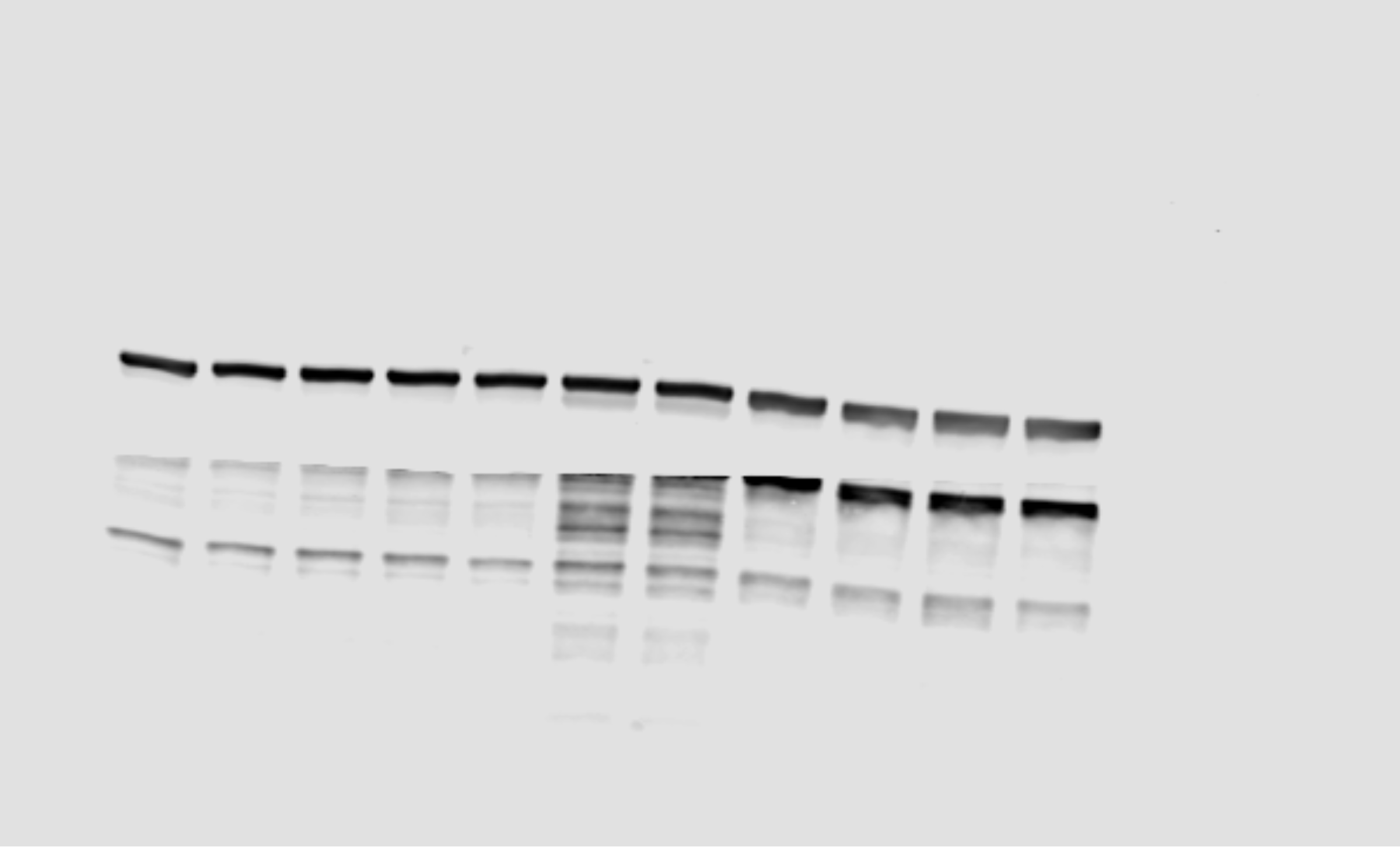

Supplement: Figure 3—source data 1. [file elife-87098-fig3-data1.zip › Figure 3-source data 1/Figure 3C-images/2_second gel_800.tif]

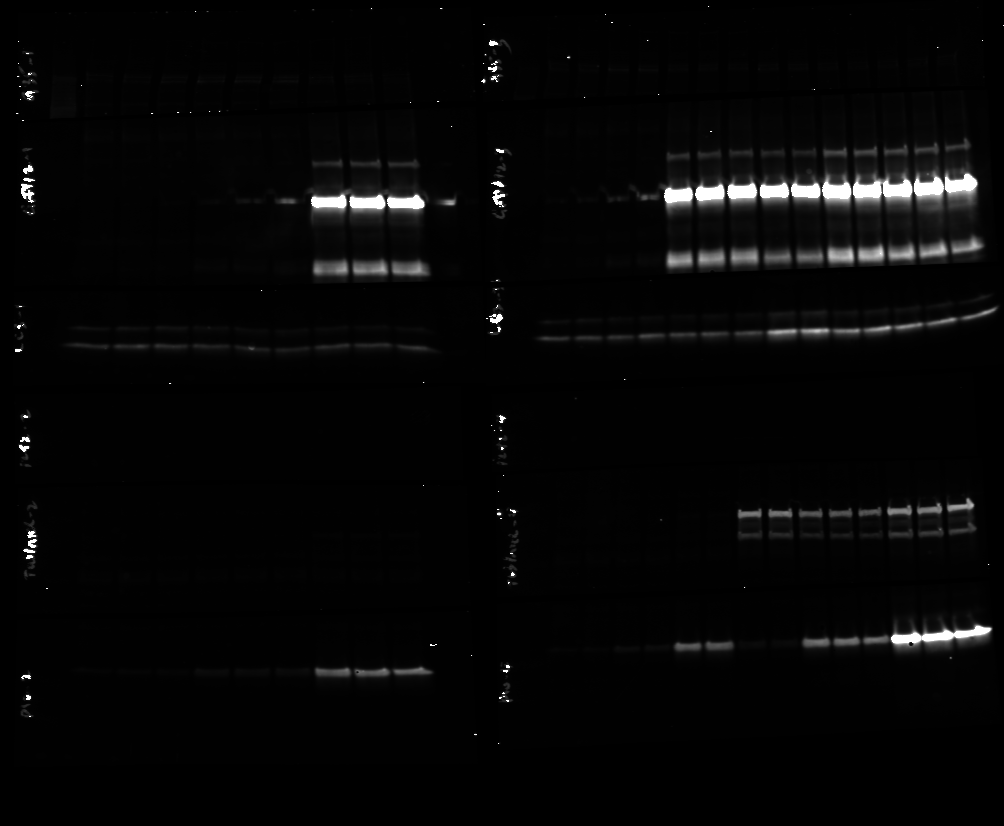

Supplement: Figure 3—source data 1. [file elife-87098-fig3-data1.zip › Figure 3-source data 1/Figure 3A-images/800_2.tif]

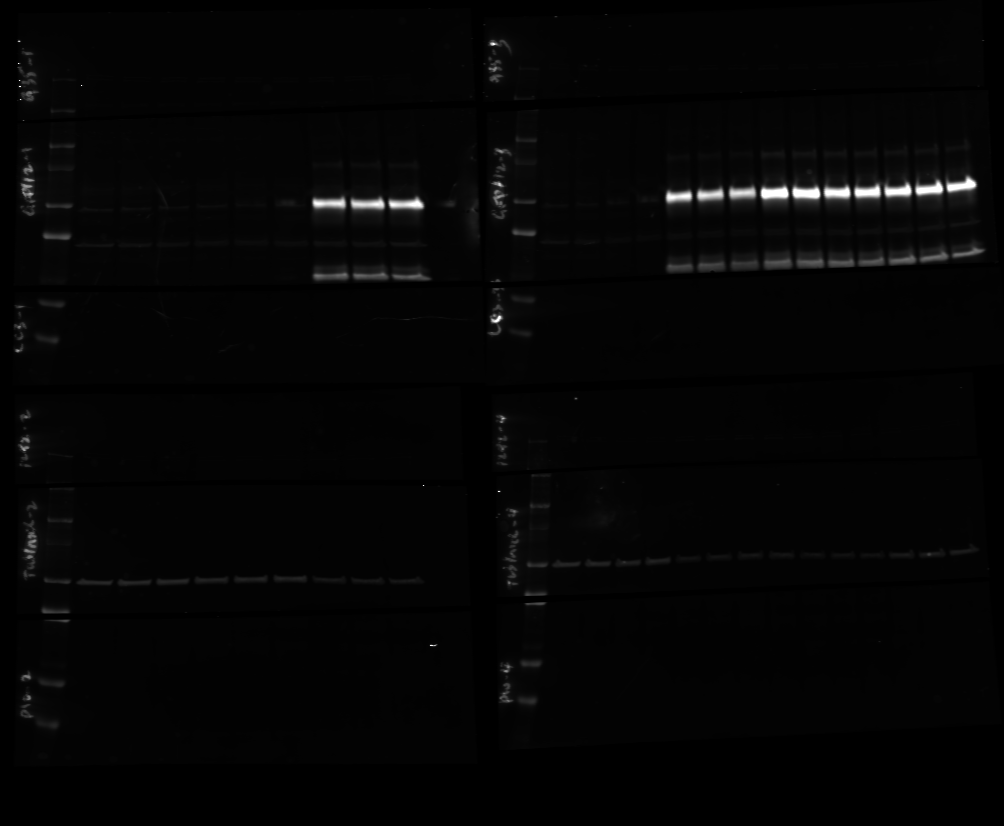

Supplement: Figure 3—source data 1. [file elife-87098-fig3-data1.zip › Figure 3-source data 1/Figure 3A-images/700_1.tif]

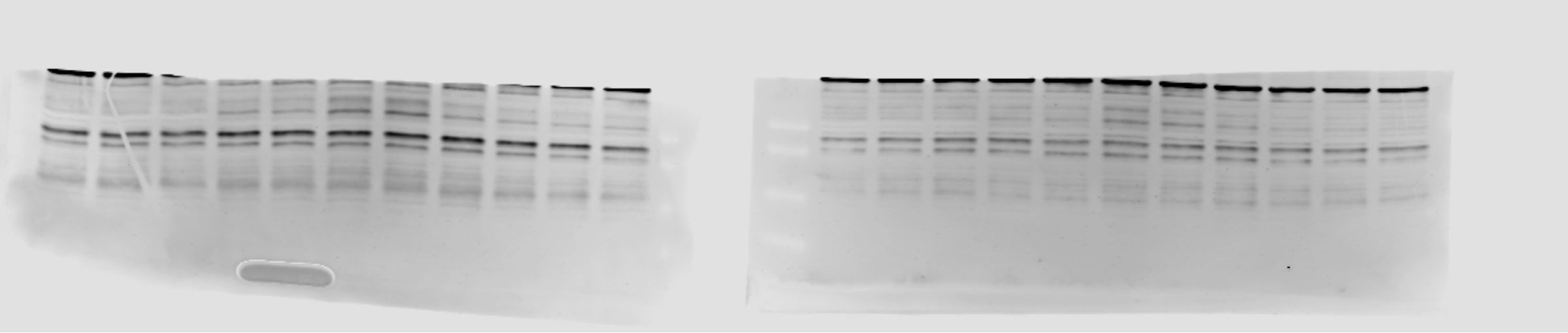

Supplement: Figure 3—source data 1. [file elife-87098-fig3-data1.zip › Figure 3-source data 1/Figure 3C-images/1_totalRab10.tif]

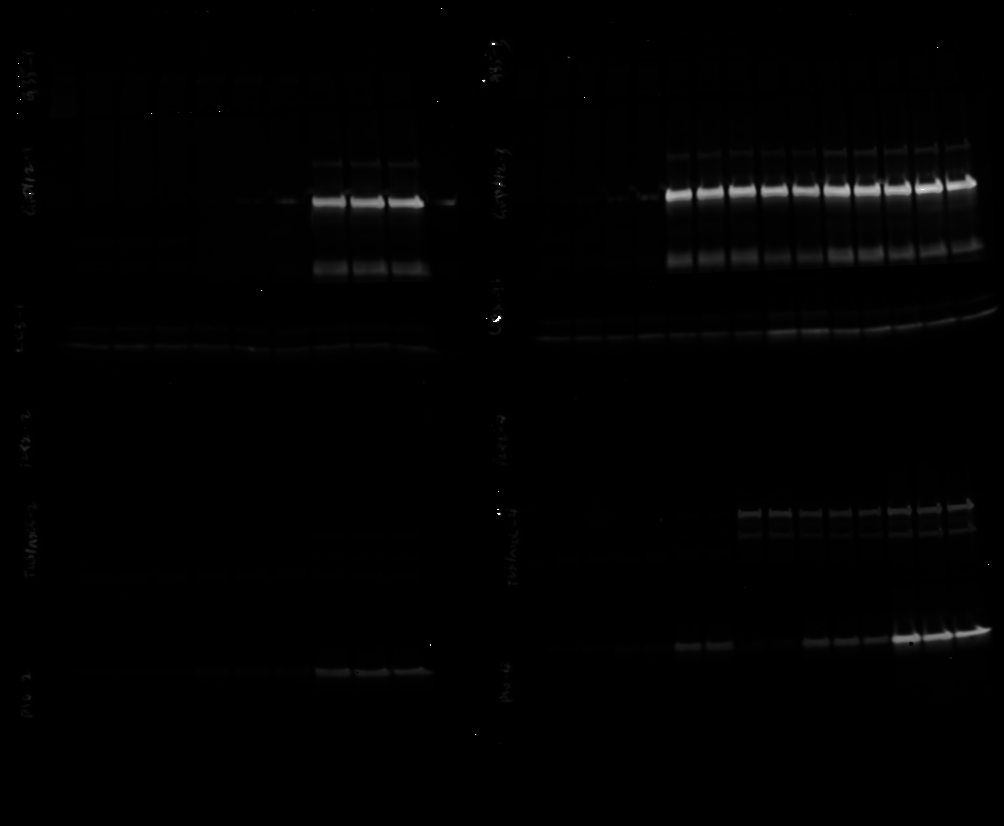

Supplement: Figure 3—source data 1. [file elife-87098-fig3-data1.zip › Figure 3-source data 1/Figure 3A-images/800_3.tif]

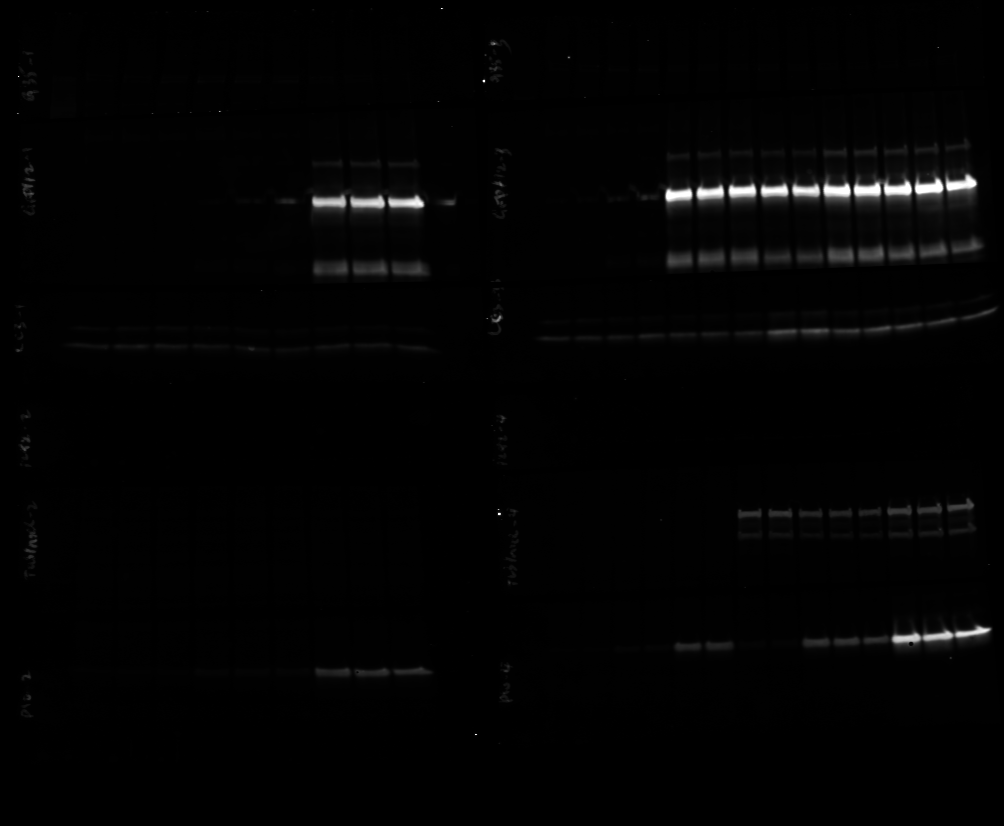

Supplement: Figure 3—source data 1. [file elife-87098-fig3-data1.zip › Figure 3-source data 1/Figure 3A-images/800_1.tif]

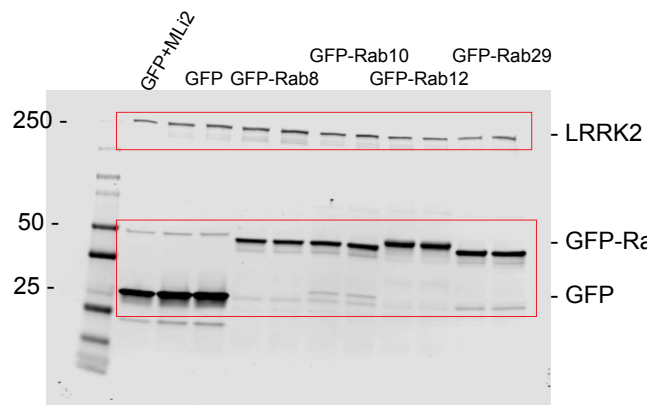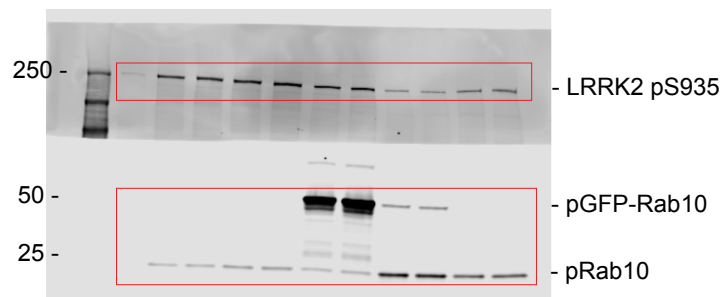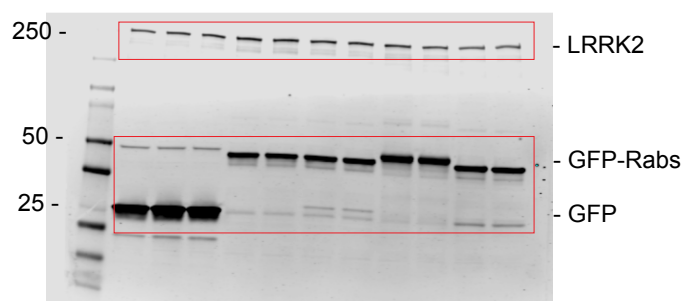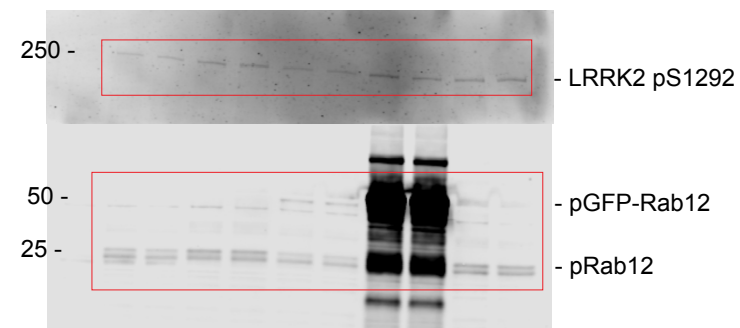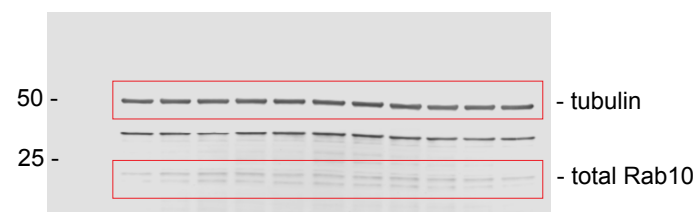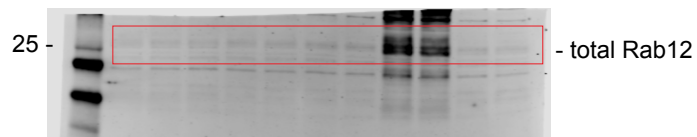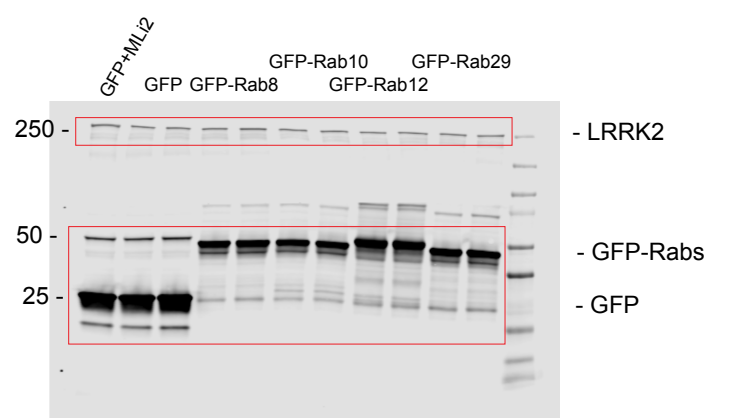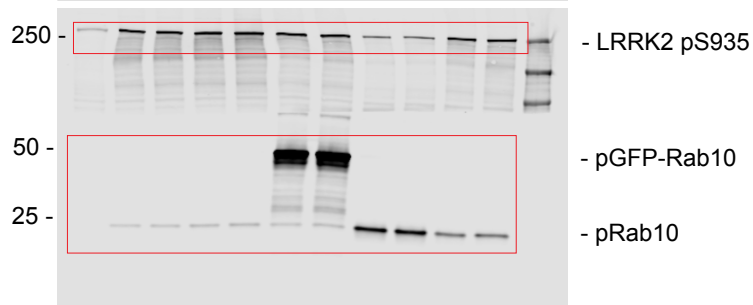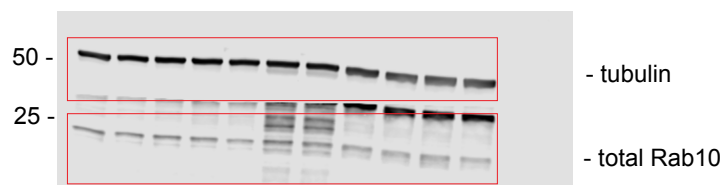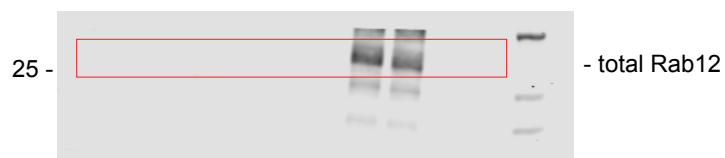

Supplement: Figure 3—source data 1. [file elife-87098-fig3-data1.zip › Figure 3-source data 1/annotated/Supporting material for figure 3C_3D - annotated blots.pdf]

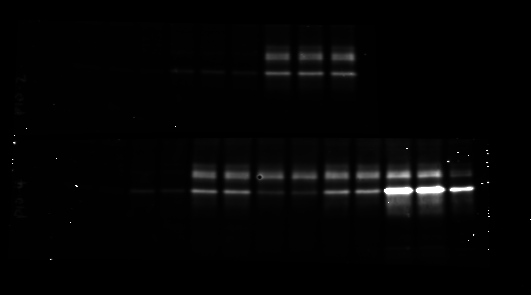

Supplement: Figure 4—source data 1. [file elife-87098-fig4-data1.zip › Figure 4C-source data 1/800_4.tif]

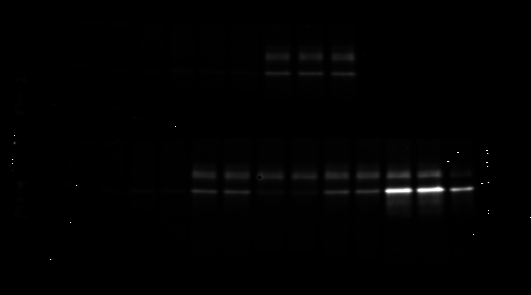

Supplement: Figure 4—source data 1. [file elife-87098-fig4-data1.zip › Figure 4C-source data 1/800_5.tif]

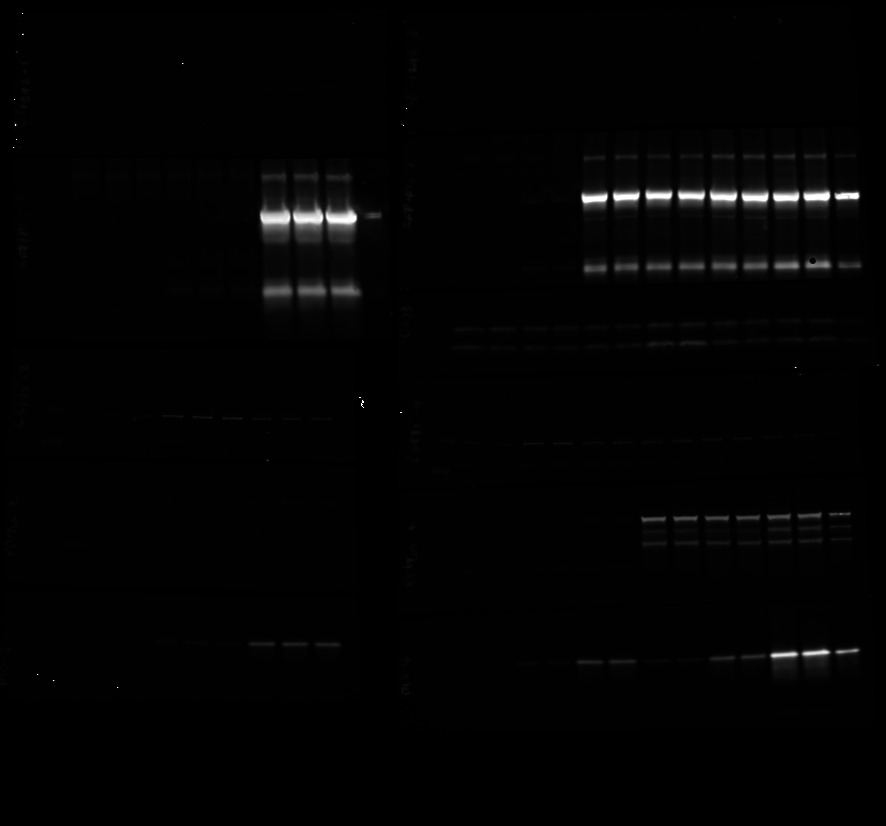

Supplement: Figure 4—source data 1. [file elife-87098-fig4-data1.zip › Figure 4C-source data 1/800_1.tif]

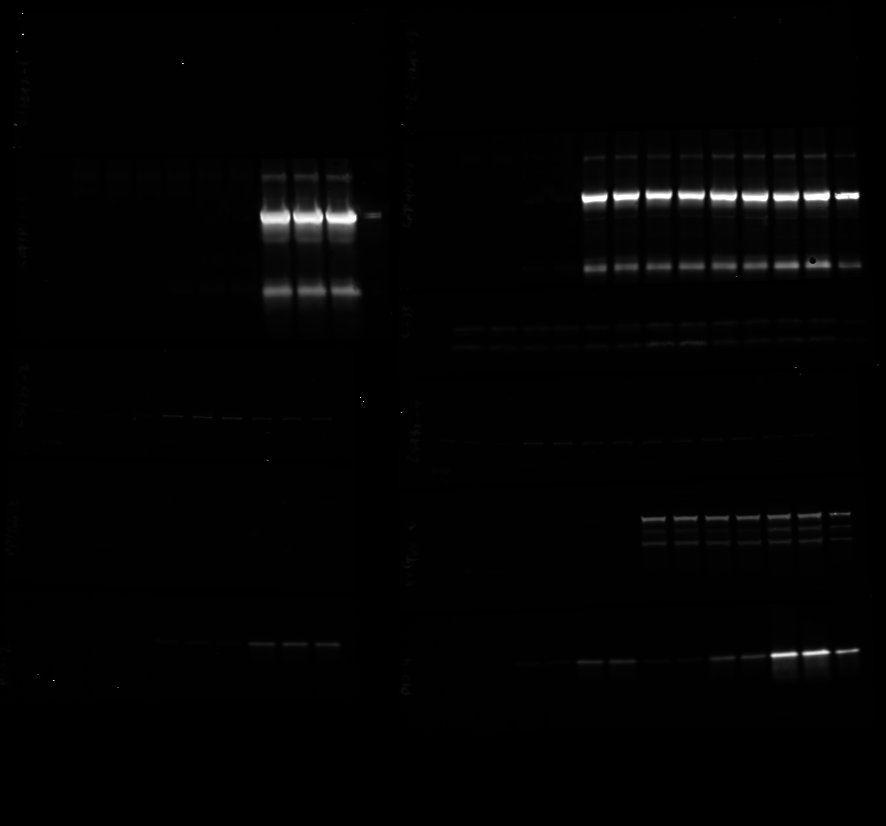

Supplement: Figure 4—source data 1. [file elife-87098-fig4-data1.zip › Figure 4C-source data 1/800_2.tif]

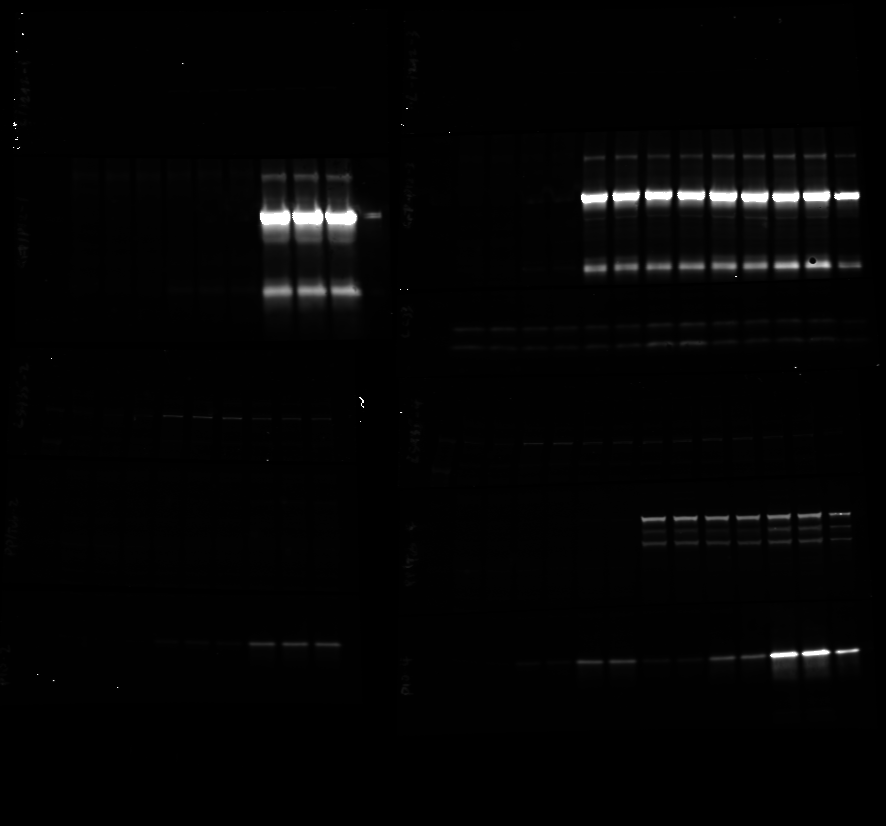

Supplement: Figure 4—source data 1. [file elife-87098-fig4-data1.zip › Figure 4C-source data 1/800_3.tif]

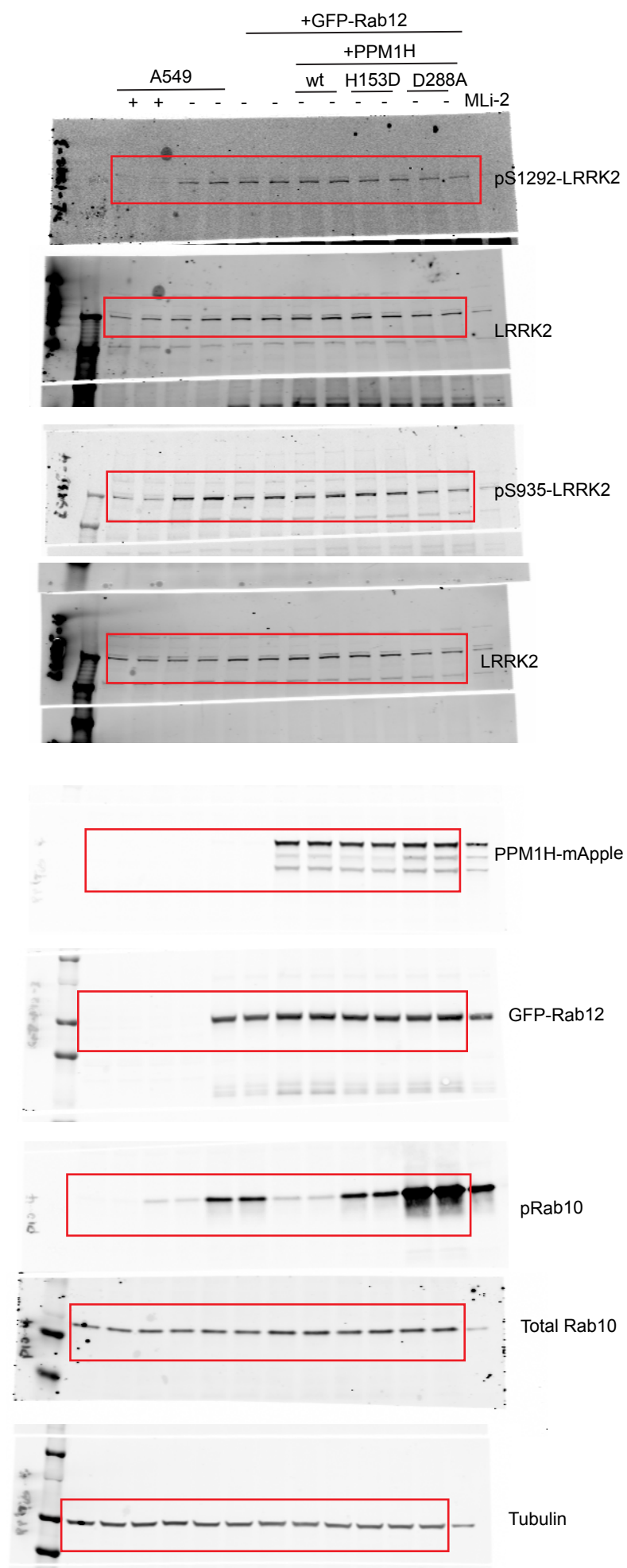

Supplement: Figure 4—source data 1. [file elife-87098-fig4-data1.zip › Figure 4C-source data 1/Supporting material for figure 4C_D_annotated blots.pdf]

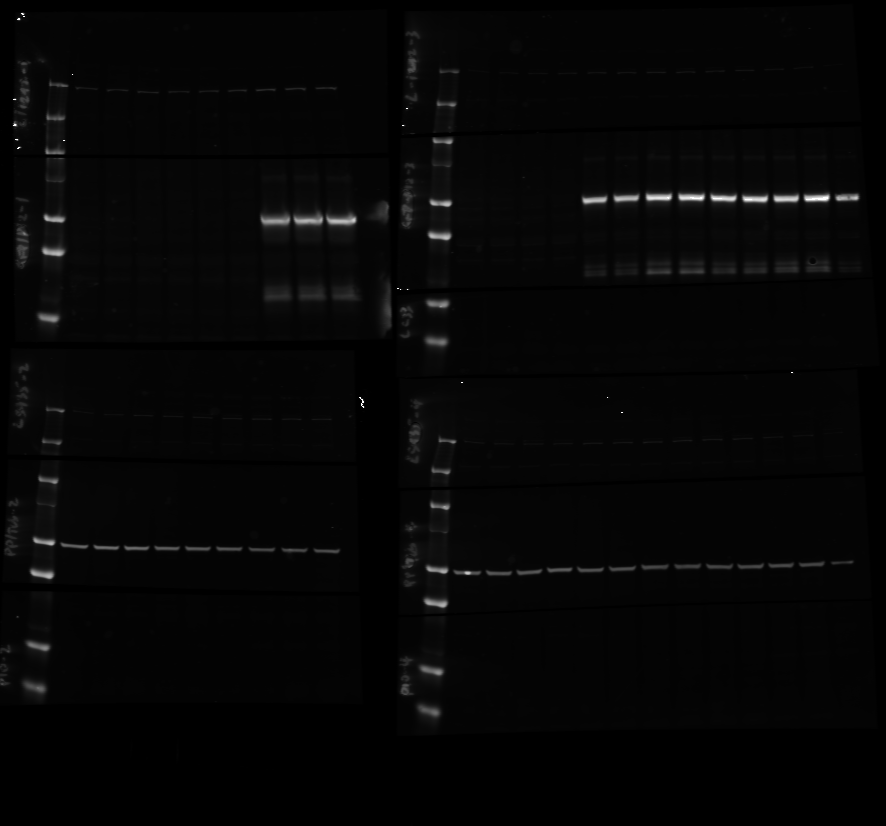

Supplement: Figure 4—source data 1. [file elife-87098-fig4-data1.zip › Figure 4C-source data 1/700_1.tif]

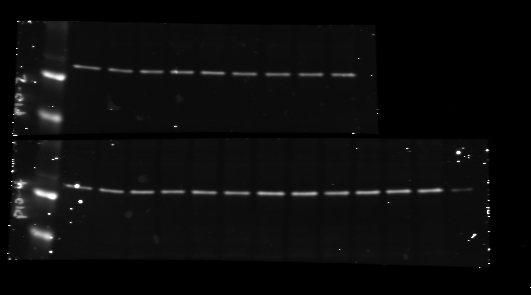

Supplement: Figure 4—source data 1. [file elife-87098-fig4-data1.zip › Figure 4C-source data 1/700_5.tif]

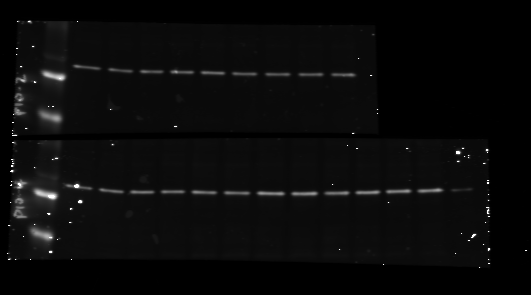

Supplement: Figure 4—source data 1. [file elife-87098-fig4-data1.zip › Figure 4C-source data 1/700_4.tif]

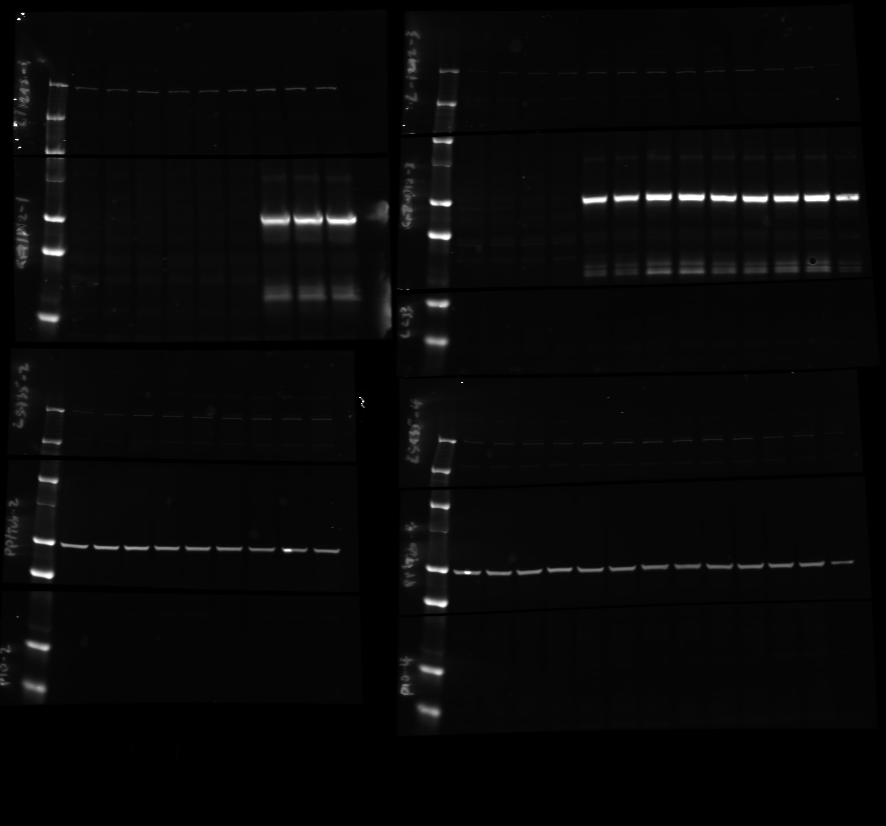

Supplement: Figure 4—source data 1. [file elife-87098-fig4-data1.zip › Figure 4C-source data 1/700-2.tif]

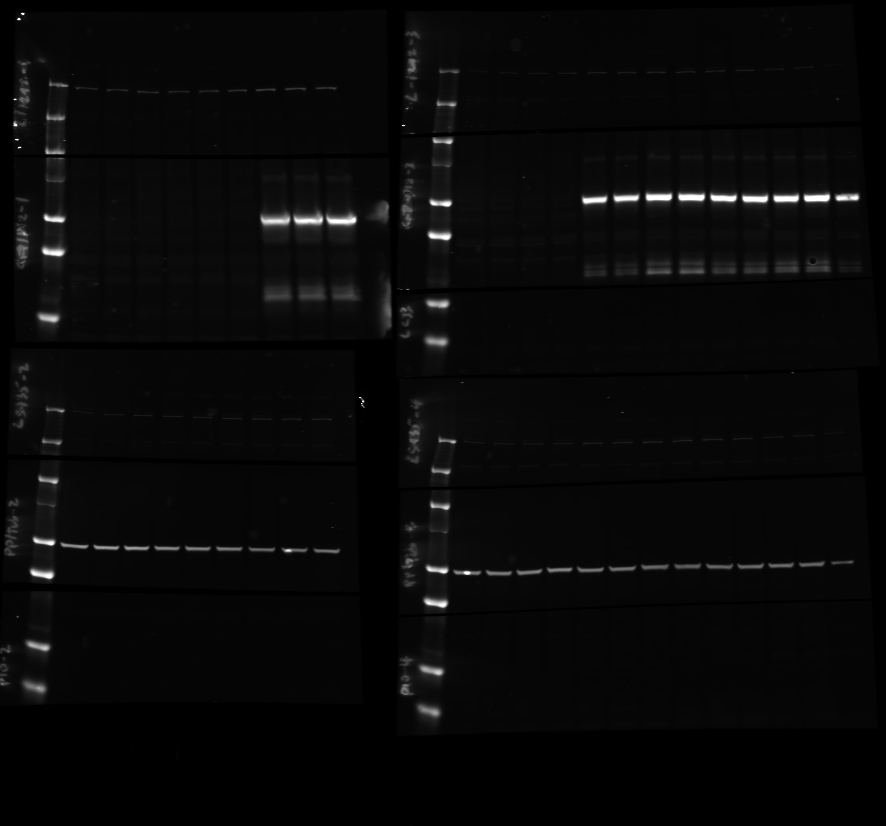

Supplement: Figure 4—source data 1. [file elife-87098-fig4-data1.zip › Figure 4C-source data 1/700-3.tif]

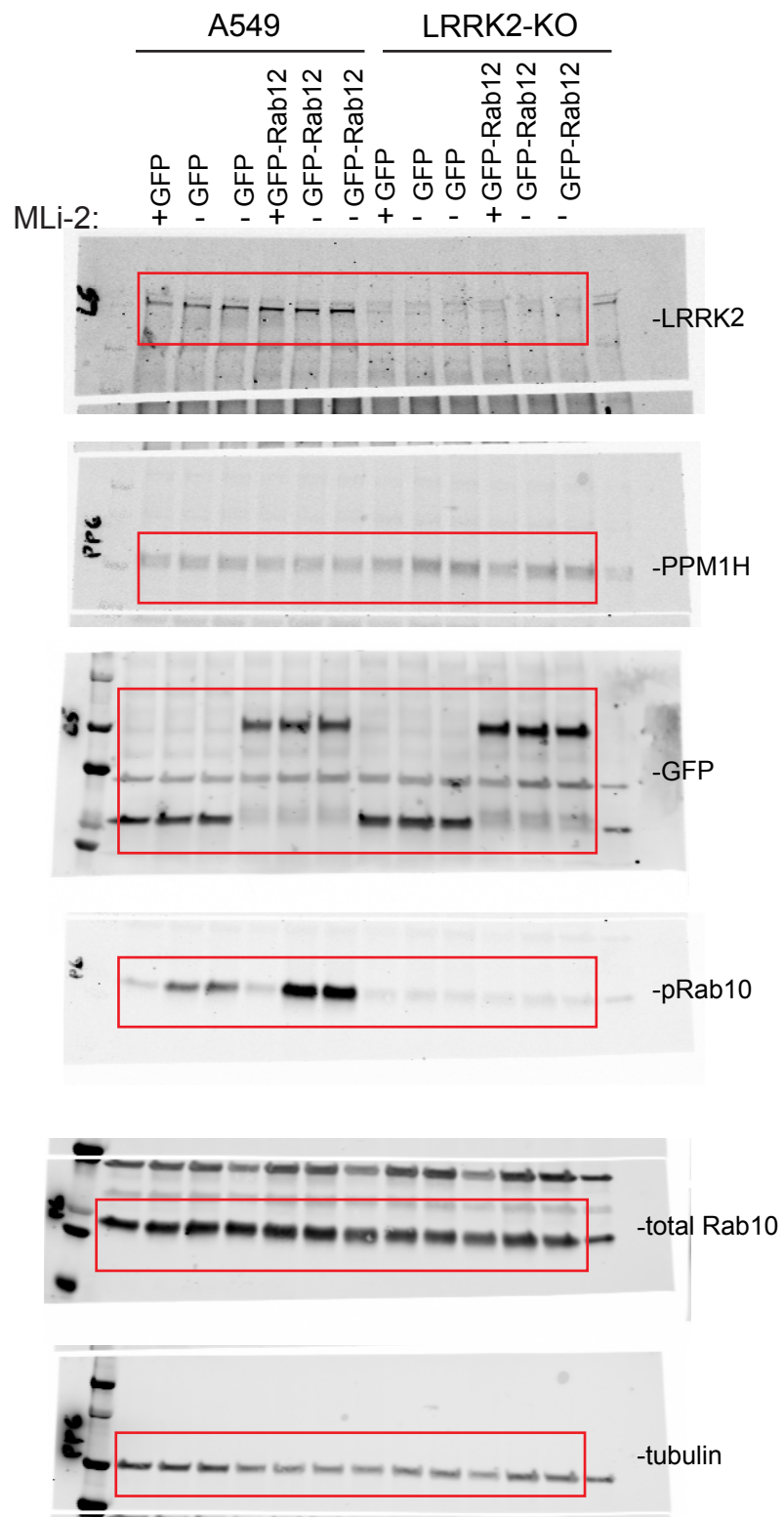

Supplement: Figure 5—source data 1. [file elife-87098-fig5-data1.zip › Figure 5-source data 1/Supporting material for figure 5A_B_annotated blots.pdf]

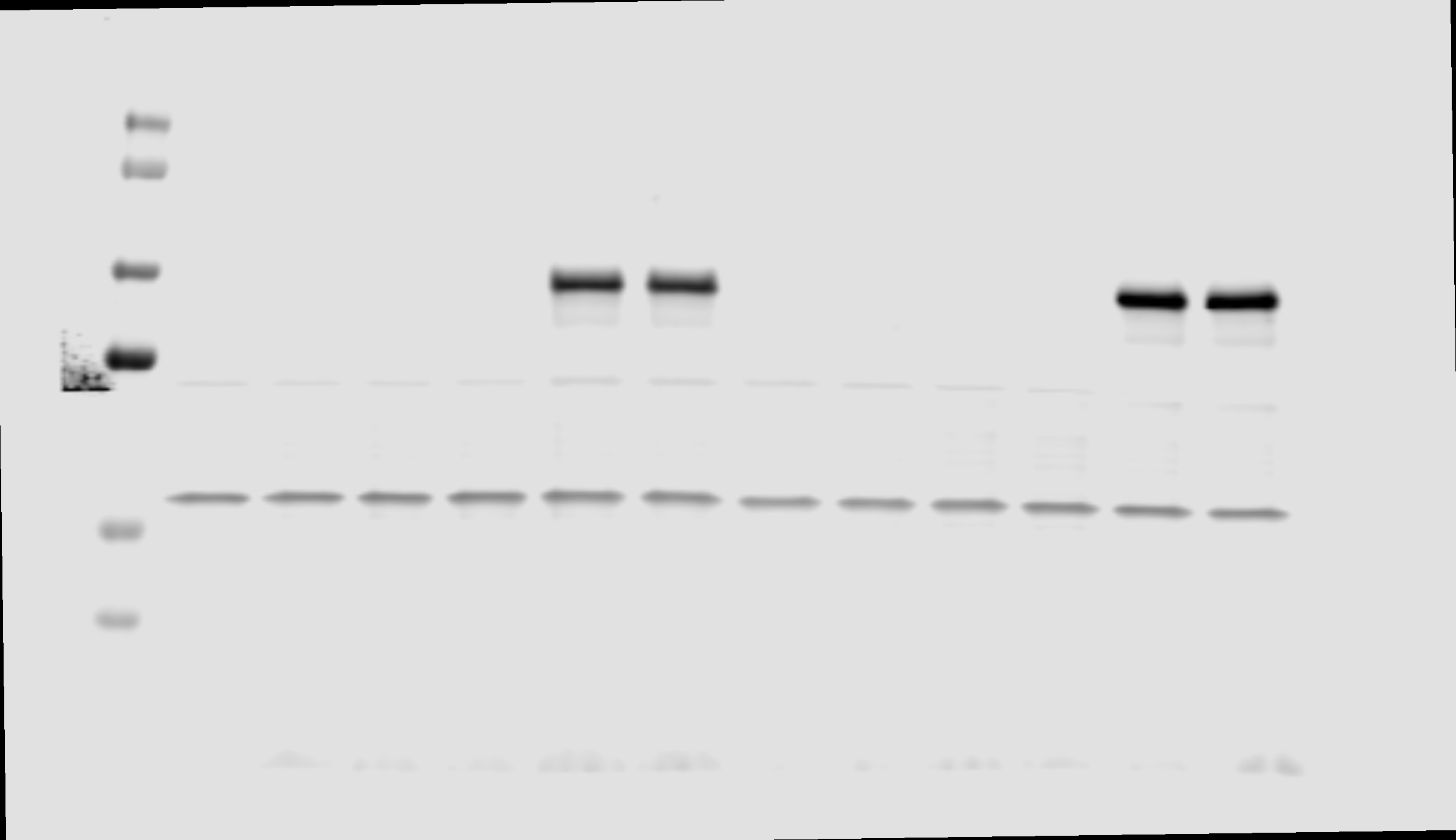

Supplement: Figure 5—source data 1. [file elife-87098-fig5-data1.zip › Figure 5-source data 1/Figure 5C-source data 1/4_680.tif]

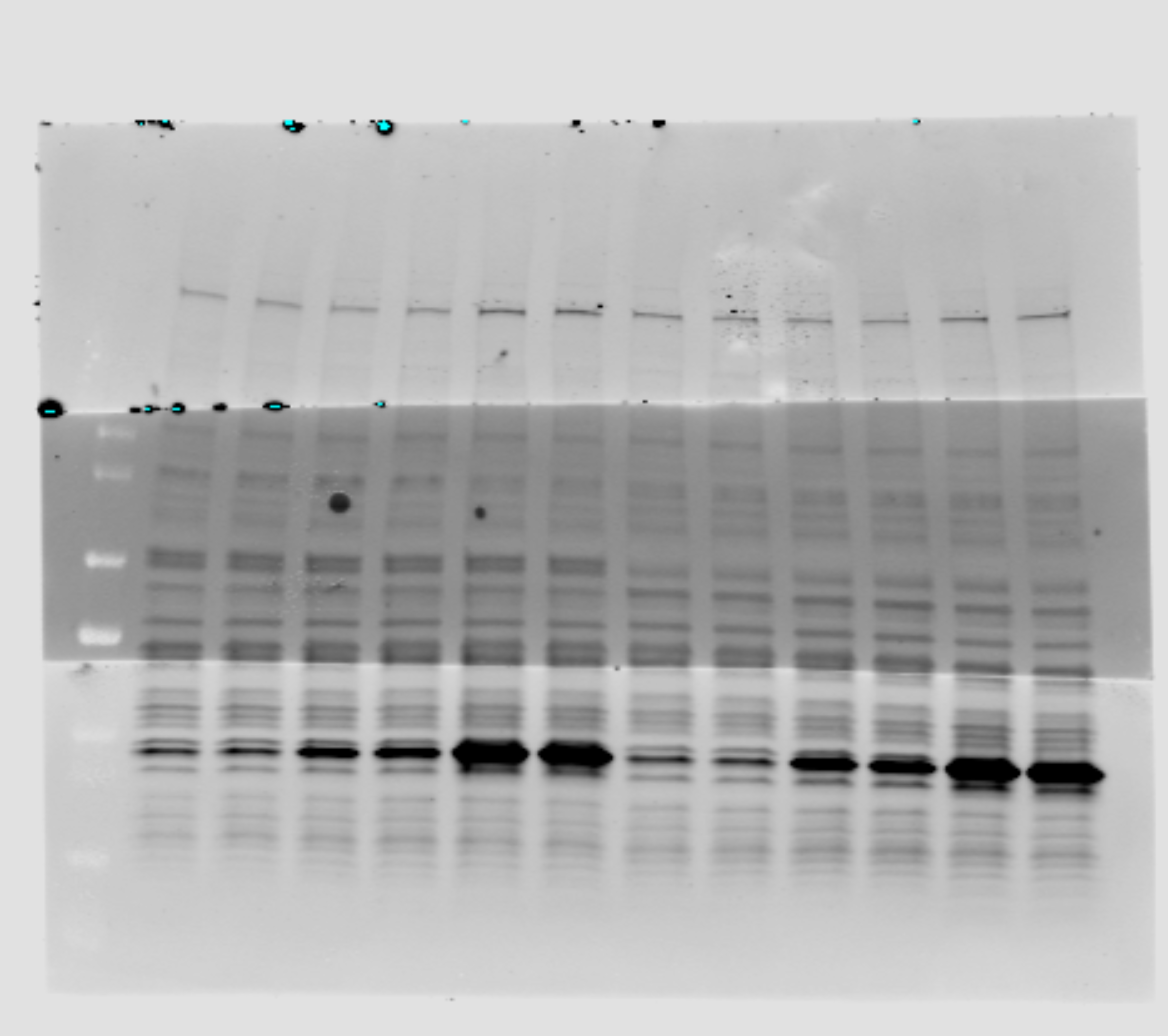

Supplement: Figure 5—source data 1. [file elife-87098-fig5-data1.zip › Figure 5-source data 1/Figure 5C-source data 1/4_800.tif]

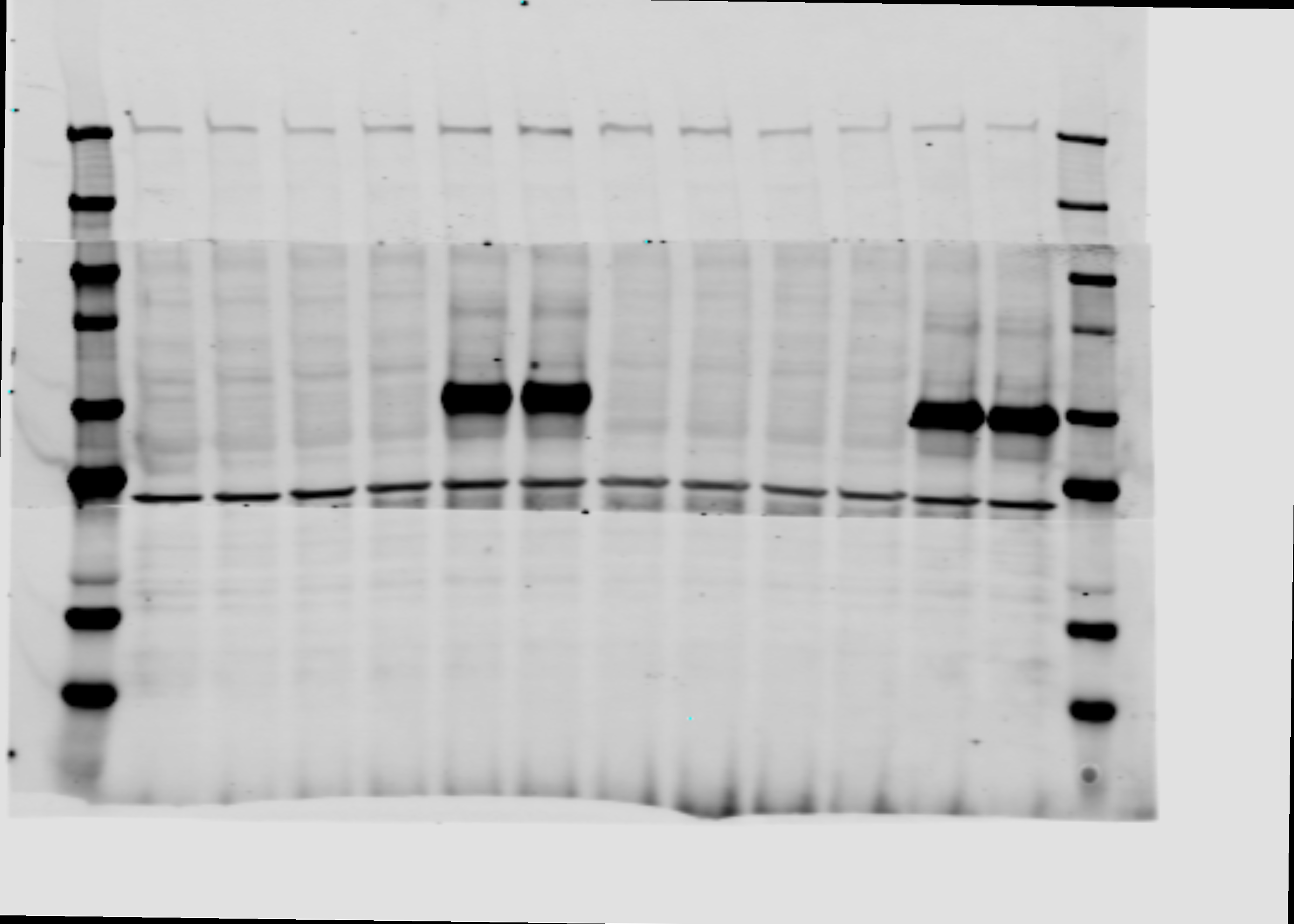

Supplement: Figure 5—source data 1. [file elife-87098-fig5-data1.zip › Figure 5-source data 1/Figure 5C-source data 1/1_680_dark.tif]

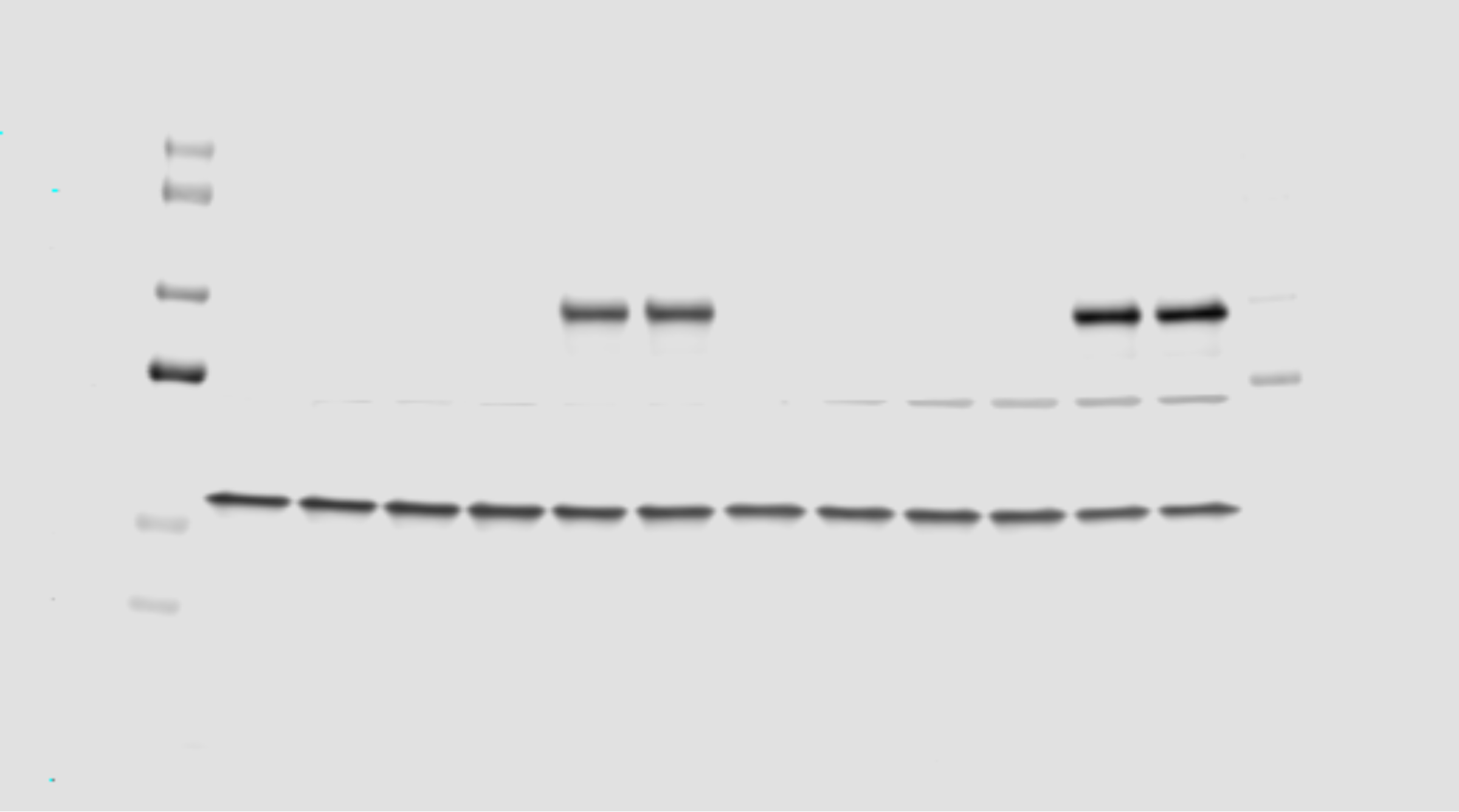

Supplement: Figure 5—source data 1. [file elife-87098-fig5-data1.zip › Figure 5-source data 1/Figure 5C-source data 1/2_680.tif]

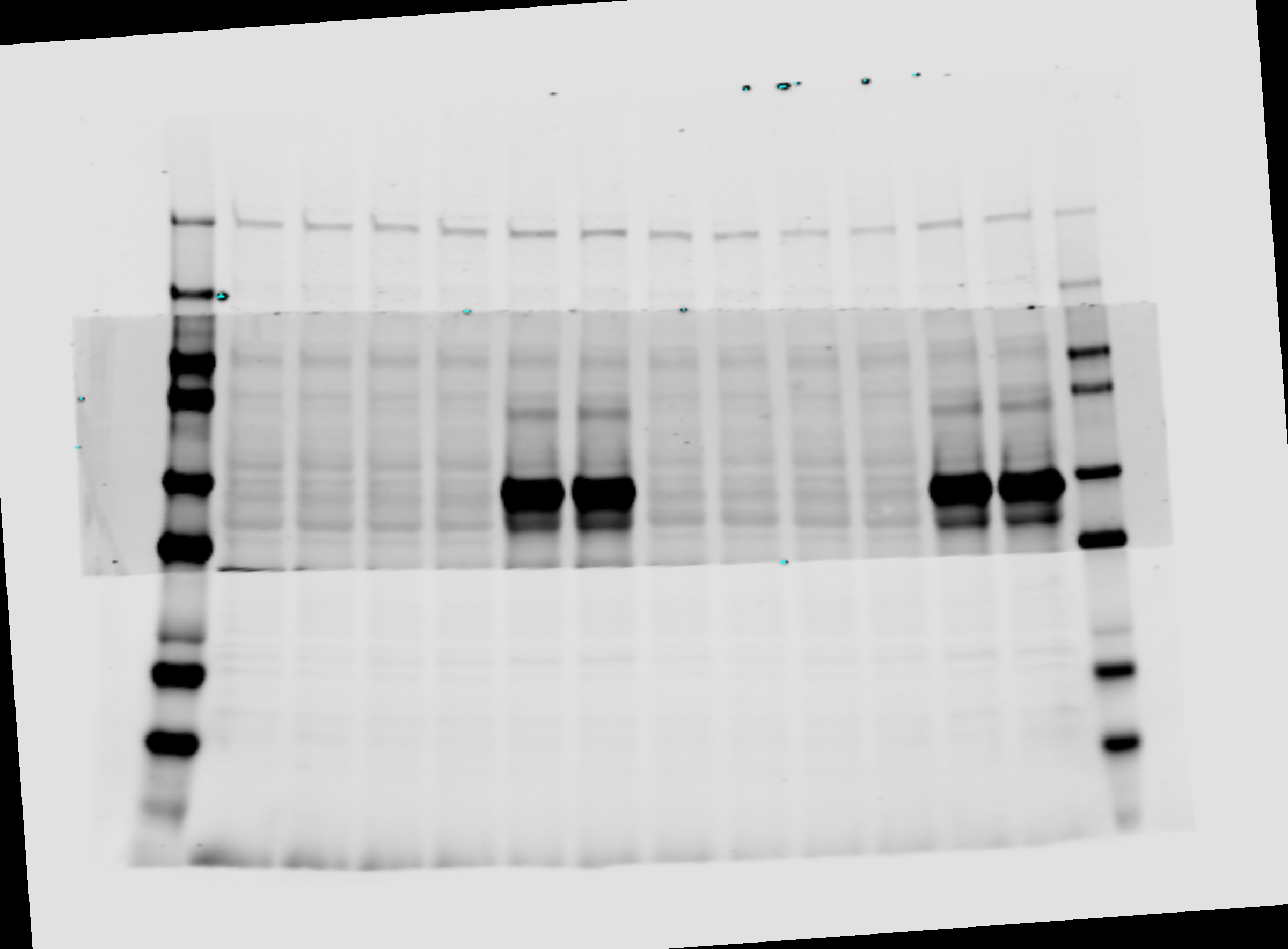

Supplement: Figure 5—source data 1. [file elife-87098-fig5-data1.zip › Figure 5-source data 1/Figure 5C-source data 1/2_680_dark.tif]

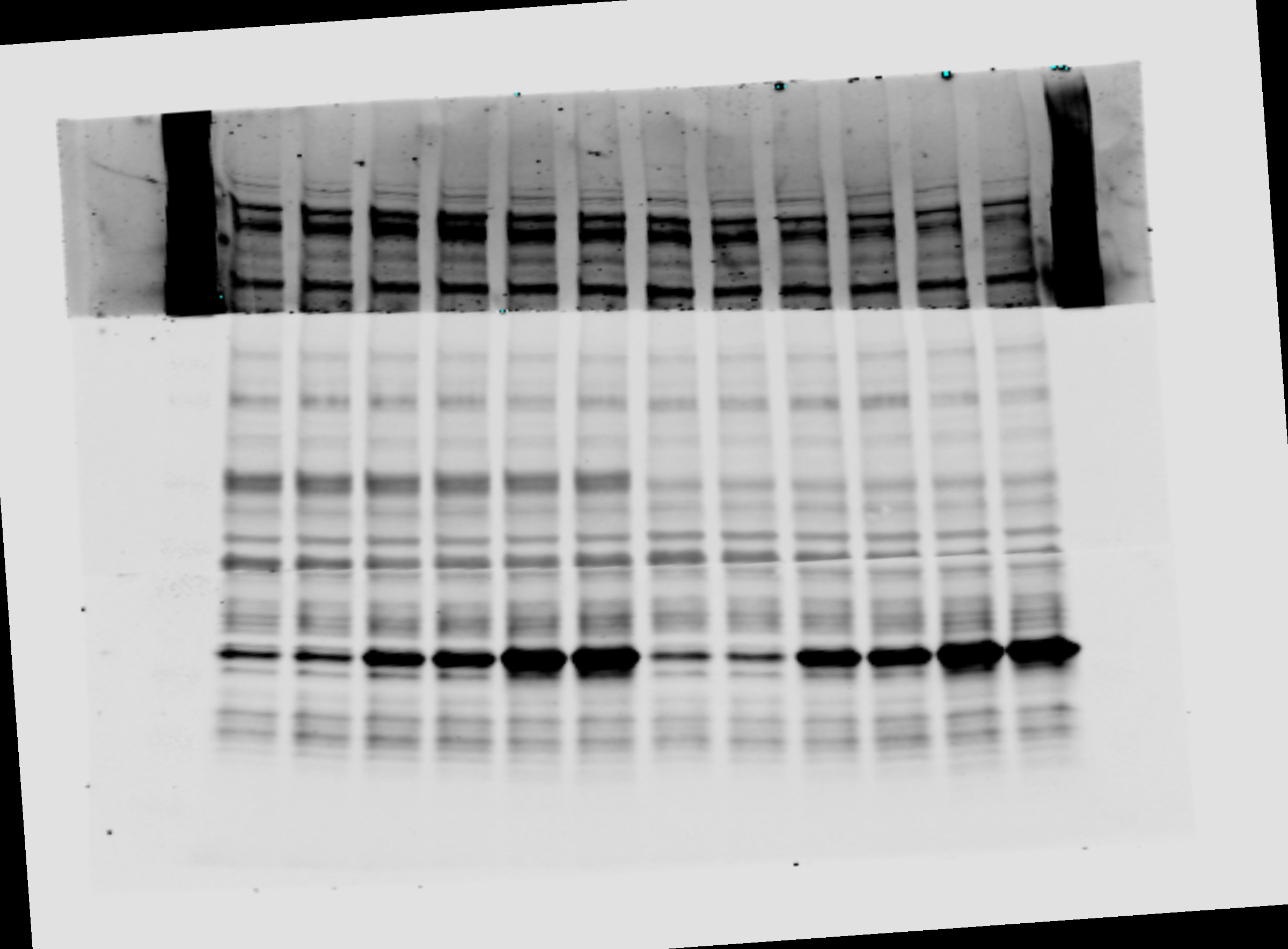

Supplement: Figure 5—source data 1. [file elife-87098-fig5-data1.zip › Figure 5-source data 1/Figure 5C-source data 1/2_800_dark.tif]

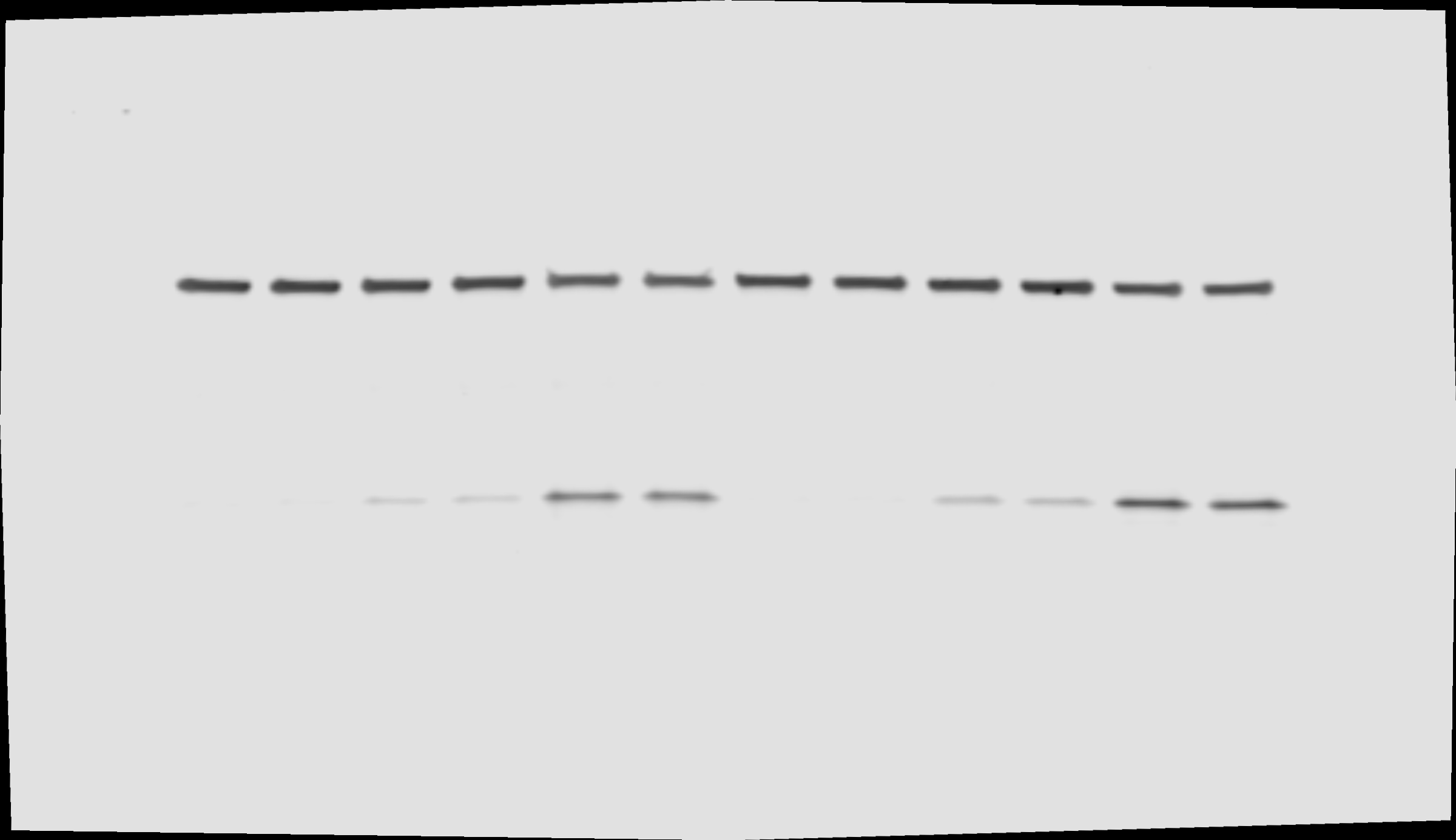

Supplement: Figure 5—source data 1. [file elife-87098-fig5-data1.zip › Figure 5-source data 1/Figure 5C-source data 1/4_800_dark.tif]

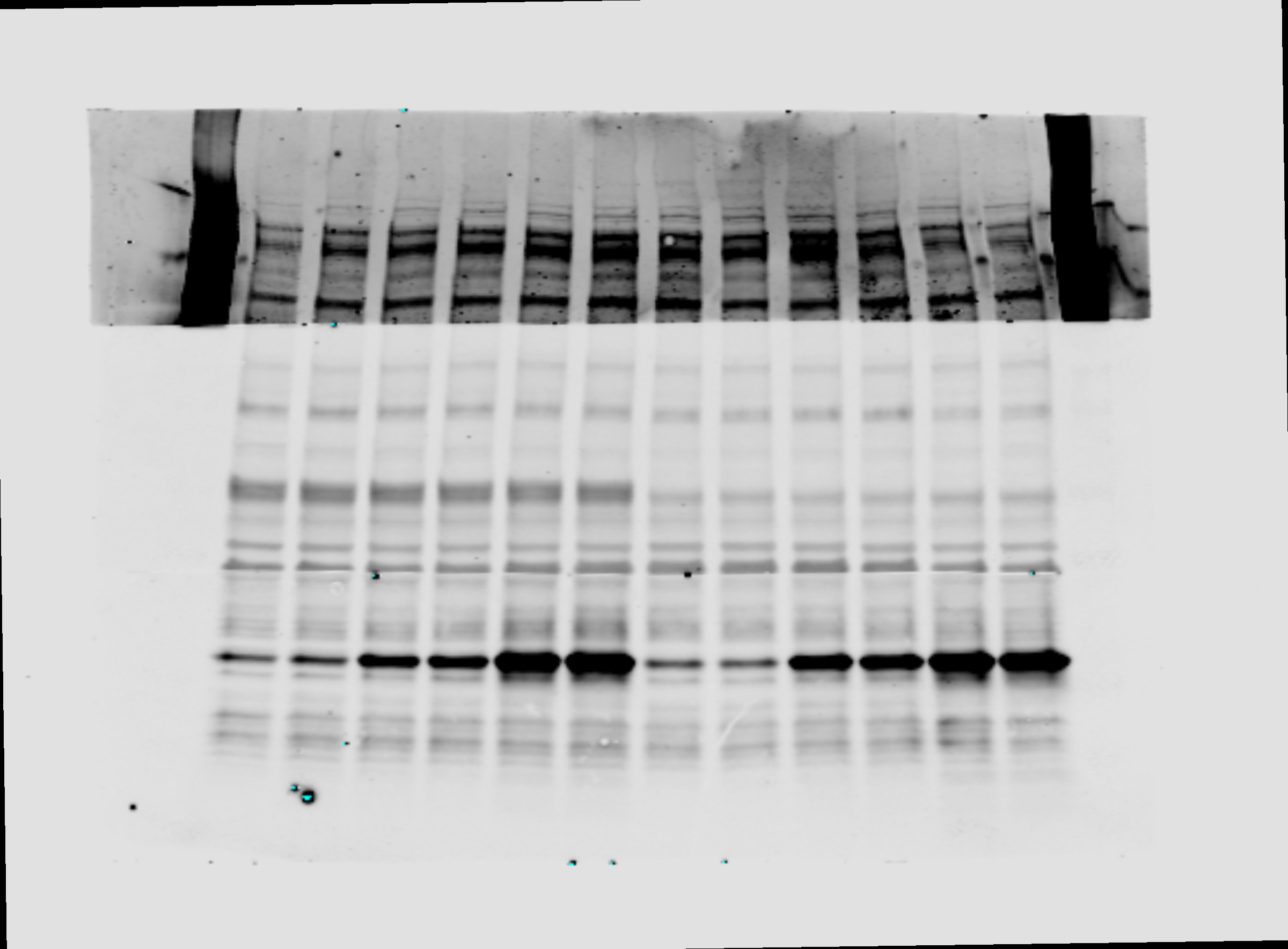

Supplement: Figure 5—source data 1. [file elife-87098-fig5-data1.zip › Figure 5-source data 1/Figure 5C-source data 1/3_800_dark.tif]

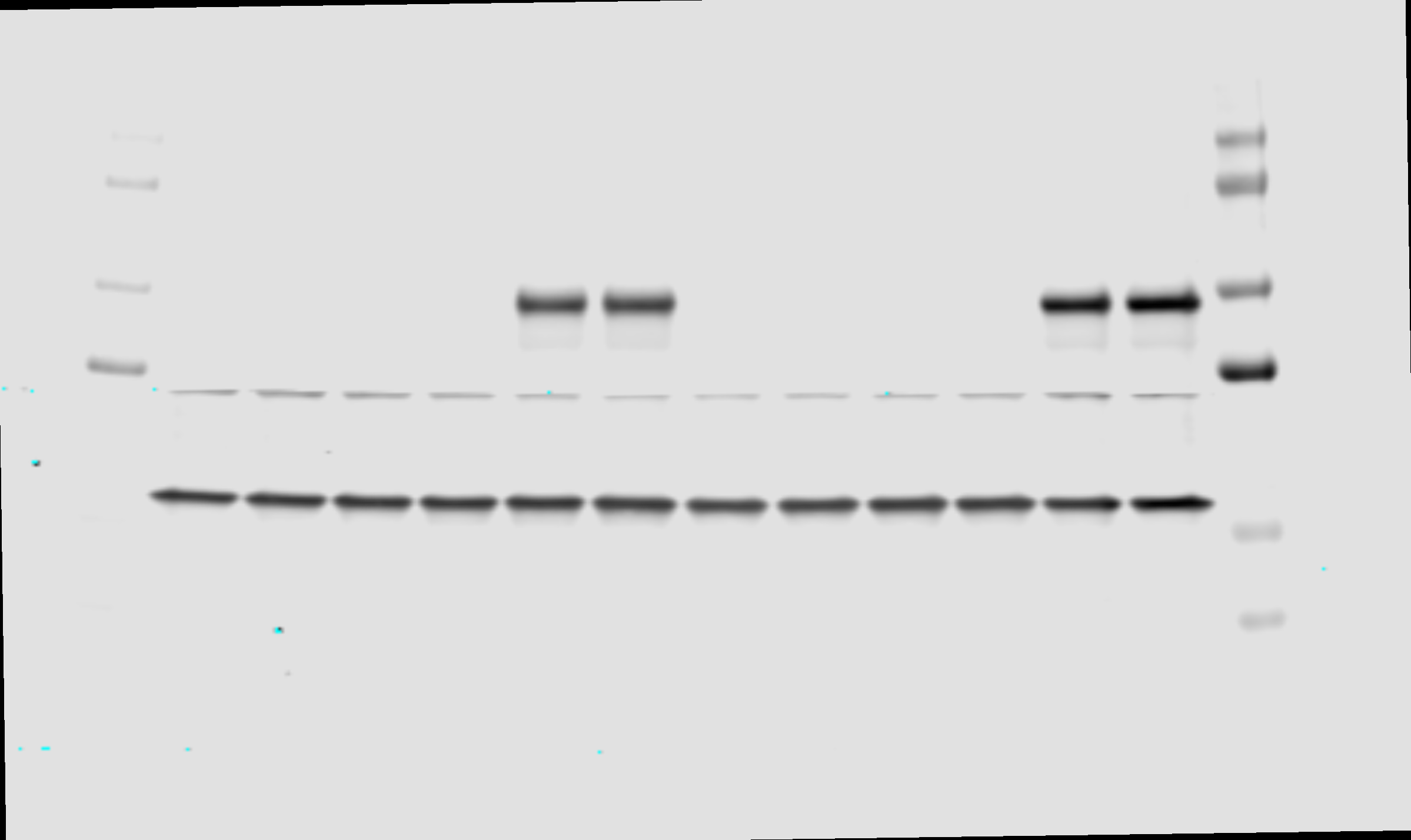

Supplement: Figure 5—source data 1. [file elife-87098-fig5-data1.zip › Figure 5-source data 1/Figure 5C-source data 1/3_680.tif]

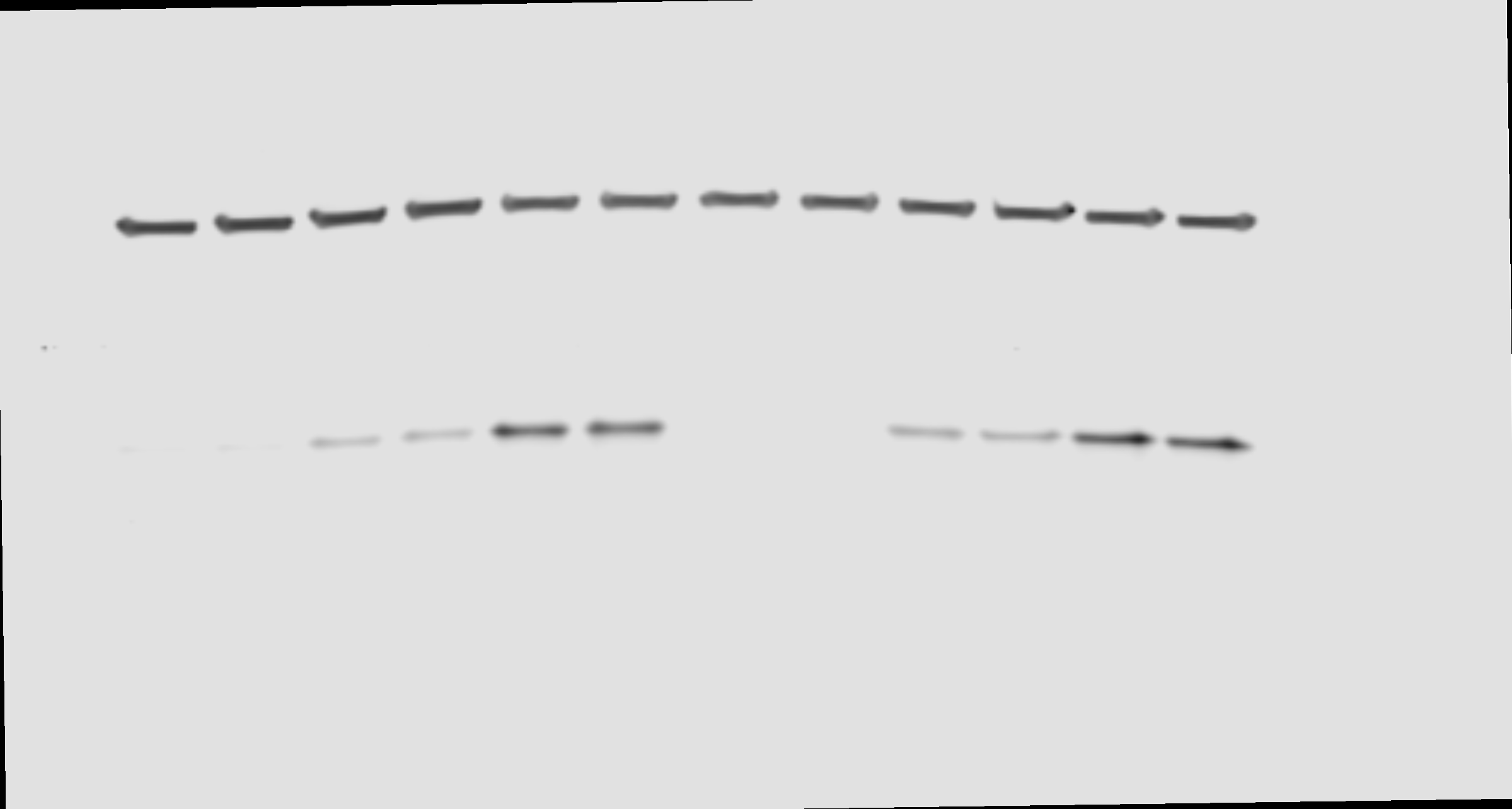

Supplement: Figure 5—source data 1. [file elife-87098-fig5-data1.zip › Figure 5-source data 1/Figure 5C-source data 1/1_800.tif]

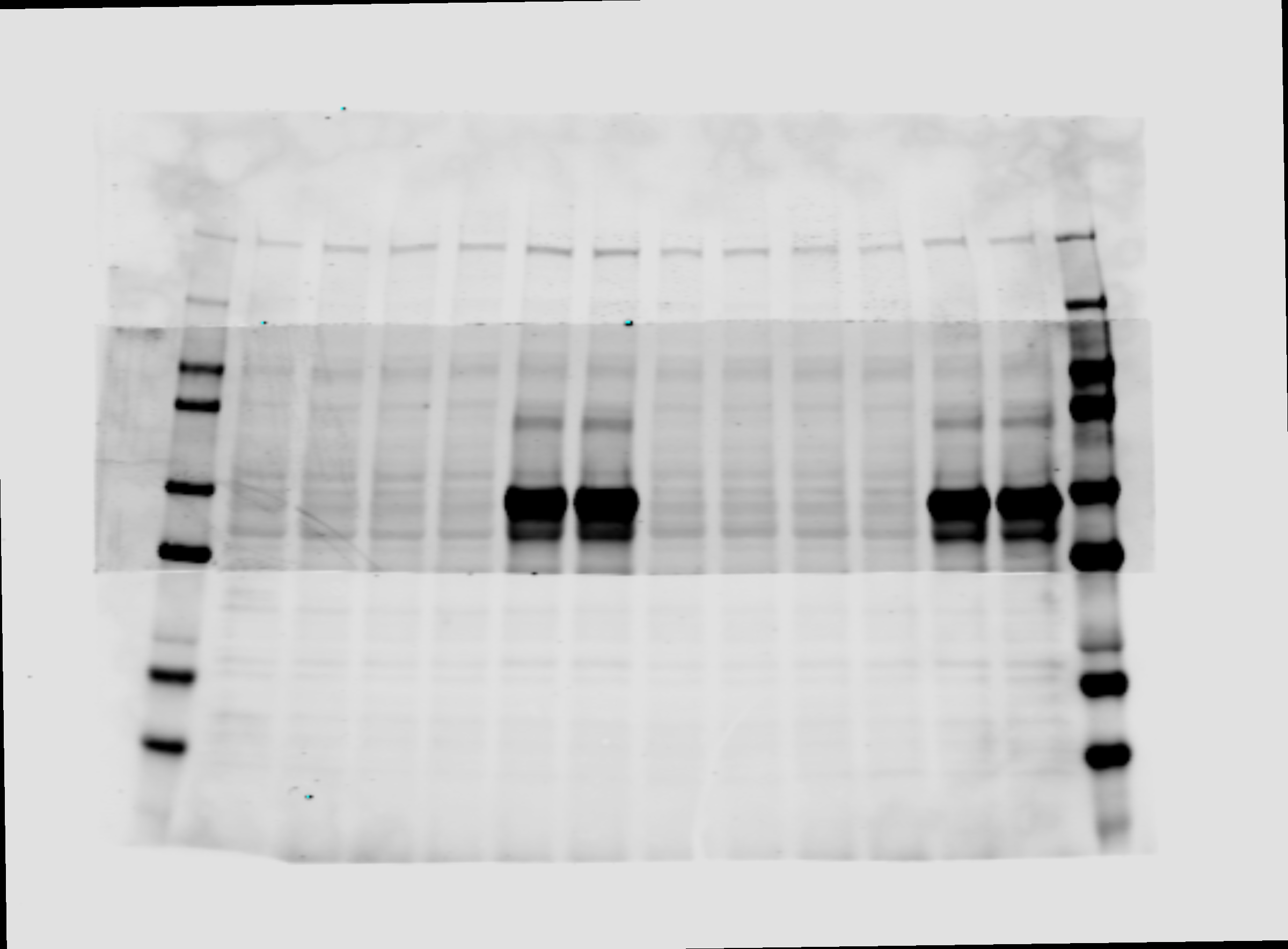

Supplement: Figure 5—source data 1. [file elife-87098-fig5-data1.zip › Figure 5-source data 1/Figure 5C-source data 1/3_680_dark.tif]

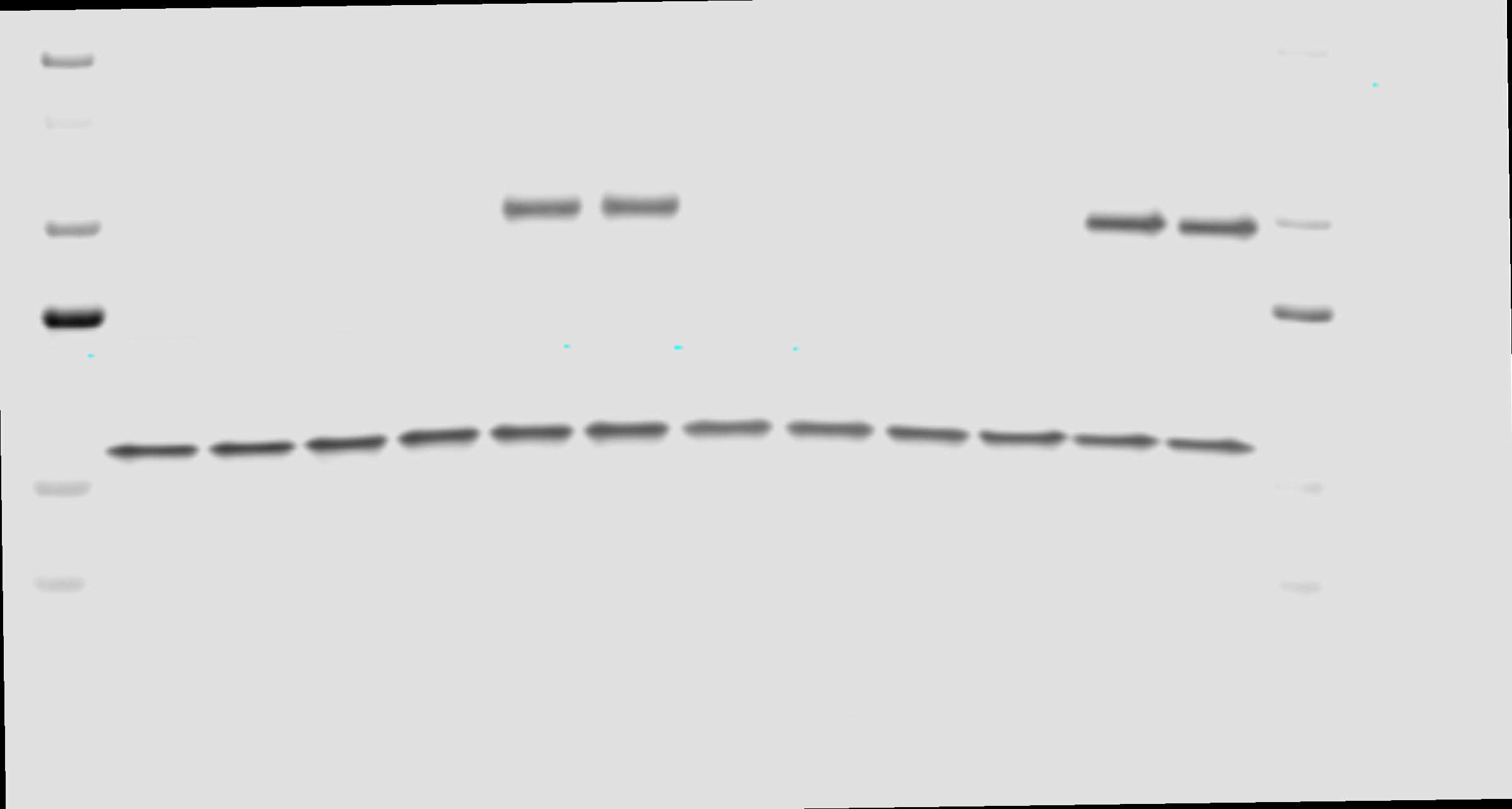

Supplement: Figure 5—source data 1. [file elife-87098-fig5-data1.zip › Figure 5-source data 1/Figure 5C-source data 1/1_680.tif]

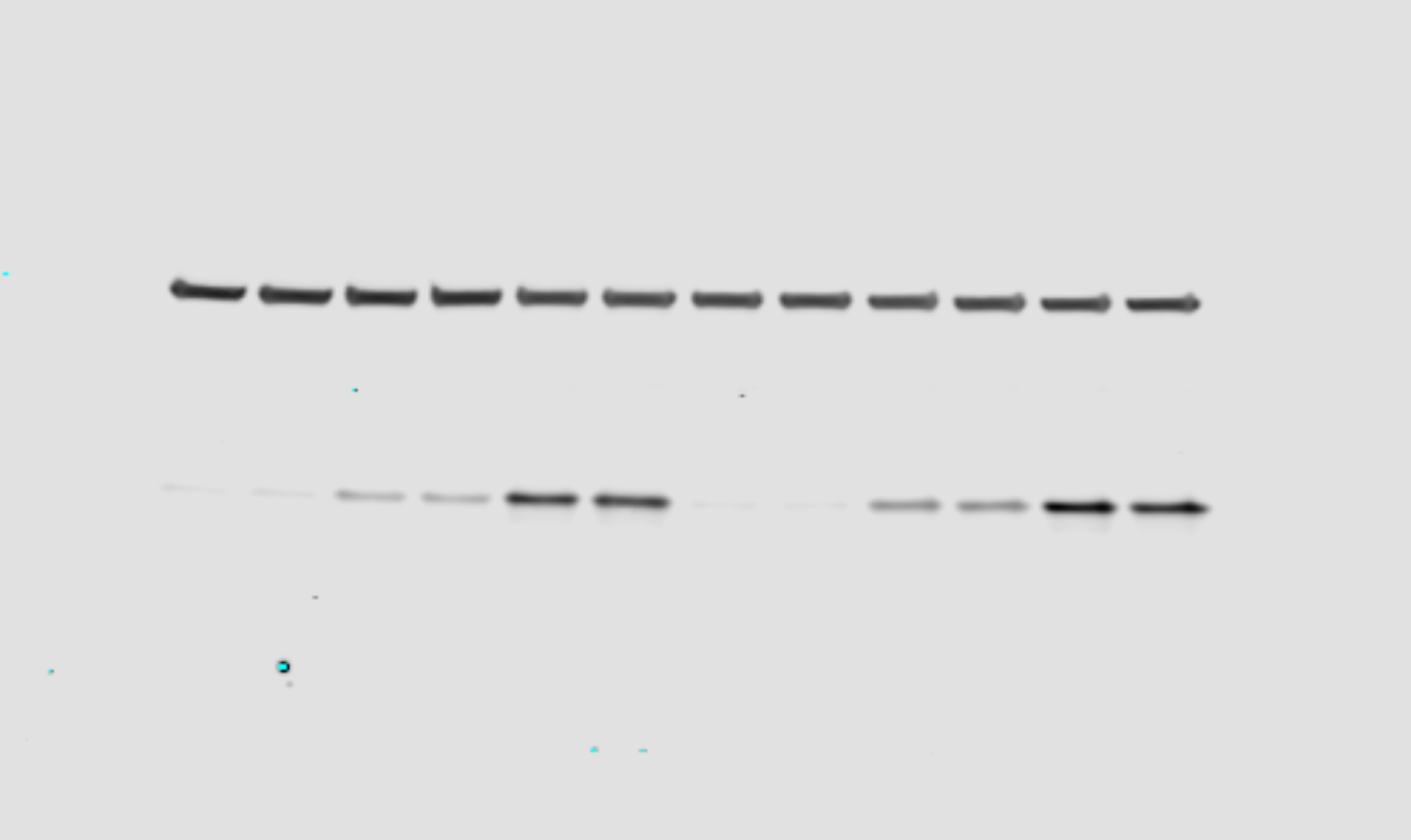

Supplement: Figure 5—source data 1. [file elife-87098-fig5-data1.zip › Figure 5-source data 1/Figure 5C-source data 1/3_800.tif]

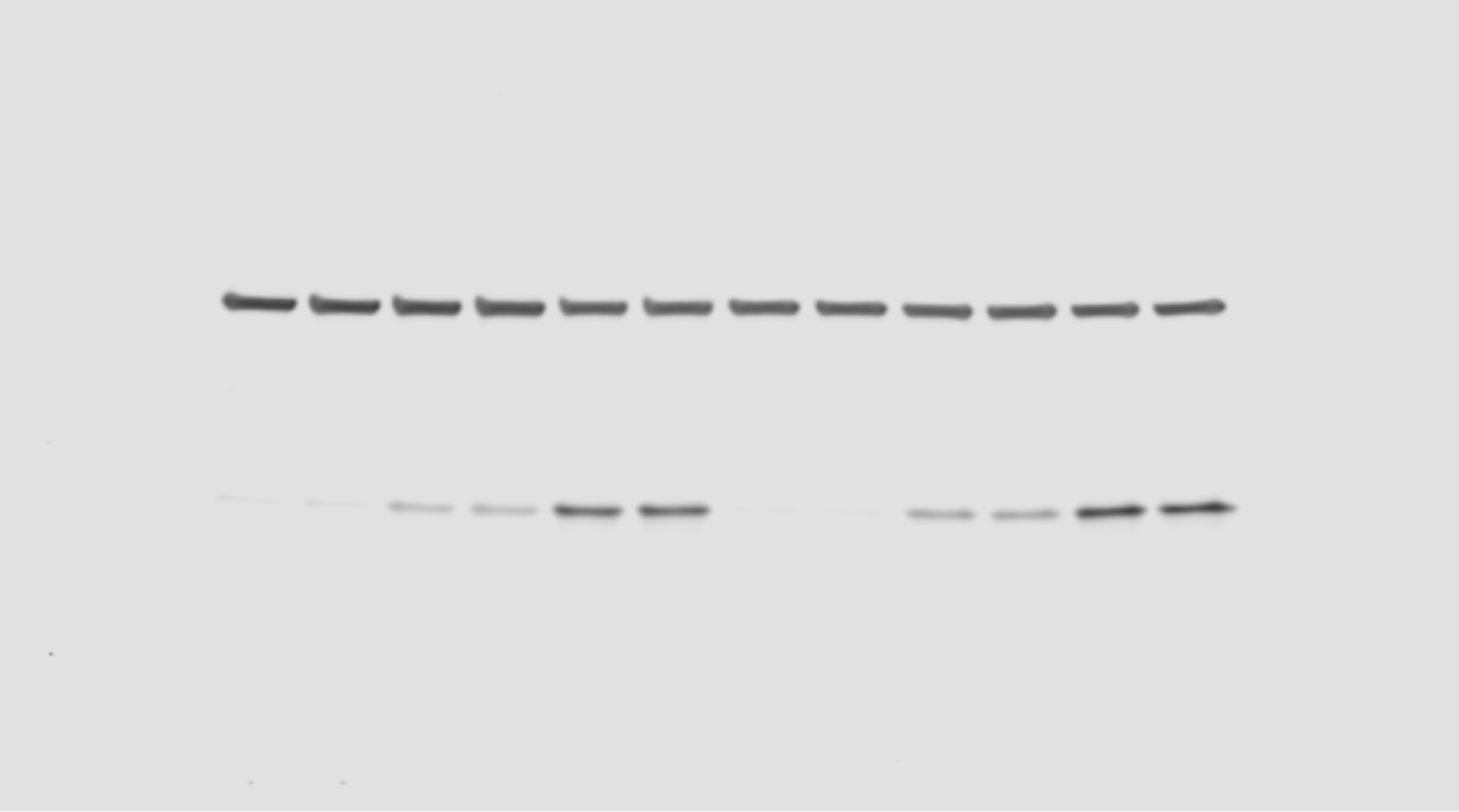

Supplement: Figure 5—source data 1. [file elife-87098-fig5-data1.zip › Figure 5-source data 1/Figure 5C-source data 1/2_800.tif]
