# Supplementary material for: Genome-wide screen reveals Rab12 GTPase as a critical activator of Parkinson’s disease-linked LRRK2 kinase
Source: eLife. 2023 Oct 24;12:e87098. doi: 10.7554/eLife.87098 (PMC10708890; doi:10.7554/eLife.87098)
Supplement: Figure 3—source data 1. [file elife-87098-fig3-data1.zip › Figure 3-source data 1/annotated/Supporting material for figure 3E_3F - annotated blots.pdf]

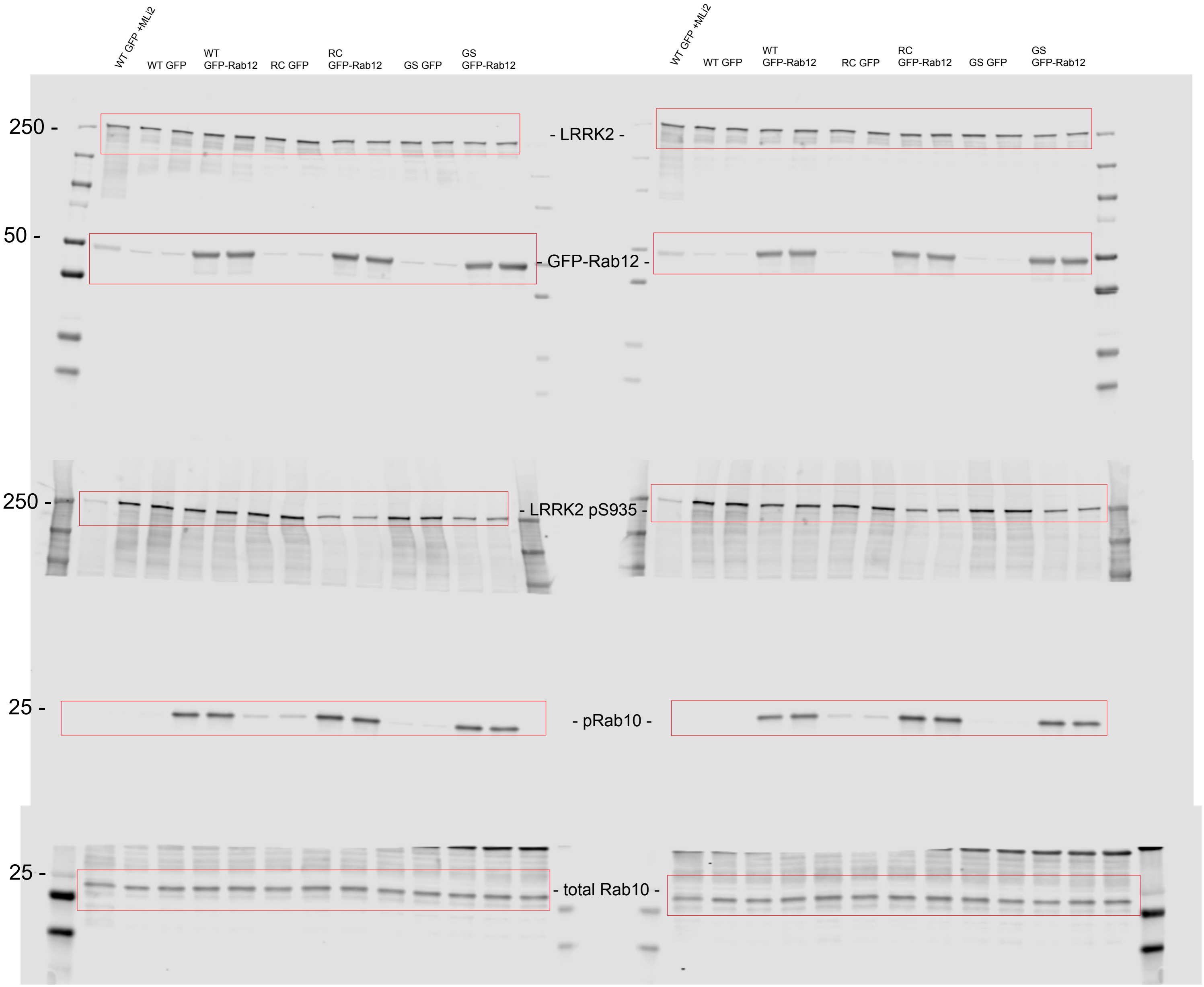

WT GFP +MLi2
WT GFP
WT GFP-Rab12
RC GFP
RC GFP-Rab12
GS GFP
GS GFP-Rab12
250 -
50 -
- LRRK2 -
- GFP-Rab12 -
250 -
- LRRK2 pS935 -
25 -
- pRab10 -
25 -
- total Rab10 -

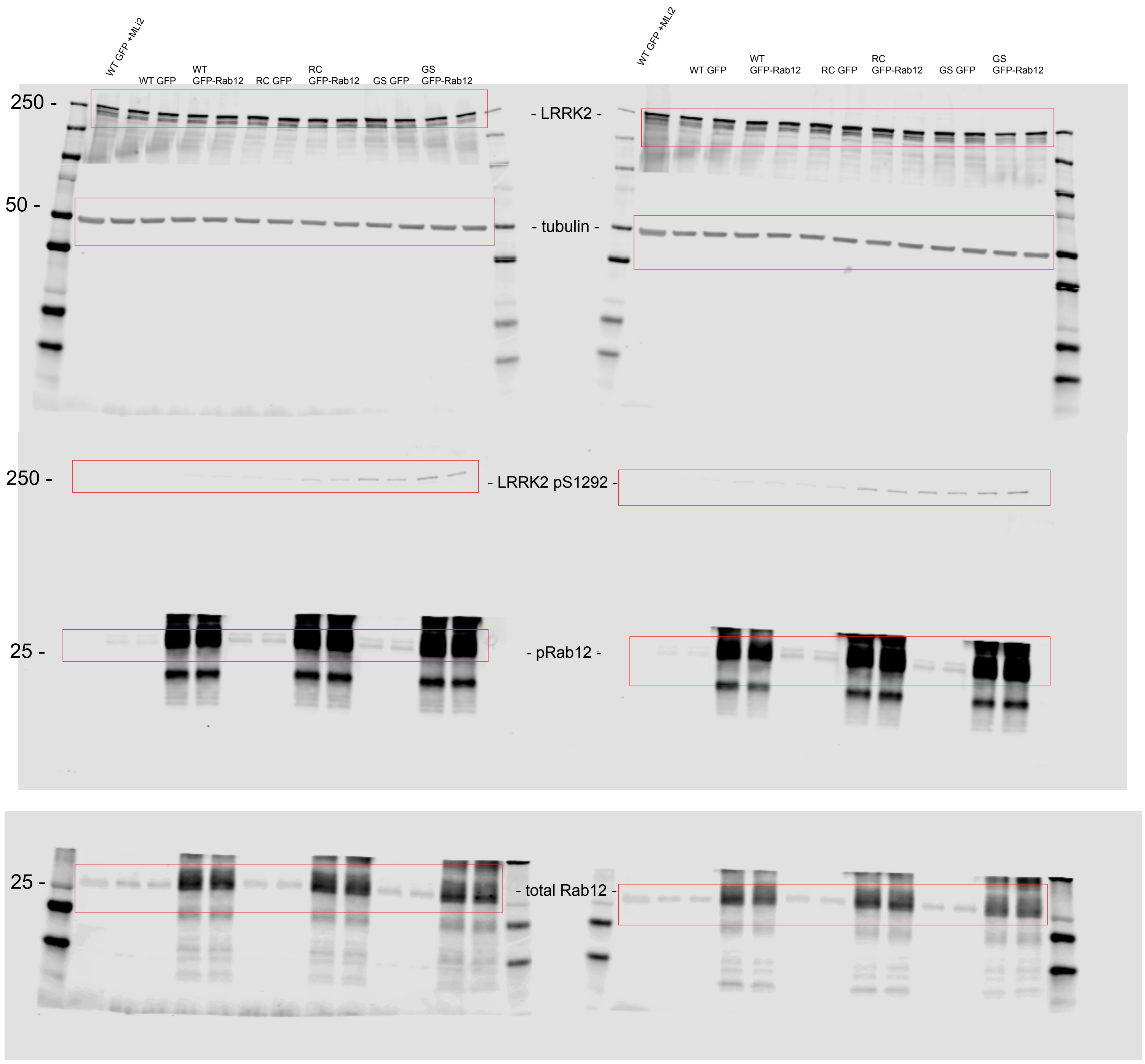
WT GFP +MLi2
WT GFP
WT GFP-Rab12
RC GFP
RC GFP-Rab12
GS GFP
GS GFP-Rab12
WT GFP +MLi2
WT GFP
WT GFP-Rab12
RC GFP
RC GFP-Rab12
GS GFP
GS GFP-Rab12
250 -
50 -
- LRRK2 -
- tubulin -
250 -
- LRRK2 pS1292 -
25 -
- pRab12 -
25 -
- total Rab12 -
