# Supplementary material for: Genome-wide screen reveals Rab12 GTPase as a critical activator of Parkinson’s disease-linked LRRK2 kinase
Source: eLife. 2023 Oct 24;12:e87098. doi: 10.7554/eLife.87098 (PMC10708890; doi:10.7554/eLife.87098)
Supplement: Figure 5—source data 1. [file elife-87098-fig5-data1.zip › Figure 5-source data 1/Supporting material for figure 5C_5D - annotated blots.pdf]

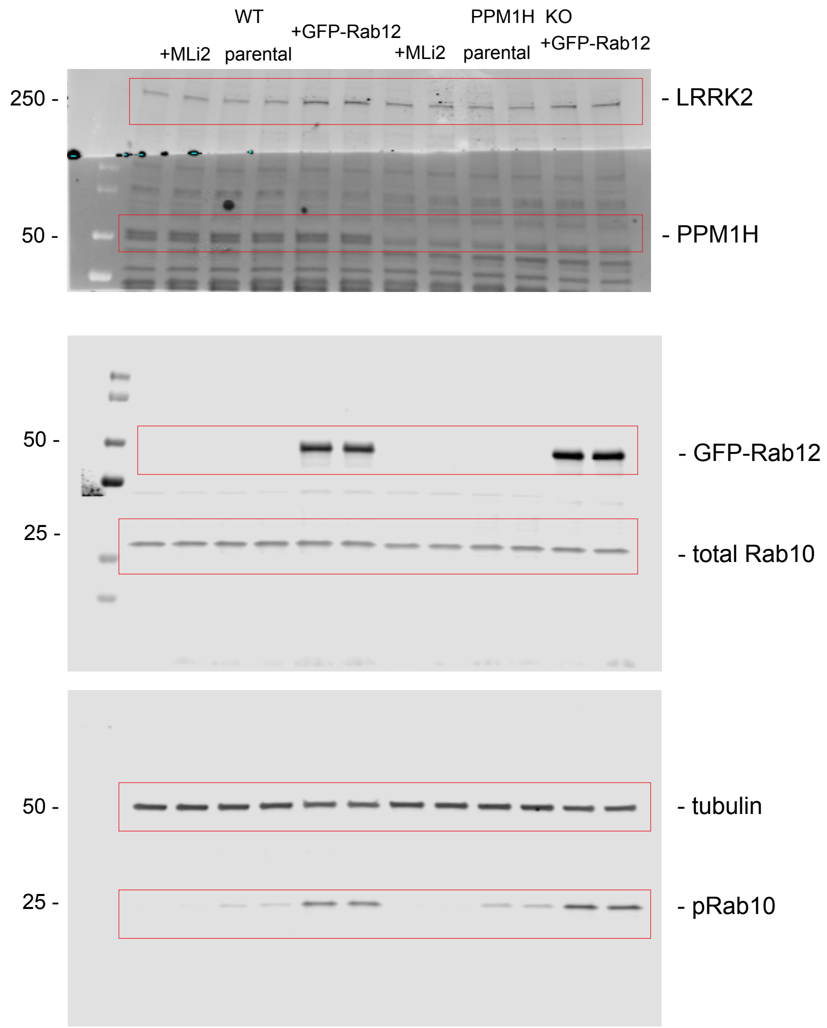
WT
+MLi2
parental
+GFP-Rab12
PPM1H KO
+MLi2
parental
+GFP-Rab12
250 -
50 -
- LRRK2
- PPM1H
50 -
25 -
- GFP-Rab12
- total Rab10
50 -
25 -
- tubulin
- pRab10

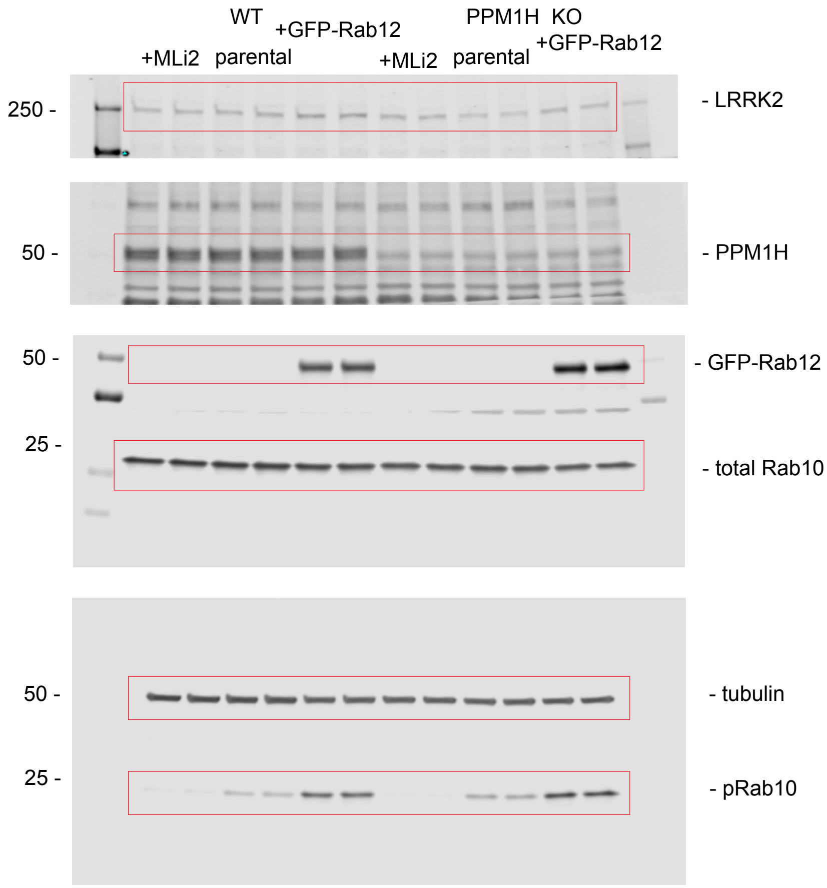
WT
+MLi2
parental
+GFP-Rab12
PPM1H KO
+MLi2
parental
+GFP-Rab12
250 -
- LRRK2
50 -
- PPM1H
50 -
25 -
- GFP-Rab12
- total Rab10
50 -
25 -
- tubulin
- pRab10

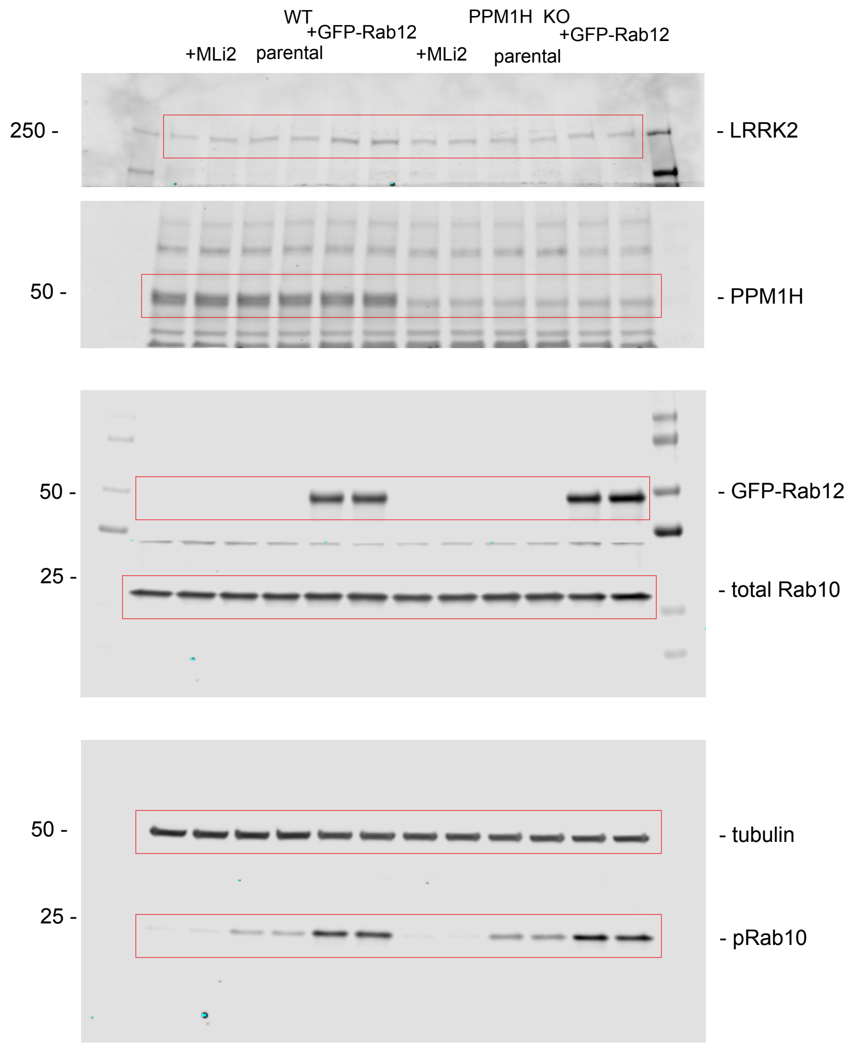
WT
+GFP-Rab12
+MLi2
parental
PPM1H KO
+GFP-Rab12
+MLi2
parental
250 -
- LRRK2
50 -
- PPM1H
50 -
- GFP-Rab12
25 -
- total Rab10
50 -
- tubulin
25 -
- pRab10

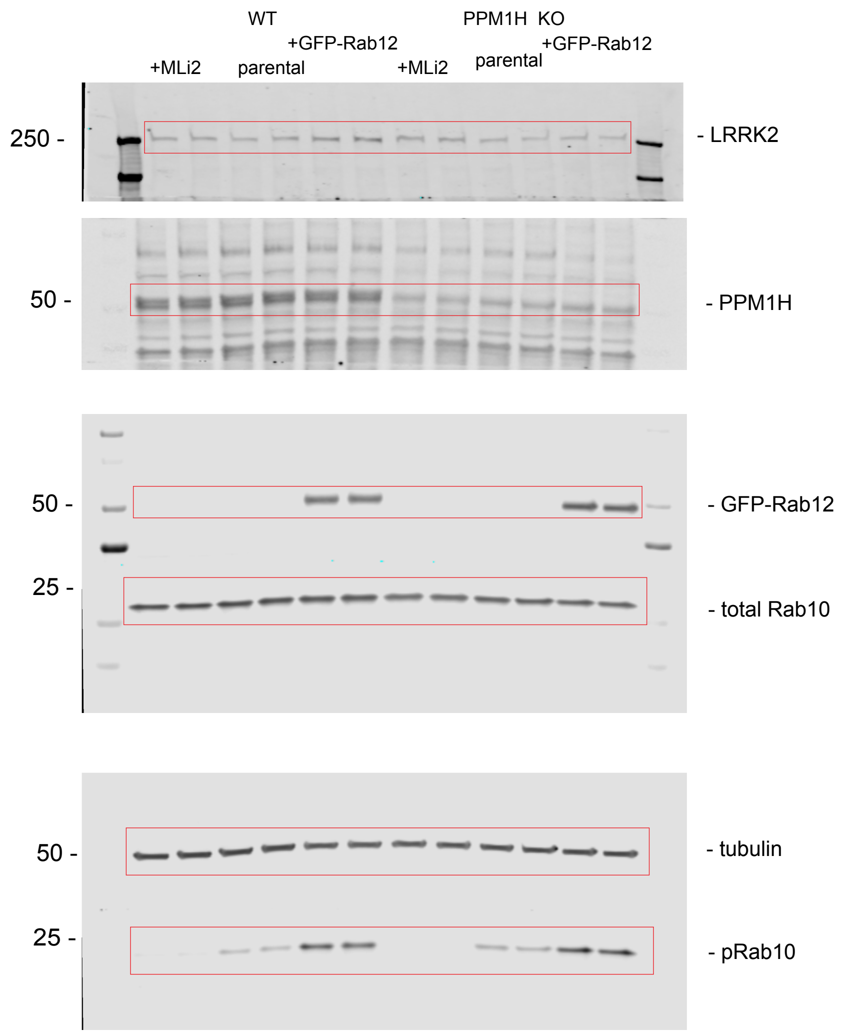
WT
+GFP-Rab12
+MLi2
parental
PPM1H KO
+GFP-Rab12
+MLi2
parental
250 -
- LRRK2
50 -
- PPM1H
50 -
- GFP-Rab12
25 -
- total Rab10
50 -
- tubulin
25 -
- pRab10
