# Supplementary figures and images for: Genome-wide screen reveals Rab12 GTPase as a critical activator of Parkinson’s disease-linked LRRK2 kinase (part 2 of 2)
Source: eLife. 2023 Oct 24;12:e87098. doi: 10.7554/eLife.87098 (PMC10708890; doi:10.7554/eLife.87098)

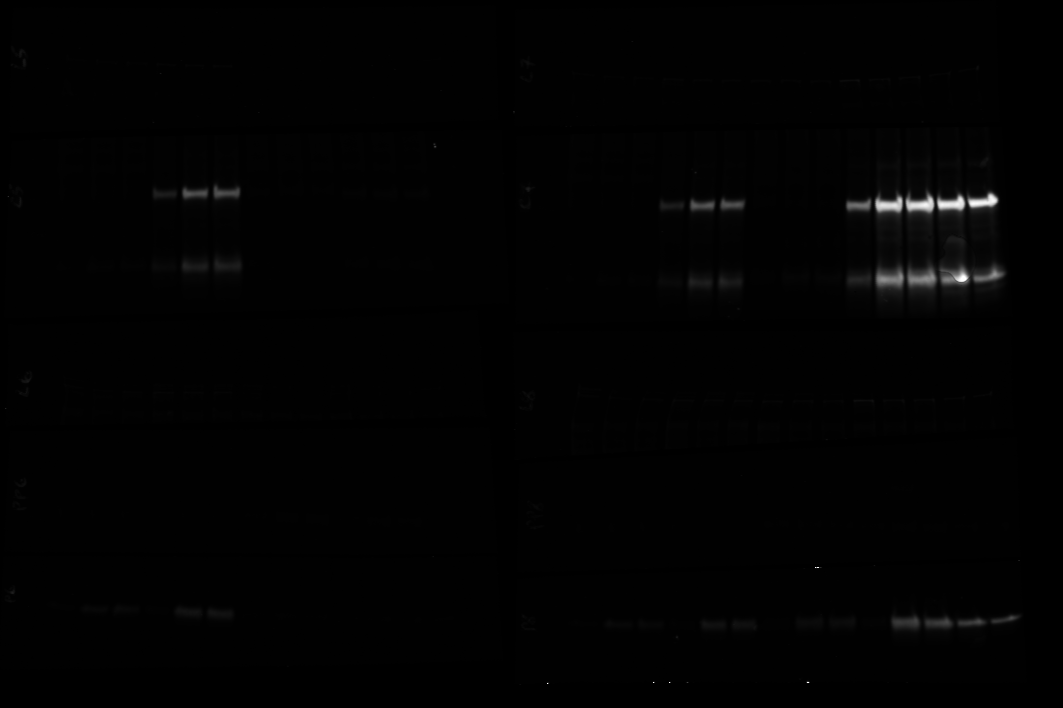

Supplement: Figure 5—source data 1. [file elife-87098-fig5-data1.zip › Figure 5-source data 1/Figure 5A-source data 1/800_1.tif]

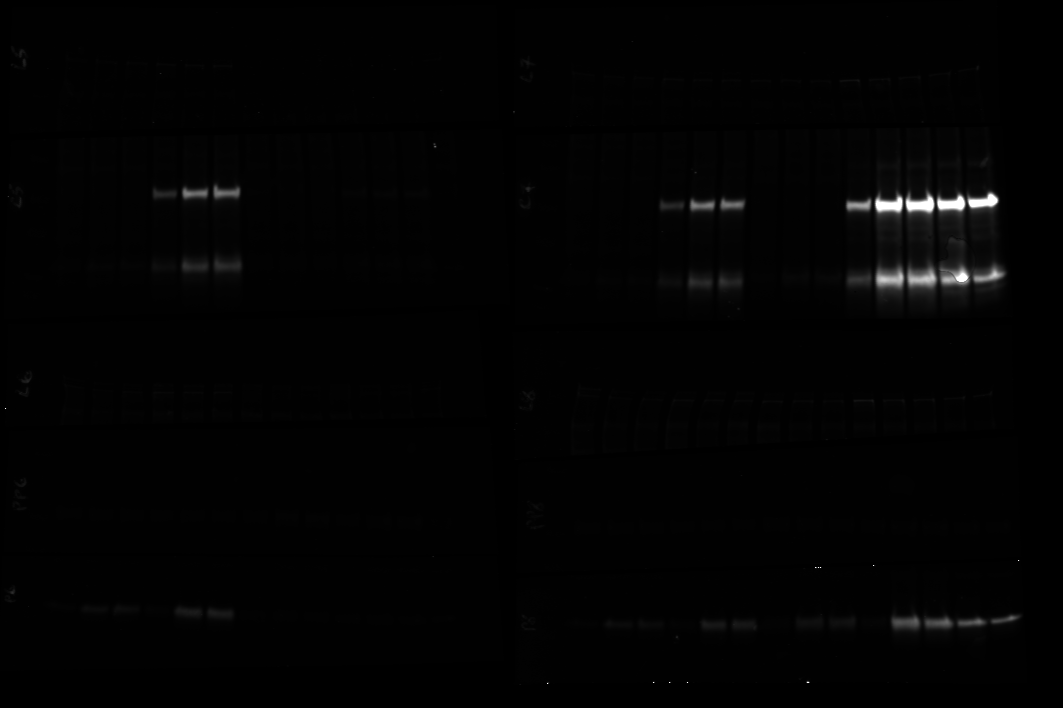

Supplement: Figure 5—source data 1. [file elife-87098-fig5-data1.zip › Figure 5-source data 1/Figure 5A-source data 1/800_2.tif]

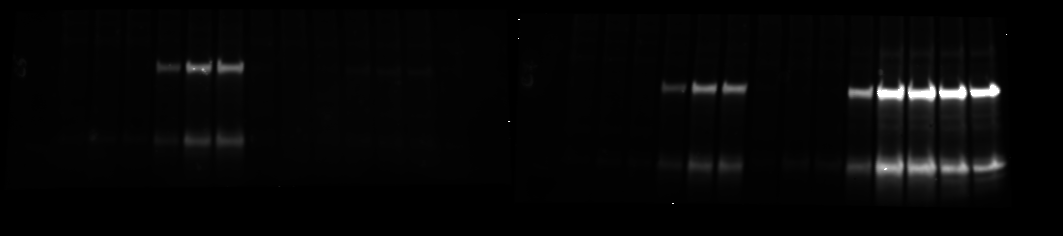

Supplement: Figure 5—source data 1. [file elife-87098-fig5-data1.zip › Figure 5-source data 1/Figure 5A-source data 1/800_3.tif]

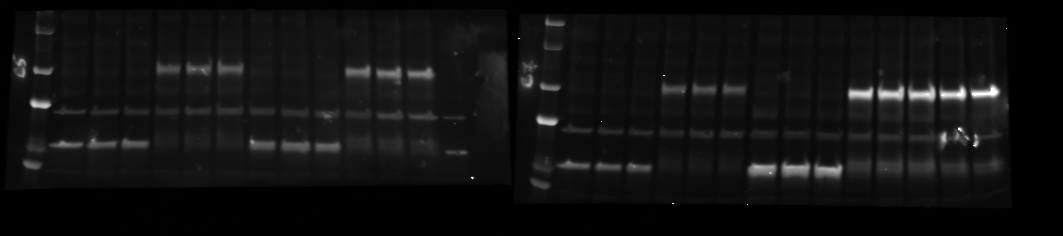

Supplement: Figure 5—source data 1. [file elife-87098-fig5-data1.zip › Figure 5-source data 1/Figure 5A-source data 1/700_3.tif]

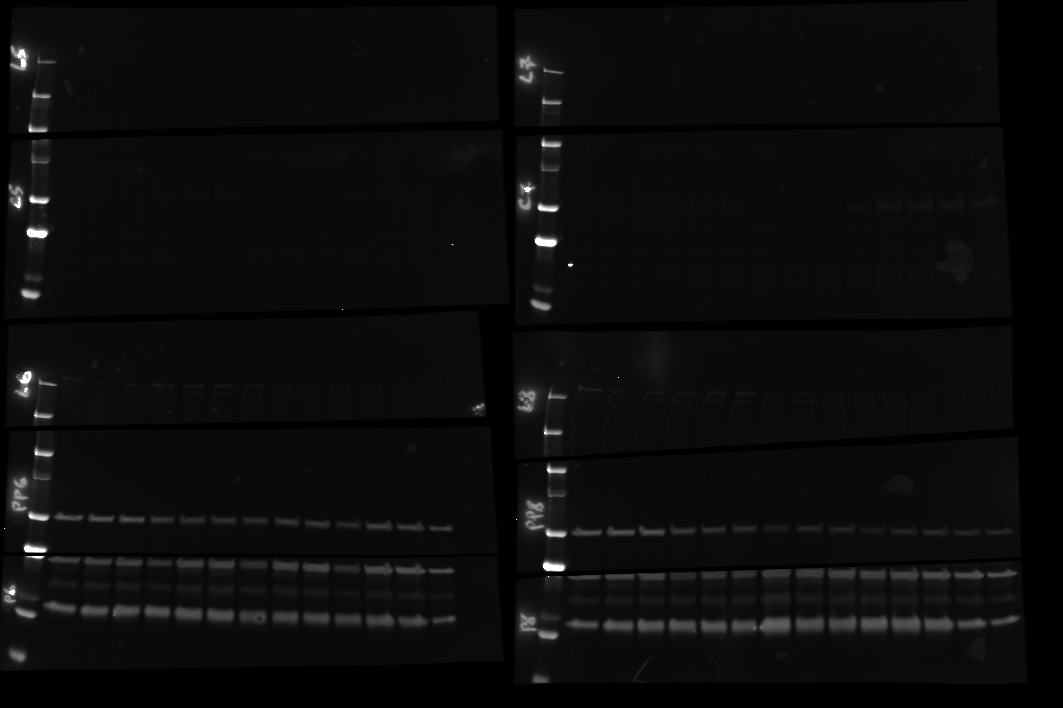

Supplement: Figure 5—source data 1. [file elife-87098-fig5-data1.zip › Figure 5-source data 1/Figure 5A-source data 1/700_2.tif]

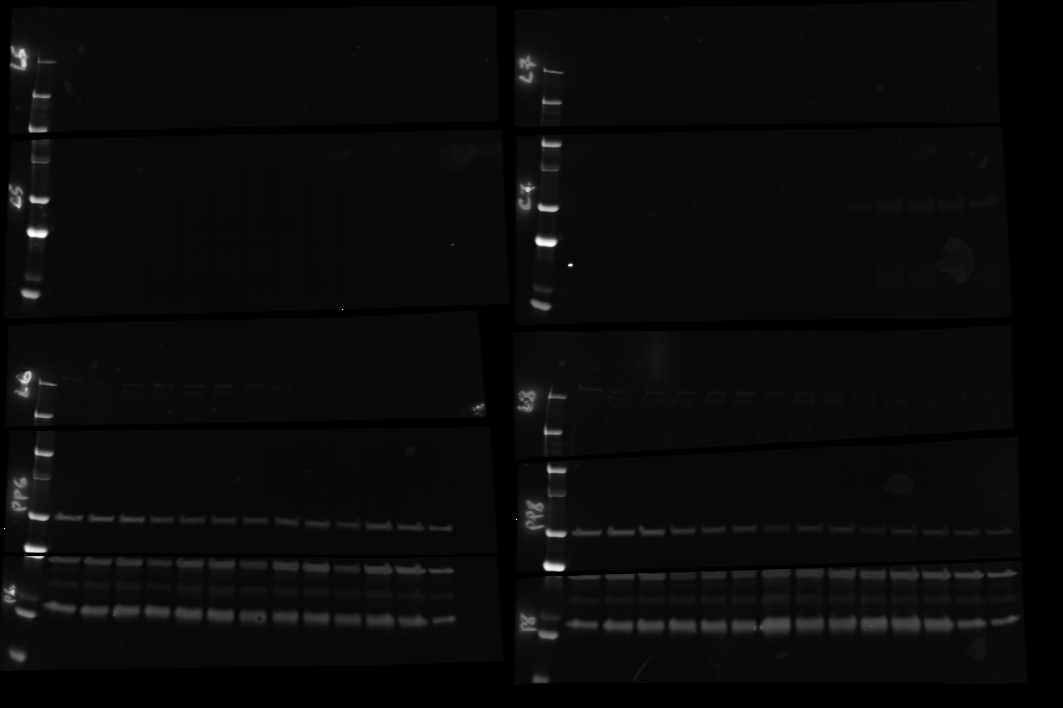

Supplement: Figure 5—source data 1. [file elife-87098-fig5-data1.zip › Figure 5-source data 1/Figure 5A-source data 1/700_1.tif]

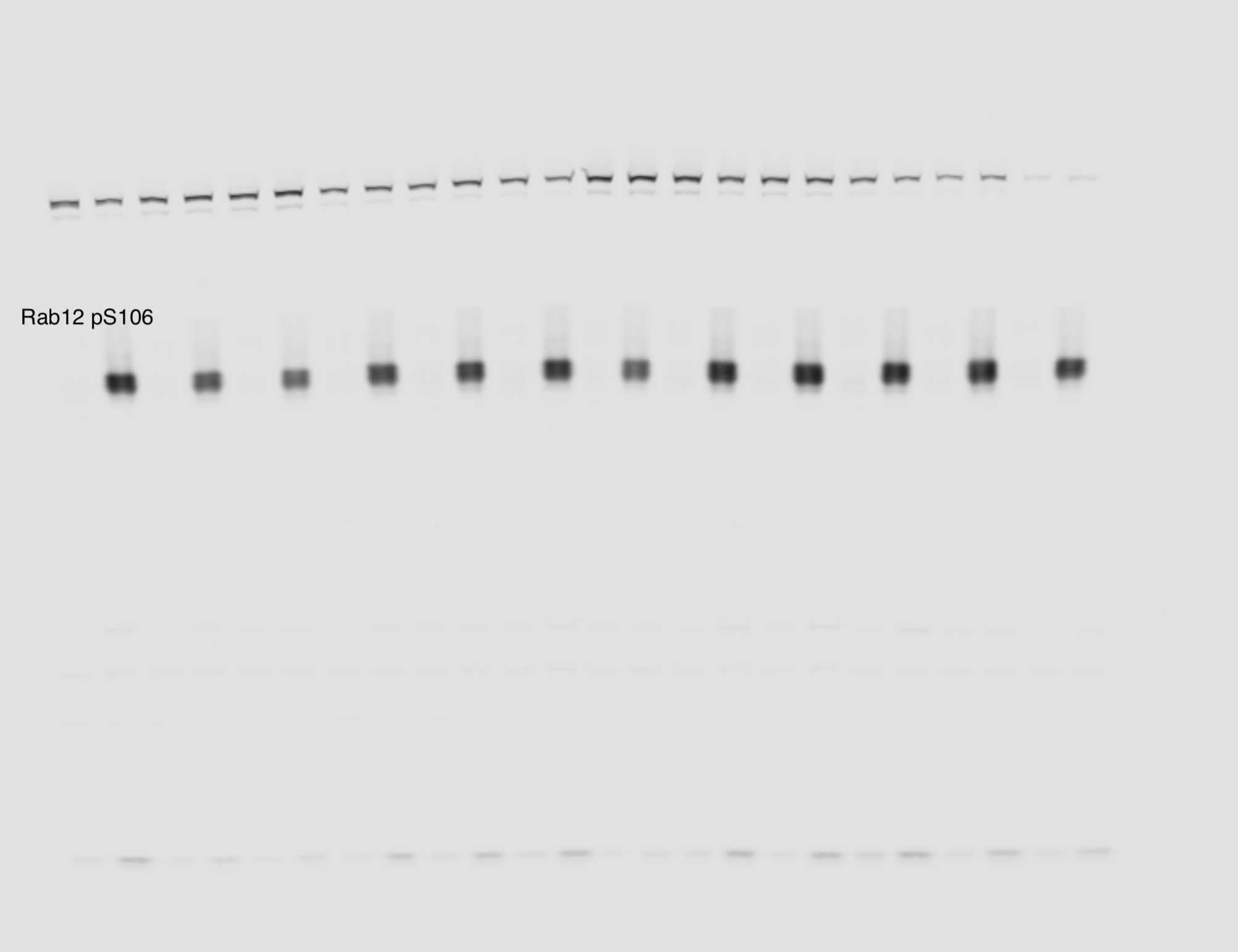

Supplement: Figure 7—figure supplement 1—source data 1. [file elife-87098-fig7-figsupp1-data1.zip › Figure 7-figure supplement 1-source data 1/annotated/Figure 7 Figure Suppl 1 Rab12_800-low.tif]

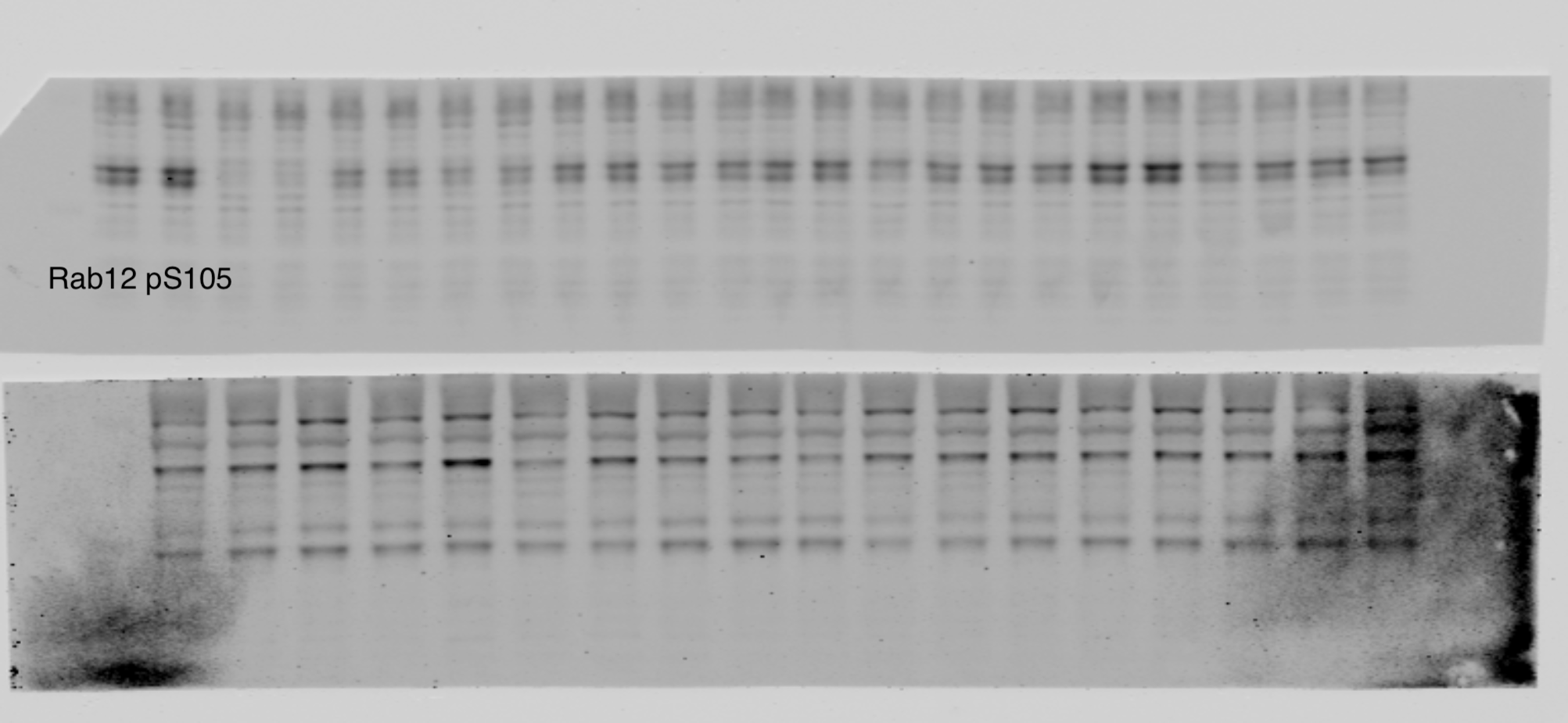

Supplement: Figure 7—figure supplement 1—source data 1. [file elife-87098-fig7-figsupp1-data1.zip › Figure 7-figure supplement 1-source data 1/annotated/Additional blots quantified in Figure 7B_800-2.tif]

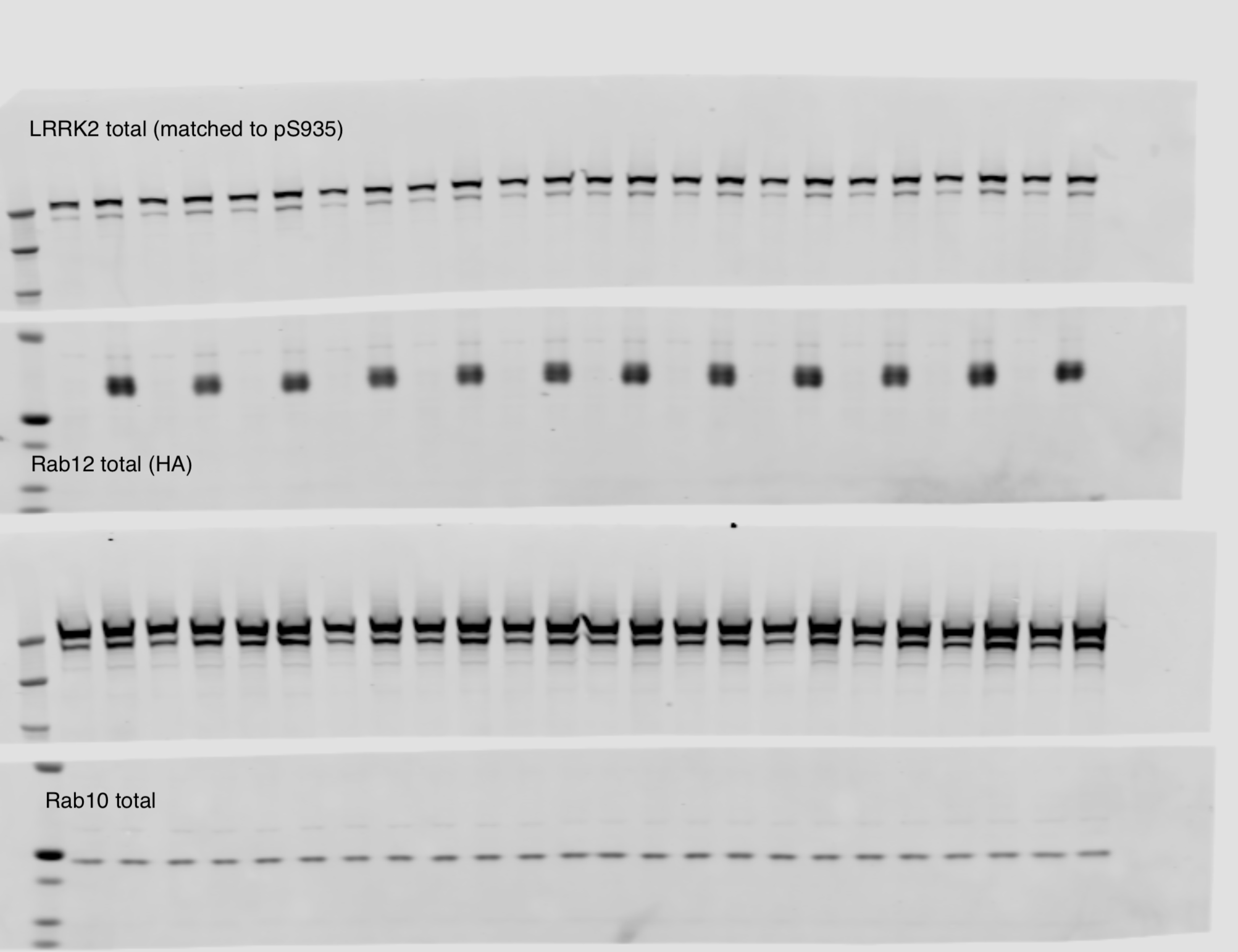

Supplement: Figure 7—figure supplement 1—source data 1. [file elife-87098-fig7-figsupp1-data1.zip › Figure 7-figure supplement 1-source data 1/annotated/Figure 7 Figure Suppl 1 Rab12_700.tif]

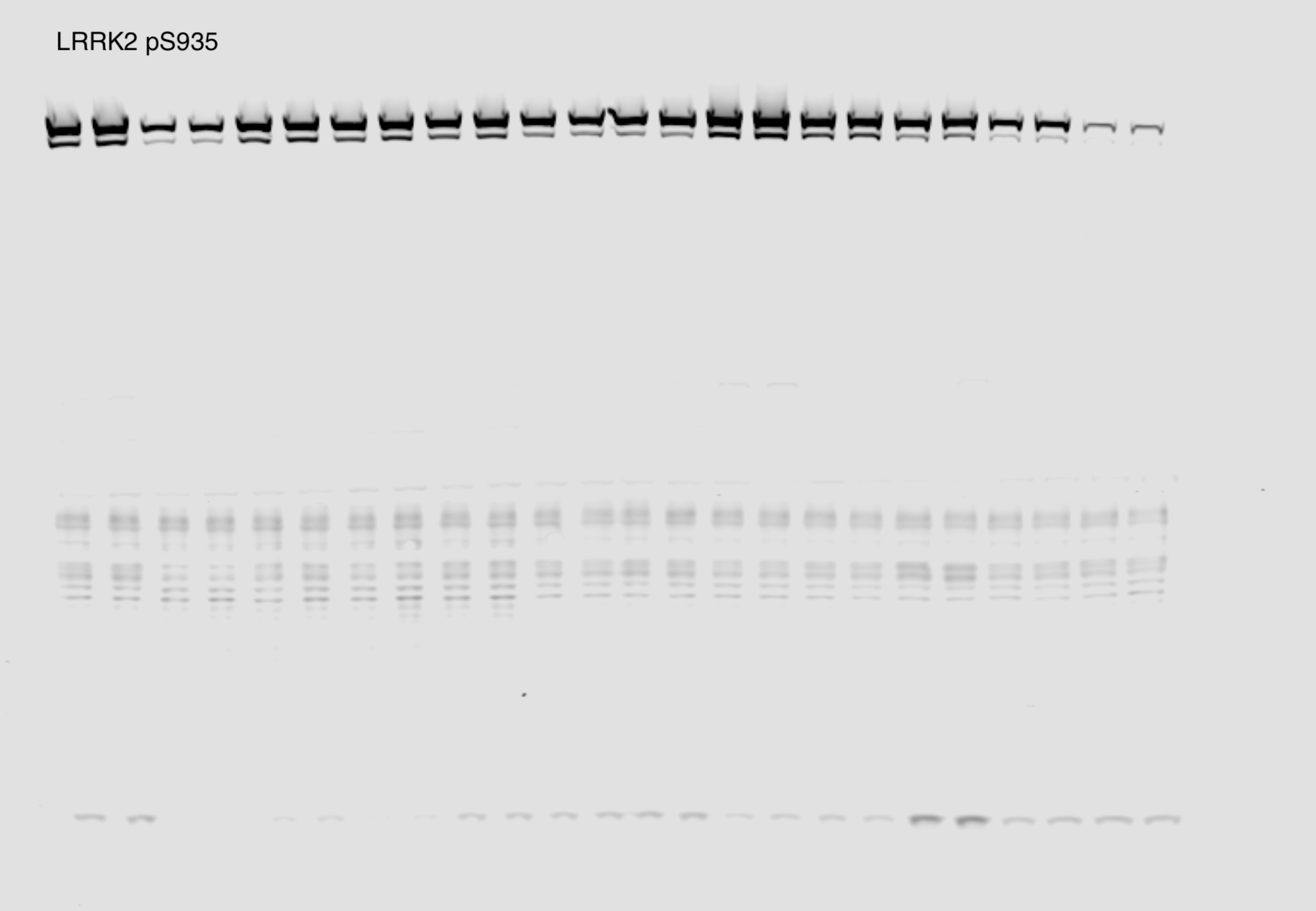

Supplement: Figure 7—figure supplement 1—source data 1. [file elife-87098-fig7-figsupp1-data1.zip › Figure 7-figure supplement 1-source data 1/annotated/Additional blots quantified in Figure 7B_800-low.tif]

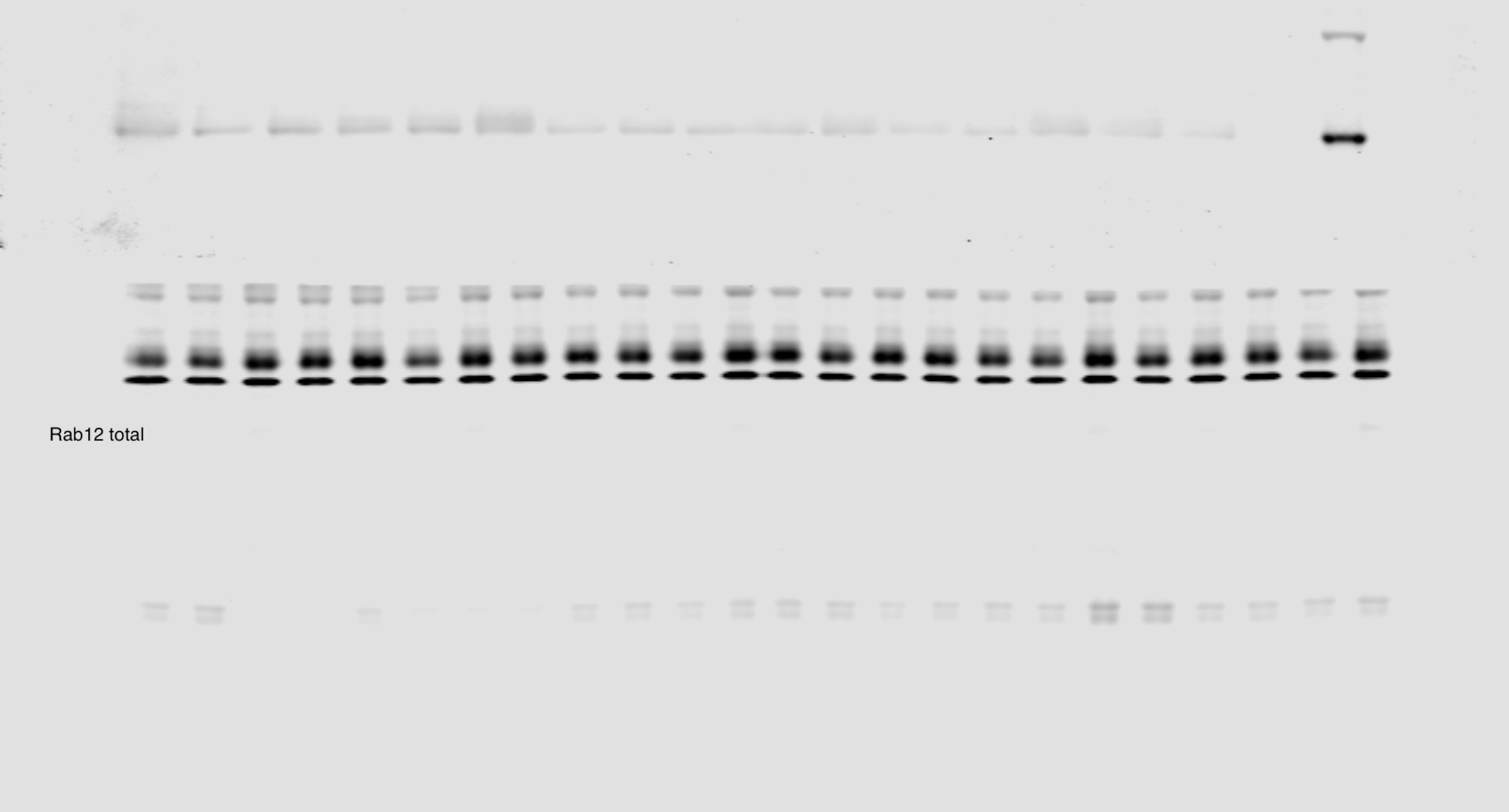

Supplement: Figure 7—figure supplement 1—source data 1. [file elife-87098-fig7-figsupp1-data1.zip › Figure 7-figure supplement 1-source data 1/annotated/Figure 7 Figure Suppl 1_800-low-2.tif]

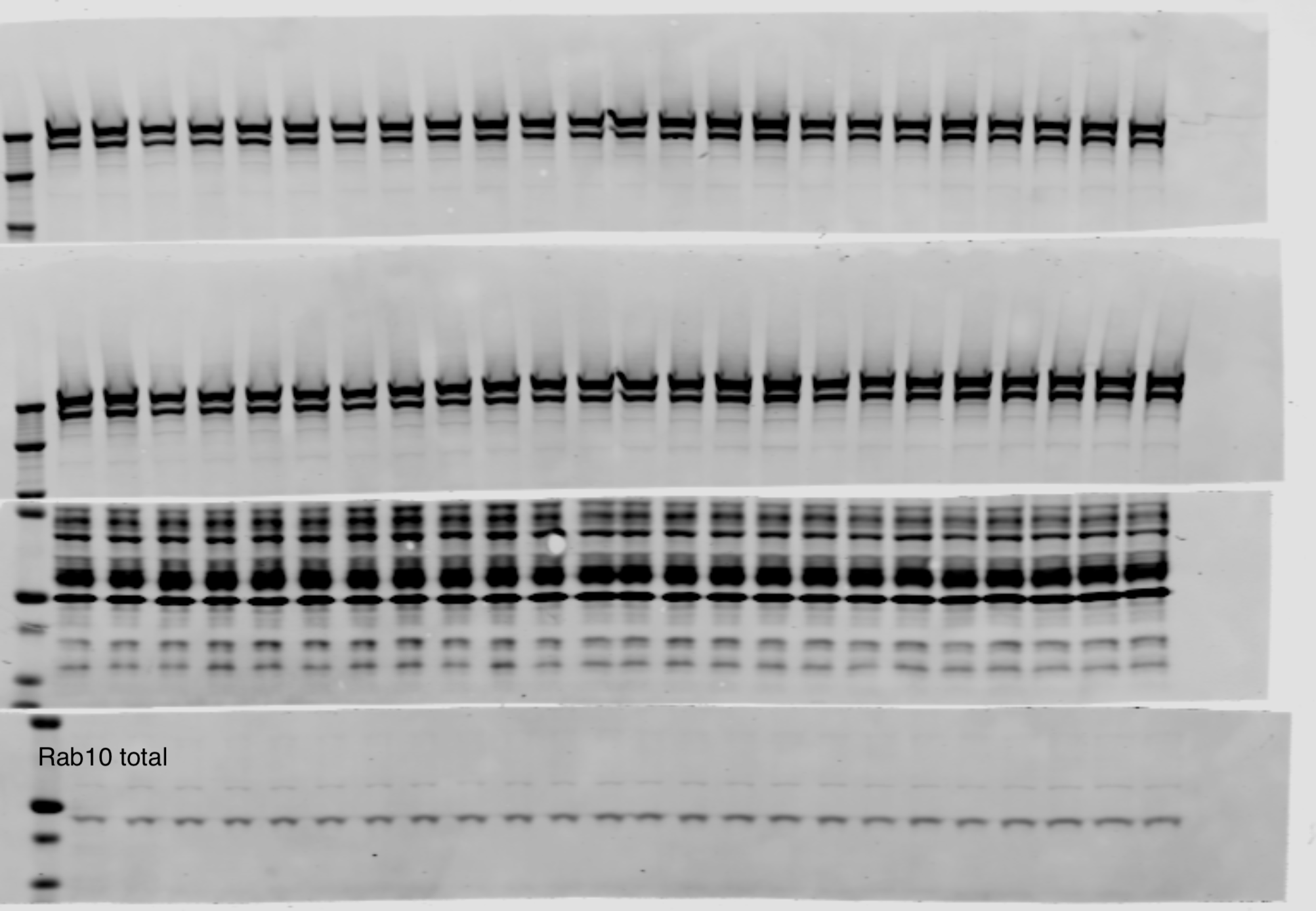

Supplement: Figure 7—figure supplement 1—source data 1. [file elife-87098-fig7-figsupp1-data1.zip › Figure 7-figure supplement 1-source data 1/annotated/Additional blots quantified in Figure 7B_700.tif]

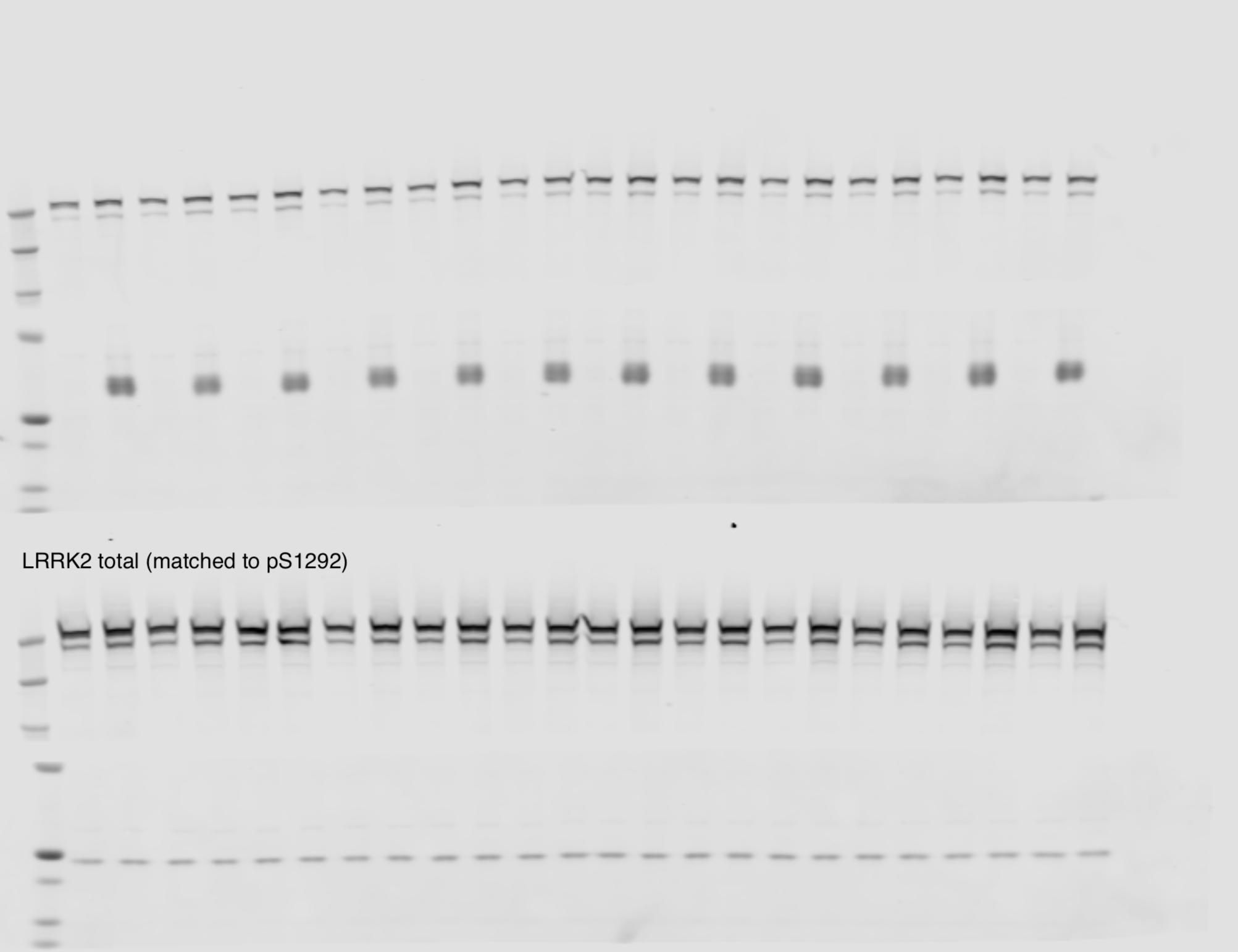

Supplement: Figure 7—figure supplement 1—source data 1. [file elife-87098-fig7-figsupp1-data1.zip › Figure 7-figure supplement 1-source data 1/annotated/Figure 7 Figure Suppl 1 Rab12_700-low.tif]

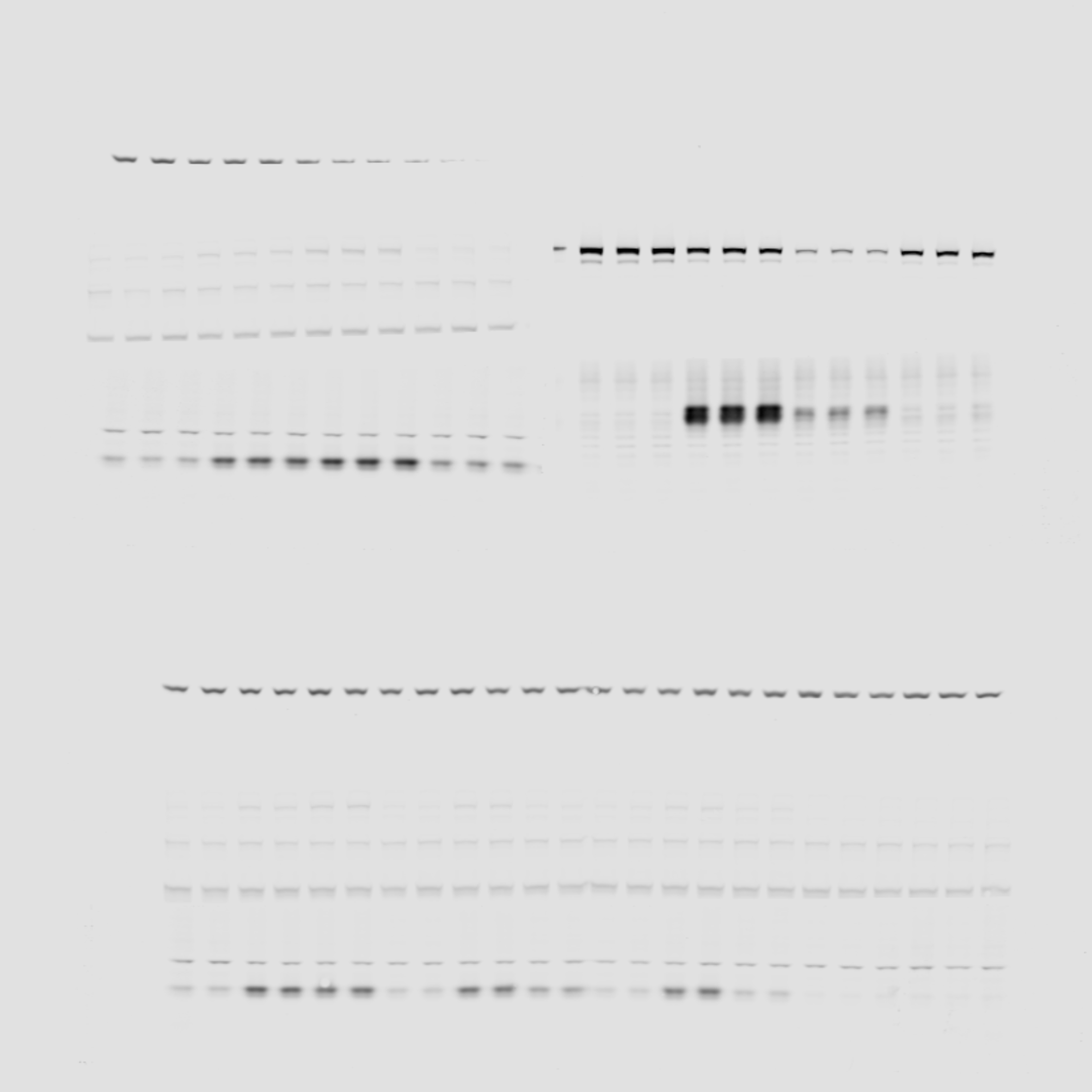

Supplement: Figure 7—figure supplement 1—source data 1. [file elife-87098-fig7-figsupp1-data1.zip › Figure 7-figure supplement 1-source data 1/raw images/Fig7_Suppl1_28-04-2023_800-low.tif]

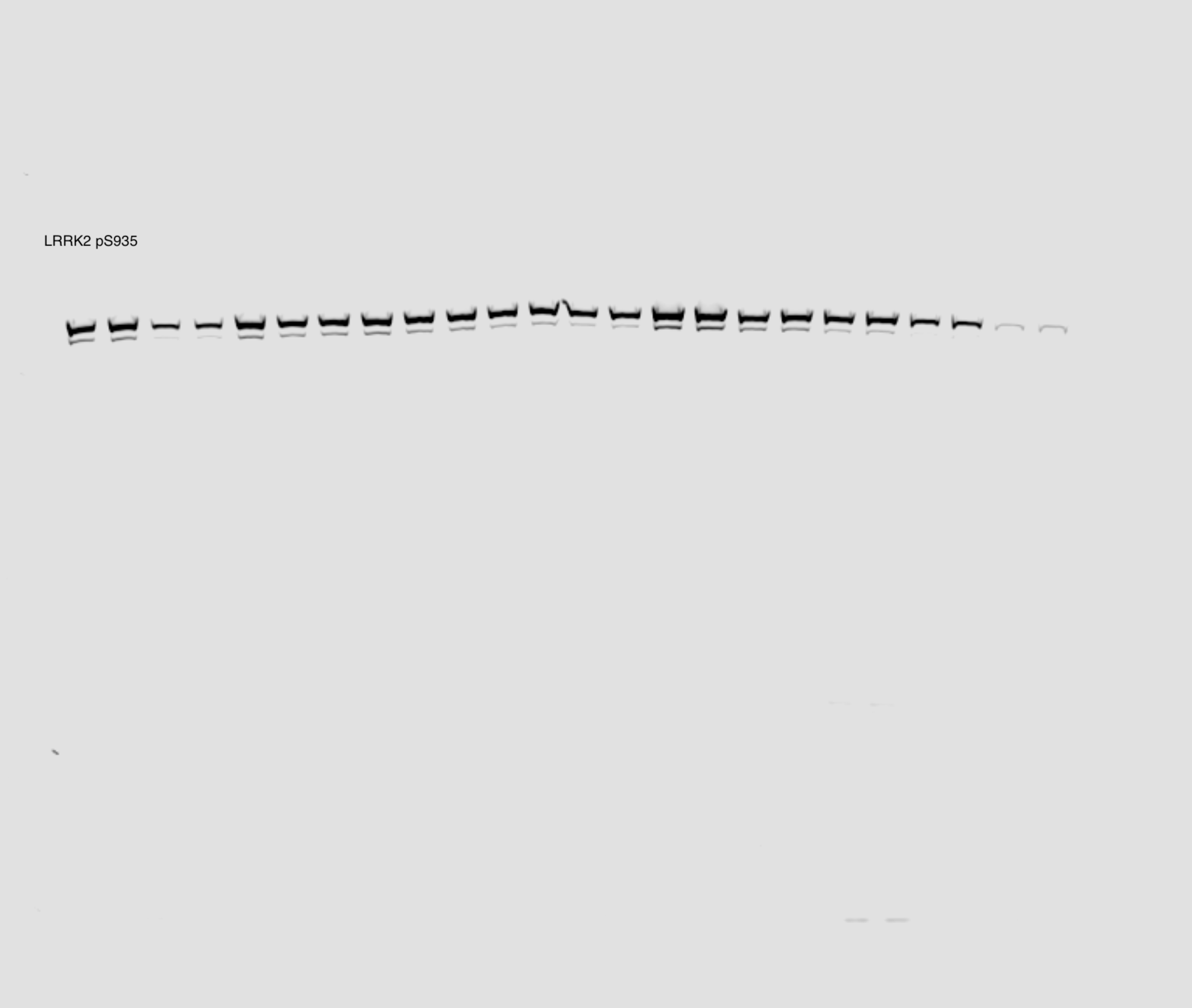

Supplement: Figure 7—figure supplement 1—source data 1. [file elife-87098-fig7-figsupp1-data1.zip › Figure 7-figure supplement 1-source data 1/annotated/Figure 7 Figure Suppl 1_800-low.tif]

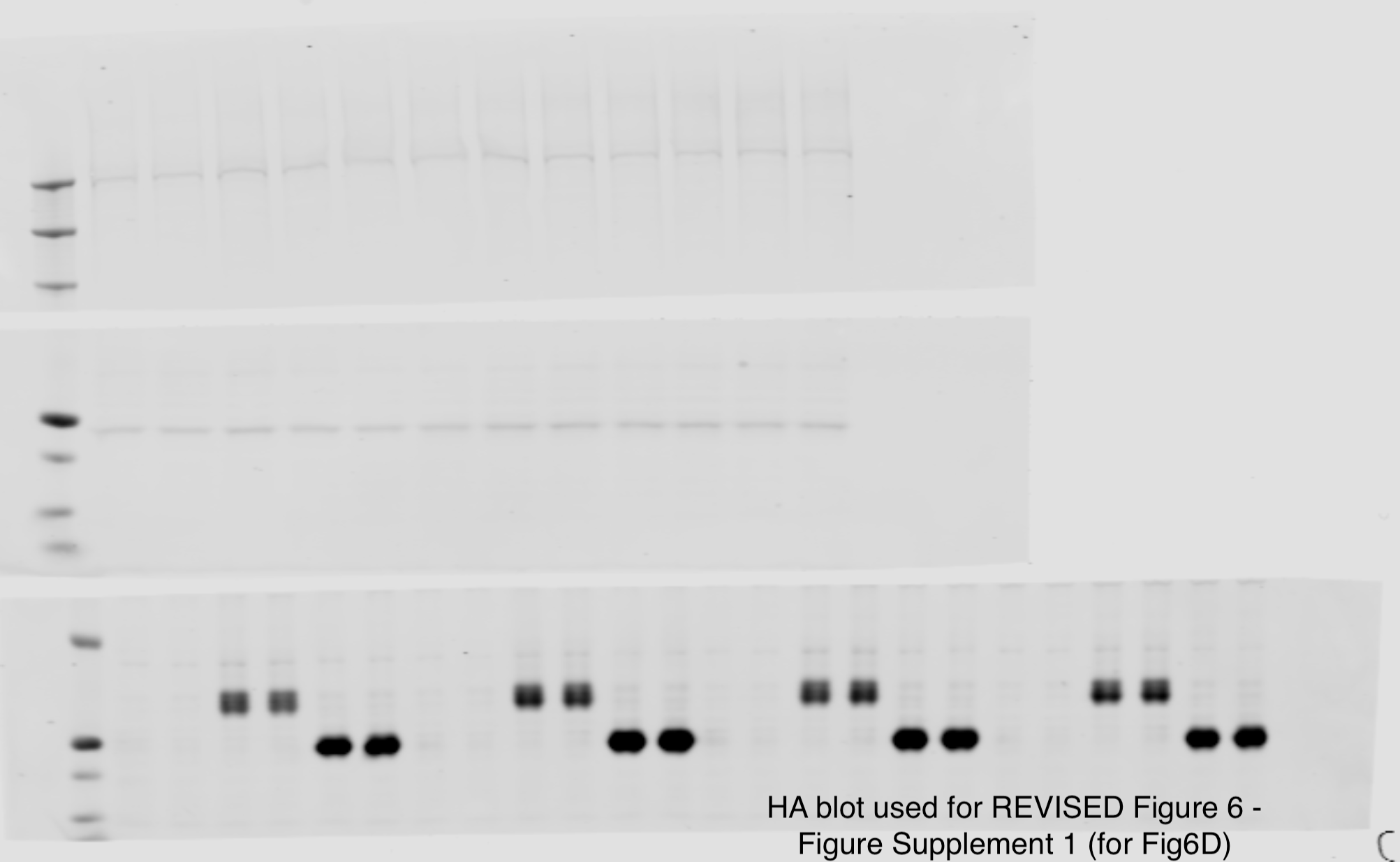

Supplement: Figure 7—figure supplement 1—source data 1. [file elife-87098-fig7-figsupp1-data1.zip › Figure 7-figure supplement 1-source data 1/annotated/REVISED-Fig7-FigSupplement1_03-05-2023_700.tif]

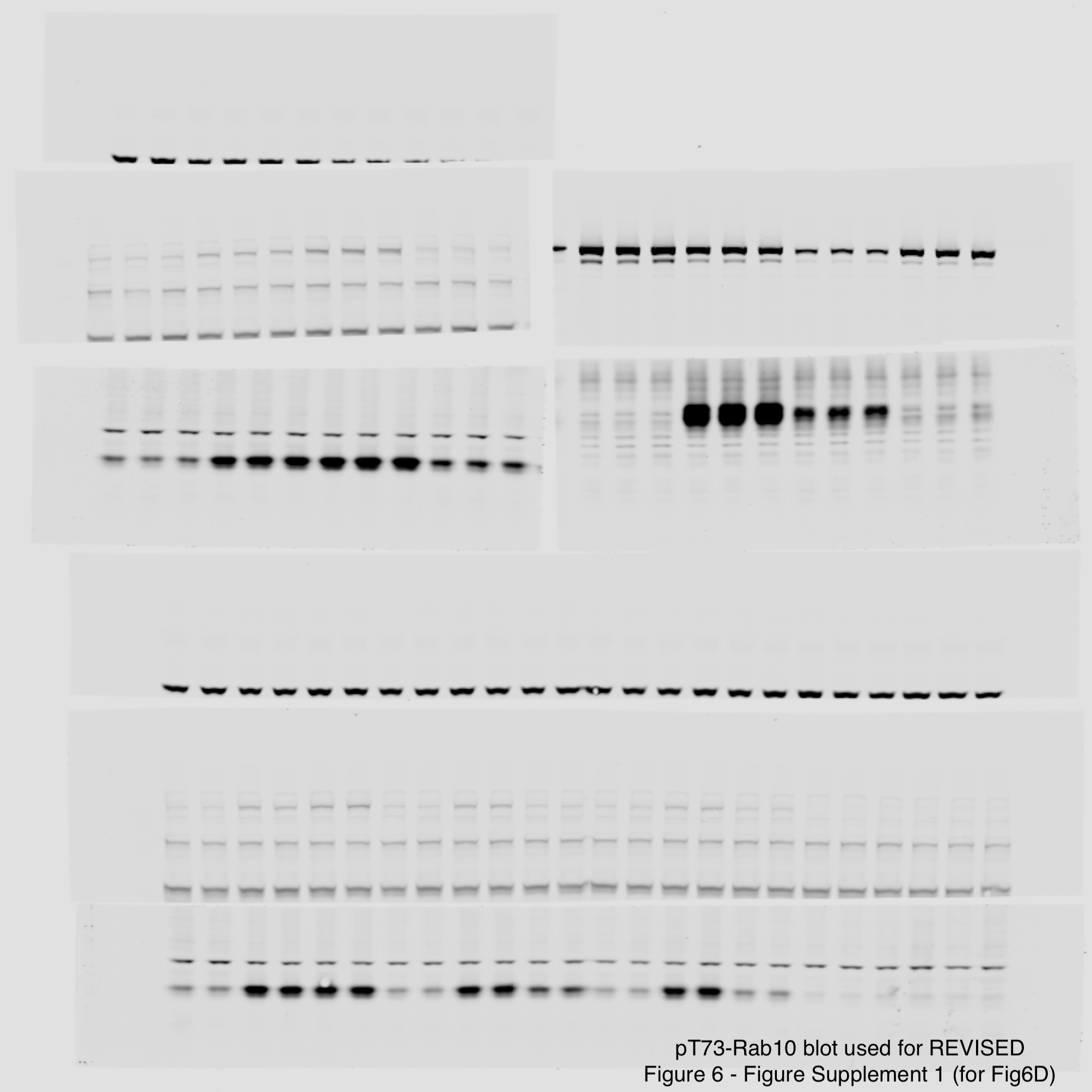

Supplement: Figure 7—figure supplement 1—source data 1. [file elife-87098-fig7-figsupp1-data1.zip › Figure 7-figure supplement 1-source data 1/annotated/REVISED-Fig7-FigSupplement1_28-04-2023_800.tif]

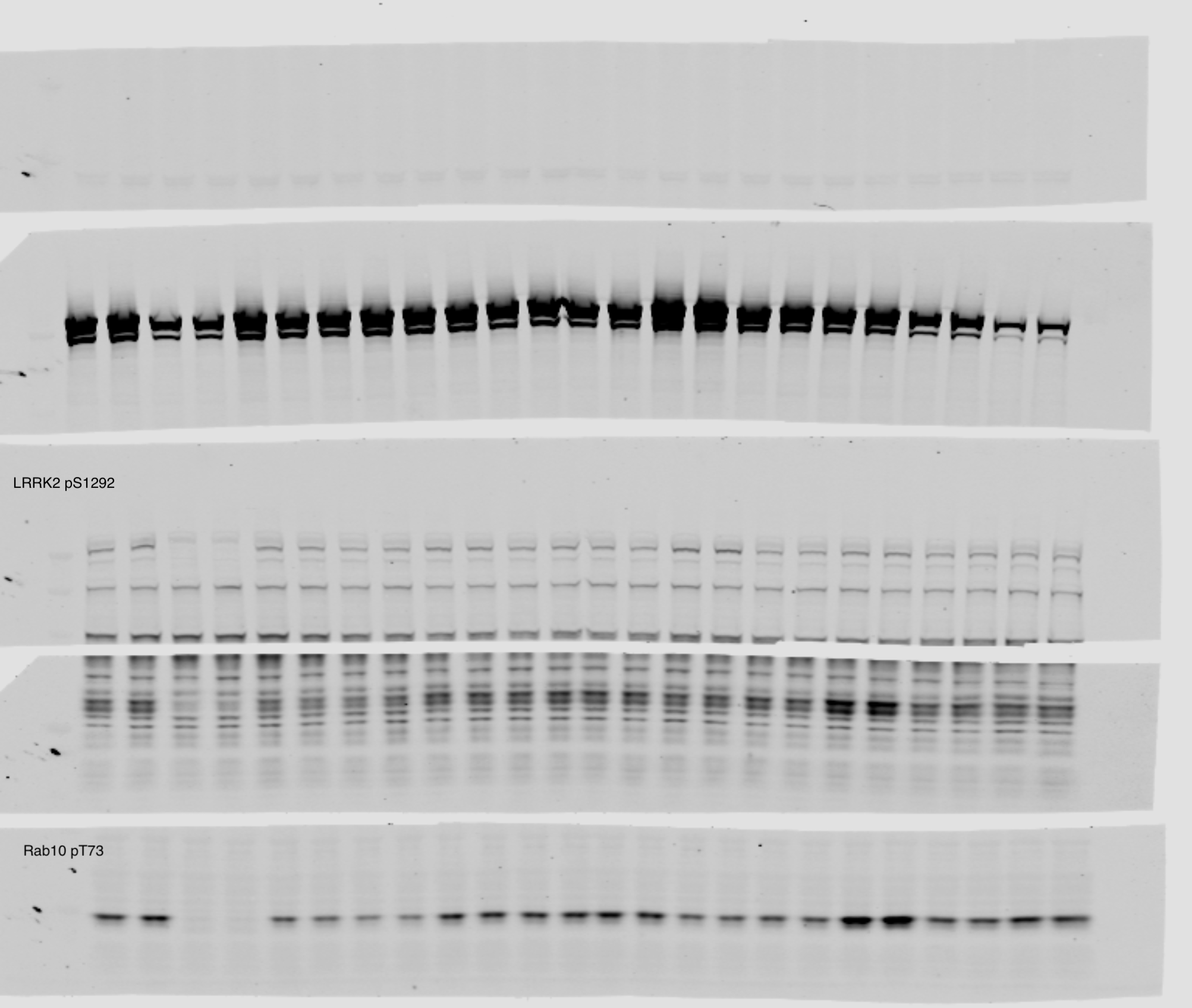

Supplement: Figure 7—figure supplement 1—source data 1. [file elife-87098-fig7-figsupp1-data1.zip › Figure 7-figure supplement 1-source data 1/annotated/Figure 7 Figure Suppl 1_800.tif]

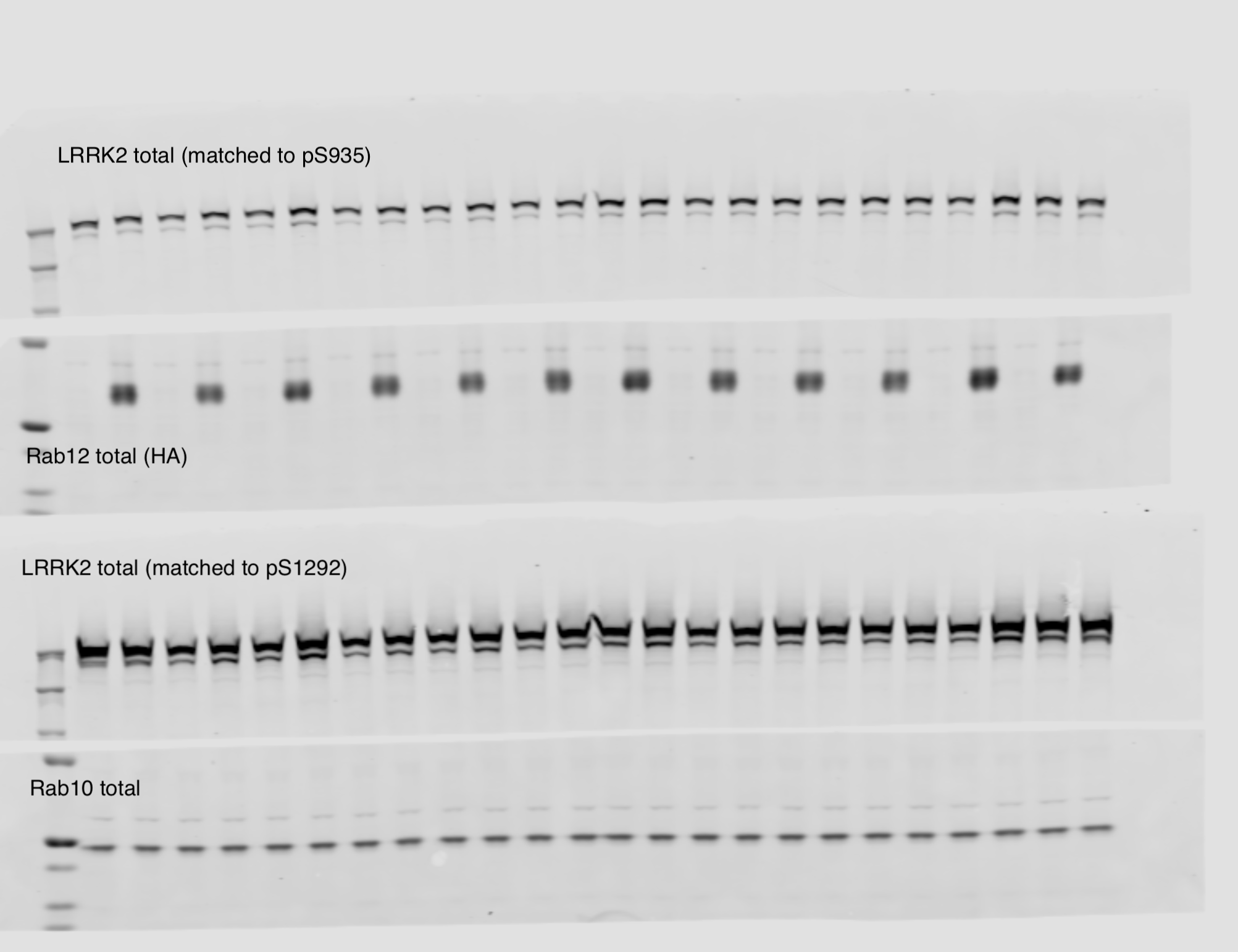

Supplement: Figure 7—figure supplement 1—source data 1. [file elife-87098-fig7-figsupp1-data1.zip › Figure 7-figure supplement 1-source data 1/annotated/Additional blots quantified in Figure 7C_700.tif]

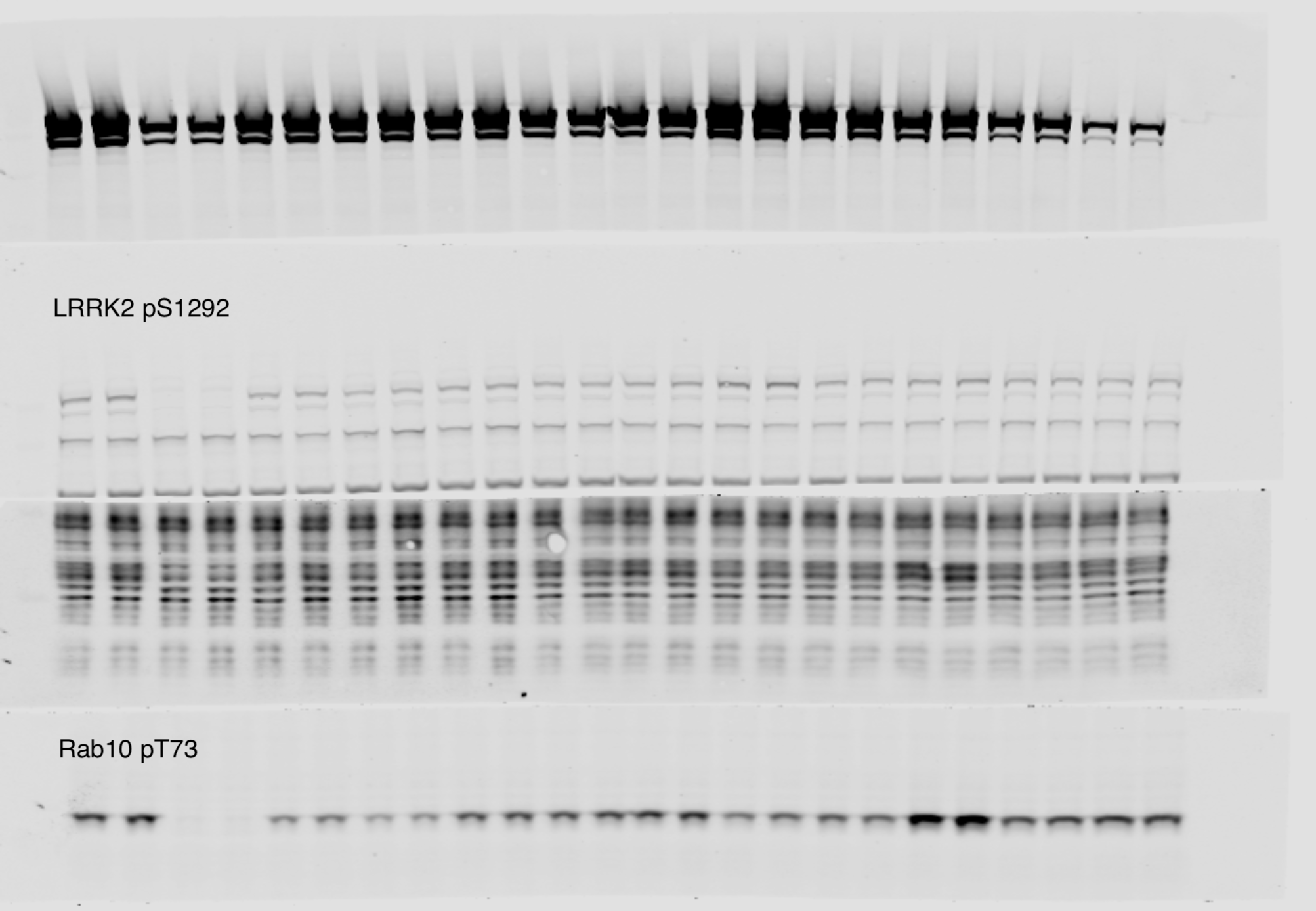

Supplement: Figure 7—figure supplement 1—source data 1. [file elife-87098-fig7-figsupp1-data1.zip › Figure 7-figure supplement 1-source data 1/annotated/Additional blots quantified in Figure 7B_800.tif]

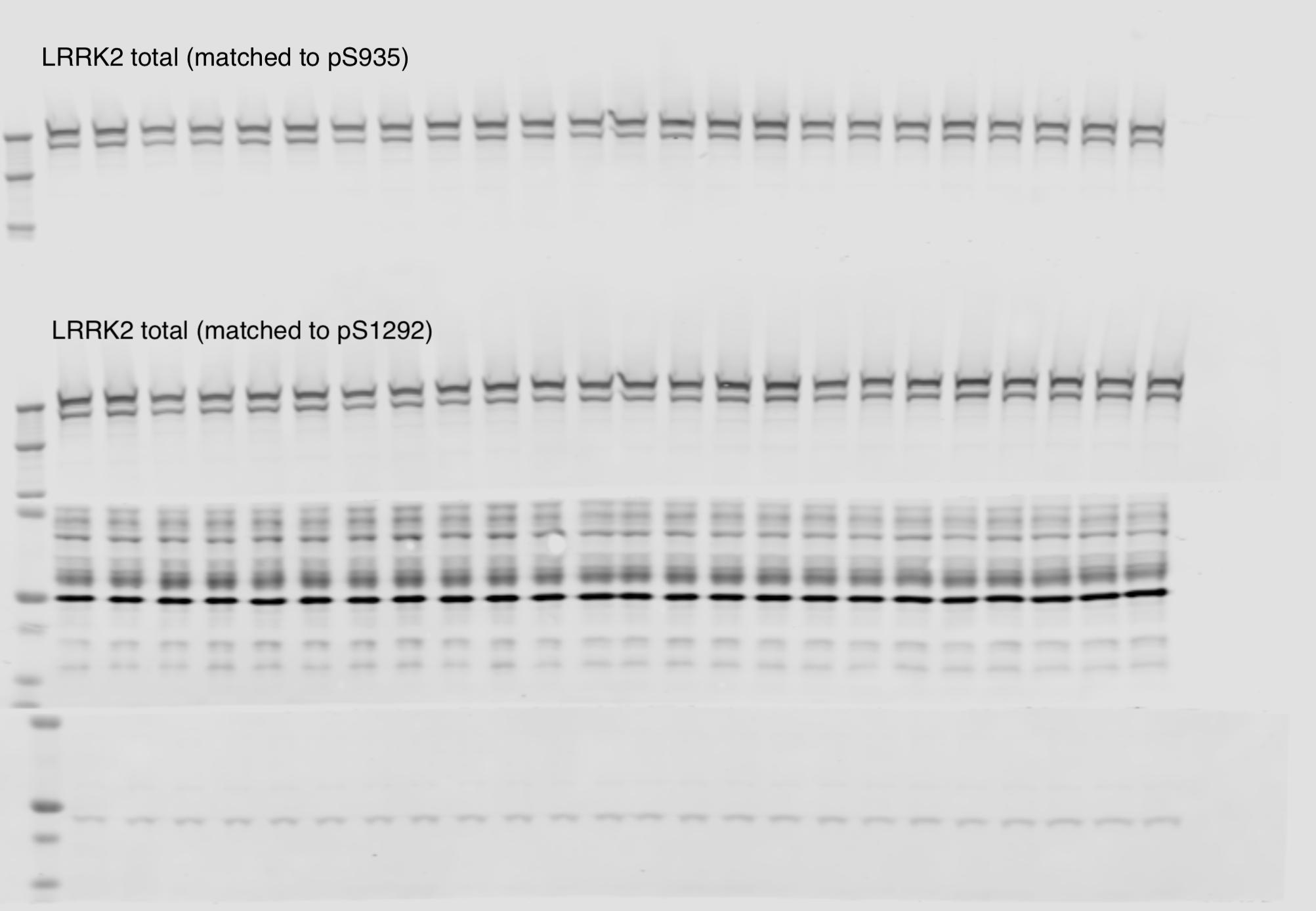

Supplement: Figure 7—figure supplement 1—source data 1. [file elife-87098-fig7-figsupp1-data1.zip › Figure 7-figure supplement 1-source data 1/annotated/Additional blots quantified in Figure 7B_700-low.tif]

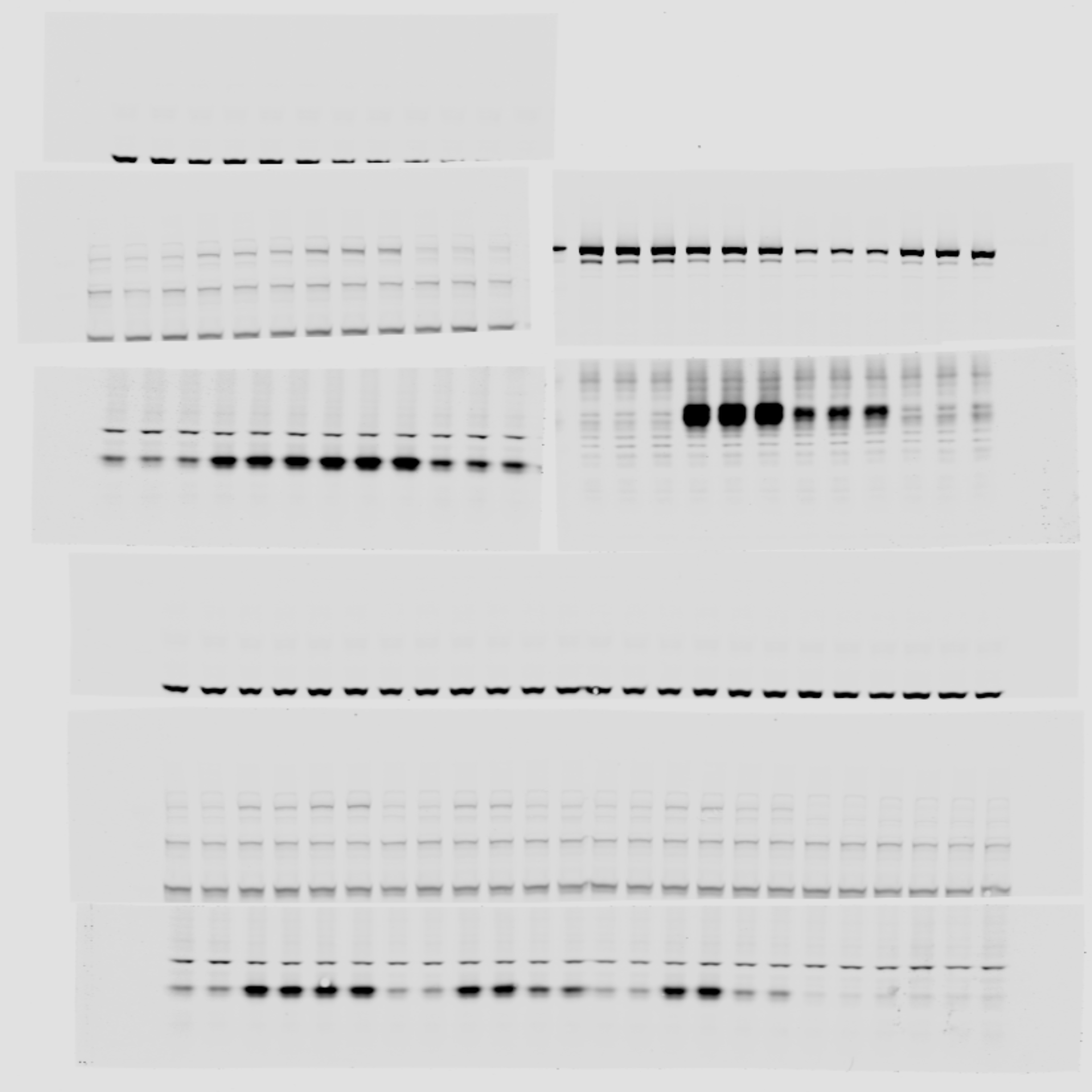

Supplement: Figure 7—figure supplement 1—source data 1. [file elife-87098-fig7-figsupp1-data1.zip › Figure 7-figure supplement 1-source data 1/raw images/Fig7_Suppl1_28-04-2023_800.tif]

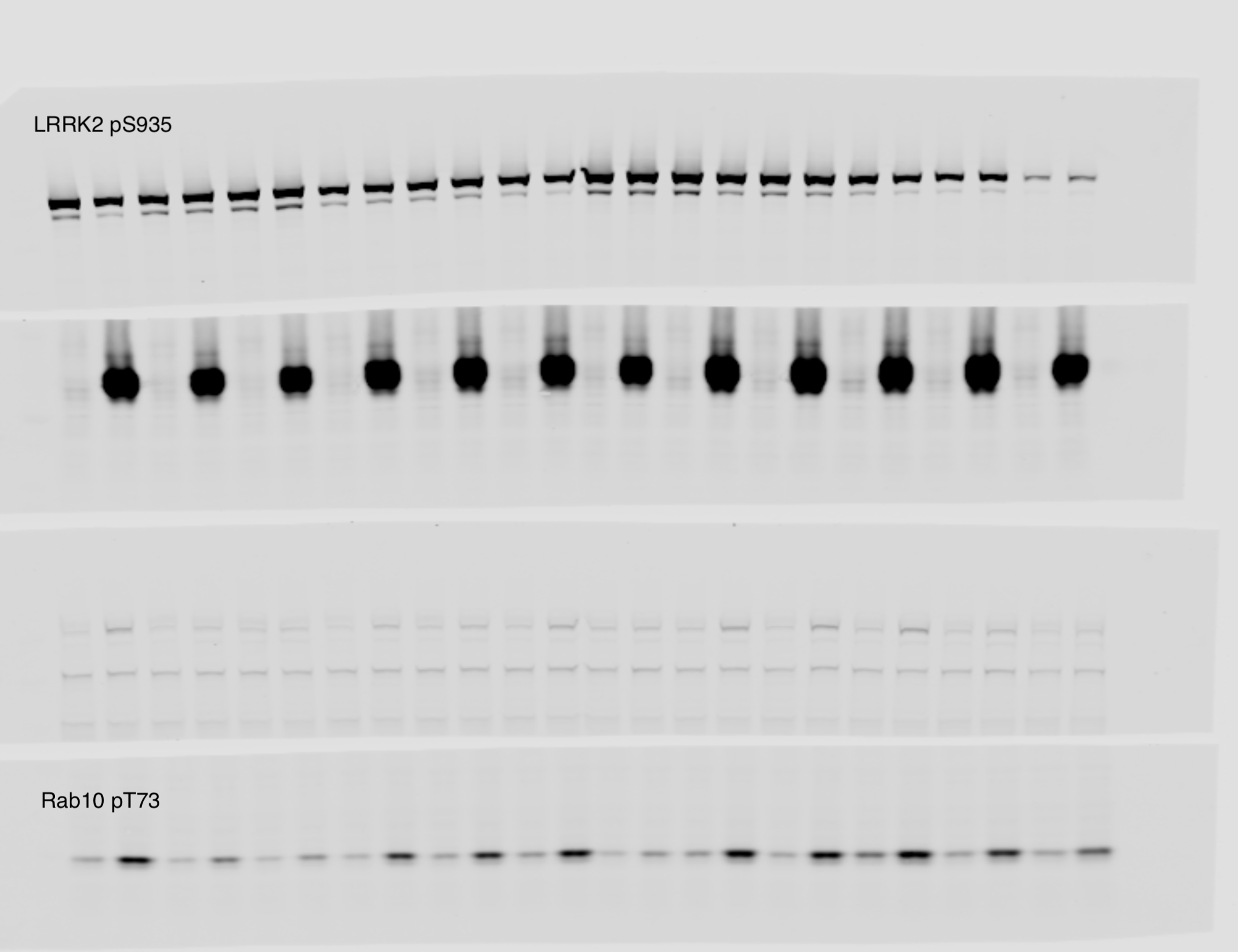

Supplement: Figure 7—figure supplement 1—source data 1. [file elife-87098-fig7-figsupp1-data1.zip › Figure 7-figure supplement 1-source data 1/annotated/Figure 7 Figure Suppl 1 Rab12_800.tif]

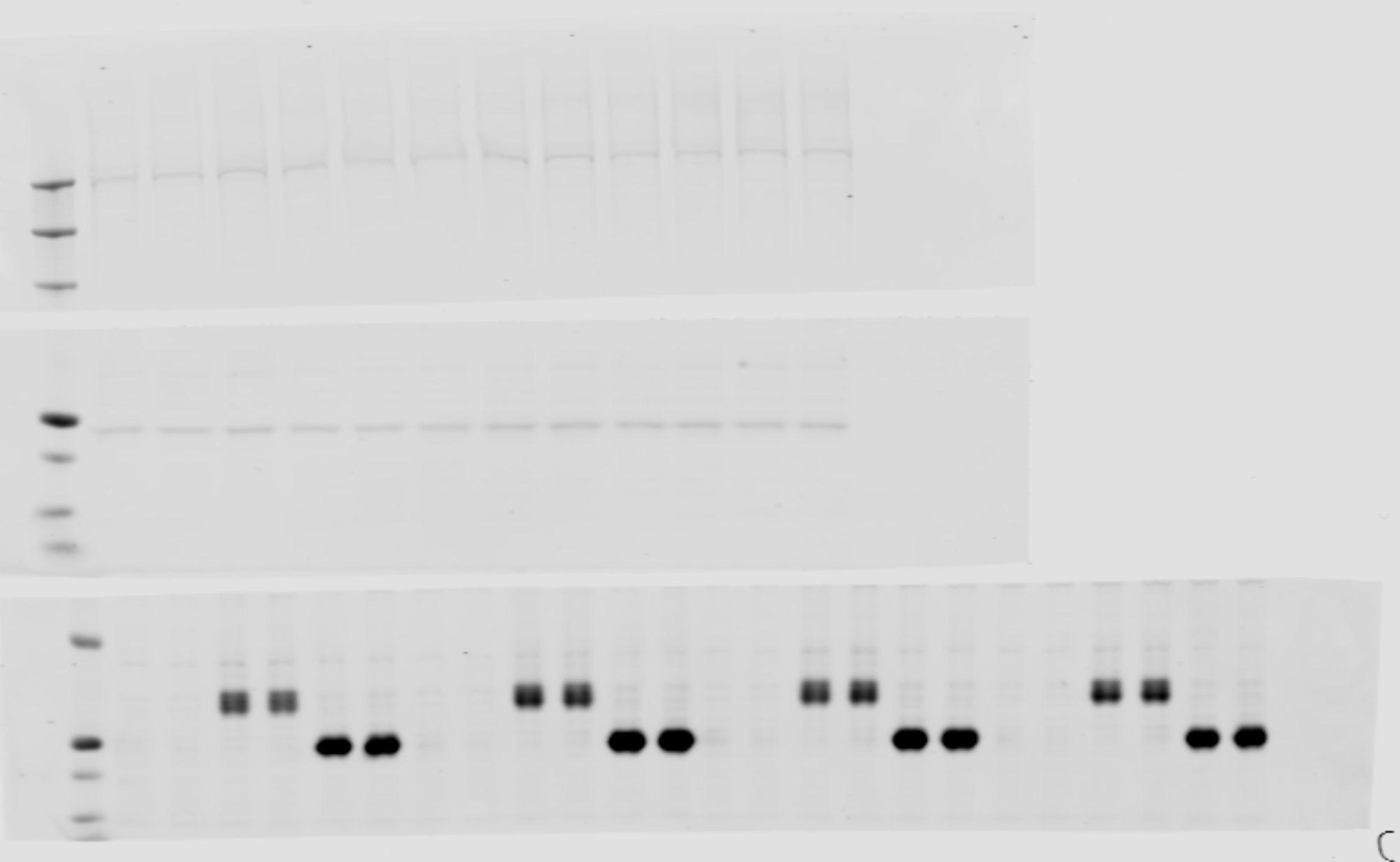

Supplement: Figure 7—figure supplement 1—source data 1. [file elife-87098-fig7-figsupp1-data1.zip › Figure 7-figure supplement 1-source data 1/raw images/Fig7_Suppl1_03-05-2023_700.tif]

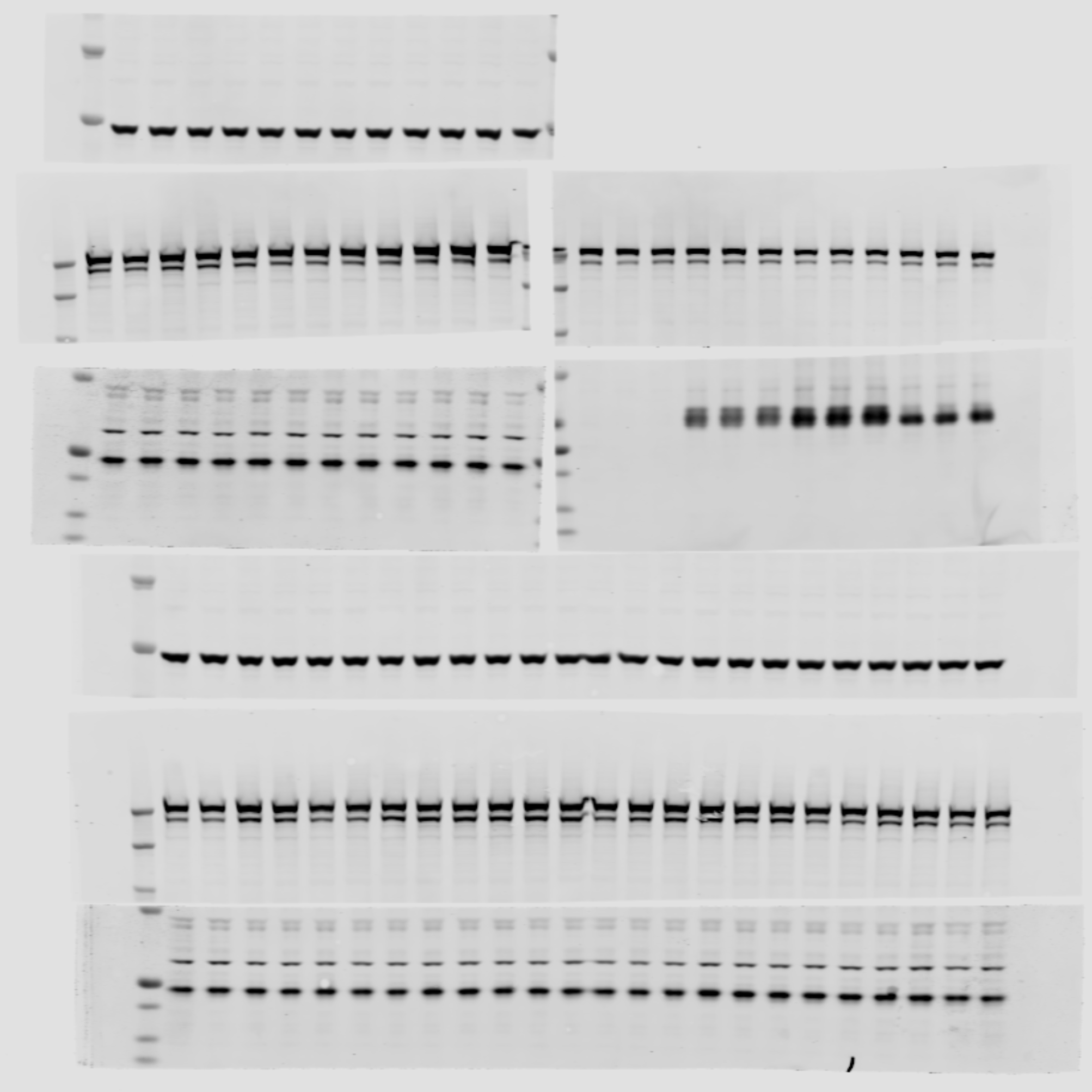

Supplement: Figure 7—figure supplement 1—source data 1. [file elife-87098-fig7-figsupp1-data1.zip › Figure 7-figure supplement 1-source data 1/raw images/Fig7_Suppl1_28-04-2023_700.tif]

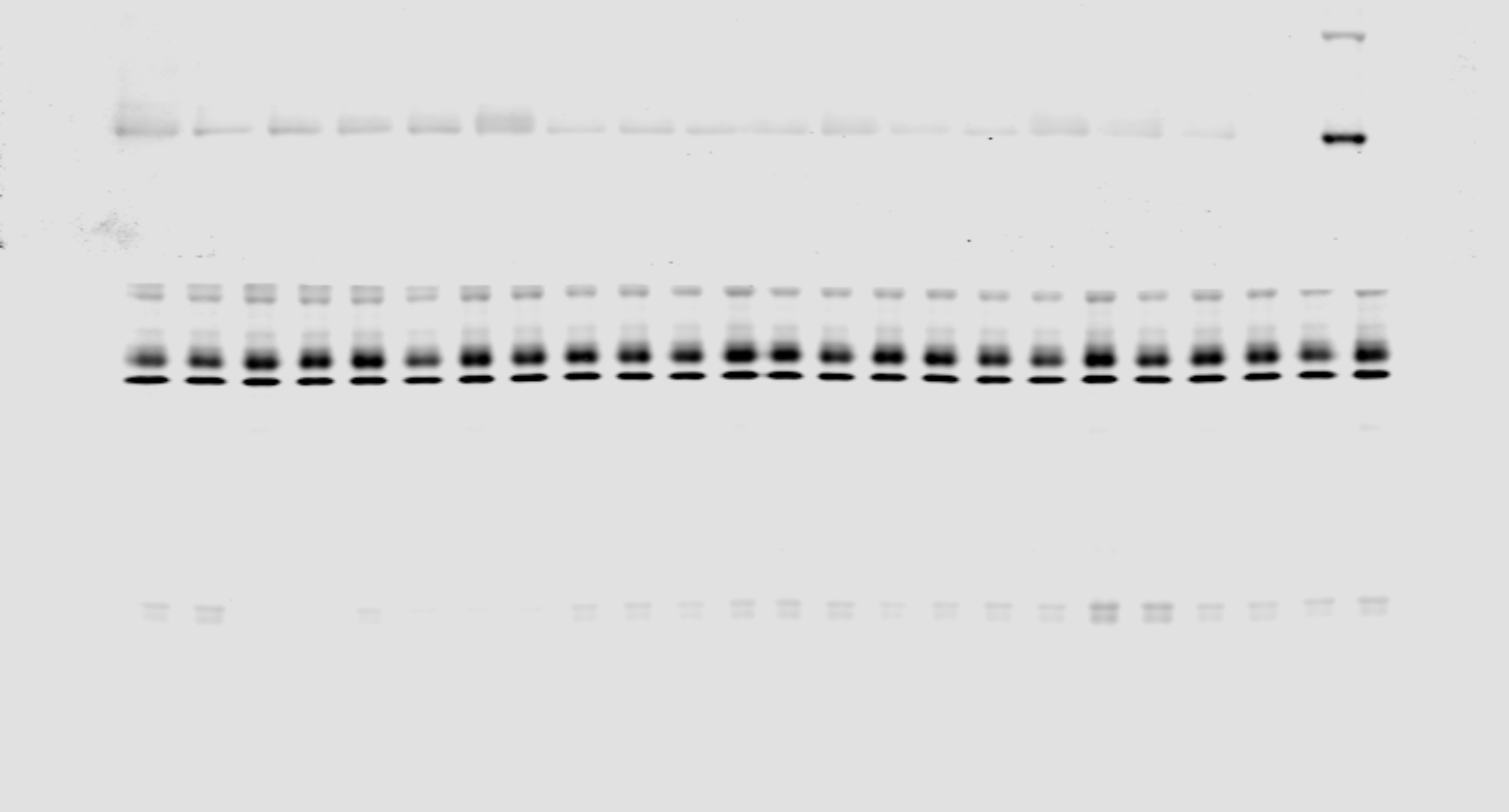

Supplement: Figure 7—figure supplement 1—source data 1. [file elife-87098-fig7-figsupp1-data1.zip › Figure 7-figure supplement 1-source data 1/raw images/Fig7_Suppl1_09-02-23_800-low.tif]

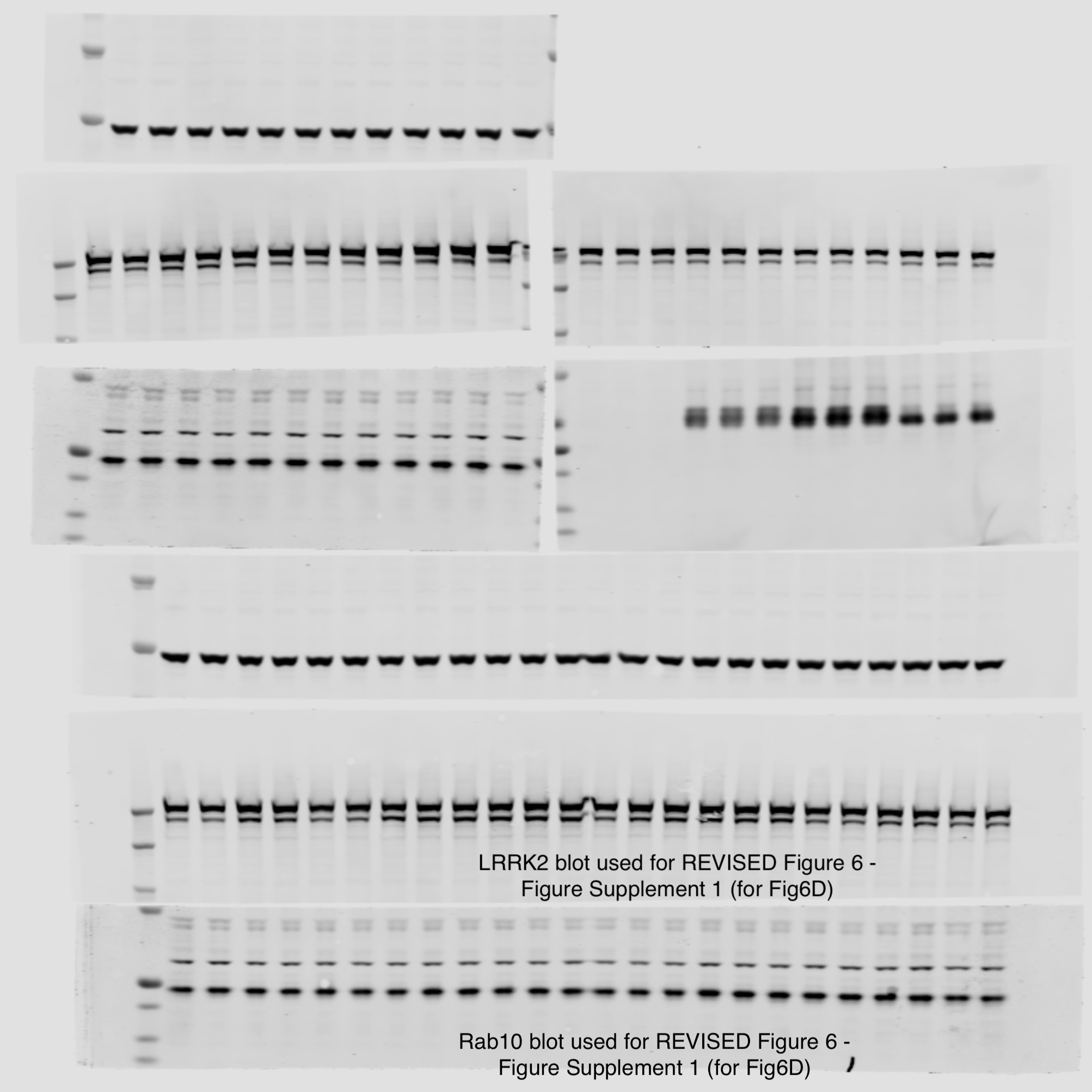

Supplement: Figure 7—figure supplement 1—source data 1. [file elife-87098-fig7-figsupp1-data1.zip › Figure 7-figure supplement 1-source data 1/annotated/REVISED-Fig7-FigSupplement1_28-04-2023_700.tif]

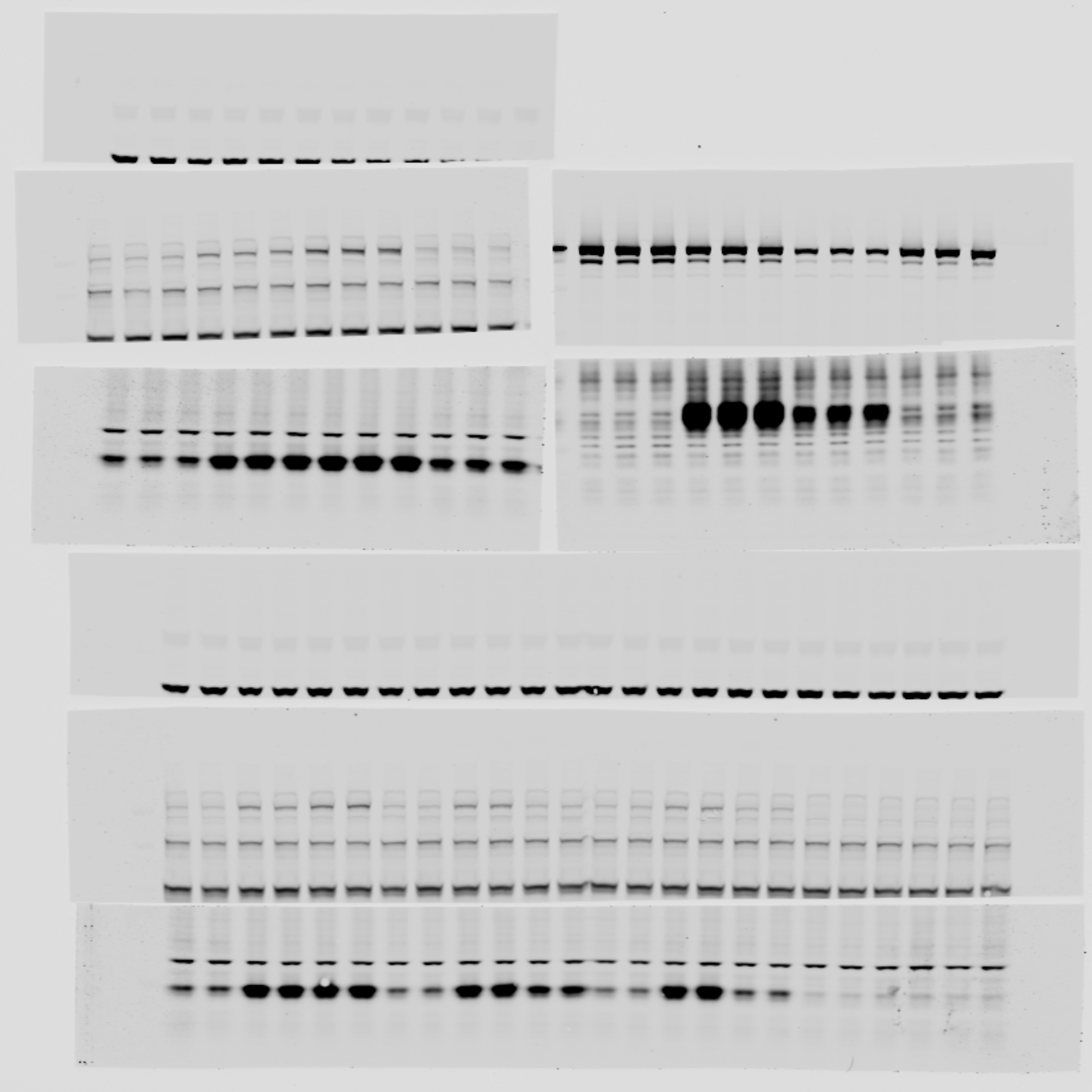

Supplement: Figure 7—figure supplement 1—source data 1. [file elife-87098-fig7-figsupp1-data1.zip › Figure 7-figure supplement 1-source data 1/raw images/Fig7_Suppl1_28-04-2023_800-high.tif]

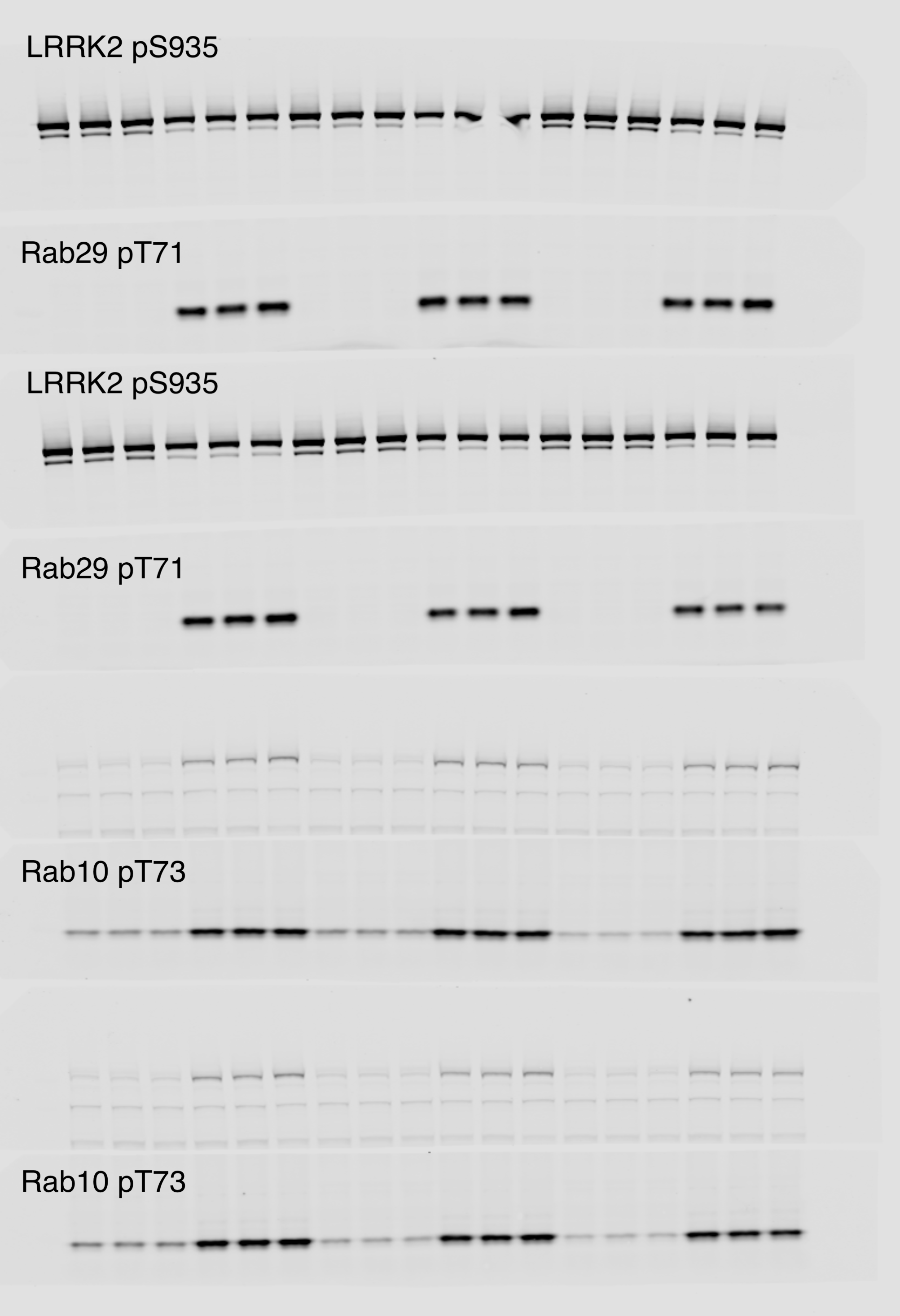

Supplement: Figure 7—figure supplement 1—source data 1. [file elife-87098-fig7-figsupp1-data1.zip › Figure 7-figure supplement 1-source data 1/annotated/Figure 7 Figure Suppl 1 Rab29_800.tif]

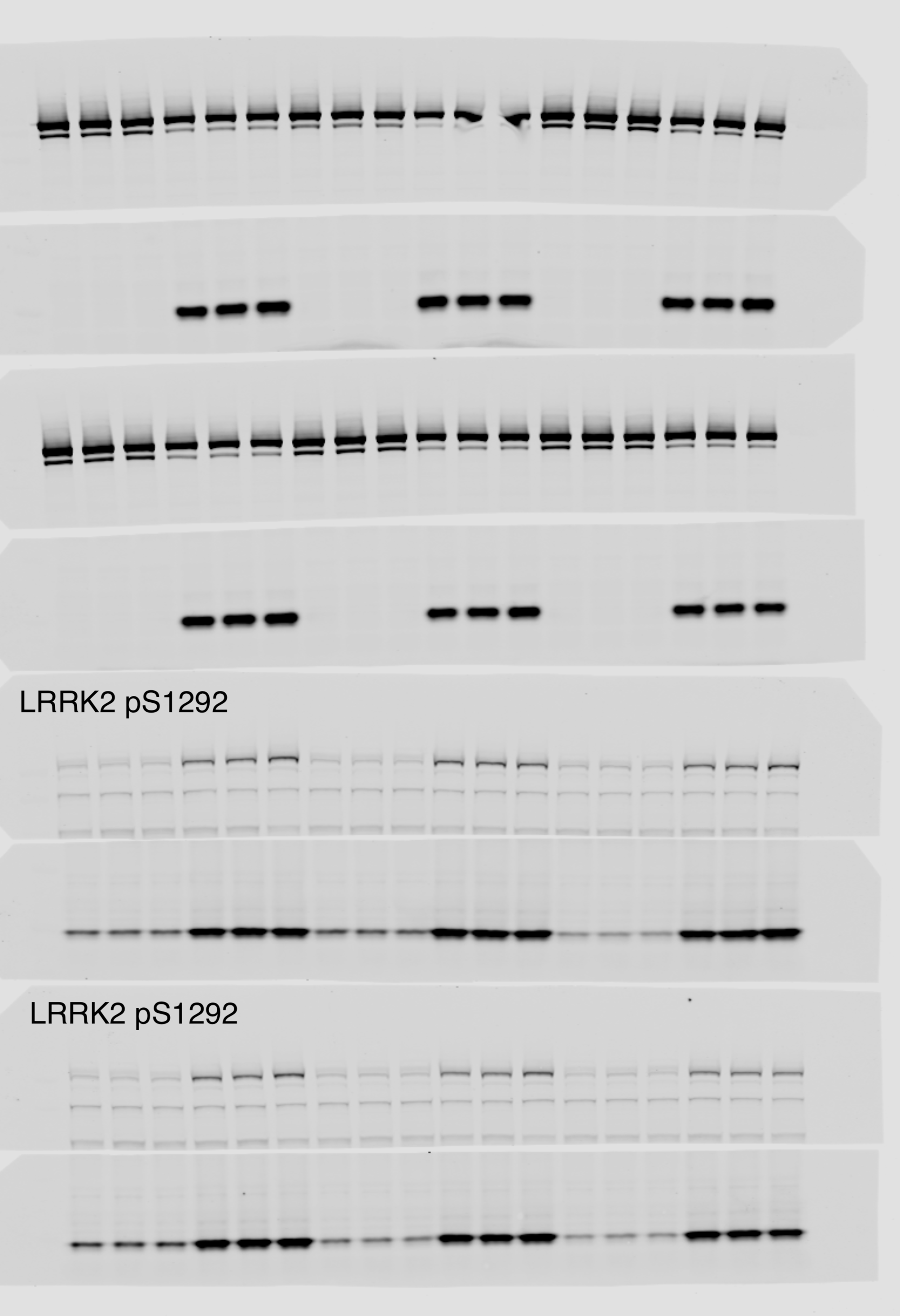

Supplement: Figure 7—figure supplement 1—source data 1. [file elife-87098-fig7-figsupp1-data1.zip › Figure 7-figure supplement 1-source data 1/annotated/Figure 7 Figure Suppl 1 Rab29_800-high.tif]

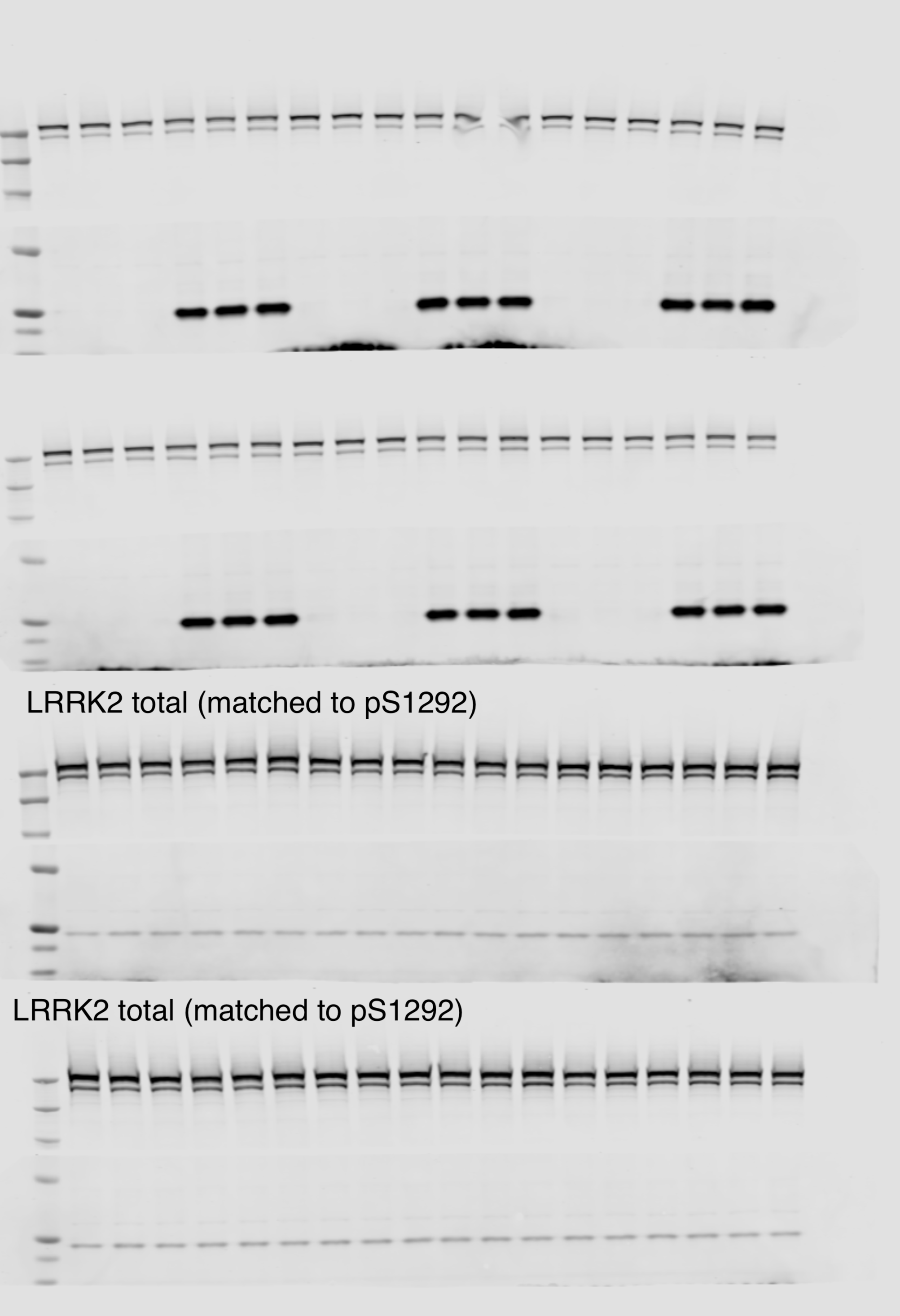

Supplement: Figure 7—figure supplement 1—source data 1. [file elife-87098-fig7-figsupp1-data1.zip › Figure 7-figure supplement 1-source data 1/annotated/Figure 7 Figure Suppl 1 Rab29_700.tif]

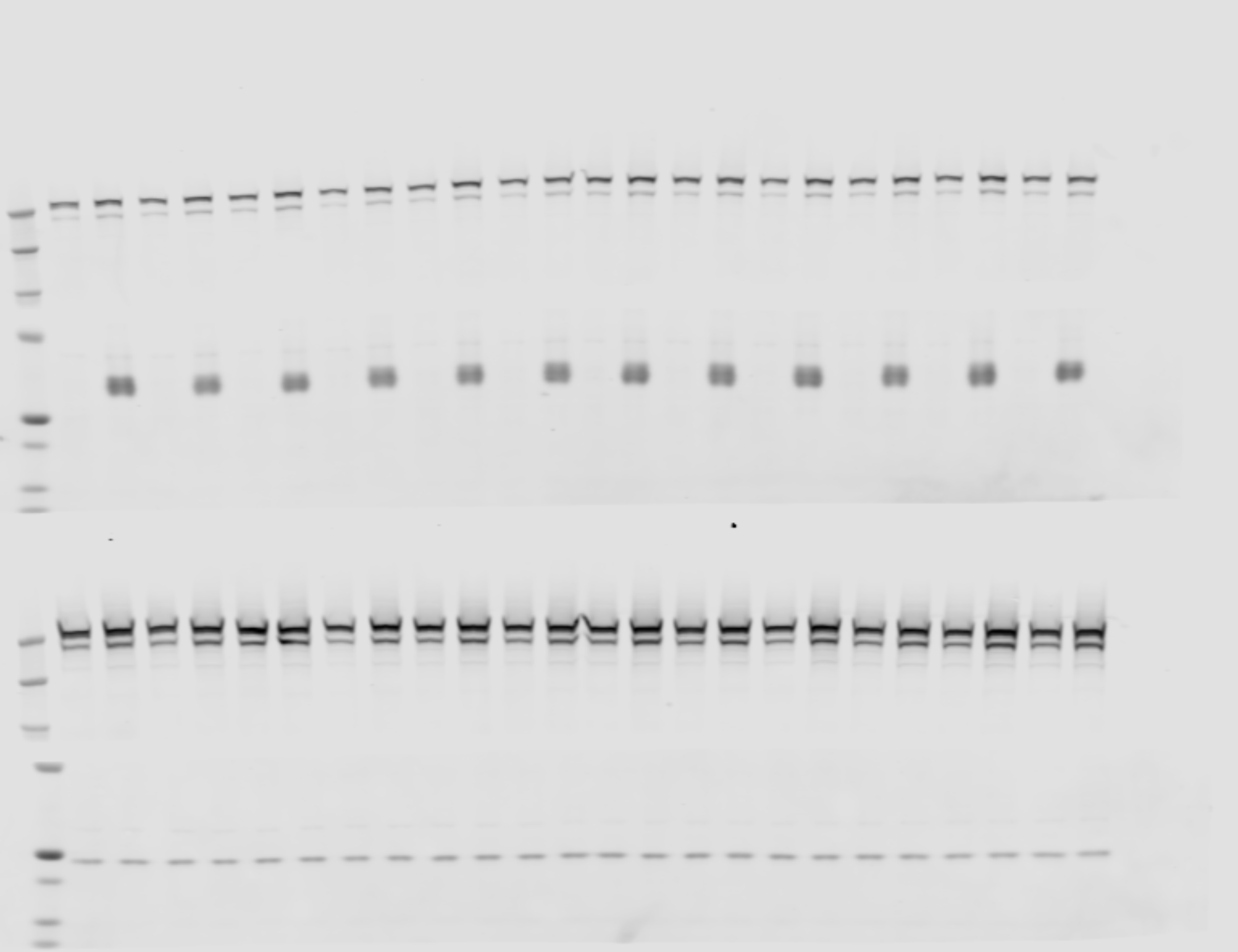

Supplement: Figure 7—figure supplement 1—source data 1. [file elife-87098-fig7-figsupp1-data1.zip › Figure 7-figure supplement 1-source data 1/raw images/Fig7_Suppl1_26-01-23_700-low.tif]

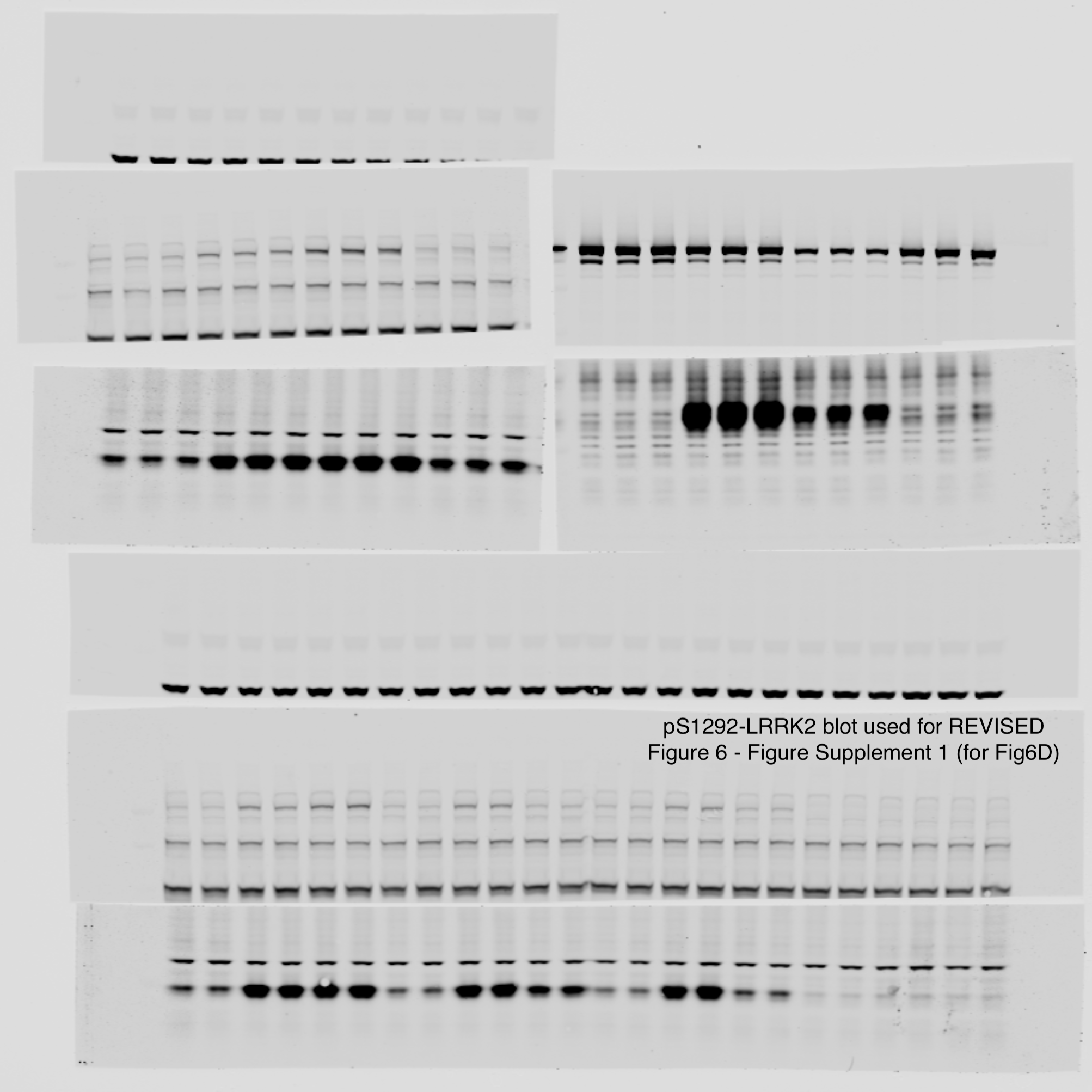

Supplement: Figure 7—figure supplement 1—source data 1. [file elife-87098-fig7-figsupp1-data1.zip › Figure 7-figure supplement 1-source data 1/annotated/REVISED-Fig7-FigSupplement1_28-04-2023_800-high.tif]

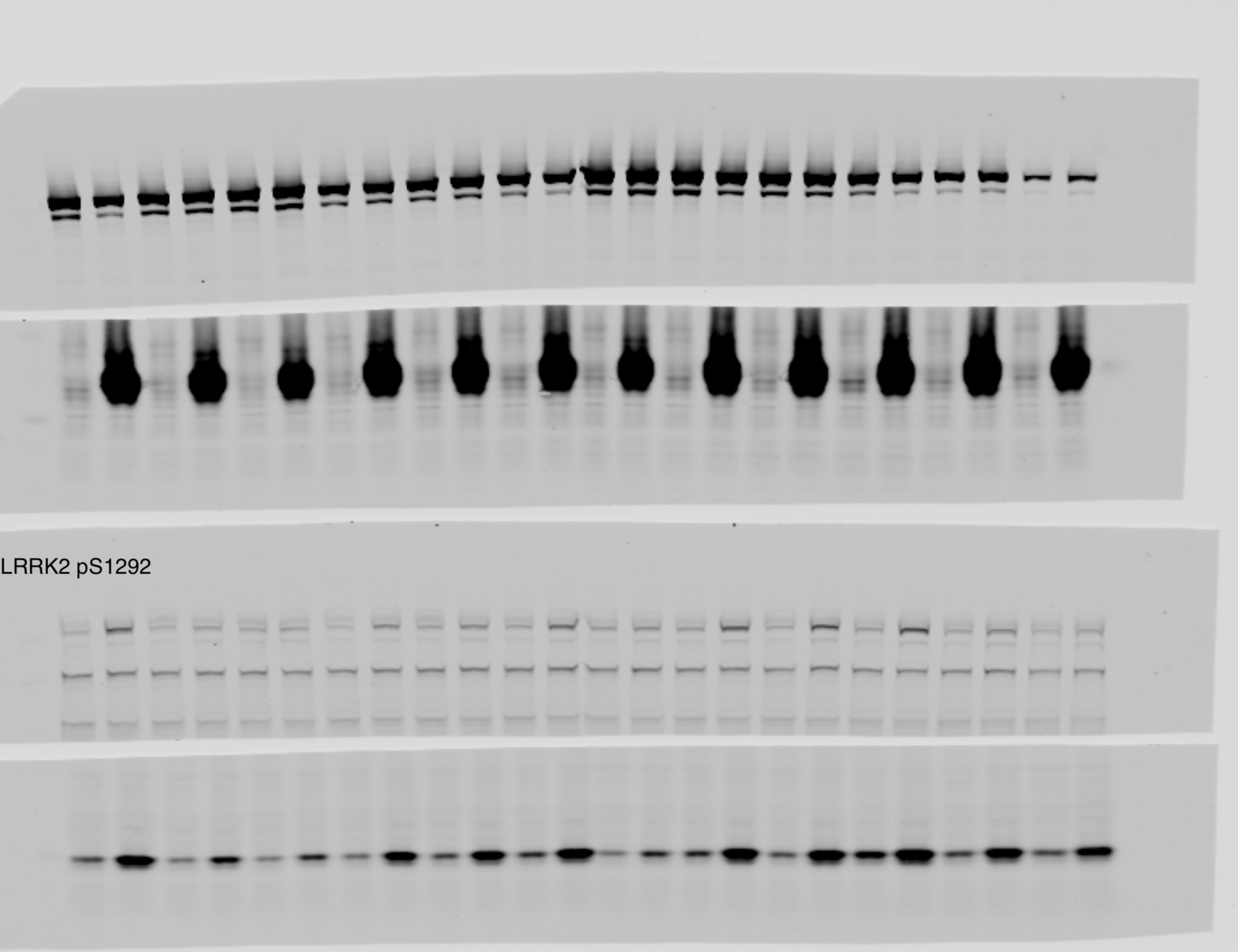

Supplement: Figure 7—figure supplement 1—source data 1. [file elife-87098-fig7-figsupp1-data1.zip › Figure 7-figure supplement 1-source data 1/annotated/Figure 7 Figure Suppl 1 Rab12_800-high.tif]

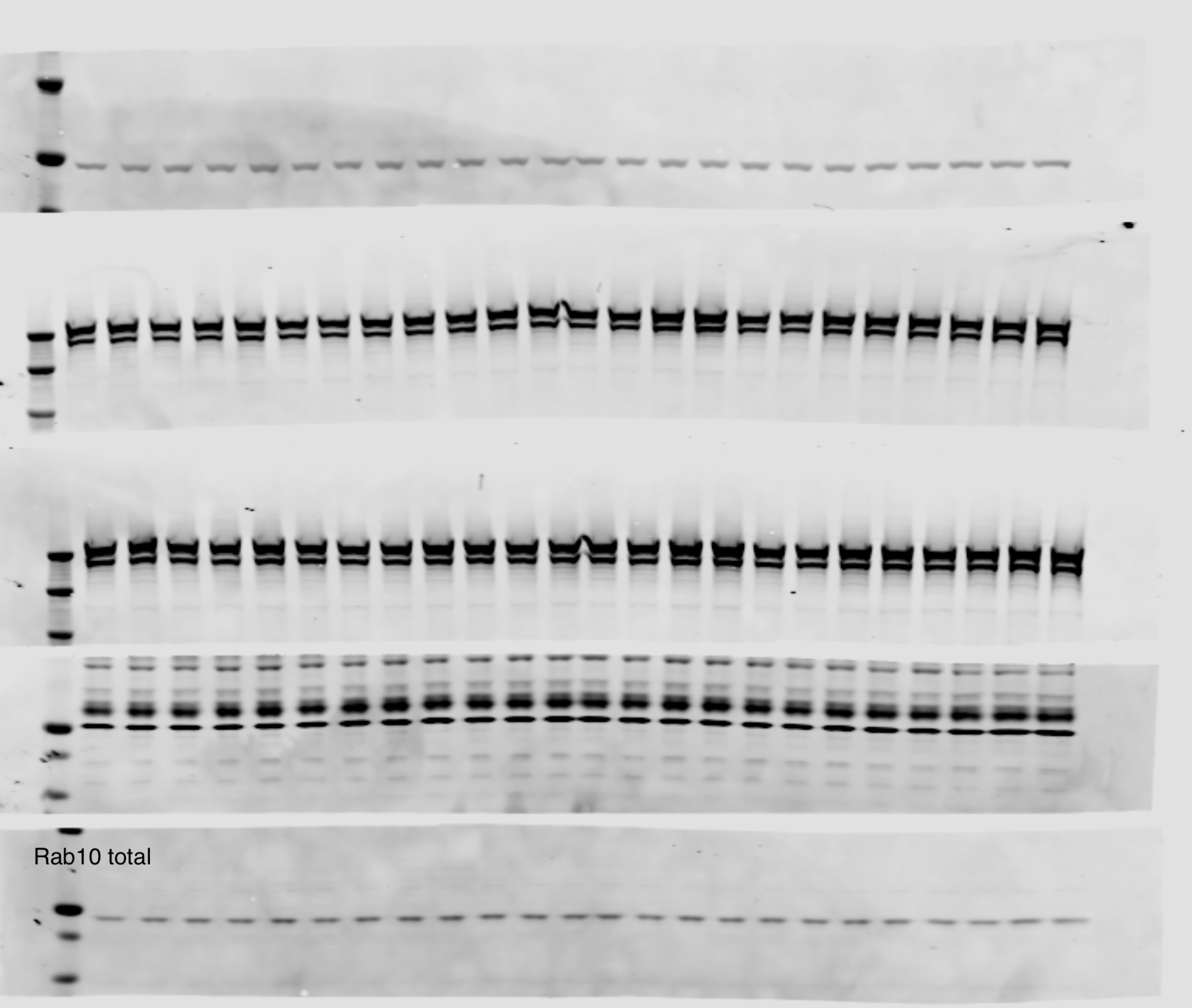

Supplement: Figure 7—figure supplement 1—source data 1. [file elife-87098-fig7-figsupp1-data1.zip › Figure 7-figure supplement 1-source data 1/annotated/Figure 7 Figure Suppl 1_700.tif]

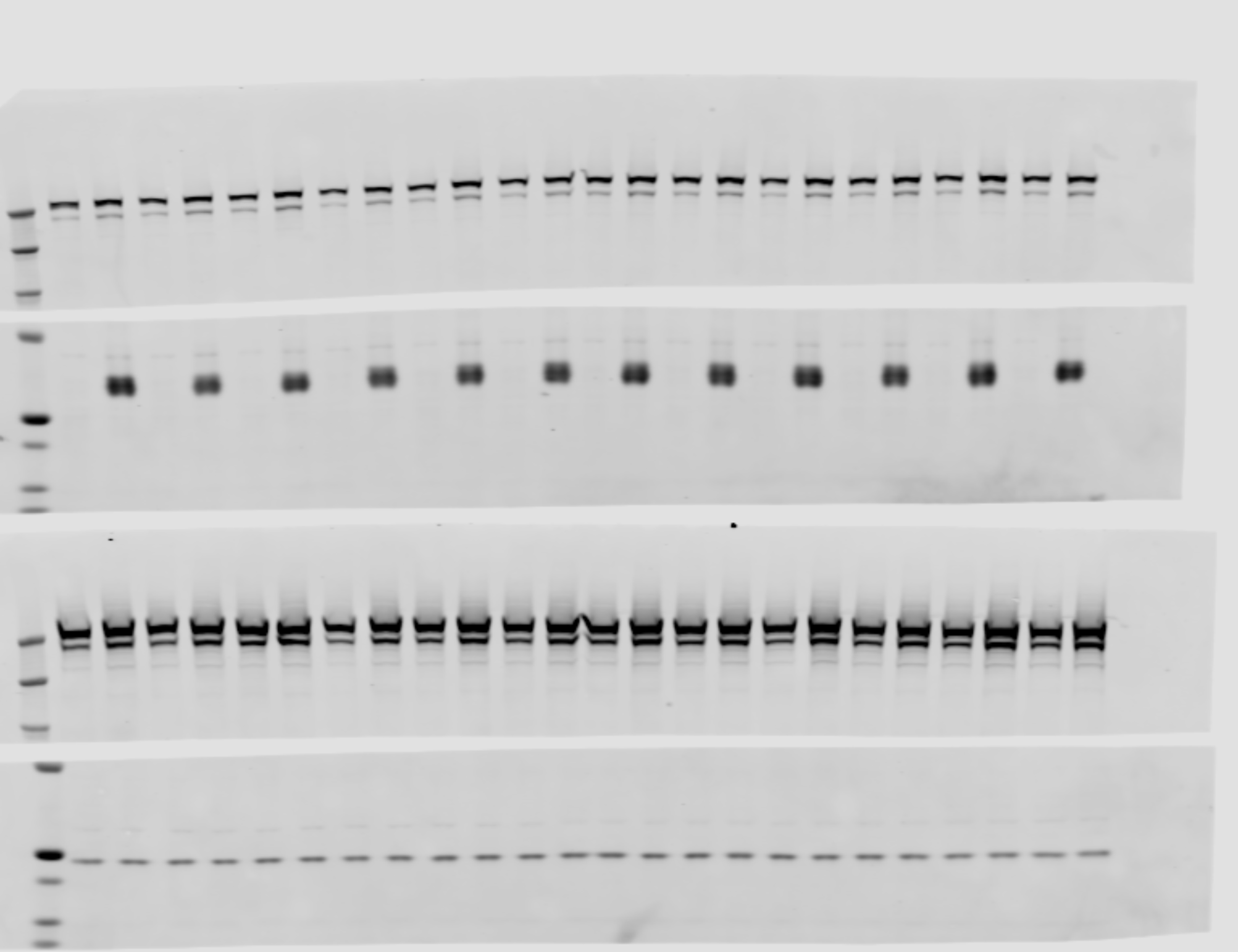

Supplement: Figure 7—figure supplement 1—source data 1. [file elife-87098-fig7-figsupp1-data1.zip › Figure 7-figure supplement 1-source data 1/raw images/Fig7_Suppl1_26-01-23_700.tif]

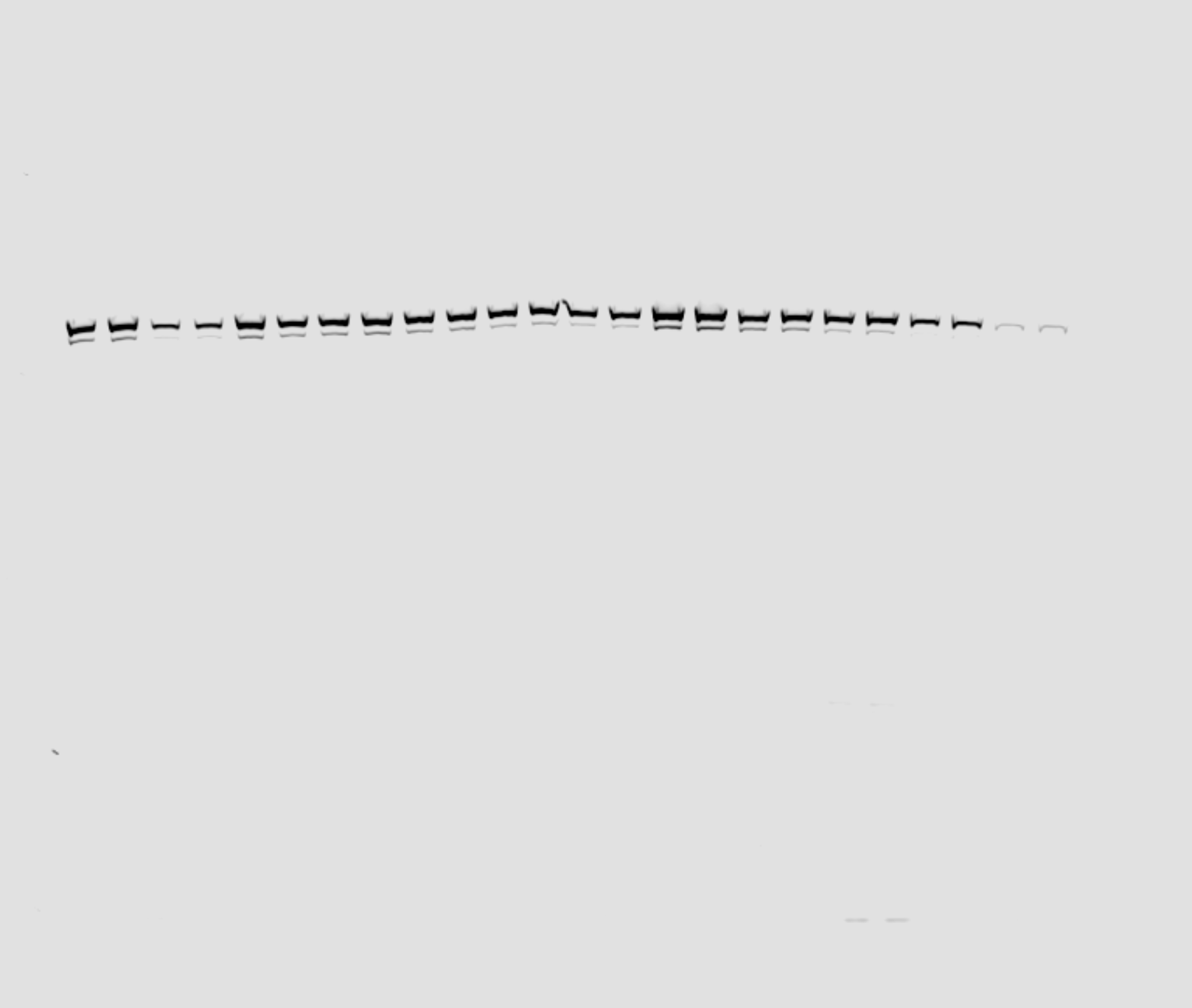

Supplement: Figure 7—figure supplement 1—source data 1. [file elife-87098-fig7-figsupp1-data1.zip › Figure 7-figure supplement 1-source data 1/raw images/Fig7_Suppl1_27-09-22_800-low.tif]

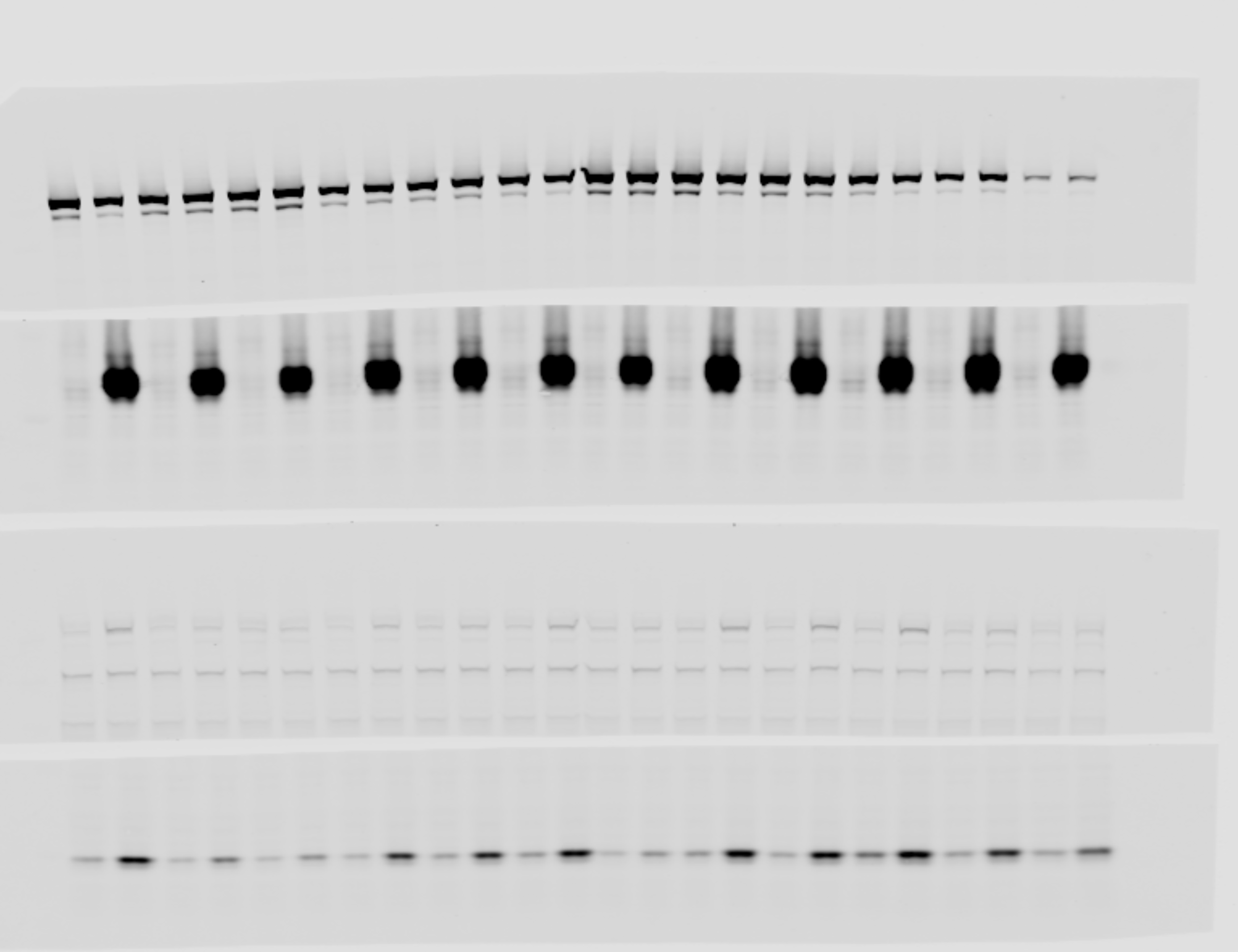

Supplement: Figure 7—figure supplement 1—source data 1. [file elife-87098-fig7-figsupp1-data1.zip › Figure 7-figure supplement 1-source data 1/raw images/Fig7_Suppl1_26-01-23_800.tif]

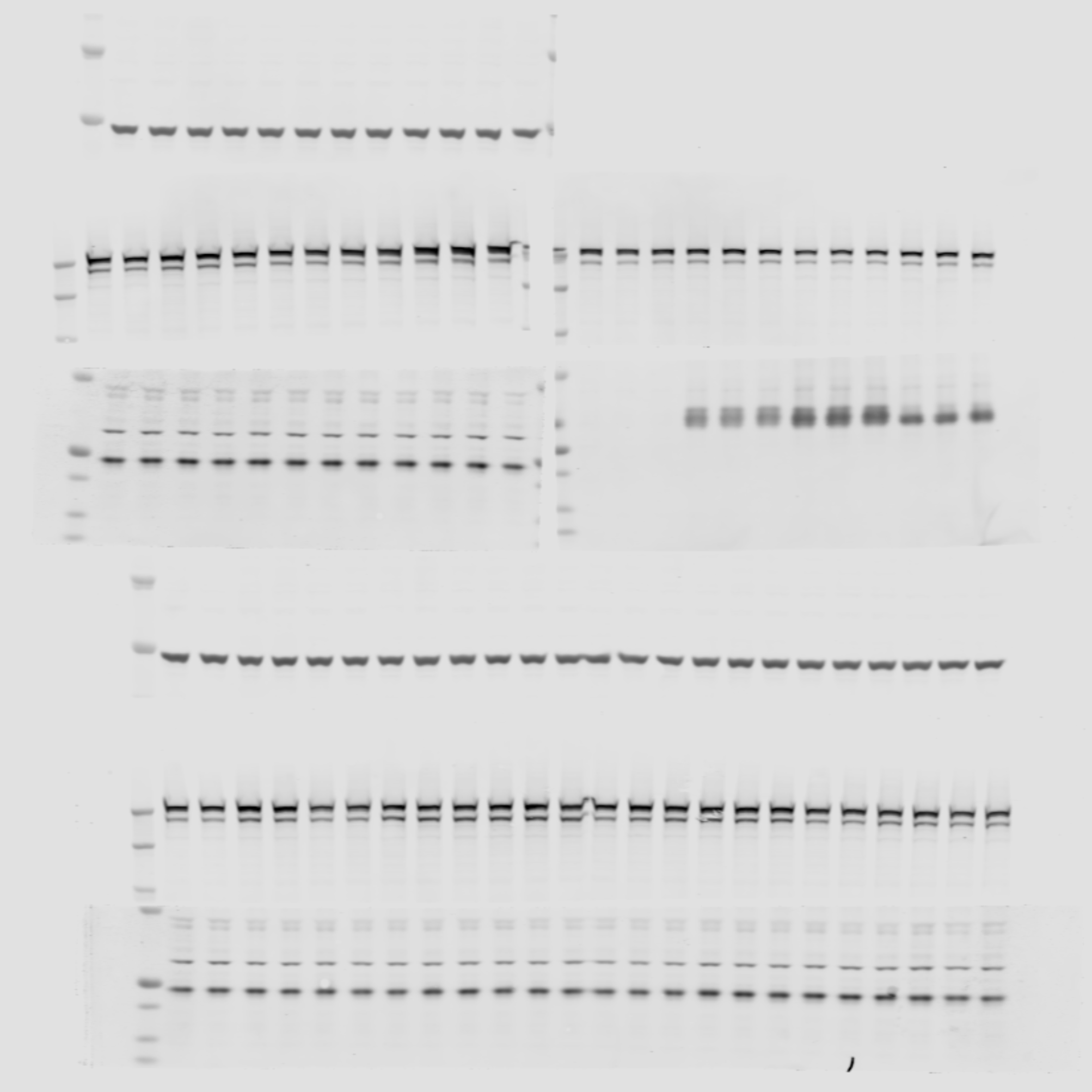

Supplement: Figure 7—figure supplement 1—source data 1. [file elife-87098-fig7-figsupp1-data1.zip › Figure 7-figure supplement 1-source data 1/raw images/Fig7_Suppl1_28-04-2023_700-low.tif]

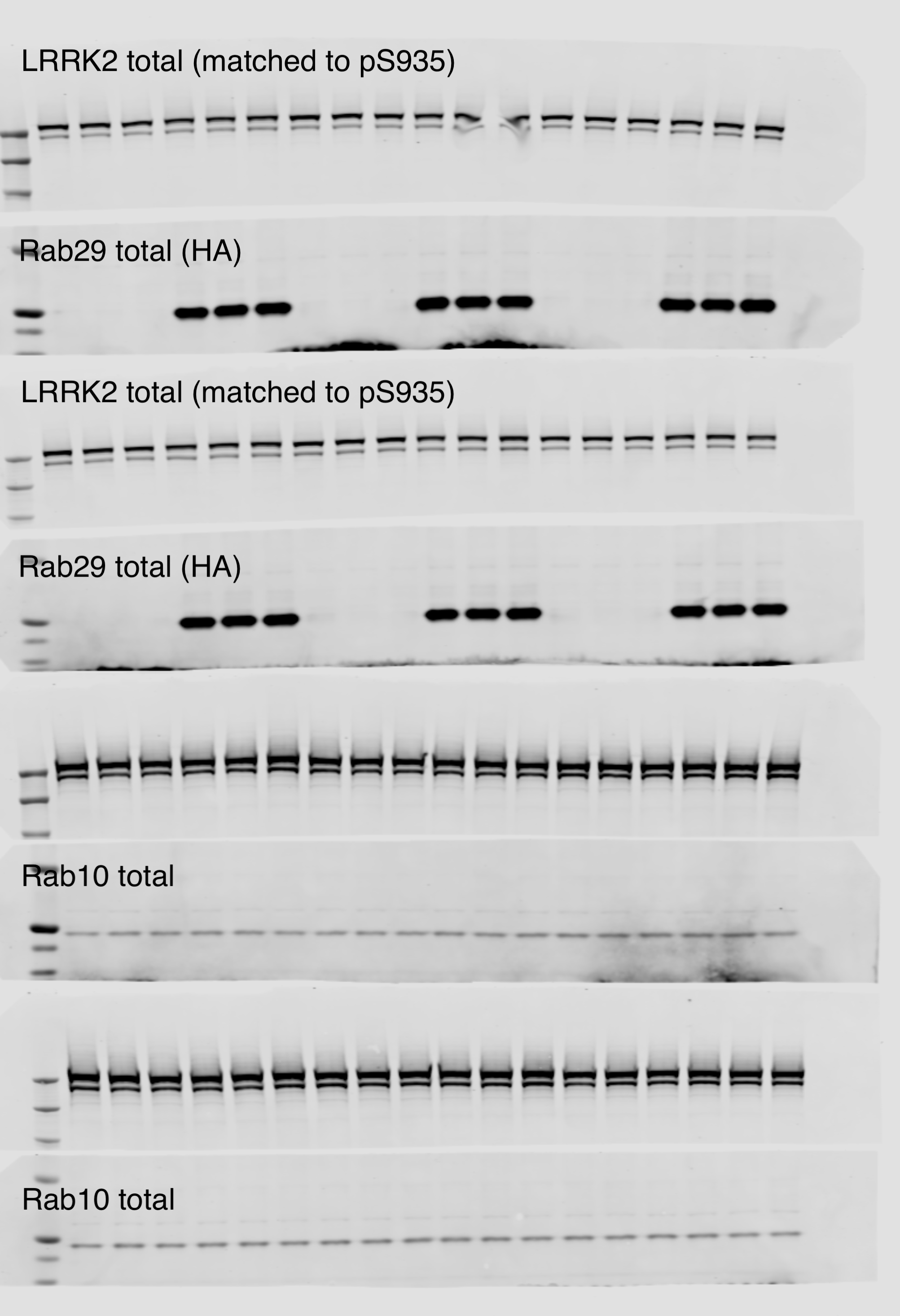

Supplement: Figure 7—figure supplement 1—source data 1. [file elife-87098-fig7-figsupp1-data1.zip › Figure 7-figure supplement 1-source data 1/annotated/Figure 7 Figure Suppl 1 Rab29_700-high.tif]

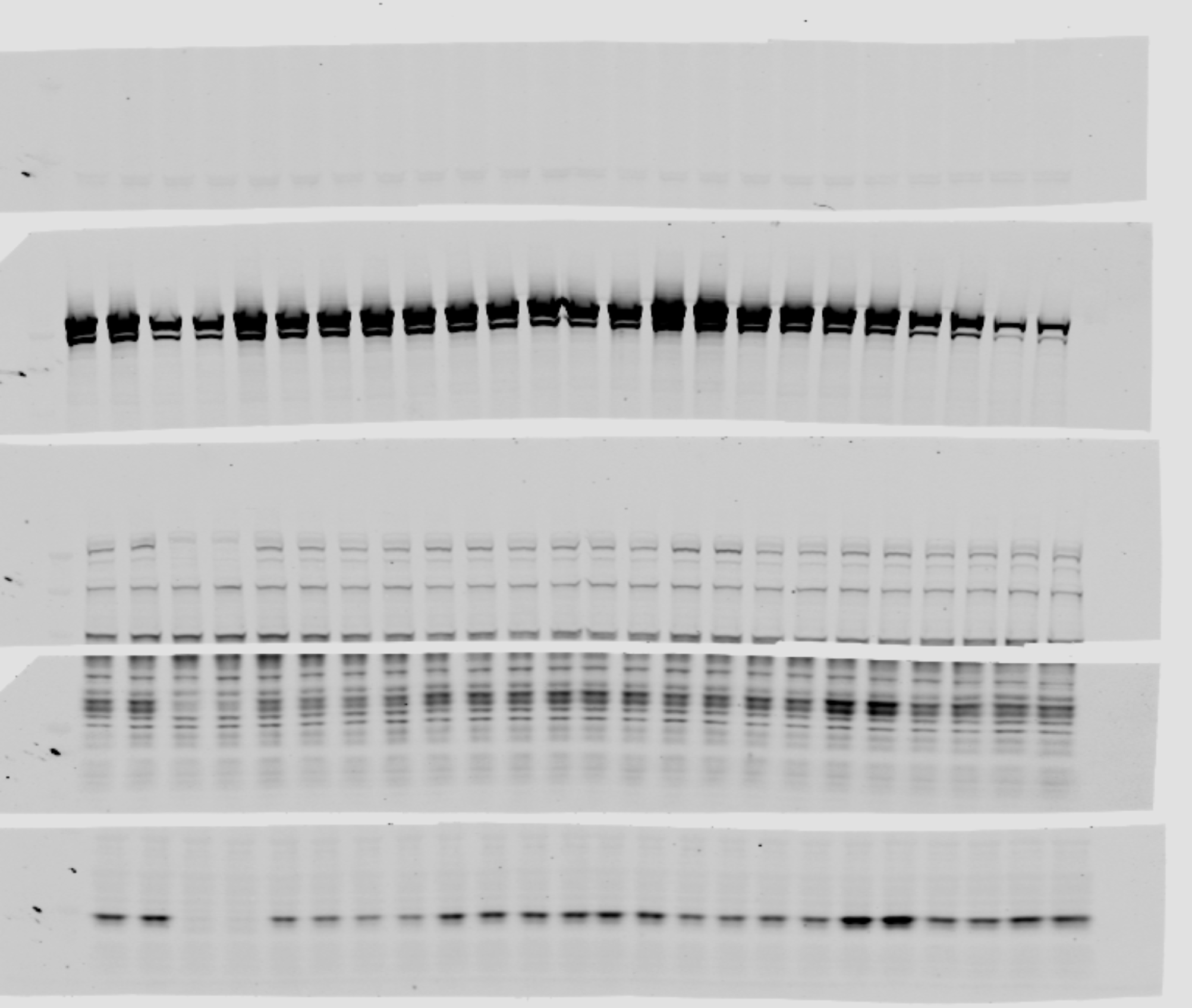

Supplement: Figure 7—figure supplement 1—source data 1. [file elife-87098-fig7-figsupp1-data1.zip › Figure 7-figure supplement 1-source data 1/raw images/Fig7_Suppl1_27-09-22_800.tif]

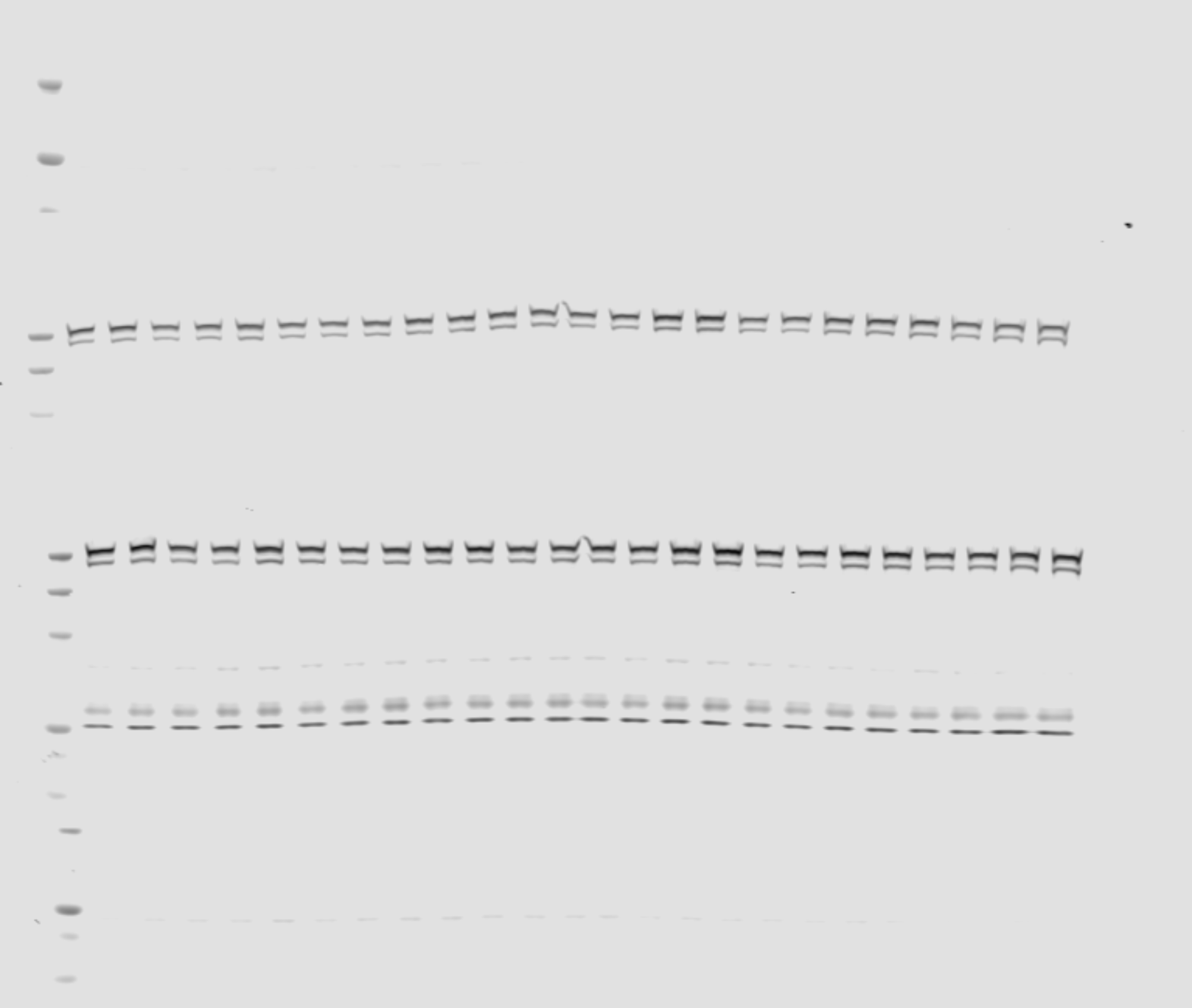

Supplement: Figure 7—figure supplement 1—source data 1. [file elife-87098-fig7-figsupp1-data1.zip › Figure 7-figure supplement 1-source data 1/raw images/Fig7_Suppl1_27-09-22_700-low.tif]

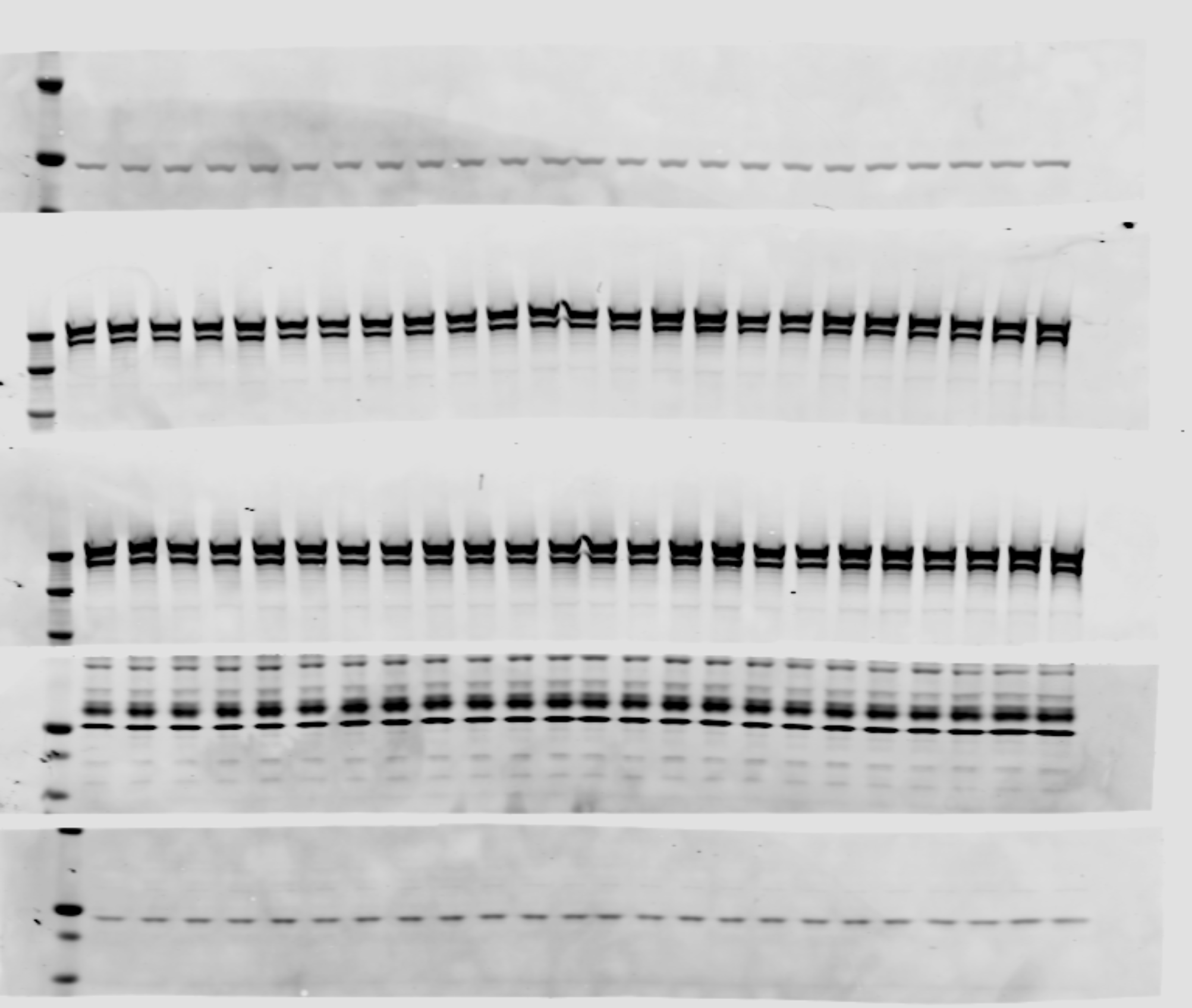

Supplement: Figure 7—figure supplement 1—source data 1. [file elife-87098-fig7-figsupp1-data1.zip › Figure 7-figure supplement 1-source data 1/raw images/Fig7_Suppl1_27-09-22_700.tif]

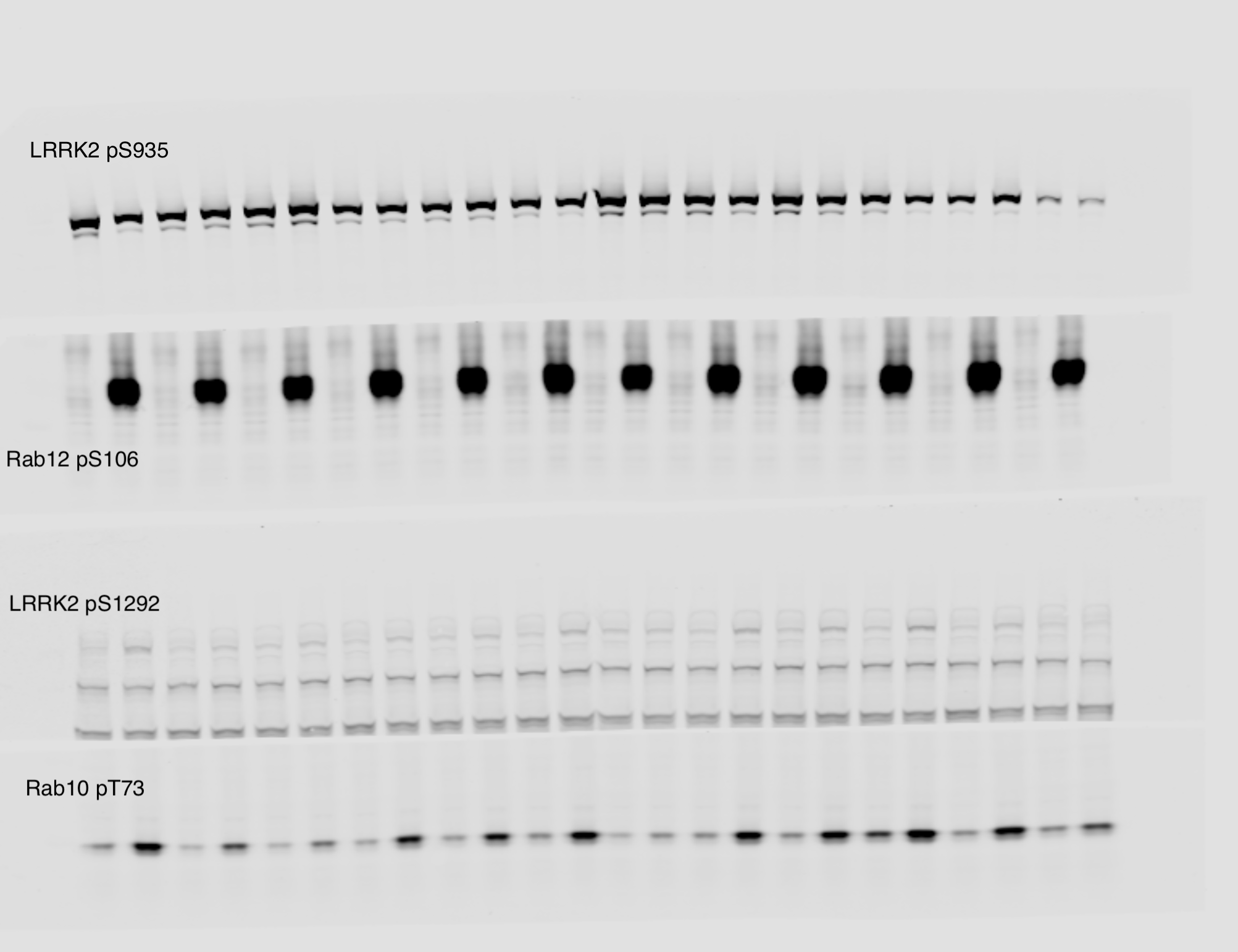

Supplement: Figure 7—figure supplement 1—source data 1. [file elife-87098-fig7-figsupp1-data1.zip › Figure 7-figure supplement 1-source data 1/annotated/Additional blots quantified in Figure 7C_800.tif]

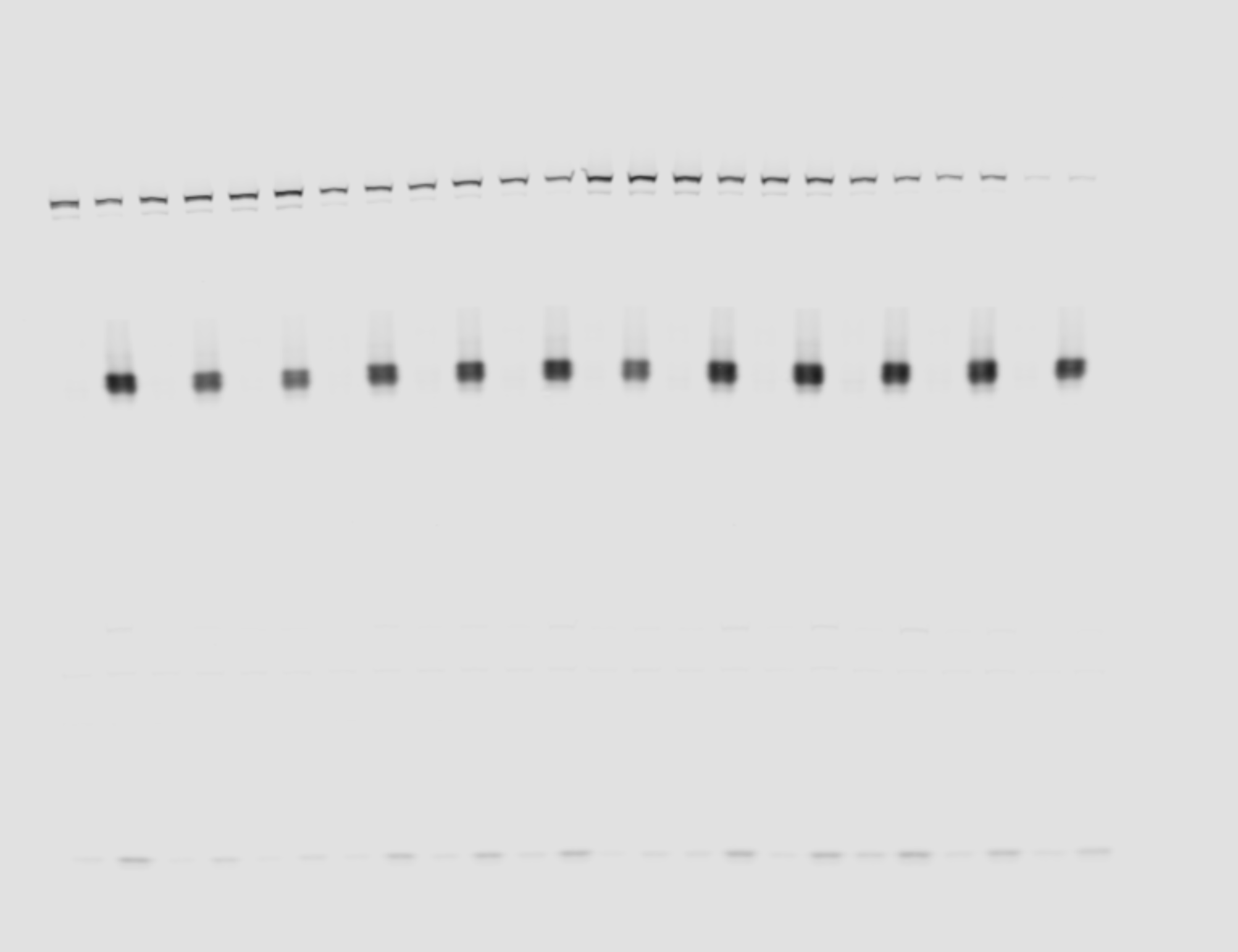

Supplement: Figure 7—figure supplement 1—source data 1. [file elife-87098-fig7-figsupp1-data1.zip › Figure 7-figure supplement 1-source data 1/raw images/Fig7_Suppl1_26-01-23_800-low.tif]

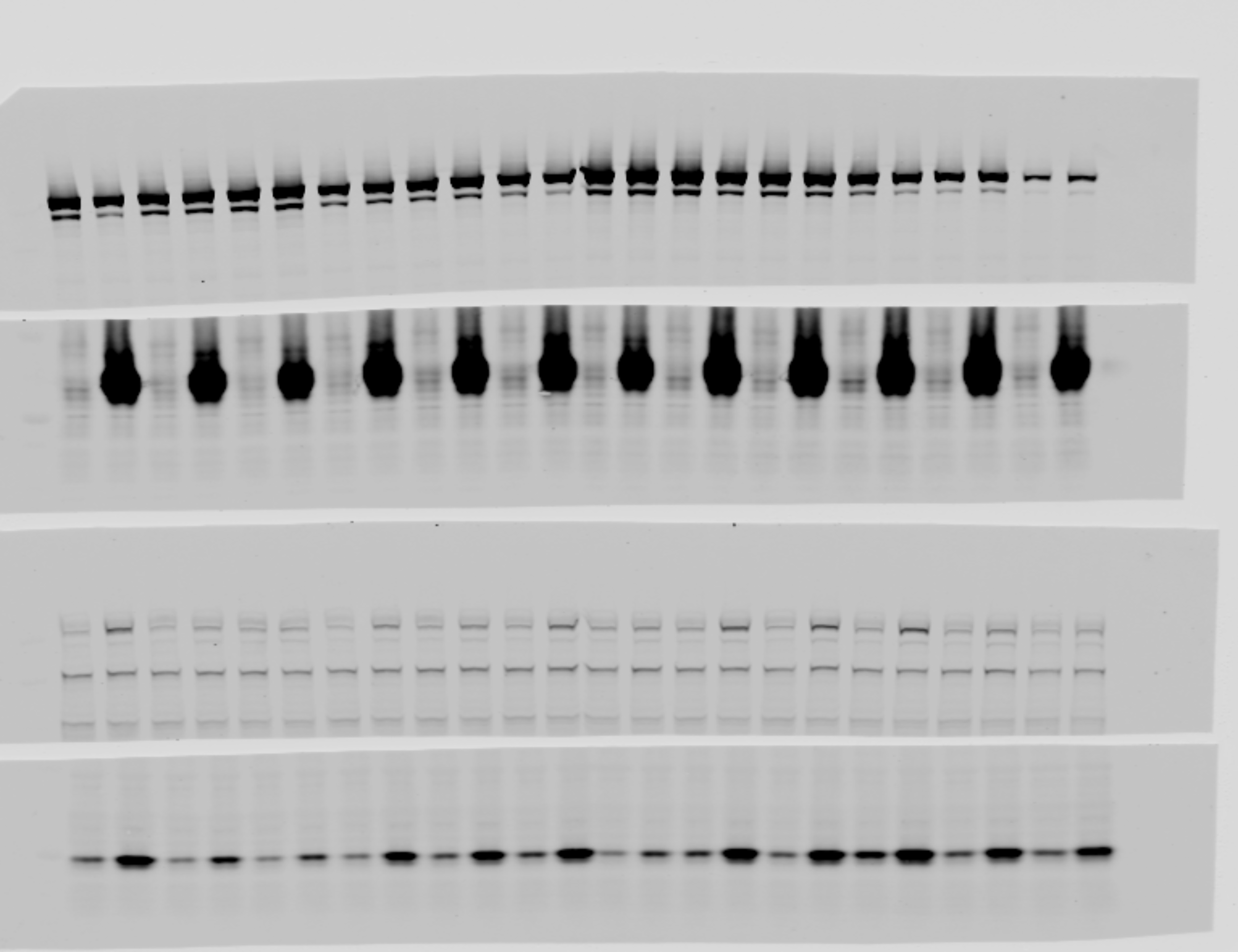

Supplement: Figure 7—figure supplement 1—source data 1. [file elife-87098-fig7-figsupp1-data1.zip › Figure 7-figure supplement 1-source data 1/raw images/Fig7_Suppl1_26-01-23_800-high.tif]

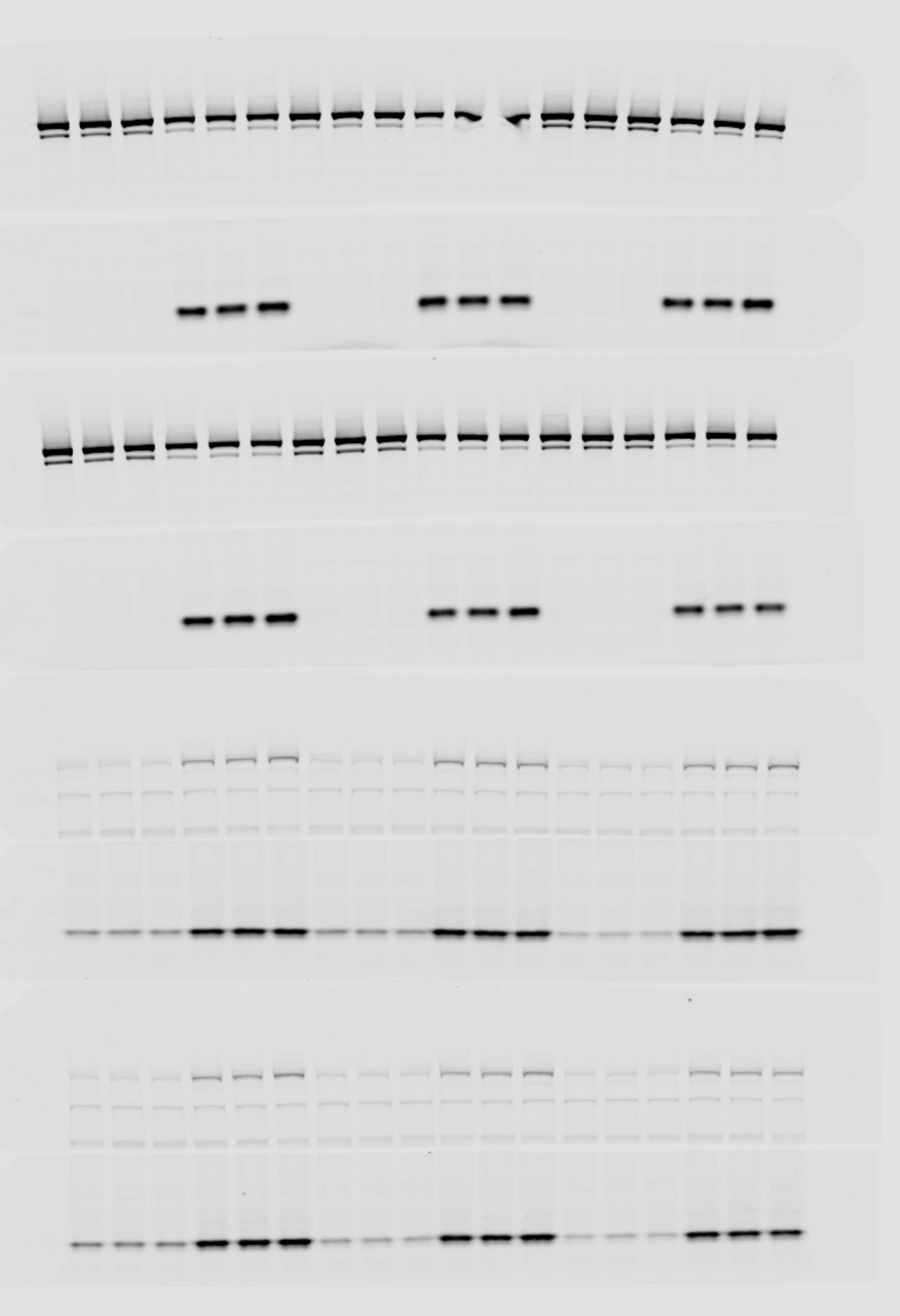

Supplement: Figure 7—figure supplement 1—source data 1. [file elife-87098-fig7-figsupp1-data1.zip › Figure 7-figure supplement 1-source data 1/raw images/Fig7_Suppl1_22-12-22_800.tif]

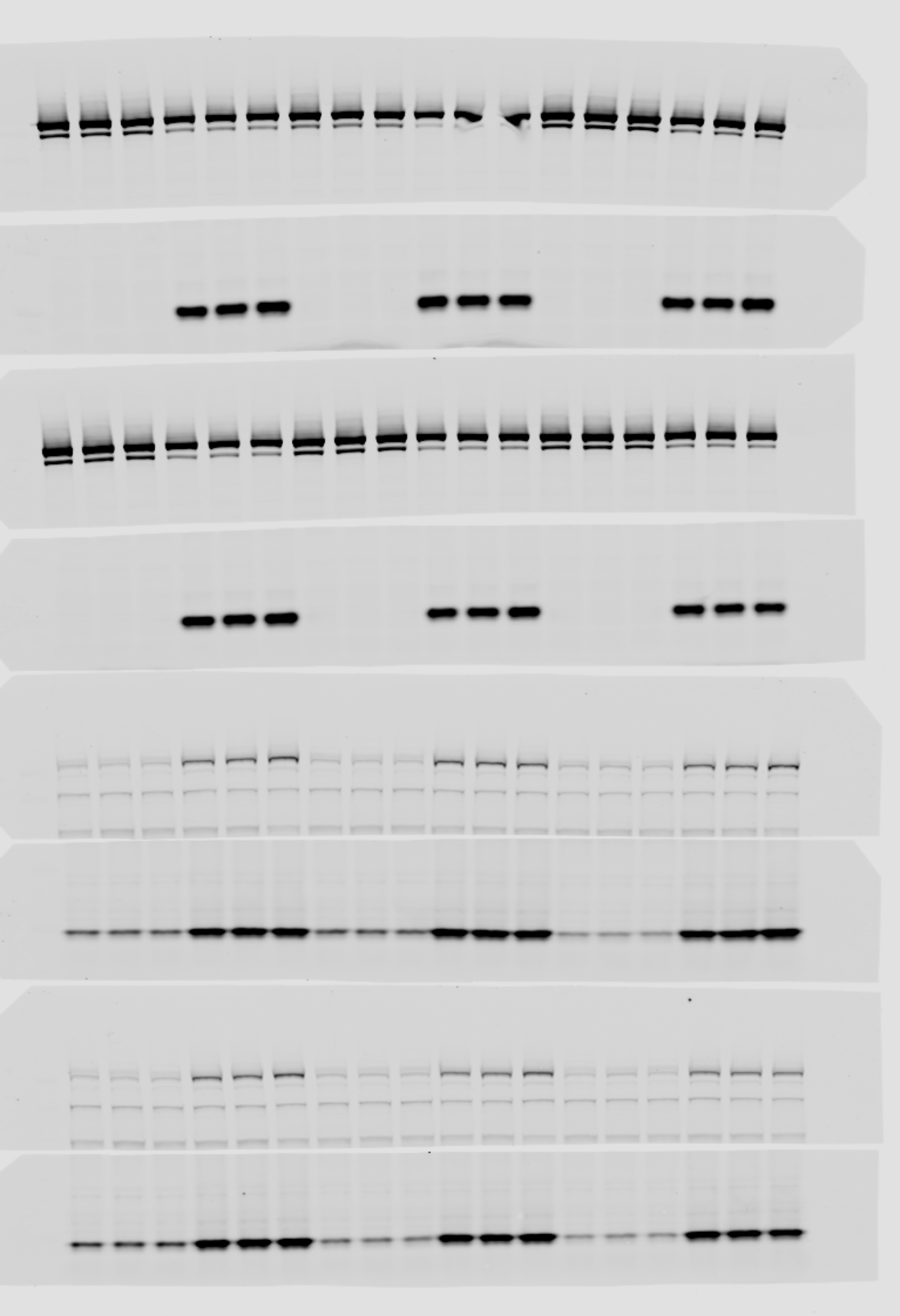

Supplement: Figure 7—figure supplement 1—source data 1. [file elife-87098-fig7-figsupp1-data1.zip › Figure 7-figure supplement 1-source data 1/raw images/Fig7_Suppl1_22-12-22_800-high.tif]

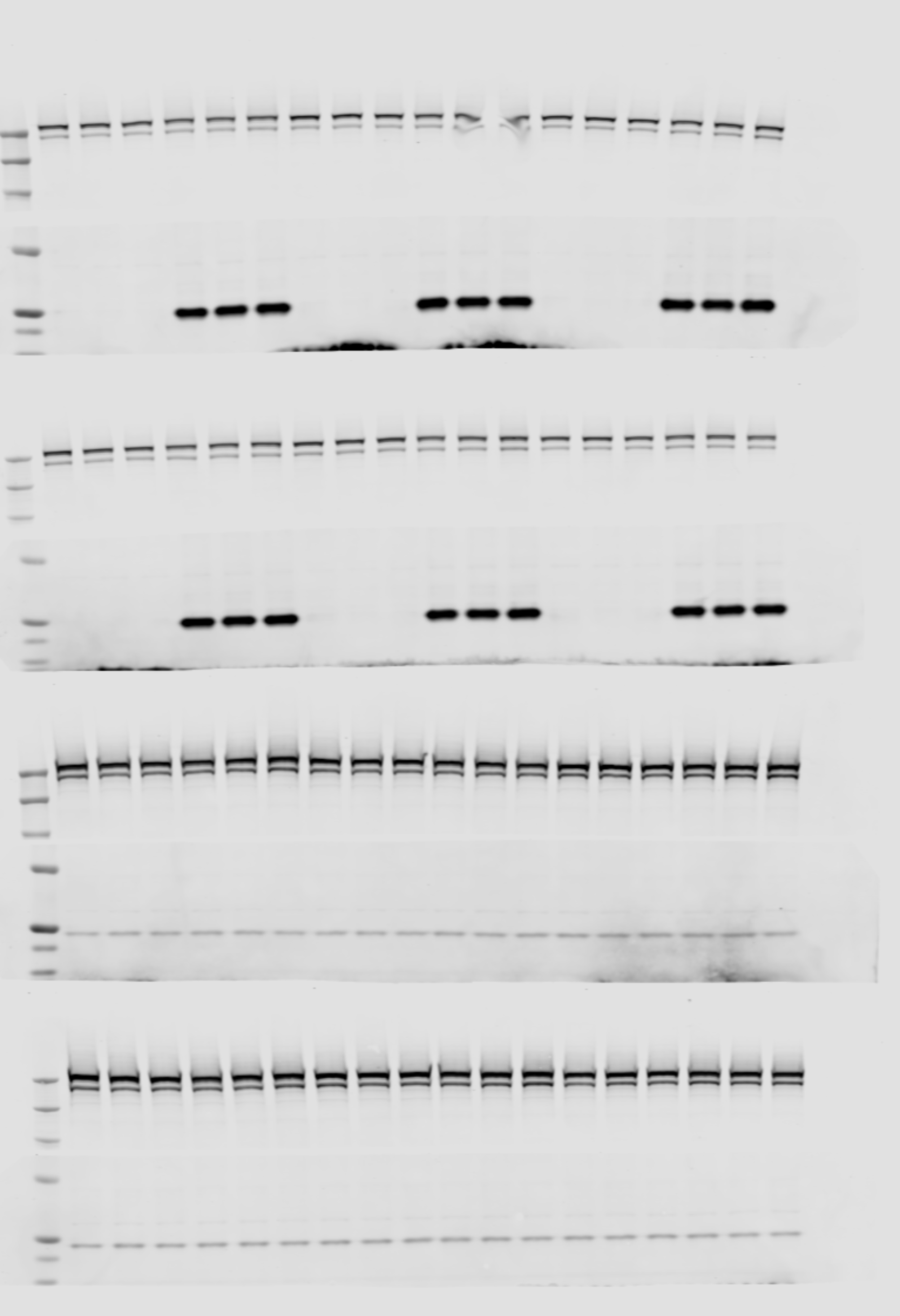

Supplement: Figure 7—figure supplement 1—source data 1. [file elife-87098-fig7-figsupp1-data1.zip › Figure 7-figure supplement 1-source data 1/raw images/Fig7_Suppl1_22-12-22_700.tif]

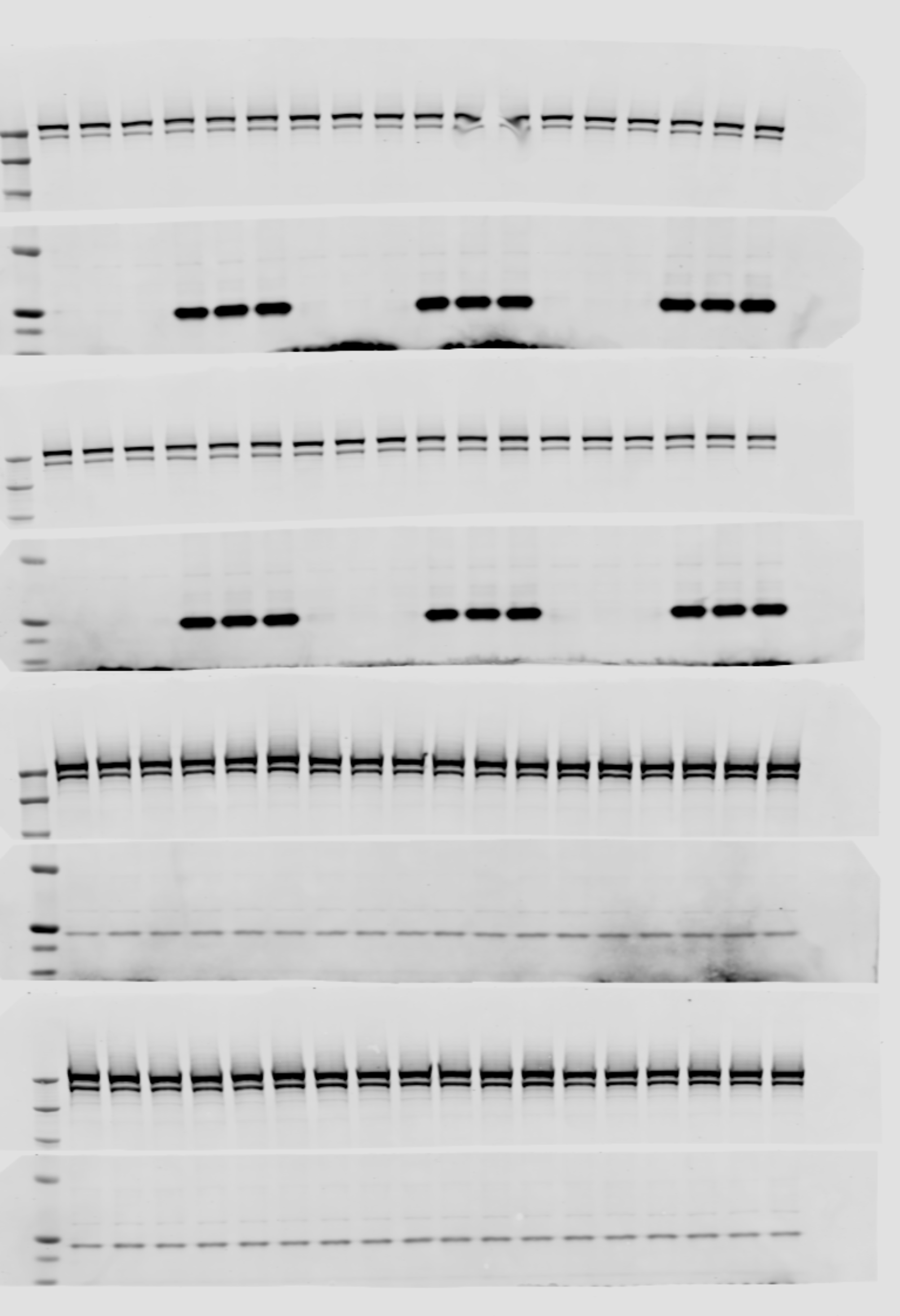

Supplement: Figure 7—figure supplement 1—source data 1. [file elife-87098-fig7-figsupp1-data1.zip › Figure 7-figure supplement 1-source data 1/raw images/Fig7_Suppl1_22-12-22_700-high.tif]

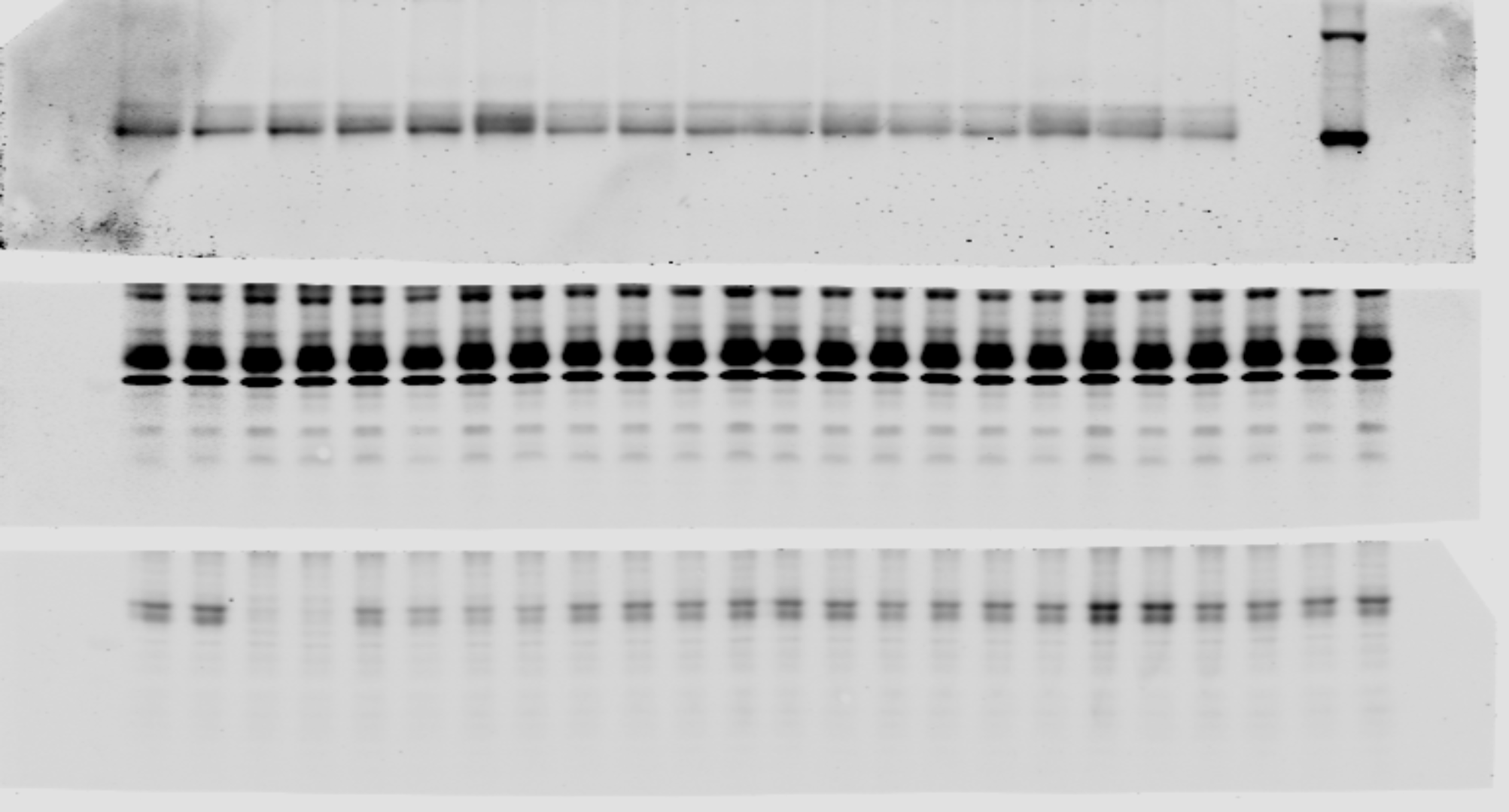

Supplement: Figure 7—figure supplement 1—source data 1. [file elife-87098-fig7-figsupp1-data1.zip › Figure 7-figure supplement 1-source data 1/raw images/Fig7_Suppl1_09-02-23_800.tif]

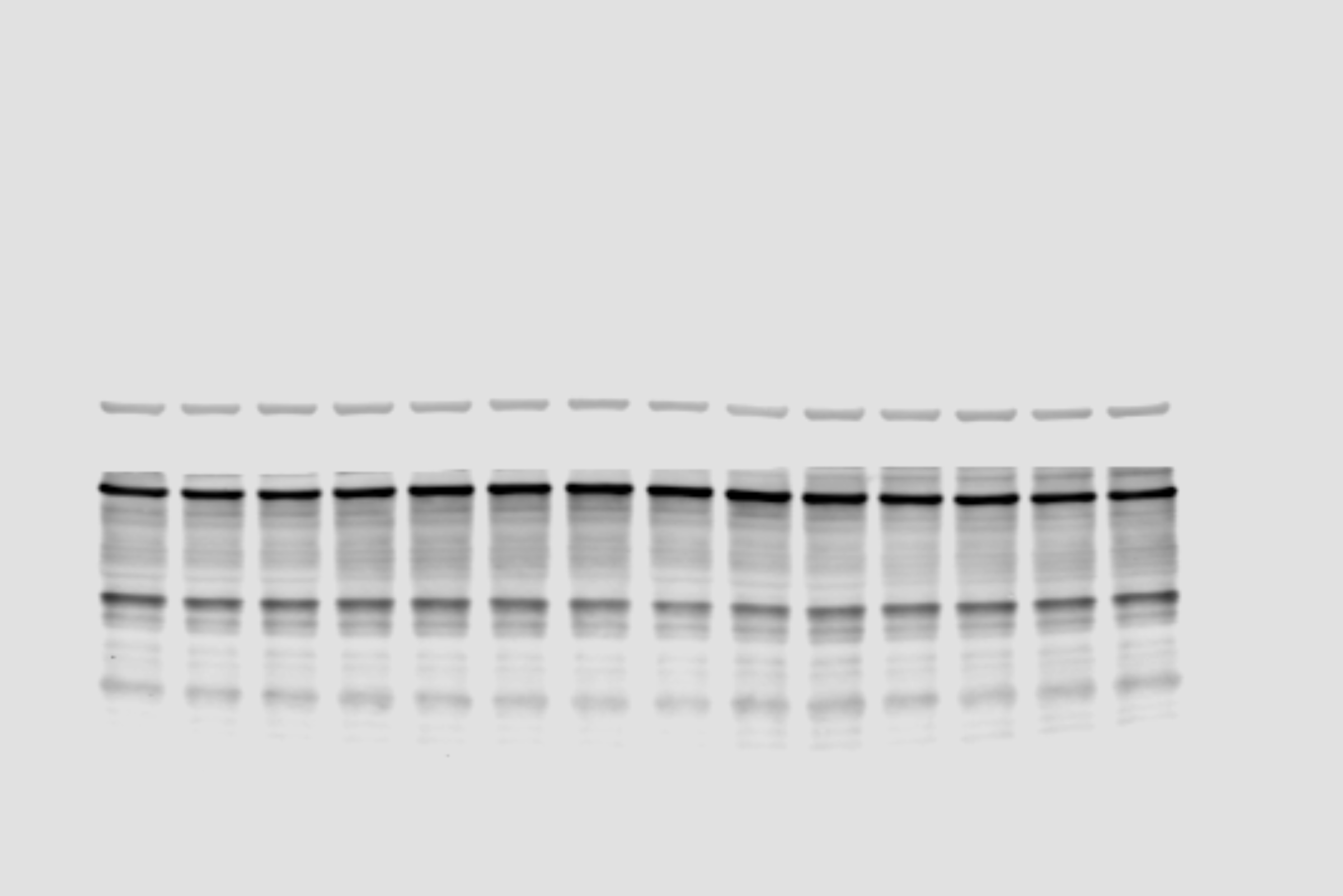

Supplement: Figure 8—source data 1. [file elife-87098-fig8-data1.zip › Figure 8-source data 1/8GH/raw images/1_third gel_800.tif]

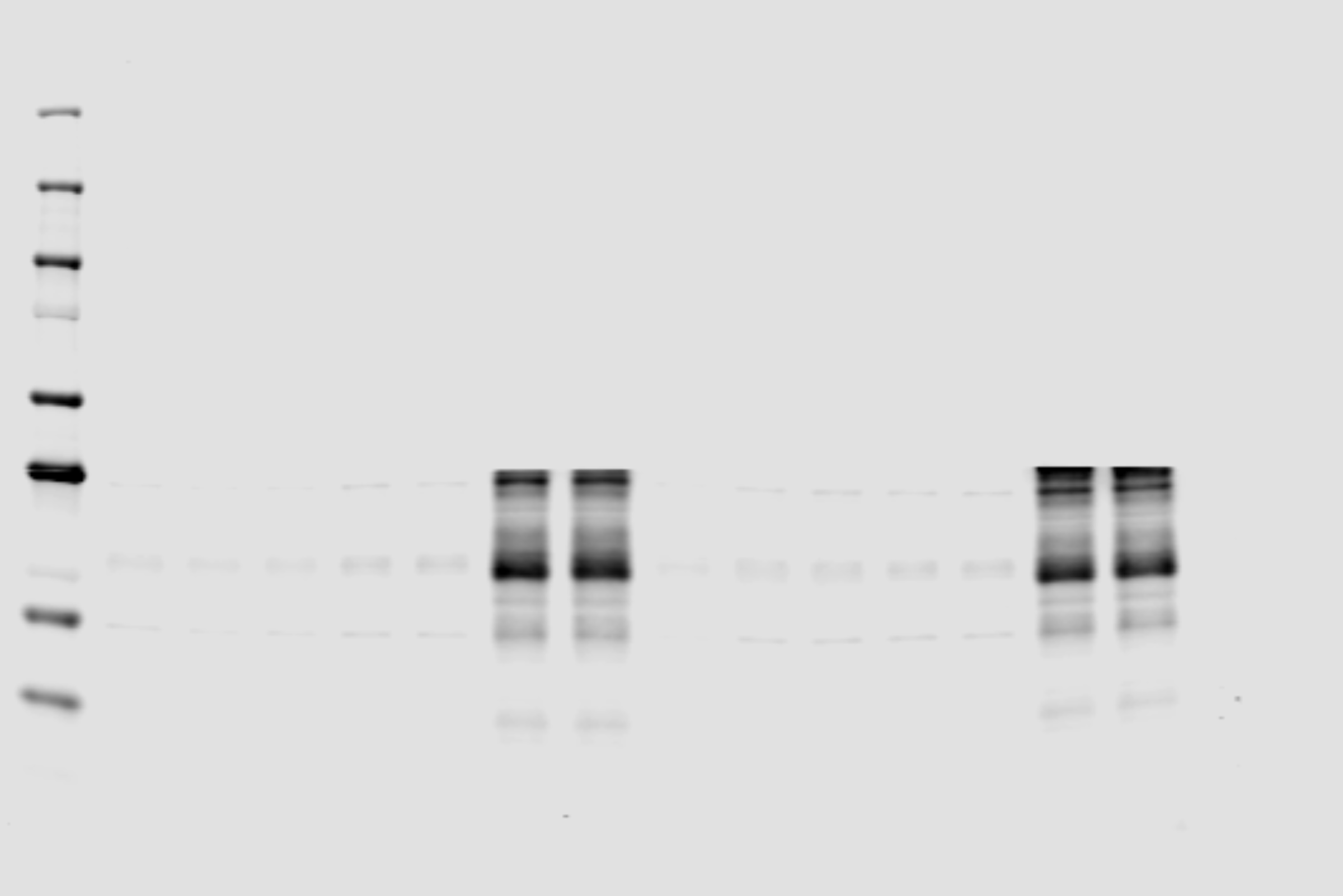

Supplement: Figure 8—source data 1. [file elife-87098-fig8-data1.zip › Figure 8-source data 1/8GH/raw images/1_third gel_680.tif]

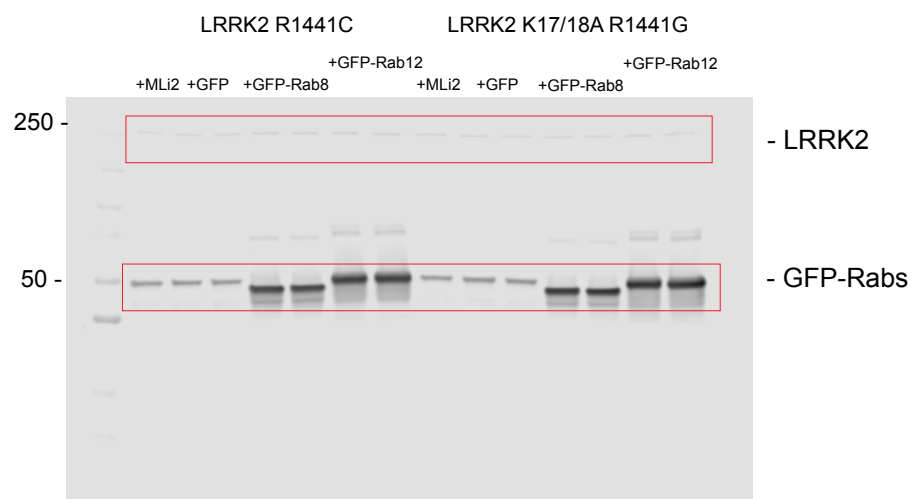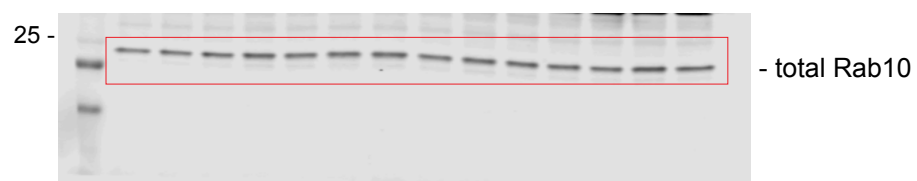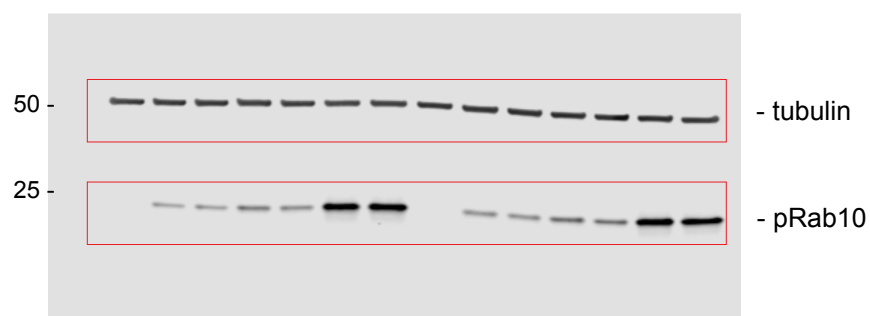

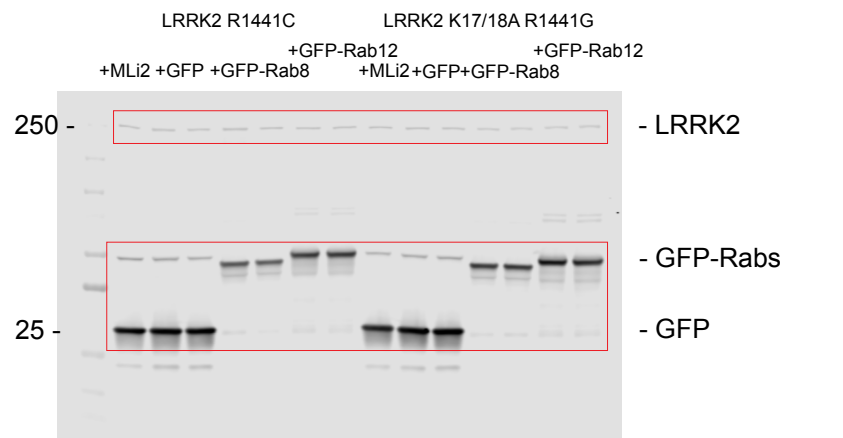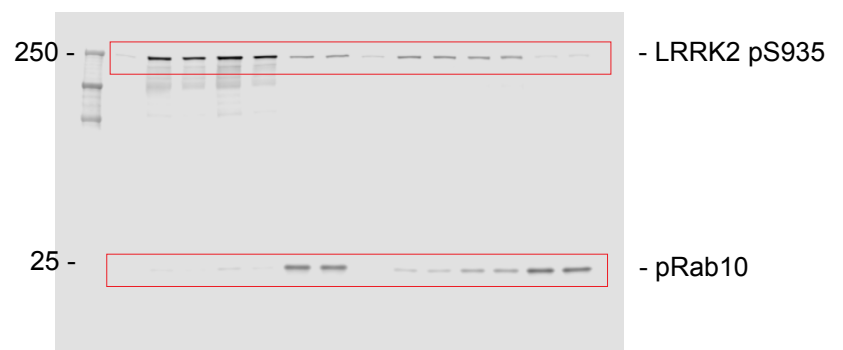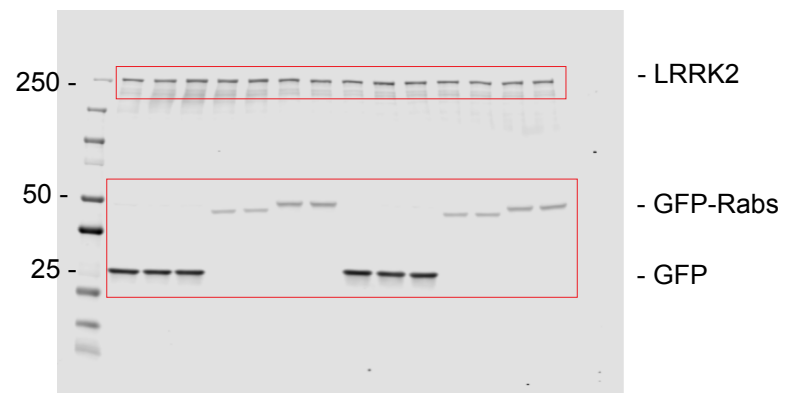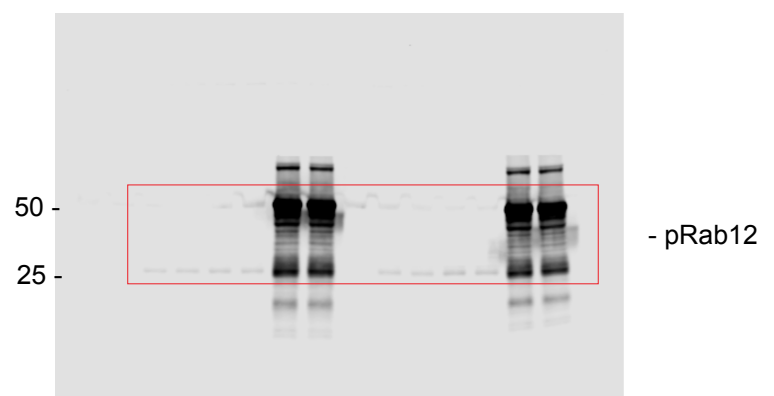

Supplement: Figure 8—source data 1. [file elife-87098-fig8-data1.zip › Figure 8-source data 1/8GH/Supporting material for figure 8GH - annotated blots.pdf]

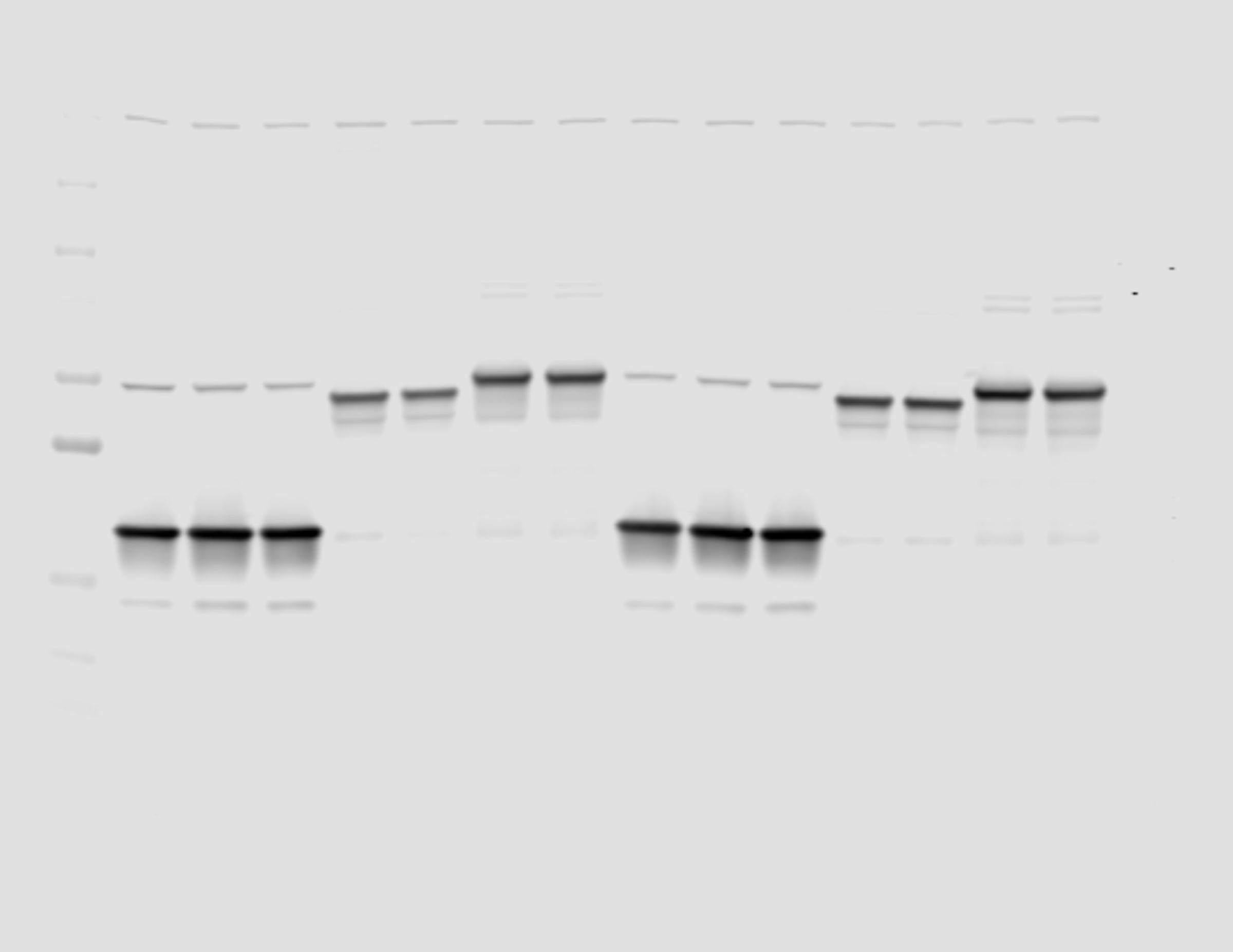

Supplement: Figure 8—source data 1. [file elife-87098-fig8-data1.zip › Figure 8-source data 1/8GH/raw images/1_first gel_680.tif]

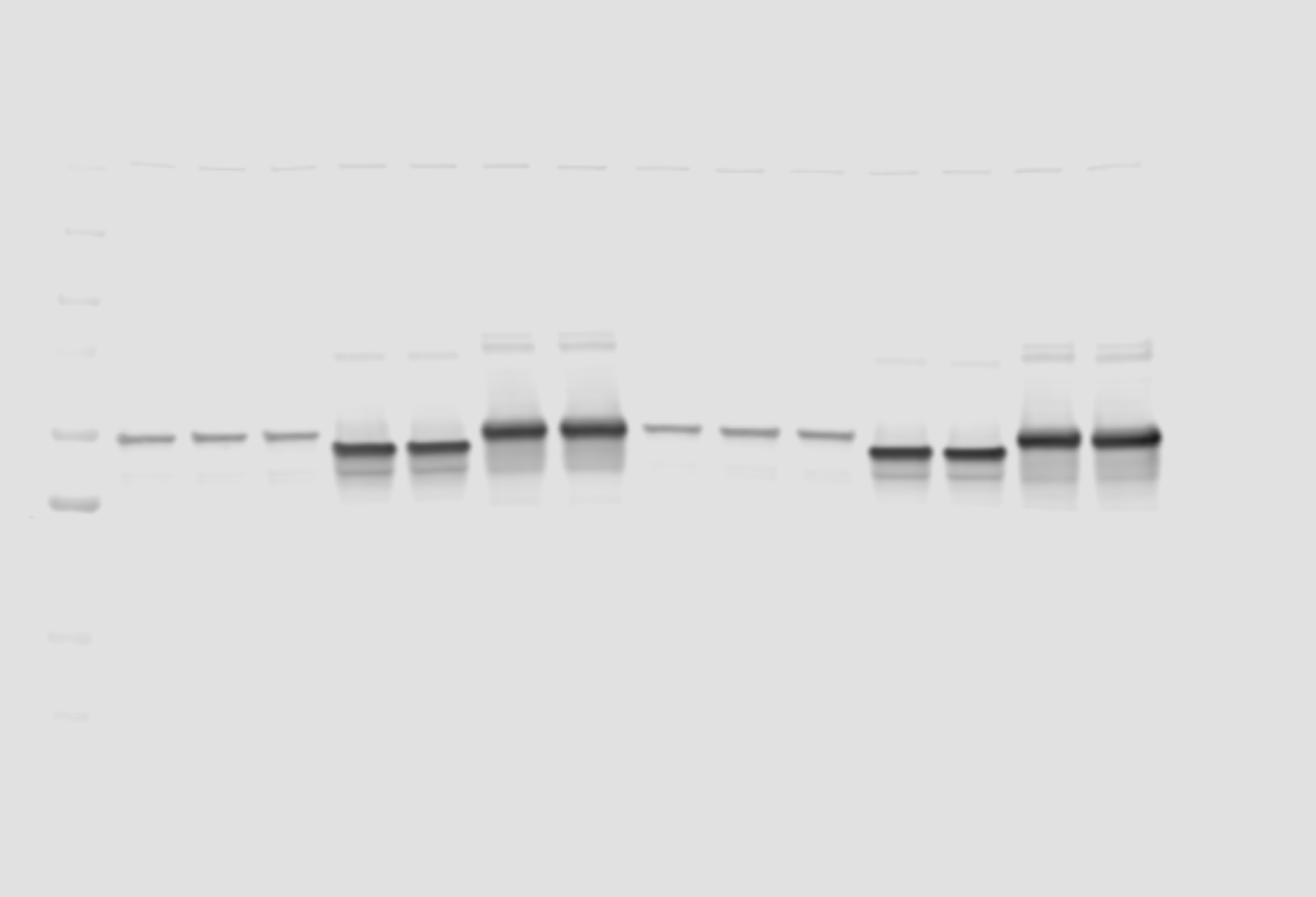

Supplement: Figure 8—source data 1. [file elife-87098-fig8-data1.zip › Figure 8-source data 1/8GH/raw images/2_680.tif]

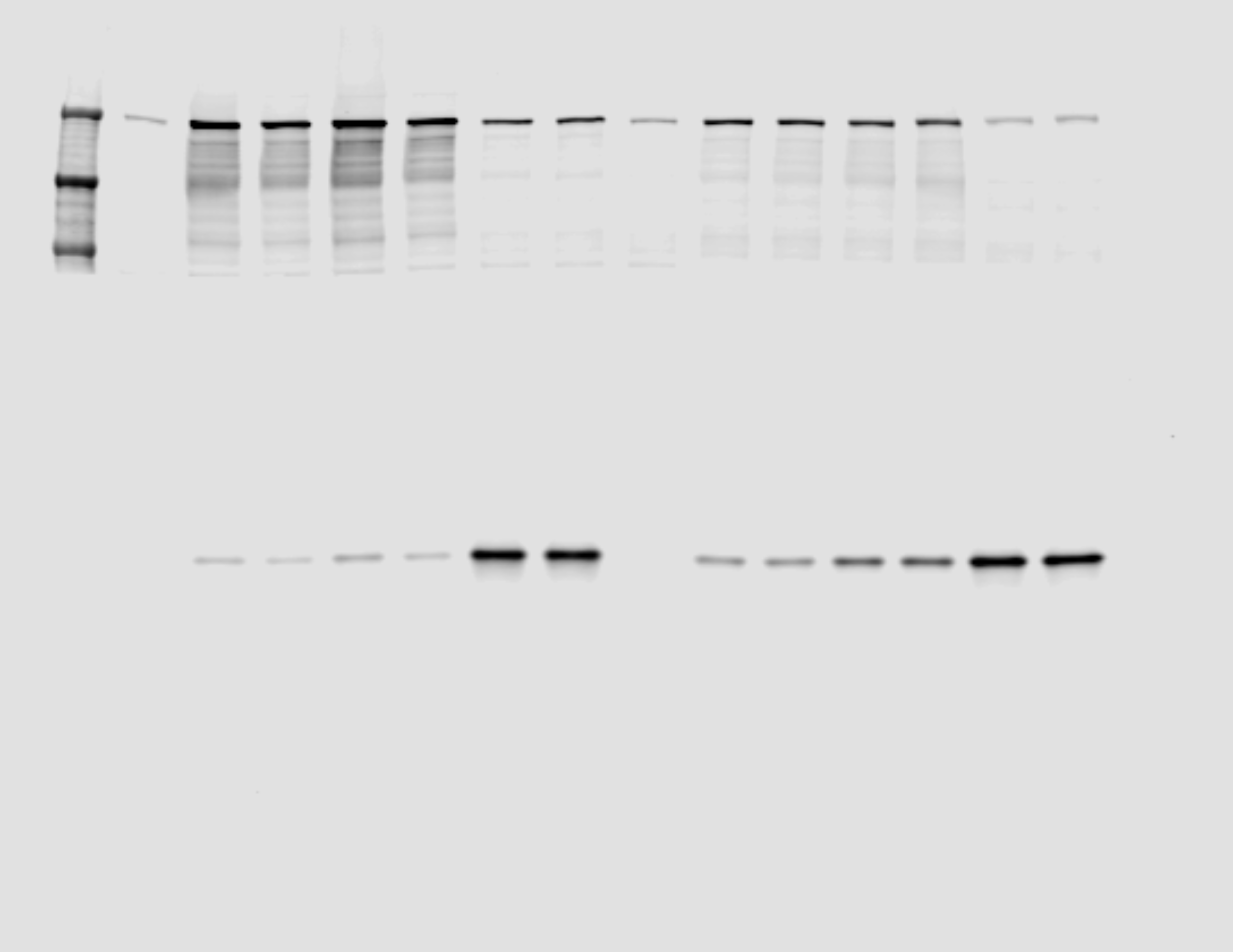

Supplement: Figure 8—source data 1. [file elife-87098-fig8-data1.zip › Figure 8-source data 1/8GH/raw images/1_first gel_800.tif]

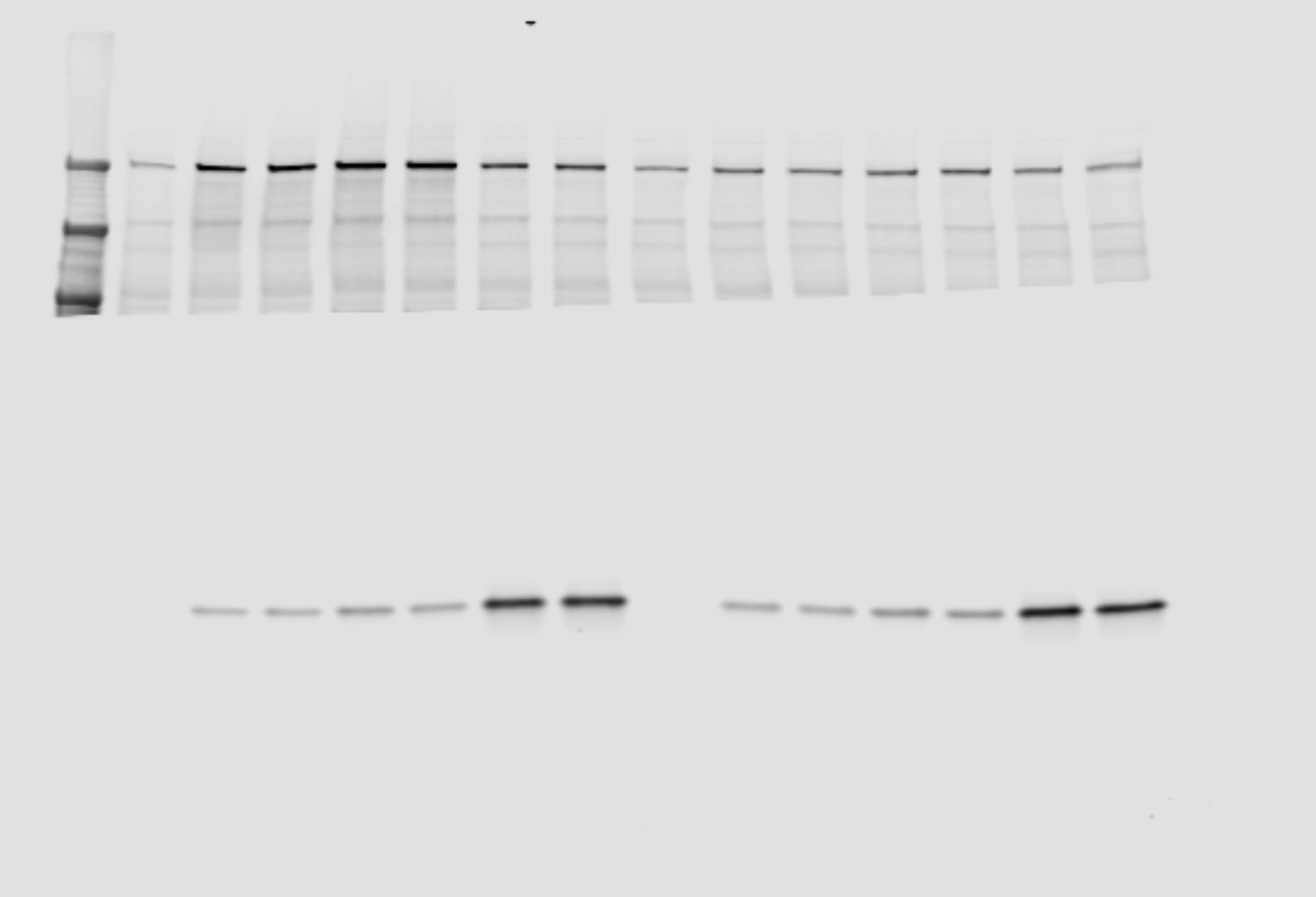

Supplement: Figure 8—source data 1. [file elife-87098-fig8-data1.zip › Figure 8-source data 1/8GH/raw images/2_800.tif]

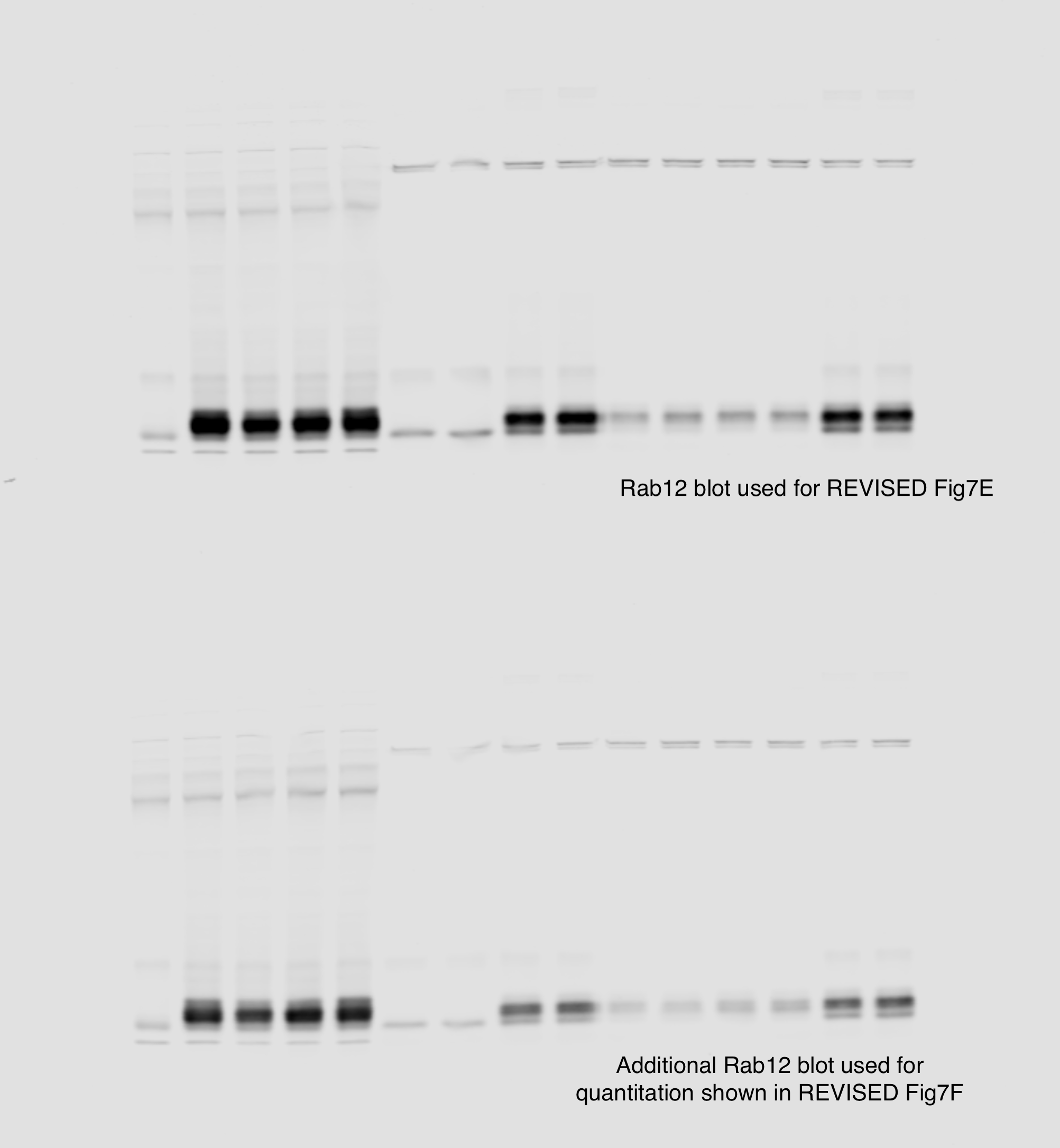

Supplement: Figure 8—source data 1. [file elife-87098-fig8-data1.zip › Figure 8-source data 1/8E/annotated/REVISED-Fig8E_30-05-2023_800.tif]

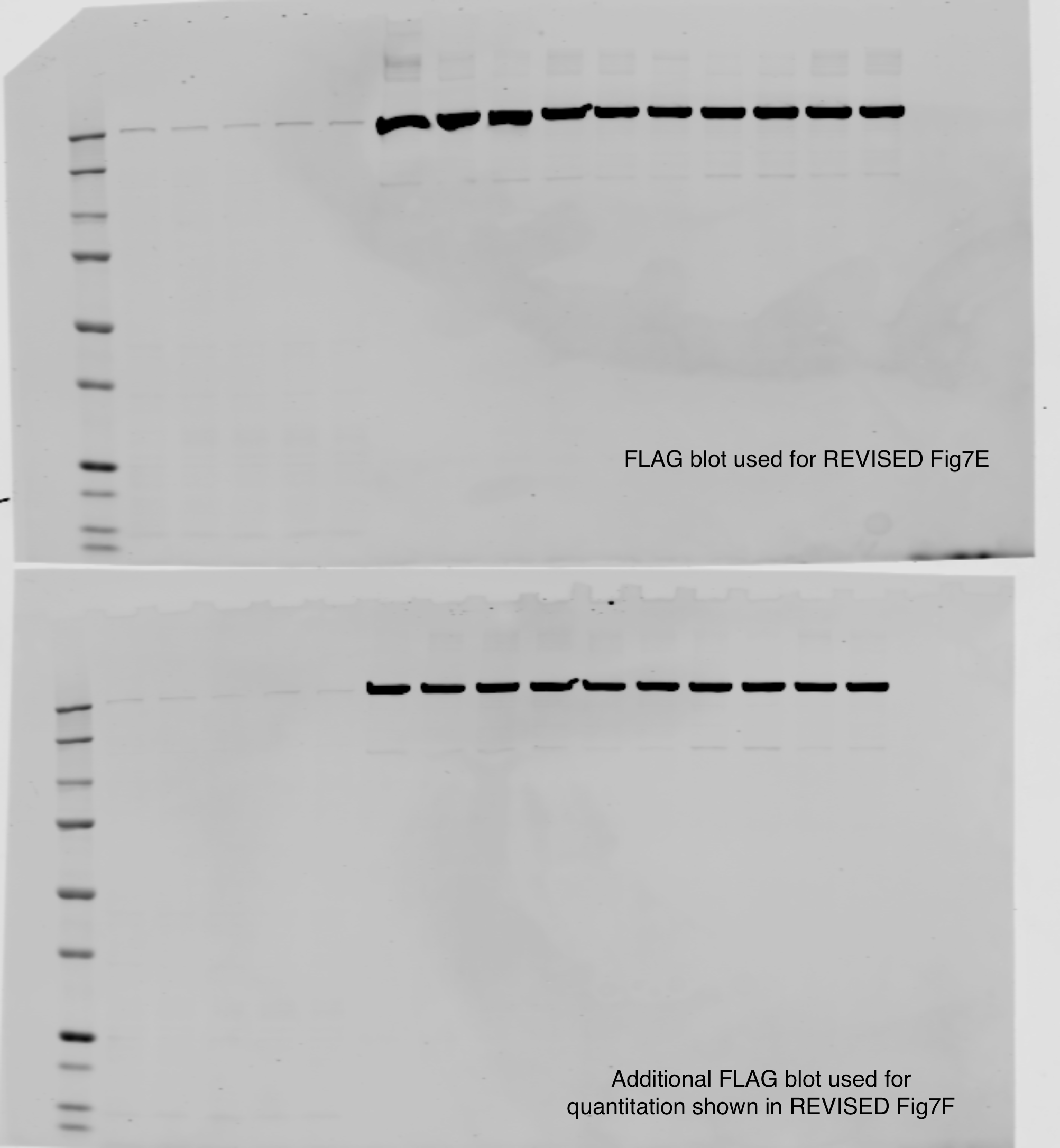

Supplement: Figure 8—source data 1. [file elife-87098-fig8-data1.zip › Figure 8-source data 1/8E/annotated/REVISED-Fig8E_31-05-2023_700.tif]

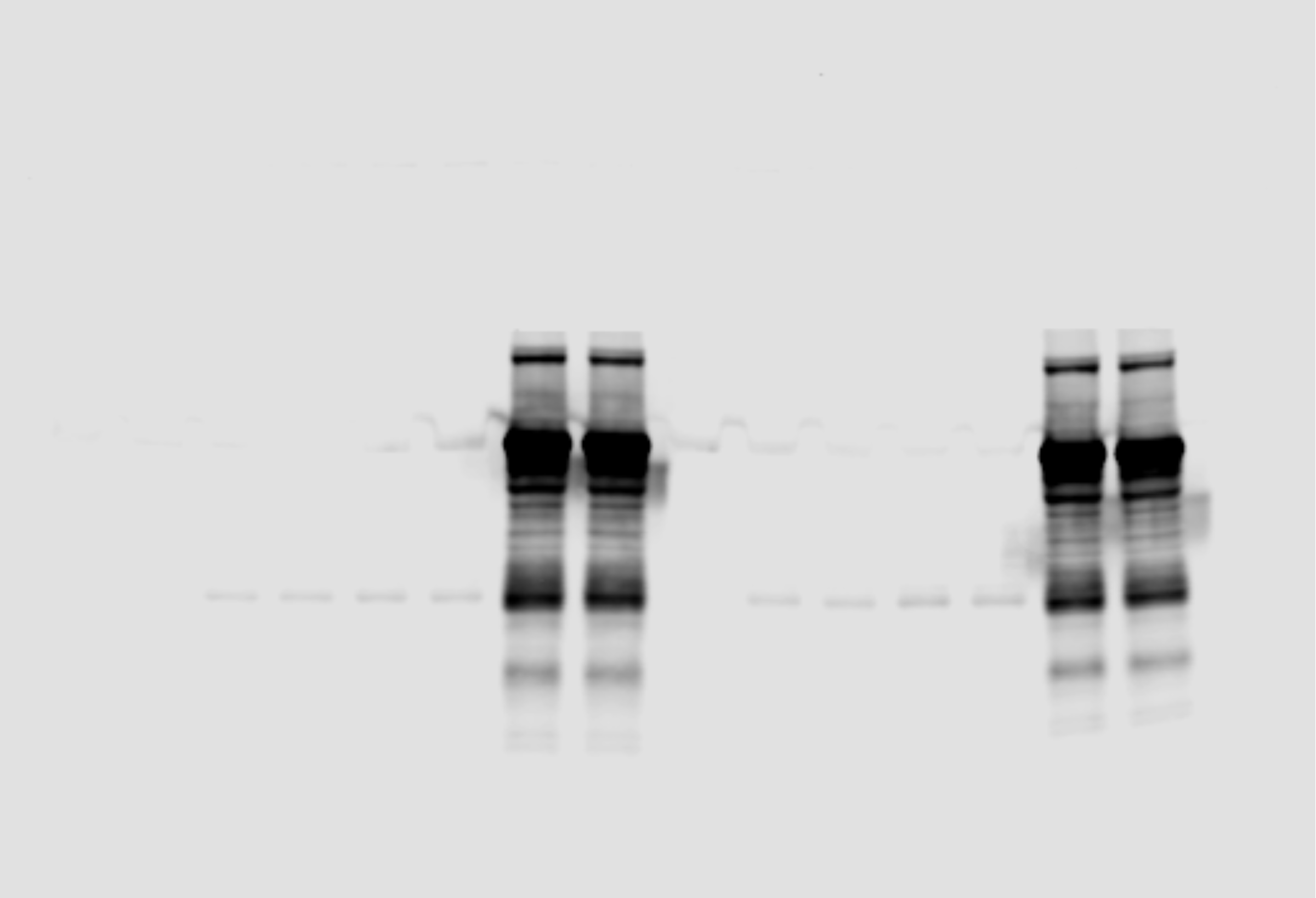

Supplement: Figure 8—source data 1. [file elife-87098-fig8-data1.zip › Figure 8-source data 1/8GH/raw images/1_second gel_800.tif]

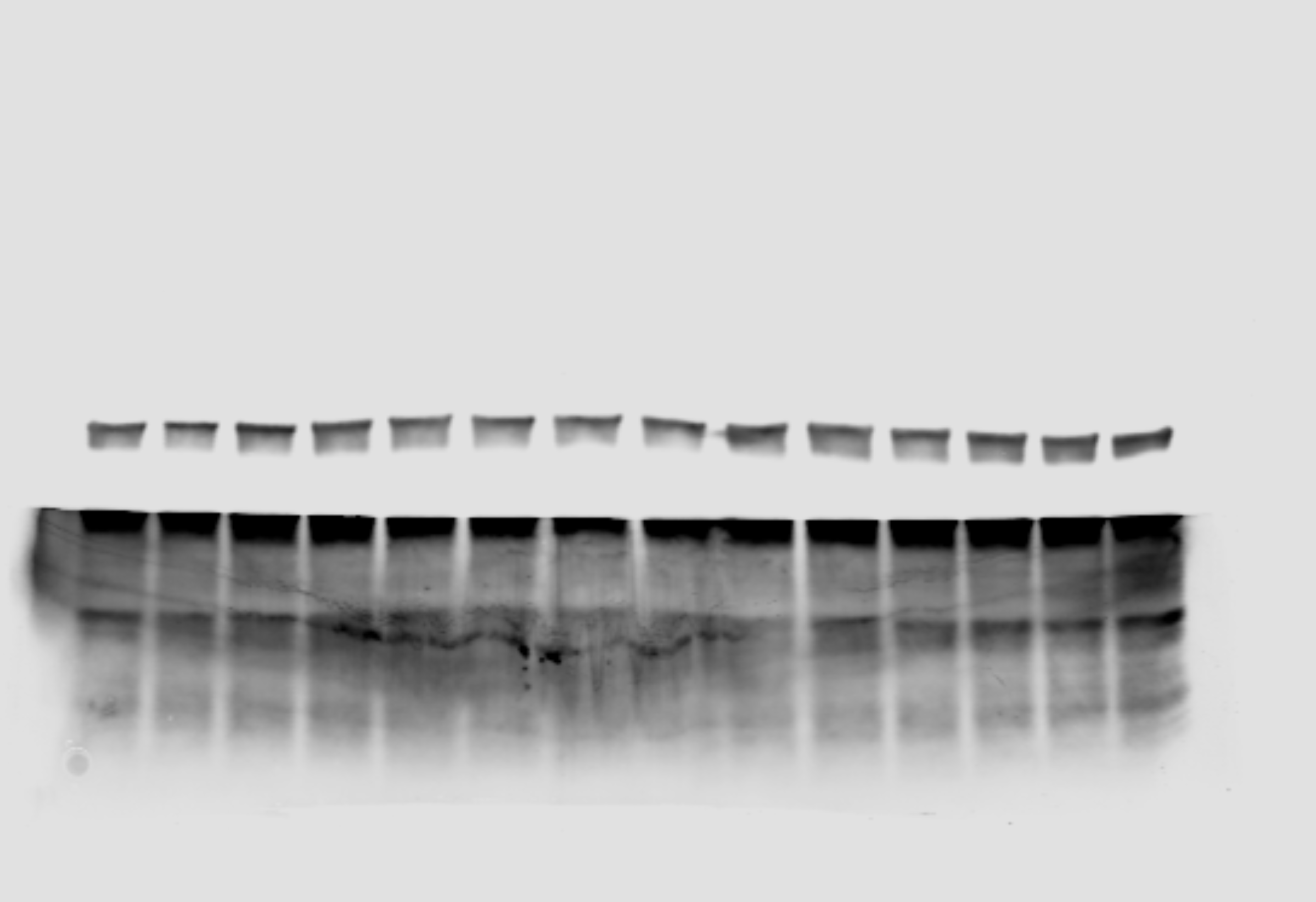

Supplement: Figure 8—source data 1. [file elife-87098-fig8-data1.zip › Figure 8-source data 1/8GH/raw images/2_tubulin.tif]

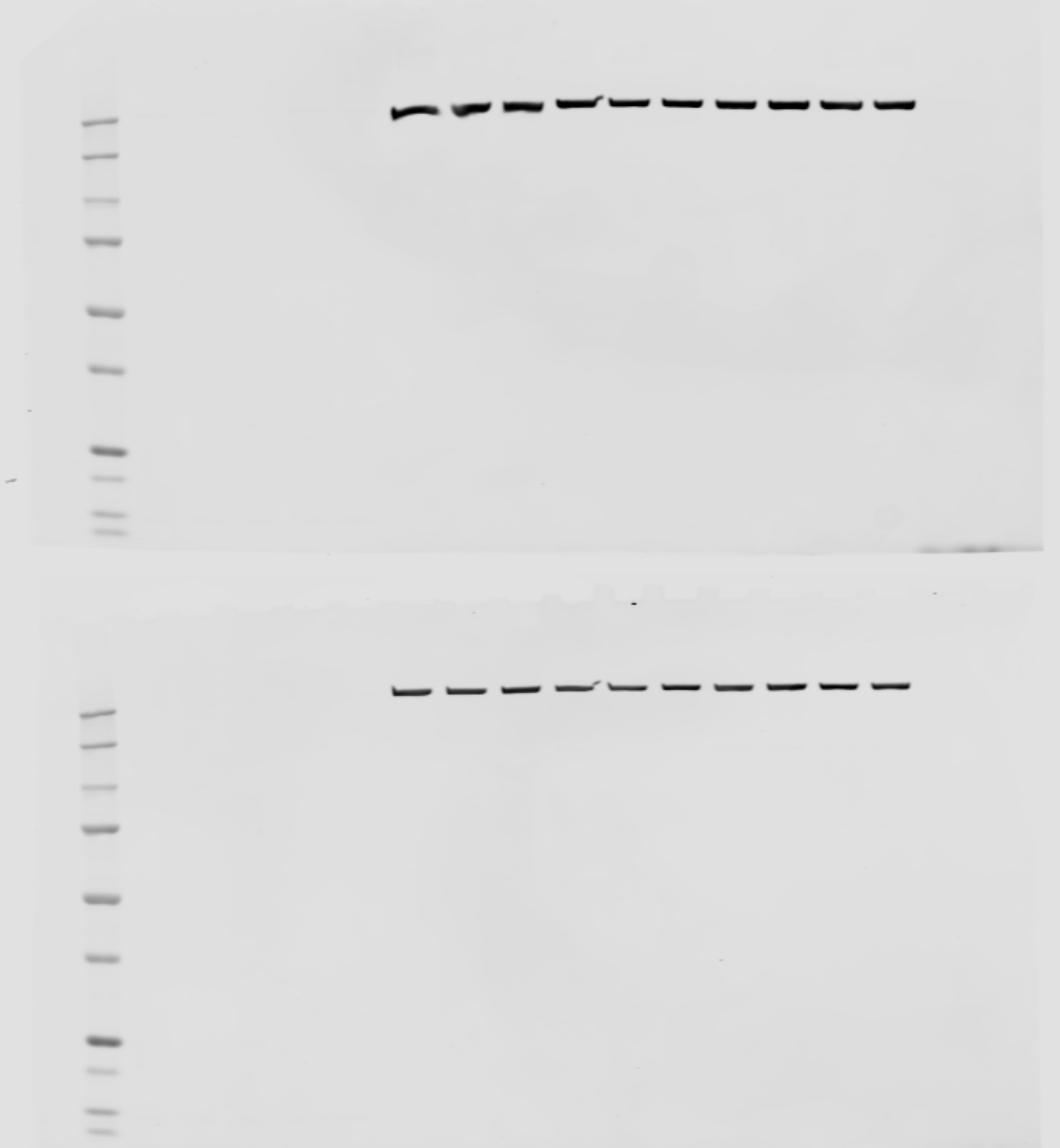

Supplement: Figure 8—source data 1. [file elife-87098-fig8-data1.zip › Figure 8-source data 1/8E/raw images/8E_700.tif]

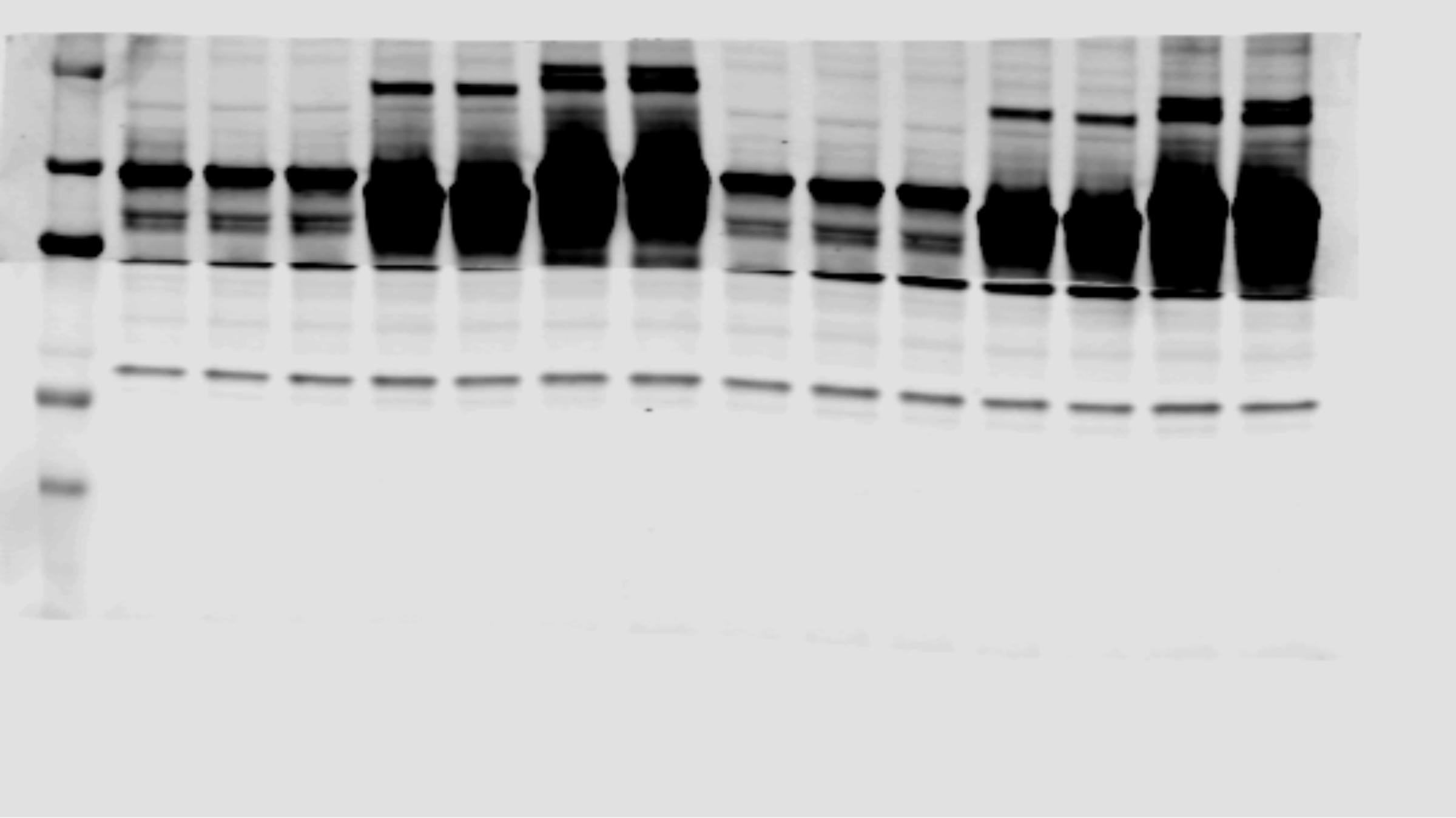

Supplement: Figure 8—source data 1. [file elife-87098-fig8-data1.zip › Figure 8-source data 1/8GH/raw images/2_680 reprobe.tif]

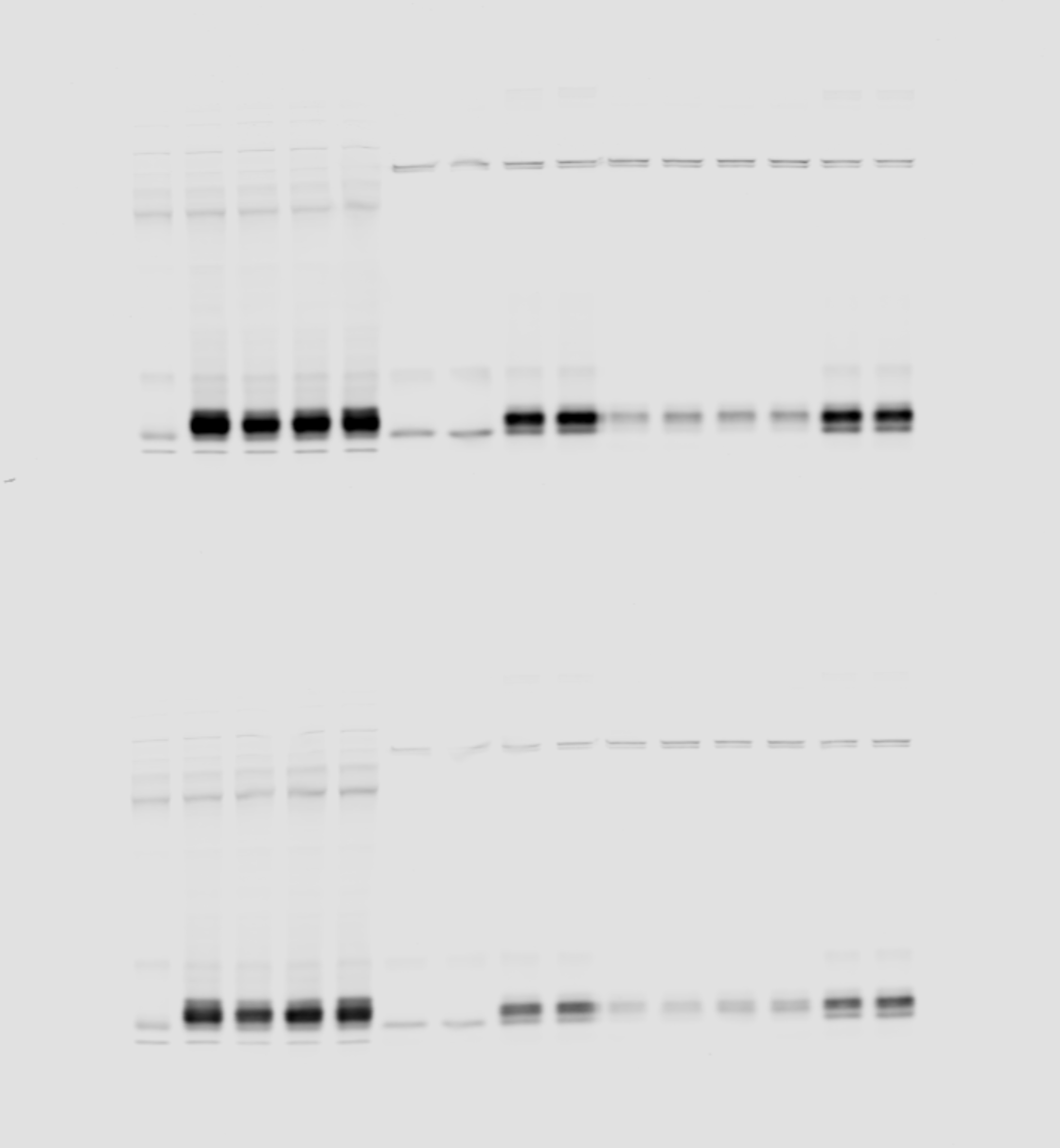

Supplement: Figure 8—source data 1. [file elife-87098-fig8-data1.zip › Figure 8-source data 1/8E/raw images/8E_800.tif]

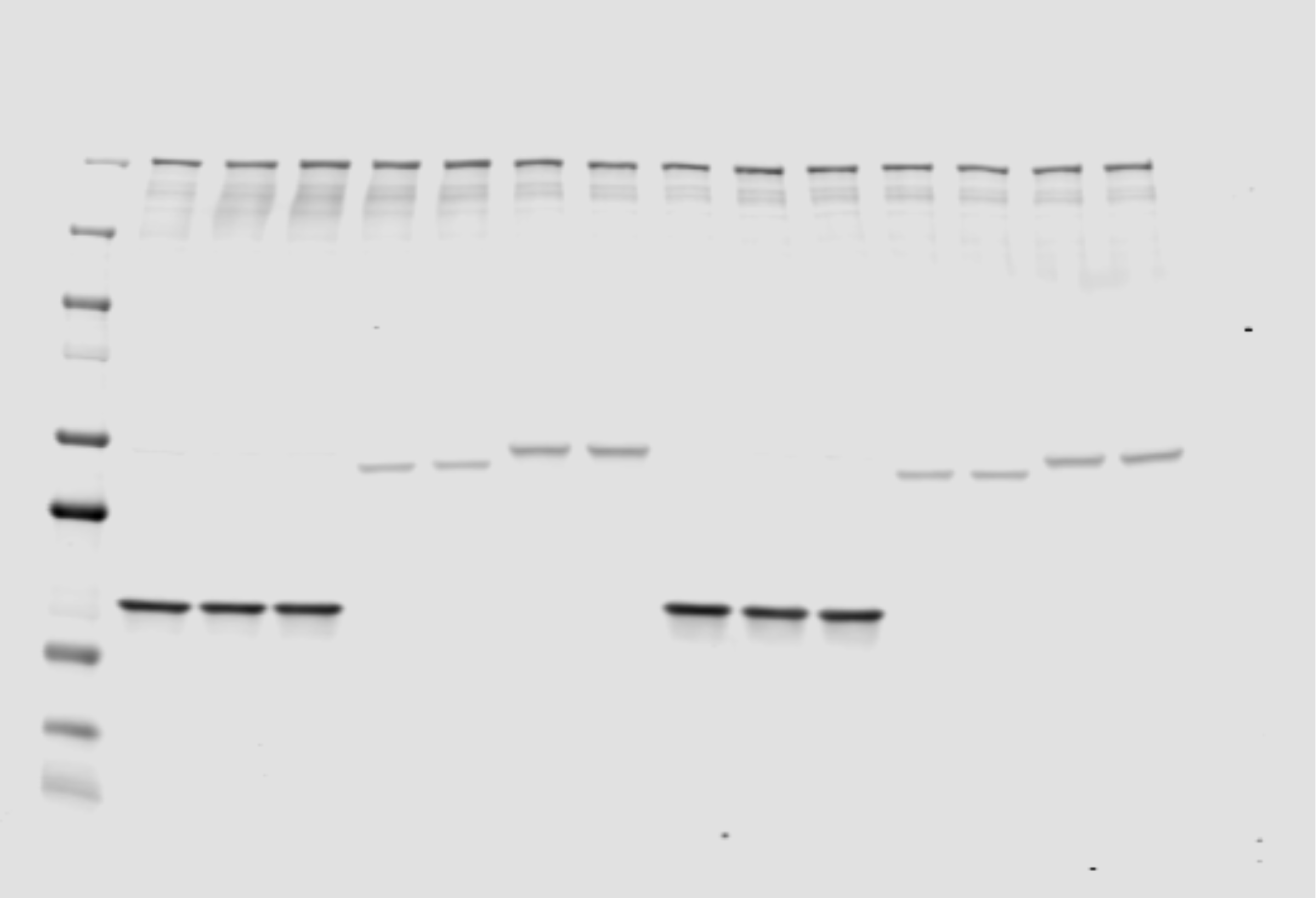

Supplement: Figure 8—source data 1. [file elife-87098-fig8-data1.zip › Figure 8-source data 1/8GH/raw images/1_second gel_680.tif]

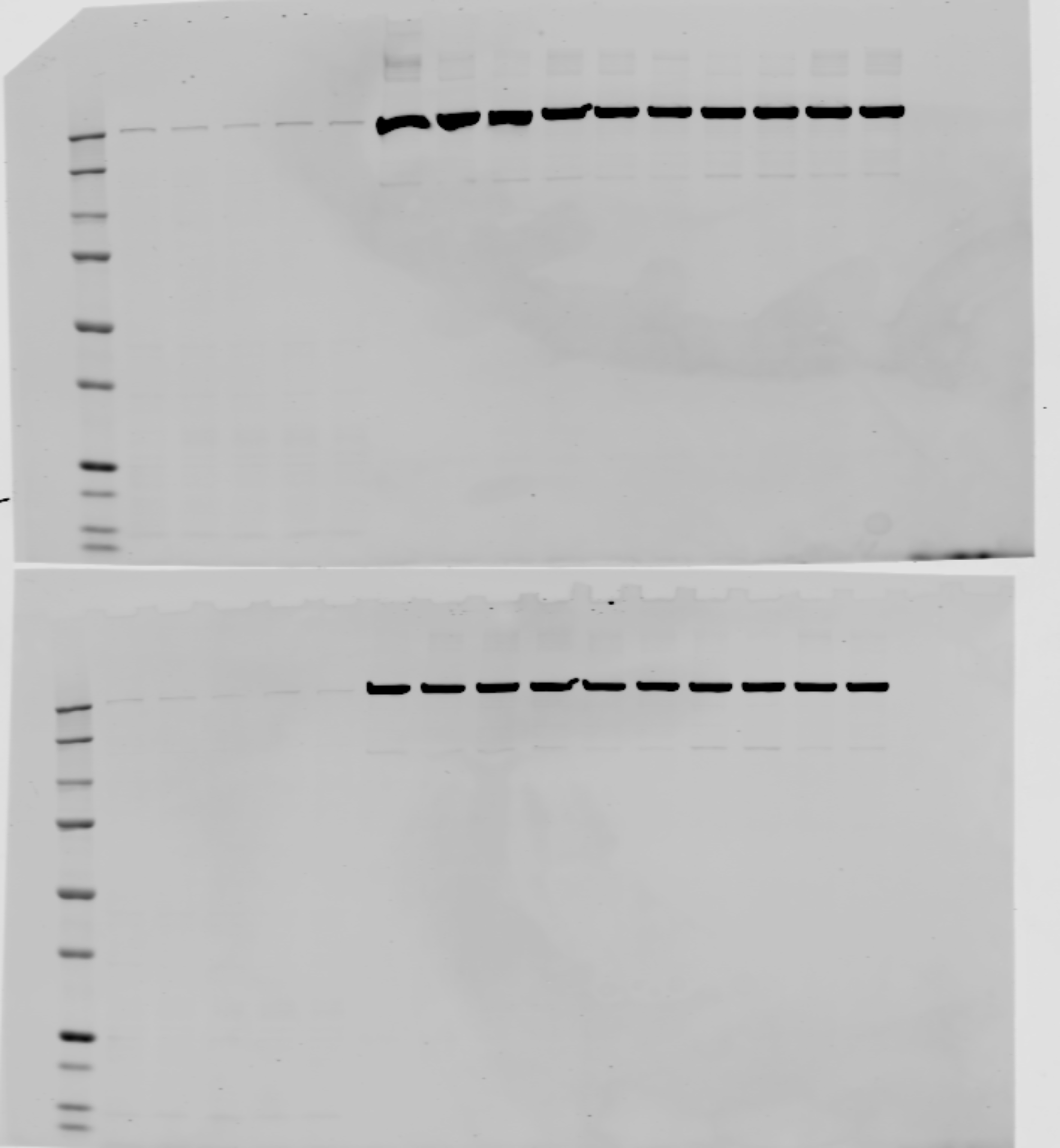

Supplement: Figure 8—source data 1. [file elife-87098-fig8-data1.zip › Figure 8-source data 1/8E/raw images/8E_re-probed_700.tif]

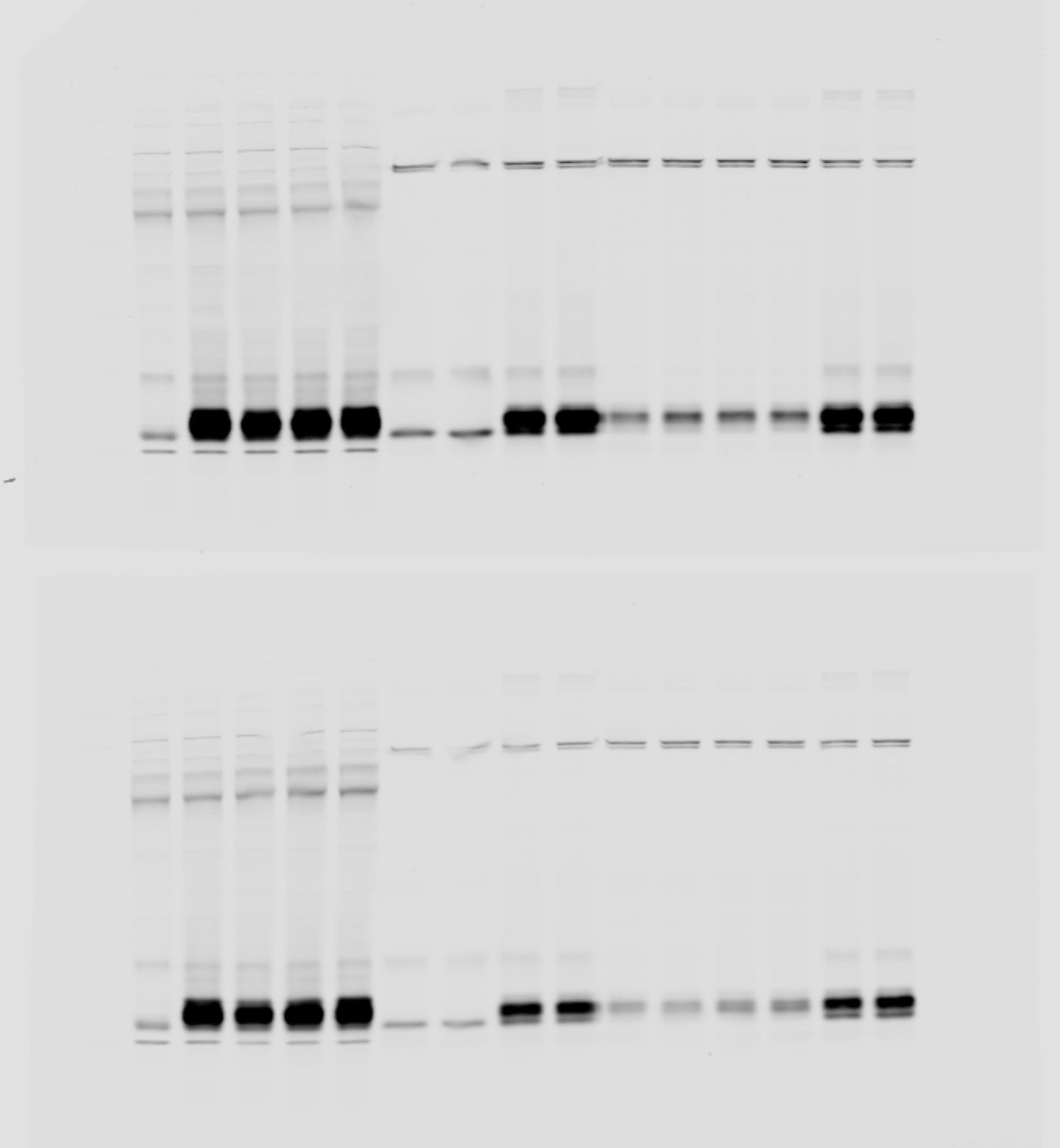

Supplement: Figure 8—source data 1. [file elife-87098-fig8-data1.zip › Figure 8-source data 1/8E/raw images/8E_800-high.tif]

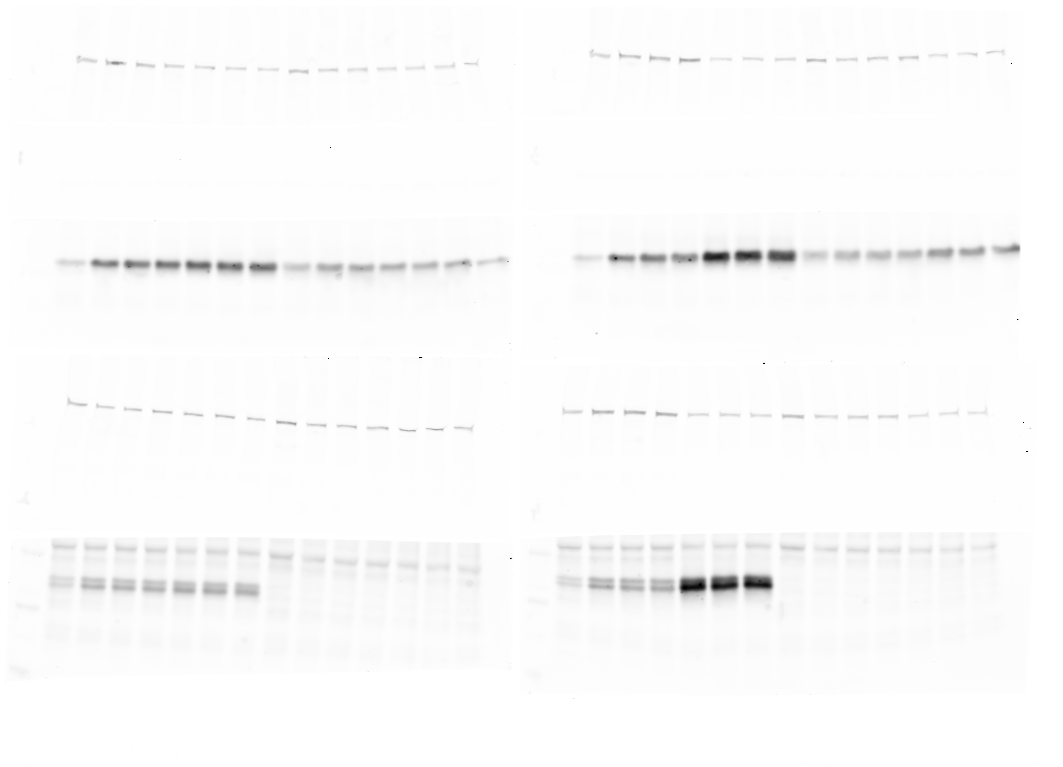

Supplement: Figure 9—source data 1. [file elife-87098-fig9-data1.zip › Figure 9-source data 1/Revised Fig 9A and Fig 9F_800_.tif]

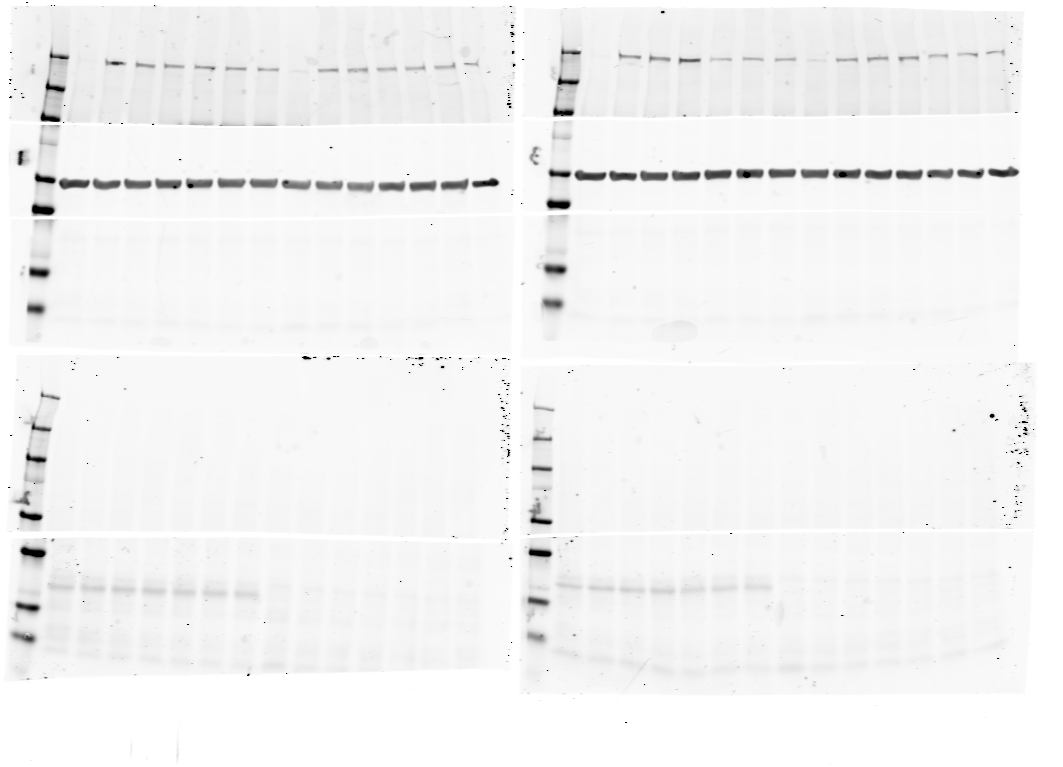

Supplement: Figure 9—source data 1. [file elife-87098-fig9-data1.zip › Figure 9-source data 1/Revised Fig 9A and Fig 9F_700_.tif]

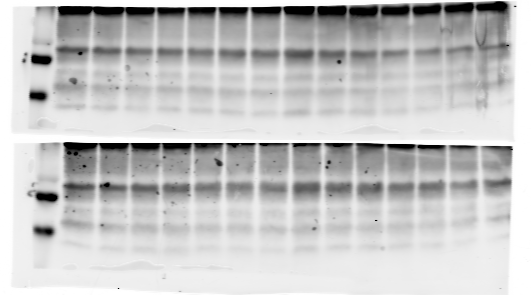

Supplement: Figure 9—source data 1. [file elife-87098-fig9-data1.zip › Figure 9-source data 1/Revised Fig 9A and Fig 9F_700__.tif]

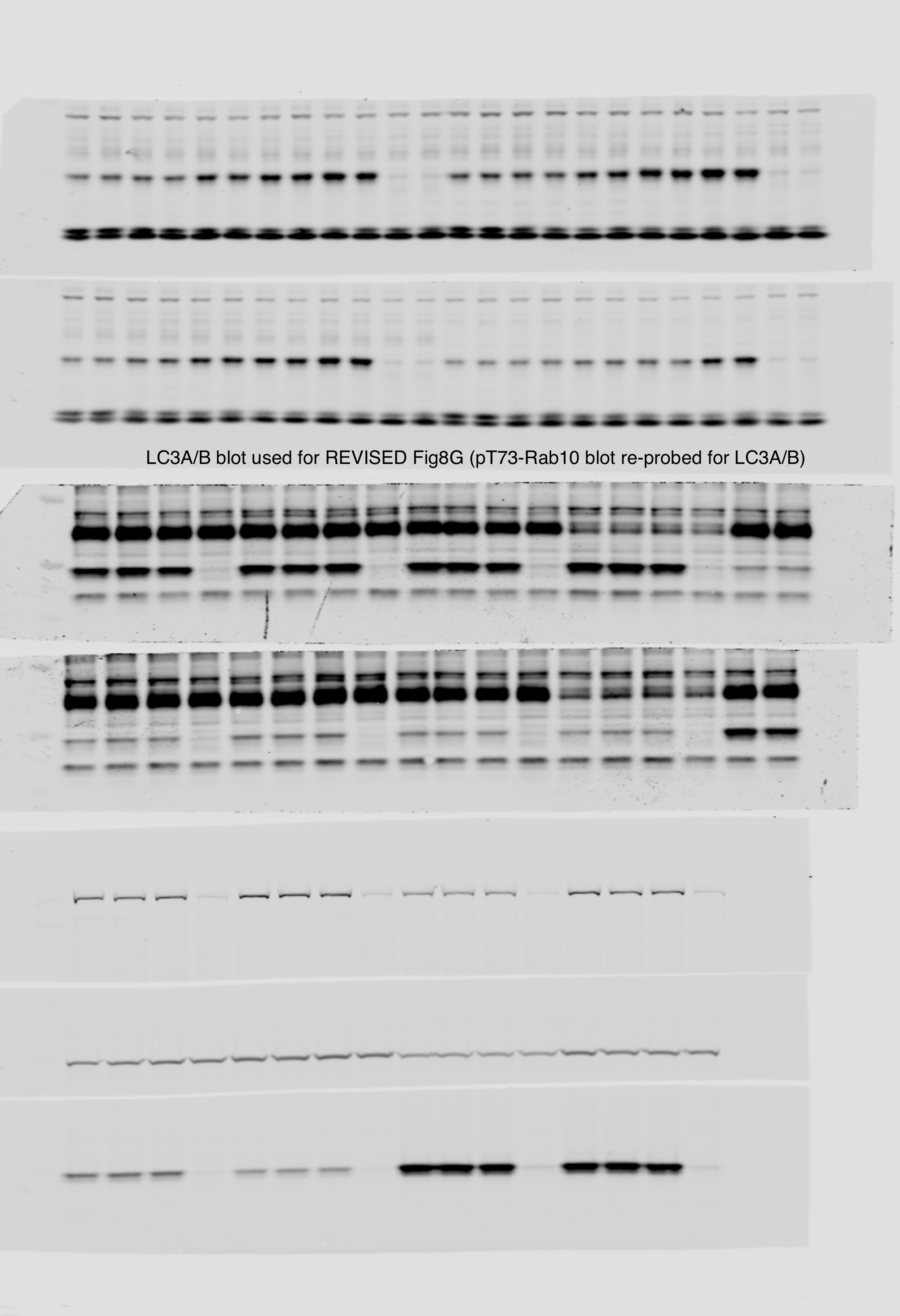

Supplement: Figure 9—source data 1. [file elife-87098-fig9-data1.zip › Figure 9-source data 1/annotated/REVISED-Fig9D_07-03-2023_800.tif]

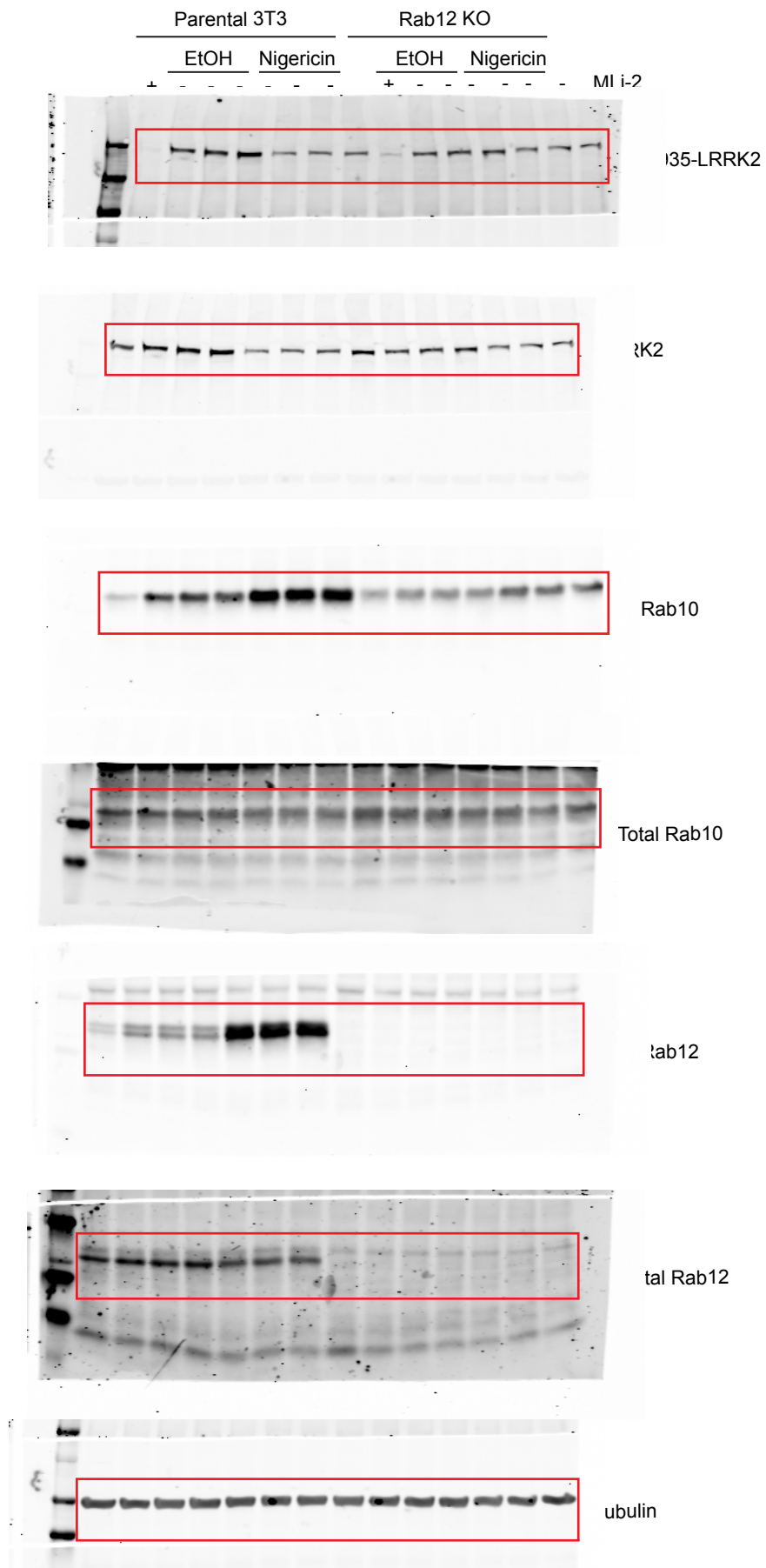

Supplement: Figure 9—source data 1. [file elife-87098-fig9-data1.zip › Figure 9-source data 1/annotated/Supporting material for figure 9F_G_H_annotated blots.pdf]

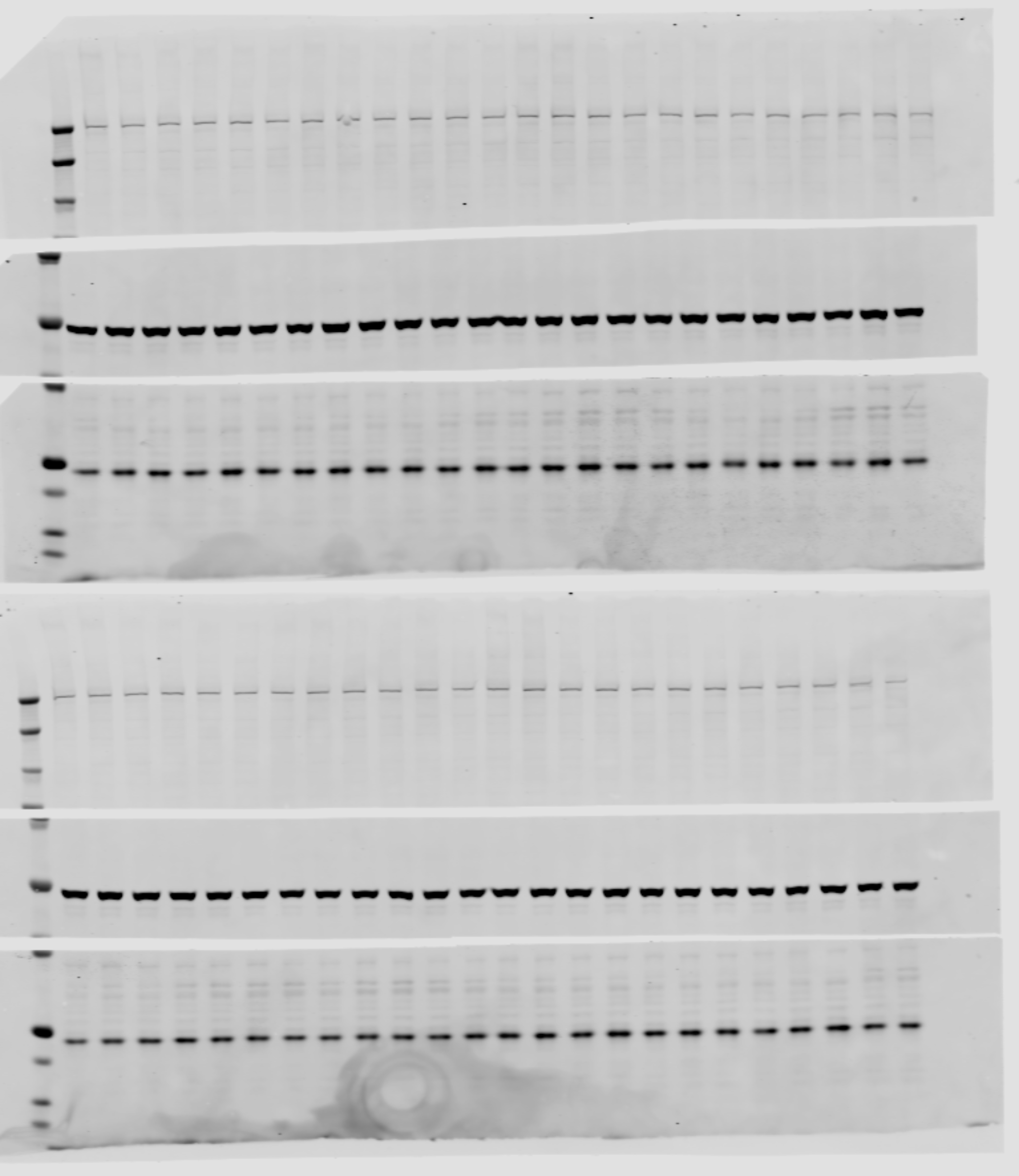

Supplement: Figure 9—source data 1. [file elife-87098-fig9-data1.zip › Figure 9-source data 1/Fig 9D/9D_700.tif]

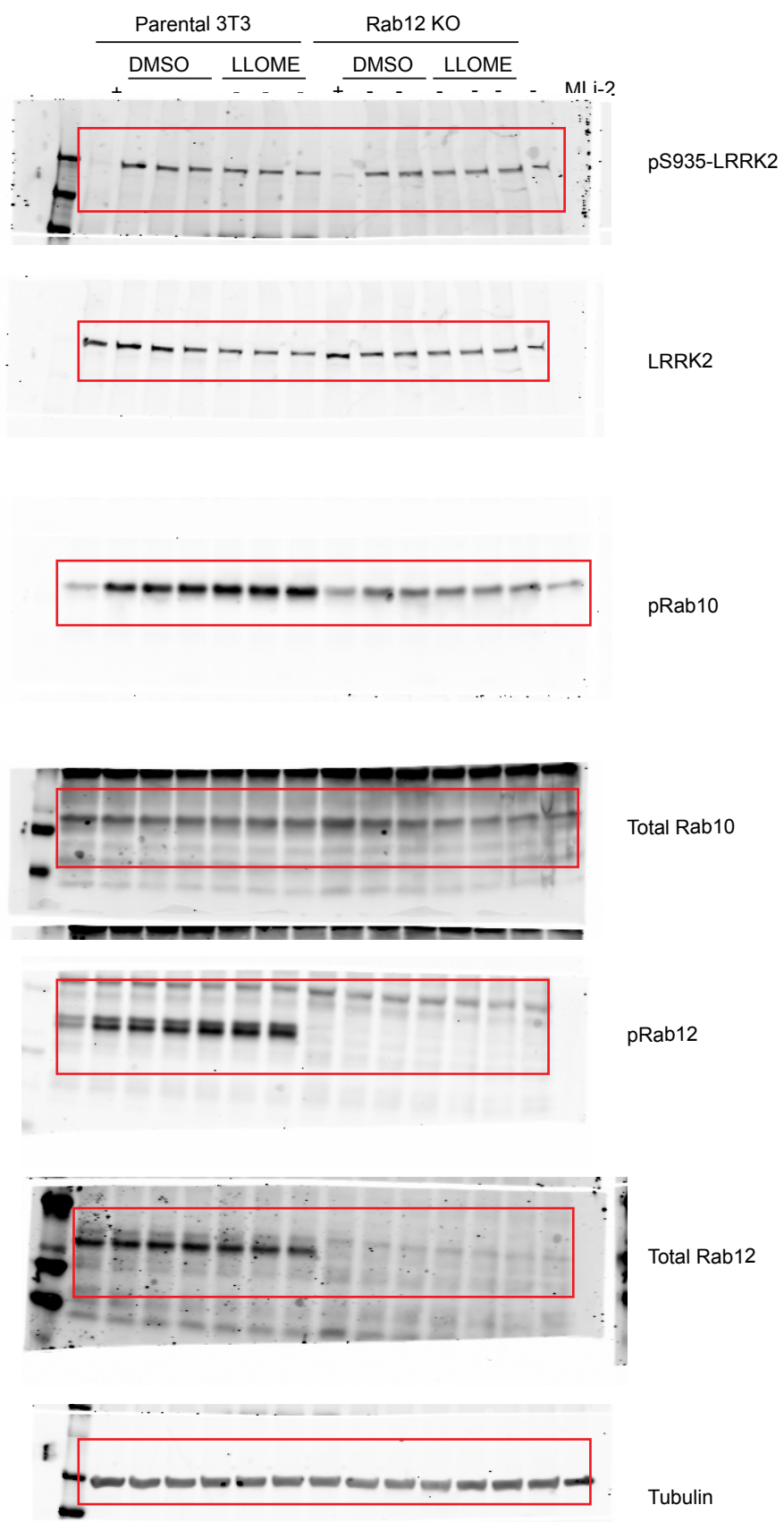

Supplement: Figure 9—source data 1. [file elife-87098-fig9-data1.zip › Figure 9-source data 1/annotated/Supporting material for figure 9A_B_C_annotated blots.pdf]

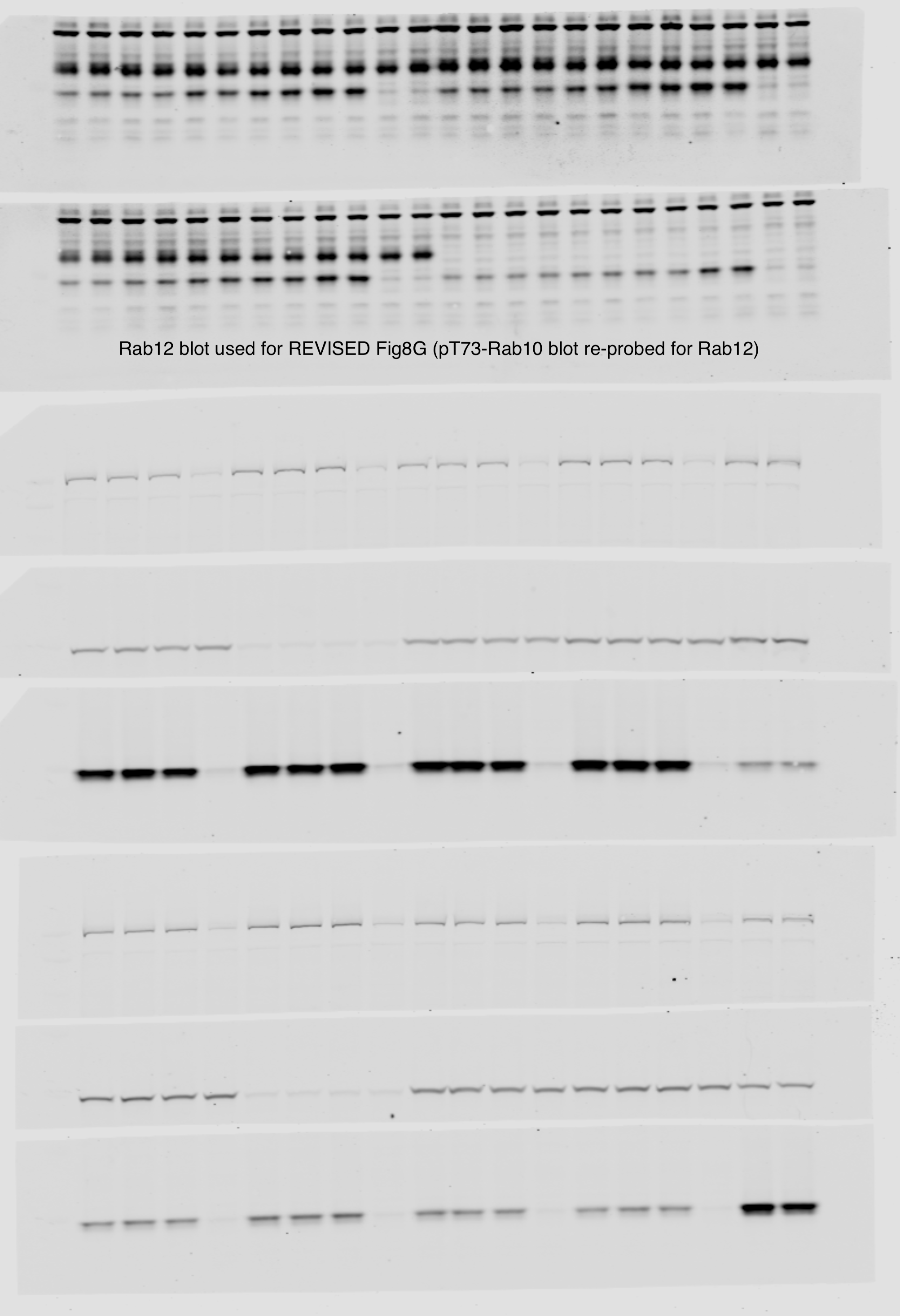

Supplement: Figure 9—source data 1. [file elife-87098-fig9-data1.zip › Figure 9-source data 1/annotated/REVISED-Fig9D_03-03-23_800.tif]

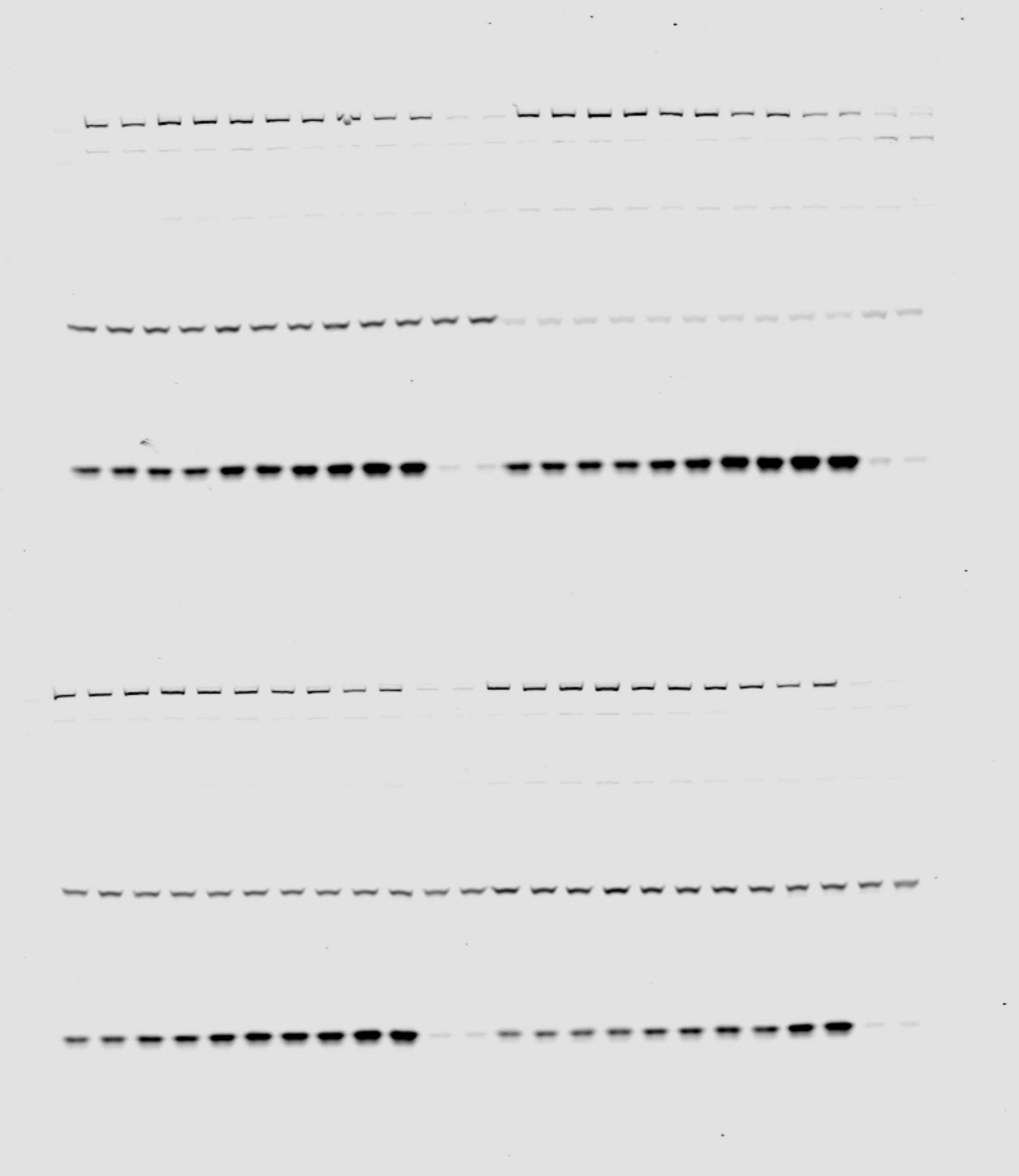

Supplement: Figure 9—source data 1. [file elife-87098-fig9-data1.zip › Figure 9-source data 1/Fig 9D/9D_800-low.tif]

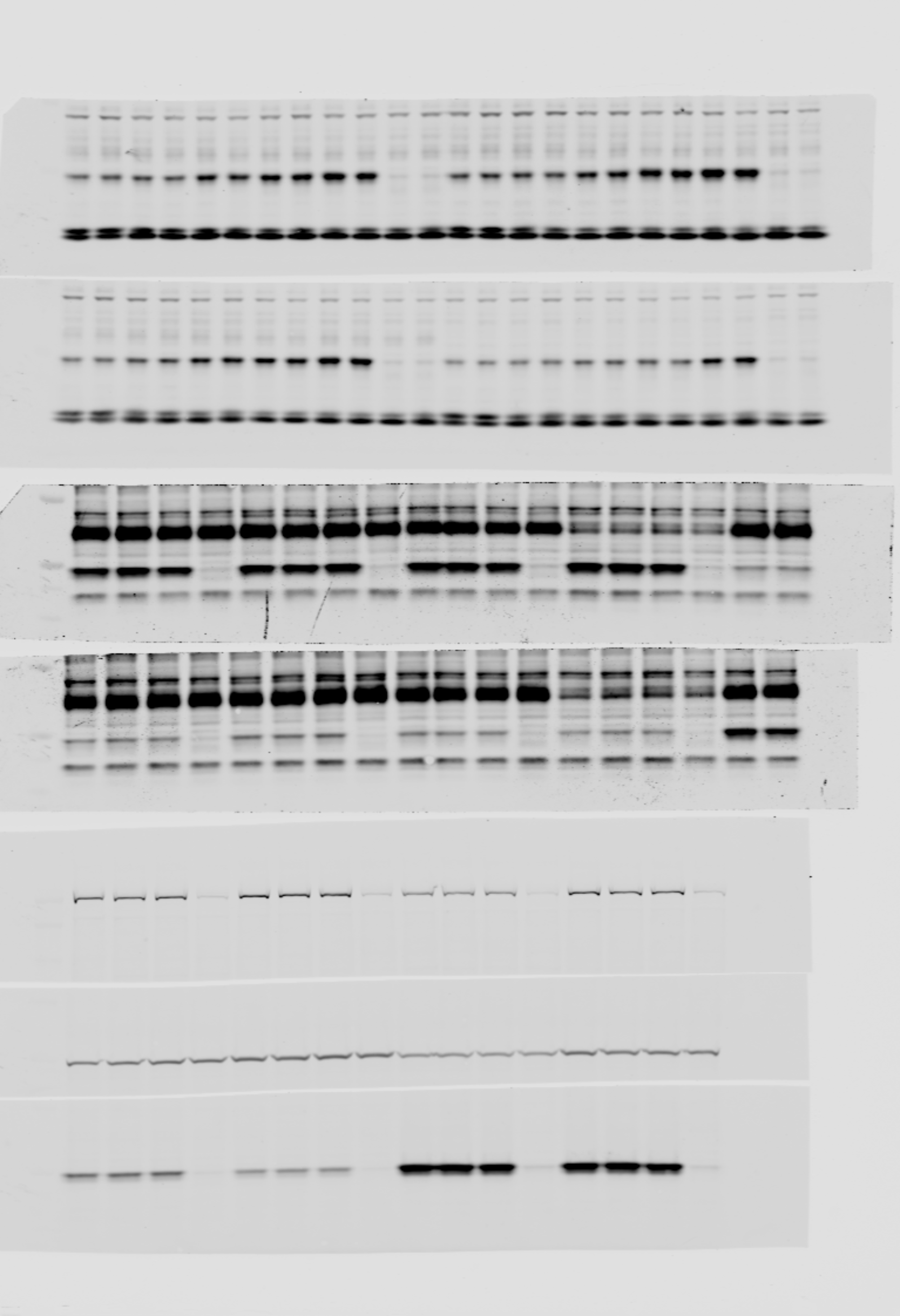

Supplement: Figure 9—source data 1. [file elife-87098-fig9-data1.zip › Figure 9-source data 1/Fig 9D/9D_LC3_800.tif]

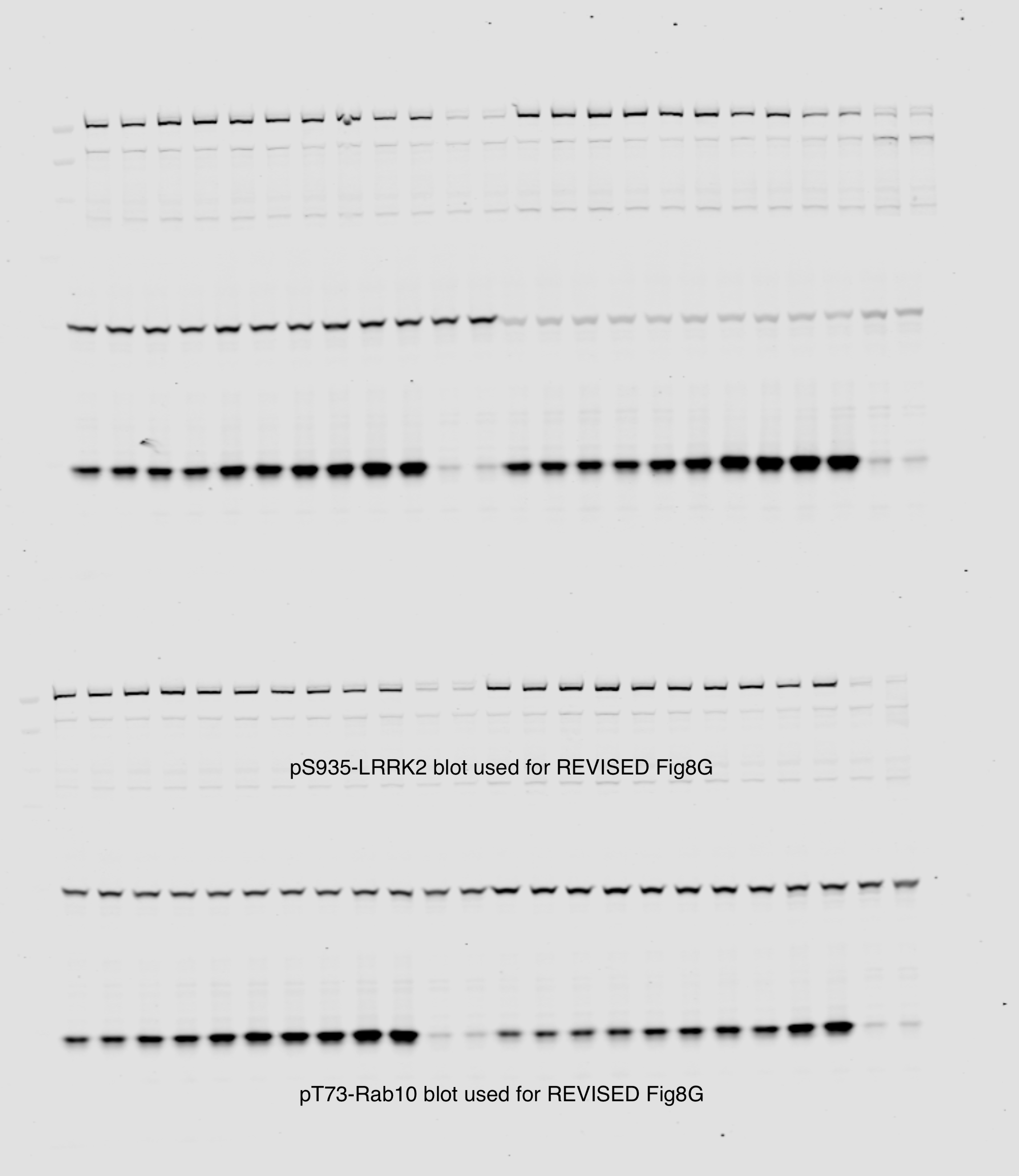

Supplement: Figure 9—source data 1. [file elife-87098-fig9-data1.zip › Figure 9-source data 1/annotated/REVISED-Fig9D_02-03-23_800.tif]

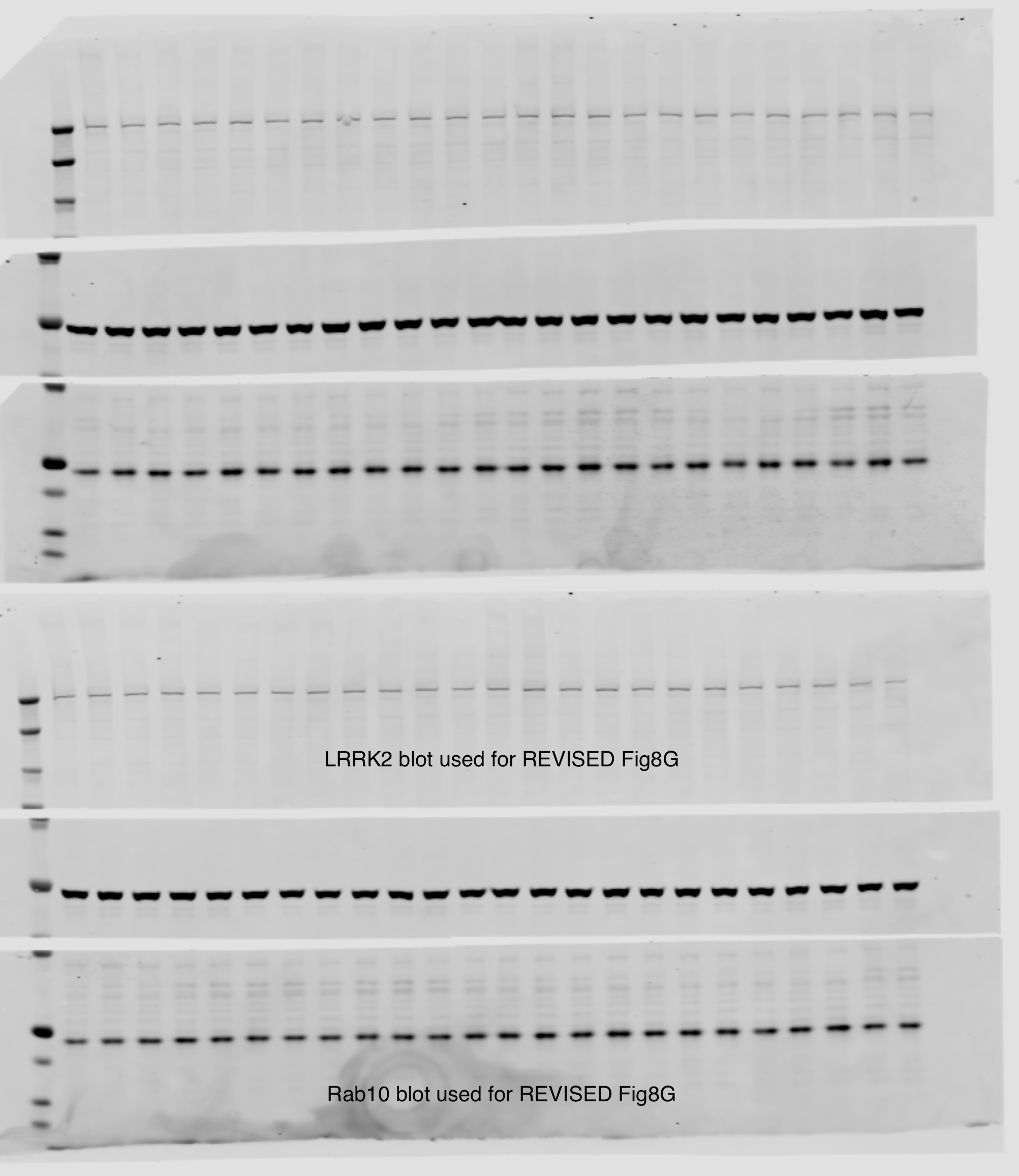

Supplement: Figure 9—source data 1. [file elife-87098-fig9-data1.zip › Figure 9-source data 1/annotated/REVISED-Fig9D_02-03-23_700.tif]

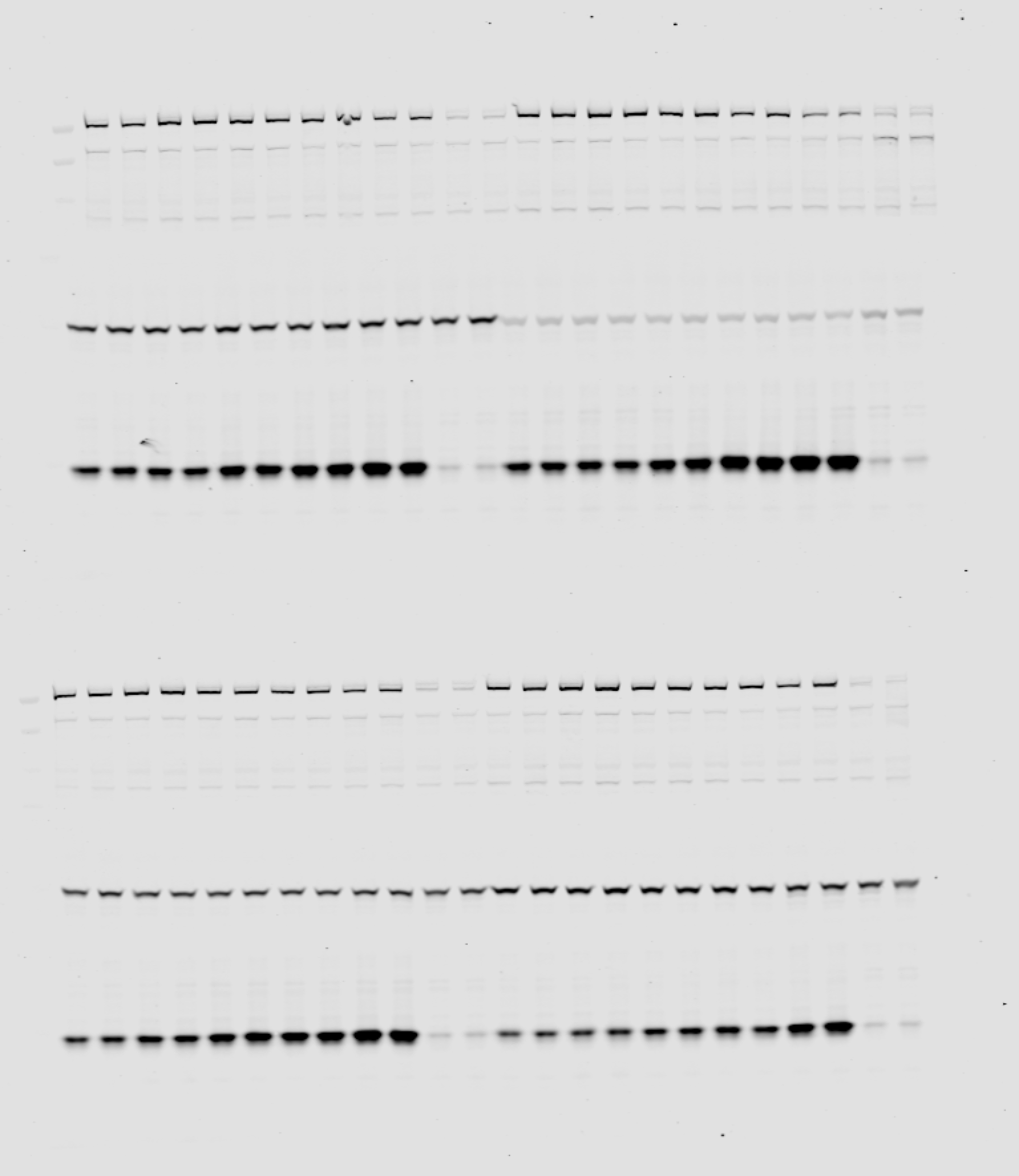

Supplement: Figure 9—source data 1. [file elife-87098-fig9-data1.zip › Figure 9-source data 1/Fig 9D/9D_800.tif]

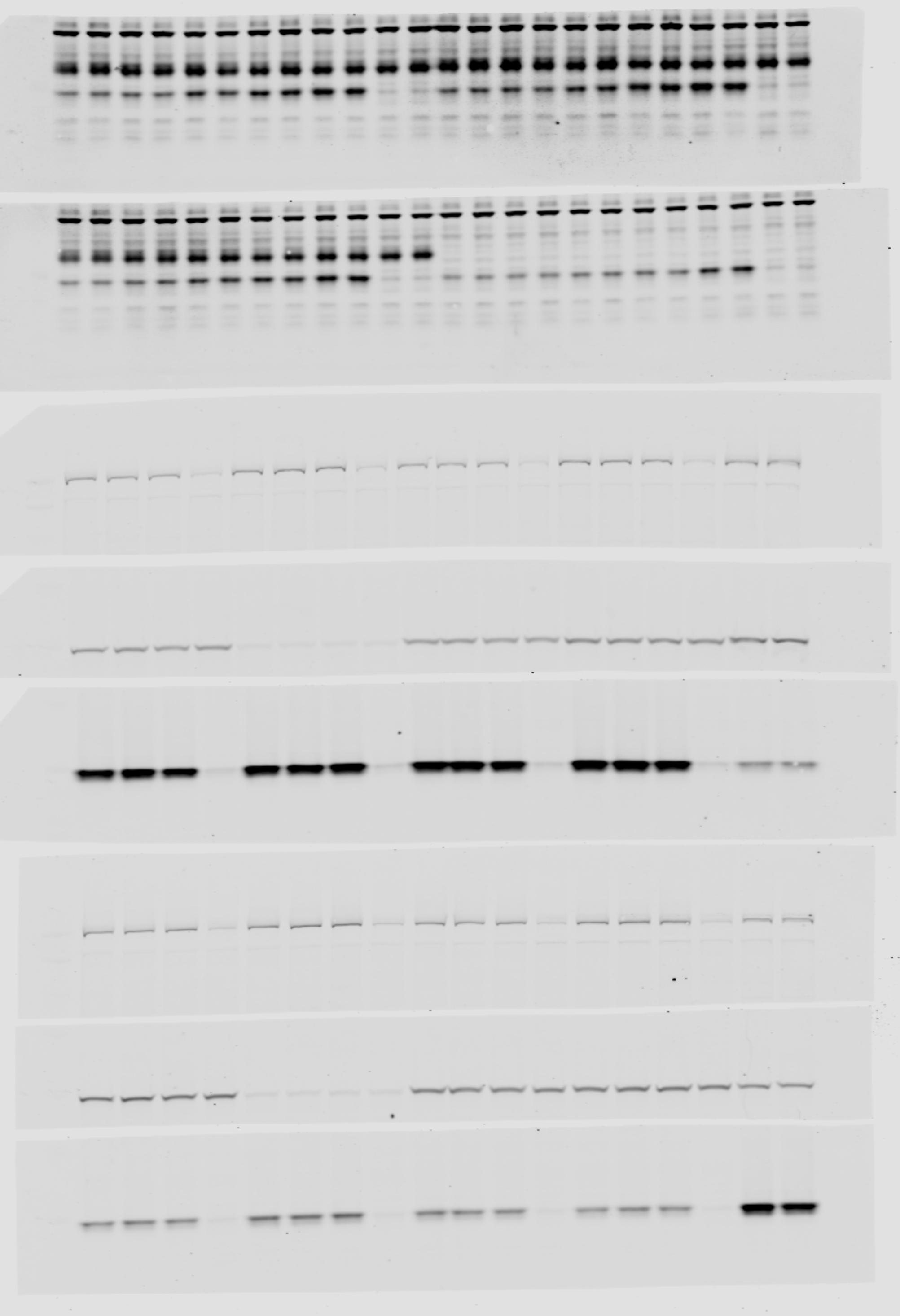

Supplement: Figure 9—source data 1. [file elife-87098-fig9-data1.zip › Figure 9-source data 1/Fig 9D/9D_re-probed_800.tif]
